# Supplementary material for: Development of a theoretical framework of factors affecting patient safety incident reporting: a theoretical review of the literature
Source: BMJ Open. 2017 Dec 27;7(12):e017155. doi: 10.1136/bmjopen-2017-017155 (PMC5770969; doi:10.1136/bmjopen-2017-017155)
Supplement: Supplementary material 1 [file bmjopen-2017-017155supp001.pdf]

eTable1: Full data extraction table of included articles

Author, Year	Study Design, Sample Size, Country	Barriers to Incident Reporting	Facilitators of Incident Reporting	Negative cases (No impact)
Albolino et al., 2010 [(1)]	Questionnaire based-study<br>820<br>Italy	Fear of mistrust in colleagues<br><br>Not considered a priority<br><br>Fear of punishment<br><br>Does not help to improve safety<br><br>Lack of time		
Alsafi et al., 2011 [(2)]	Questionnaire based-study.<br>107<br>Saudi Arabia	Not my responsibility<br><br>I do not want to lose my good relationship with my colleague<br><br>I might be reported by my colleague in turn<br><br>No incentive to error disclose<br><br>Avoiding punishment<br><br>Avoiding damage to reputation<br><br>It will not be discovered		
Anderson et al., 2013 [(3)]	Semi-structured interviews and documentary analysis	Experienced in using IR systems (Mental health staff)		

	62<br>United Kingdom	Blame culture (mental health staff)		
Arfanis et al., 2012 [4]	Semi-structured interviews<br>48<br>United Kingdom	Not used as learning tools to prevent similar occurrences elsewhere.<br><br>Pressures on time<br><br>Resources<br><br>A lack of faith in the established system<br><br>Fruitless and often pointless exercise that has little or no impact on improving patient safety and welfare<br><br>Fear of litigation<br><br>Fear of disciplinary action<br><br>Blame<br><br>The availability and ease of identifying the information<br><br>No feedback	Feedback<br><br>Learning and improvement<br><br>Anonymous web based forum as an add on to IR system	
Armitage et al., 2010 [5]	Semi-structured interviews and retrospective review of error reports<br>40	Lack of feedback		

	United Kingdom			
Ashcroft et al., 2006 [6]	Questionnaire-based Study<br>275<br>United Kingdom	Local reporting<br><br>Good patient outcome less likely to be reported than poor or bad patient outcome.<br><br>Compliance with a protocol less likely to be reported than a violation or error.<br><br>'Fault-led' attitude<br><br>One-off situations by individuals not report<br><br>Loyalty to colleagues<br><br>National reporting system<br><br>Confidence in National Patient Safety Agency	Local reporting<br><br>Poor or bad patient outcome more likely to be reported than good patient outcome<br><br>Violation of protocol or error more likely to be reported than compliance with protocol.<br><br>'Learn from mistakes' culture<br><br>Individuals making continual mistakes<br><br>National reporting system	
Backstrom et al., 2000 [7]	Questionnaire-based study.<br>748<br>Sweden	Assessment that the reaction is already well known<br><br>Forgetting to report		

		Hesitance to report on suspicion<br><br>Lack of time<br><br>Giving preference to other matters<br><br>Uncertainty about the existing rules for reporting<br><br>Difficulty in finding the right form		
Ballangrud et al., 2012 [(8)]	Questionnaire-based study.<br>220<br>Norway	Supervisor/manager expectations, actions promoting safety<br><br>Feedback and communication about error		Organisational learning and continuous improvement<br><br>Teamwork within hospital units<br><br>Communication openness<br><br>Non punitive response to errors<br><br>Staffing
Bateman et al., 1992 [(9)]	Questionnaire-based study.<br>1181	One case cannot contribute to medical knowledge	Should be financially	

	United Kingdom	Impossible to determine responsible drug<br><br>Serious ADRs well known when the drug is marketed<br><br>Professional obligation<br><br>Reporting increases personal liability<br><br>Reporting results by badgering by Committee of safety of medicines<br><br>Takes too much time to ADR report	reimbursed<br><br>Would report if easier method	
Bawazir et al., 2006 [10]	Questionnaire-based study.<br>172<br>Saudi Arabia	No reporting forms available<br><br>Reporting address unknown<br><br>Reporting form too complicated<br><br>Reporting ADRs is too time consuming<br><br>All ADRs are known<br><br>Want to publish myself<br><br>Confidentiality<br><br>Patient confidence	An obligation to do so<br><br>There was a fee<br><br>Saw colleagues doing so<br><br>Attention drawn by publication<br><br>Receiving feedback<br><br>Report through the internet	

		Difficult to admit harm to patient<br><br>Reporting could show ignorance<br><br>Fear of liability<br><br>No motivation<br><br>Insufficient clinical knowledge<br><br>Do not know how to report<br><br>Causality uncertain<br><br>One report make no difference		
Beasley et al., 2004 [11]	Focus groups<br>14<br>United States of America	Punitive system	A feedback system for submitters is necessary to maintain interest.<br><br>Safe and secure access<br><br>There needs to be easy access<br><br>What to report needs to be clearly defined<br><br>The reporting forms	

			must be simple<br><br>Error reporting must fit into a clinicians current work flow<br><br>A non-punitive system is essential<br><br>Reporter should only be required to report once if there are multiple systems	
Belton et al., 1995 (12)	Questionnaire-based study<br>284<br>United Kingdom	Report forms are not available when needed<br><br>Doctor does not like reporting confidential information<br><br>Doctor unsure how to report an ADR<br><br>Doctor fear he/she may appear foolish about reporting a suspected reaction<br><br>Doctor fears he/she may be exposed to legal liability by reporting reaction<br><br>Doctor too busy to send an ADR		

		report<br><br>Doctor is reluctant to admit he/she may have caused a patient harm<br><br>Doctor would rather collect and publish personally<br><br>Doctor believe that only safe drugs are marketed		
Belton et al., 1997 (13)	Questionnaire-based study<br>Sample size not reported<br>International: Denmark, France, Ireland, Italy, Netherlands, Portugal, Spain, Sweden, United Kingdom	Telephone number unavailable<br><br>Report forms unavailable<br><br>Address of reporting agency unavailable<br><br>Unsure how to report<br><br>Patient confidentiality<br><br>Worried about appearing foolish<br><br>Worried about legal liability (Not Denmark or Spain)<br><br>Too busy to report ADRs<br><br>Reluctant to admit they have caused a patient harm		Worried about legal liability (Not Denmark or Spain)<br><br>Ambition to publish a personal series of cases (Not Spain, Sweden or Portugal)<br><br>Patient confidentiality (Not Spain)

		Ambition to publish a personal series of cases (Not Spain, Sweden or Portugal)<br><br>Believes that all marketed drugs are safe		
Blegen et al., 2004 [14]	Questionnaire-based study<br>1105<br>United States of America	Administrative response<br><br>Personal fear<br><br>Quality management<br><br>Staffing resources<br><br>Physical resources<br><br>Peer relations<br><br>Job satisfaction		
Braithwaite et al., 2010 [15]	Questionnaire-based study.<br>2185<br>Australia	IIMS training<br><br>Accessibility of reporting system<br><br>Security of IIMS<br><br>Feedback from reports<br><br>Workplace reporting culture<br><br>Value placed on IIMS		Form of training received

Chang et al., 2012 (16)	Questionnaire-based study<br>183<br>Taiwan		Level of support	Age
Chiang et al., 2006 (17)	Questionnaire-based study.<br>597<br>Taiwan	Being blamed for MAE results<br><br>Adverse consequences from reporting<br><br>Patient's negative attitude<br><br>Physicians' reprimand<br><br>Not recognised MAEs occurred<br><br>Being recognised as incompetent<br><br>Too much time for filling reports<br><br>Think MAEs not important enough to be reported<br><br>Too much time for contacting physicians<br><br>Unclear MAE definition<br><br>Disagreement over MAE<br><br>Unrealistic expectation for administering drugs correctly		

		No positive feedback<br><br>Much emphasis on MAE as nursing quality provided<br><br>Focus on individual rather than system factors to MAEs<br><br>Administrators’ responses to MAEs do not match the severity of the errors		
Chiang et al., 2010 (18)	Questionnaire-based study<br>838<br>Taiwan	Experience of making MAEs<br><br>Nursing professional development<br><br>Fear	Same attitude towards self and co-workers<br><br>MAE reporting rate<br><br>Nursing quality	Age<br><br>Management and leadership<br><br>Administrative barriers<br><br>Reporting process
Chiang et al., 2012 (19)	Questionnaire-based study<br>1049<br>Taiwan		High scores on the safety organising scale<br><br>Tenure of present position<br><br>Self-evaluated IR rates	

			Those more willing to report their own incidents are more likely to report co-workers incidents	
Church et al., 2013 (20)	Questionnaire-based study<br>546<br>United States of America	Hierarchical structure<br><br>Poor communication<br><br>Fear of reprimand<br><br>Reprimand of other therapists and dosimetrists<br><br>Personality<br><br>Lack of reporting system		
Clark et al., 2013 (21)	Questionnaire-based study<br>228<br>International: Australia and New Zealand	Fear of being judged by colleagues<br><br>Personal Guilt<br><br>Feel it as unnecessary<br><br>Near misses are part of life		
Coley et al., 2006 (22)	Focus groups<br>8<br>United States of America	Time consuming<br><br>Inadequate staffing		

Cosentino et al., 1997 [23]	Questionnaire-based study<br>207<br>Italy	Reaction not clinically relevant<br><br>Awareness of similar reactions<br><br>Unavailability of report forms<br><br>Doubtfulness about which ADRs should be reported<br><br>Confidence about ADRs being well documented before marketing<br><br>Ignorance about reporting procedures<br><br>Too much time required to fill in the report form<br><br>Don't feel obliged to report<br><br>Don't want to create undue alarm<br><br>Uselessness of ADR spontaneous reporting		
Covell et al., 2009 [24]	Semi-structured interviews and questionnaire based study<br>50<br>Canada	Adverse consequences		
Daly et al., 2005 [25]	Questionnaire-based study<br>598	Administrators' length of time in position	Directors of nursings'	Administrators' knowledge of

	United States of America	Administrators' and Directors' length of time in facility<br><br>Administrators' length of time in profession<br><br>After internal investigation abuse was thought not to exist<br><br>Told not to report the abuse by my boss<br><br>Reported abuse in the past and IDIA did nothing<br><br>Reported abuse in the past and it led to a bad outcome<br><br>Reported abuse in the past and IDIA ruled it out	knowledge of the law in of nursing<br><br>Administrators' level of education	law<br><br>Administrators' belief that 'elders are able to get help if they need it'<br><br>Age of administrators and directors of nursing<br>Director of nursings' length of time in position<br><br>Director of nursings' length of time in profession<br><br>Director of nursings' level of education<br><br>Administrators' knowledge of the law in nursing
Davies et al., 2012	Focus groups	Lack of feedback		

[26]	19<br>International: United Kingdom/Uganda			
Ehrenpreis et al., 2012 [27]	Questionnaire-based study<br>92<br>United States of America	Unsure how to report appropriately<br><br>Did not see adverse events on a regular basis<br><br>Too busy to make reports<br><br>The existing method was too cumbersome<br><br>Voluntary reporting was not an important process	Easier to use	
Eland et al., 1999 [28]	Questionnaire-based study<br>1357<br>Netherlands	Uncertain association<br><br>Too trivial to report<br><br>Too well known to report<br><br>Unaware of the existence of a nation ADR reporting system<br><br>Unaware of the need to report ADRs<br><br>Did not know how to report ADRs<br><br>Too bureaucratic<br><br>Not enough time		

		Concerned that the report could be used in legal case for damages by the patient<br><br>If another physician had prescribed the medicine<br><br>Medication brought over counter rather than prescribed		
Elder et al., 2007 [29]	Focus groups<br>139<br>United States of America	Burden of effort<br><br>Lack of time<br><br>Forgetfulness<br><br>Information not readily available<br><br>Computer problems<br><br>Online access<br><br>What to report<br><br>Who should report<br><br>What is an AE<br><br>What information is needed<br><br>Common problems	Perceived benefit of reporting – learning and improvement<br><br>Emotional benefit<br><br>Guilt<br><br>Personal responsibility<br><br>Anonymous reporting<br><br>Easing the burden of reporting<br><br>The more harm, the more likely to report	

		Rare errors<br><br>Less serious errors unlikely to be reported<br><br>Feeling personally responsible		
Elder et al., 2008 (30)	Focus groups and questionnaire-based study<br>125<br>United States of America	Too busy with other activities<br><br>Didn't reach the patient<br><br>Risk of harm is none or little<br><br>Error made my someone new-give them a break<br><br>Feel worse emotionally<br><br>Feel like a failure<br><br>Fear punishment<br><br>Blame<br><br>Name on permanent record<br><br>Risk losing friends<br><br>Will make enemies on unit<br><br>No feedback so no personal benefits	Asked by management to make specific reports<br><br>Harm actually occurred<br><br>Risk of harm is great<br><br>Error made by someone unable to be spoken to one-to-one<br><br>Feel better emotionally<br><br>Outlet for irritation at situation or person<br><br>Honesty is a virtue	

			Get a “there but for the grace of god” understanding<br><br>Improve clinical practice<br><br>Could be a learning experience for others<br><br>No known penalty for making a report	
Erler et al., 2013 (31)	Questionnaire-based study<br>51<br>United States of America		Higher levels of teamwork<br><br>Communication openness<br><br>Perception of manager actions promoting safety	
Espin et al., 2010 (32)	Semi-structured interviews<br>37<br>Canada	Did not feel it was an error	Patient negligence<br><br>Threat of potential or actual harm to the patient<br><br>Patient advocacy	

			Following proper procedure<br><br>Error prevention<br><br>Learning opportunities	
Espin, et al., 2007 [33]	Semi-structured interviews<br>13<br>Canada	Domain-specific expertise is a necessary pre-requisite for reporting the error<br><br>Part of the surgeon's responsibility as it fell within the surgical scope of practice.	Events outside of professional boundaries were more likely to be reported<br><br>Responsible for error	
Espin et al., 2006 [34]	Semi-structured and structured interviews<br>28<br>Canada	Responsibility		
Evans et al., 2006[35]	Questionnaire-based study<br>773<br>Australia	I never get any feedback on what action is taken<br><br>I don't feel confident it is kept anonymous<br><br>The incident form takes too long to fill out and I just don't have time<br><br>I am worried about litigation		

		The incident was too trivial<br><br>When the ward is busy I forget to make a report<br><br>It's not my responsibility to report someone else's mistakes<br><br>I don’t know whose responsibility it is to make a report<br><br>I don't want to get into trouble<br><br>When it is a near miss, I don't see any point in reporting it<br><br>Even if I don;t give my details, I am sure that they'll track me down<br><br>The AIMS+ form is too complicated and requires too much detail<br><br>Junior staff are often blamed unfairly for adverse incidents<br><br>I wonder about who else is privy to the information that I disclose<br><br>If I discuss the case with the person involved nothing else needs to be done		

		I don't want the case discussed in meetings<br><br>I am worried about disciplinary action<br><br>Adverse incident reporting is unlikely to lead to system changes<br><br>My co-workers may be unsupportive		
Fairbanks et al., 2008 (36)	Interviews, focus groups and events reports from an anonymous system<br>15<br>United States of America	Blame and Shame<br><br>Punishment<br><br>Legal factors<br><br>Reluctance to tell on colleagues	Non punitive system	
Fukuda et al., 2010 (37)	Questionnaire-based study<br>Sample size not stated<br>Japan		Decreased time for reporting (nurses and physicians)<br><br>Electronic reporting (physicians)<br><br>Attendance at educational seminars (physicians)<br><br>Hospital size	Non-punitive policy (physicians/nurses)<br><br>Rate of recommendations derived from reported incidents (physicians/nurses)<br><br>Electronic

			Ownership – university hospital (physicians)<br><br>Ownership – national hospital (nurses)<br><br>Assignment of patient safety manager (physicians)	reporting (nurses)<br><br>Attendance at educational seminars (nurses)<br><br>Elapsed years of incident reporting system (physicians and nurses)<br><br>Attendance at conference (Physicians/nurses)<br><br>Ward rounds (Physicians/nurses)<br><br>Ownership – university hospital (nurses)<br><br>Ownership – national

				hospital (physicians)<br><br>Ownership – municipal + public hospitals + healthcare corporation + other (physicians/nurse)<br><br>Assignment of patient safety manager (nurses)
Gaal et al., 2010 (38)	Observational study<br>Sample size not stated<br>International: Austria, Belgium, England, France, Germany, Israel, The Netherlands, Slovenia, Switzerland, and Wales		Group (>3) practice	Practice setting<br><br>Amount of responsibility<br><br>Hours of work<br><br>Physical working conditions<br><br>Single+ dual practice
Garbutt et al., 2007	Questionnaire-based study	Private practice	Belief that errors	Perceived risk

(39)	557<br>United States of America		are one of the most serious issues in healthcare<br><br>Belief that they should report serious errors<br><br>Belief that they should report minor errors<br><br>Belief that they should report near misses<br><br>System change to improve patient safety after errors reported<br><br>If error was caused by system rather than individual failures<br><br>Personal involvement in serious errors<br><br>Assurance that the information was	for personal malpractice risk<br><br>Personal involvement in an error

			confidential<br><br>A non-punitive reporting system<br><br>A process that takes less than 2 minutes to use<br><br>Local to the clinician's unit or department	
Generali et al., 1995 [40]	Questionnaire-based study 235<br>United States of America	Unsure drug caused reaction<br><br>Do not have forms<br><br>Do not know how<br><br>Reaction was expected<br><br>Reporting would not occur to me<br><br>Fear of legal liability<br><br>Not my responsibility<br><br>Hours worked per week (>49 or <40)	Hours worked per week (43-49 hours)<br><br>Work setting	Age<br><br>Gender<br><br>Number of years in practice
Gladstone, 1995 [41]	Questionnaires and semi-structured interviews 107<br>United Kingdom	Fear of management reaction		

Green et al., 1999 (42)	Structured interview<br>30<br>United Kingdom	Lack of time/too busy<br><br>Well recognised reaction<br><br>Limited time to spend with patients<br><br>Lack of motivation<br><br>More information about ADR needed<br><br>Lack of confidence in making report<br><br>Patient confidentiality<br><br>Patient suffered an ADR to a product counter prescribed by the pharmacists being interviewed	Certainty of ADR<br><br>Suspicious of a reaction<br><br>Training<br><br>Fee for reporting<br><br>Access to patient records<br><br>Feedback<br><br>More time	
Green et al., 2001 (43)	Questionnaire-based study<br>322<br>United Kingdom	Concern that a doctor gets a copy of reporting form<br><br>Lack of confidence in discussing the ADR with the prescriber<br><br>Apprehension about sending in an inappropriate report<br><br>Lack of time to fill in a report<br><br>Concern that a report will generate extra work	Reaction is of a serious nature<br><br>The reaction is unusual<br><br>The reaction is to a new product<br><br>Certainty that the reaction is a ADR<br><br>The reaction is well	

		The absence of a fee for reporting ADRs<br><br>Lack of time to actively look for ADRs while in clinical practice<br><br>Lack of clinical knowledge makes it difficult to decide whether or not an ADR has occurred<br><br>Don't feel the need to report well recognised reactions<br><br>Reporting cards not available when needed	recognised for a particular agent<br><br>Education/training/ study days or evenings<br><br>More time to spend on wards with patients<br><br>More feedback, reminders and increased awareness<br><br>Encouragement from managers and departments<br><br>Increased collaboration with prescribers and participation on ward round<br><br>Increased accessibility of reporting cards<br><br>Cards specifically designed for the	

			use of pharmacists<br><br>More publicity in journal about reporting scheme<br><br>Online access or telephone based reporting<br><br>Development of local incentives<br><br>Increased confidence in dealing with medical staff<br><br>Making reporting a professional responsibility<br><br>A fee for reporting<br><br>ADR specialist pharmacists<br><br>Increasing awareness among other professionals that pharmacists could report ADRs	

van Grootheest et al., 2002 [(44)]	Questionnaire-based study<br>147<br>Netherlands	Causality uncertain<br><br>Too time-consuming<br><br>No reporting forms available<br><br>Reporting address unknown<br><br>Reporting form too complicated<br><br>All adverse reactions are known<br><br>Want to publish myself<br><br>Confidentiality<br><br>Fear of liability<br><br>No motivation<br><br>Insufficient clinical knowledge<br><br>Do not know how to report	Feedback<br><br>Publications<br><br>Information about the national centre<br><br>Simplification of reporting procedure<br><br>Promoting reporting as part of professional duty<br><br>Financial compensation<br><br>More attention to ADR reporting in university curriculum<br><br>Database of national centre available on the internet<br><br>Compulsory reporting<br><br>Peer reporting	Reporting could show ignorance

Haines et al., 2008 (45)	Questionnaire-based study<br>212<br>Australia	Time<br><br>If the ward is very busy<br><br>Patients' responsibility for adverse events<br><br>Cause of the incident<br><br>Other methods of documentation<br><br>Access to previous reports (non filing of incident reports in the notes)<br><br>Poor user friendliness of computer reporter systems<br><br>Made staff feel personally responsible for the form<br><br>Poor access to computers<br><br>Non reporting by role models<br><br>Absence of a definition of a fall<br><br>Blame<br><br>Absence of training	Staff believe that completing IRs improves patient safety<br><br>Staff belief that competing IRs protects against legal liability<br><br>If the patients was harmed/injured<br><br>Patient factors<br><br>Protect staff<br><br>Type of incident - preventable	

Handler et al., 2007 [46]	Focus group and questionnaire-based study<br>132<br>United States of America	Lack of readily available medication error reporting system or forms<br><br>Lack of information on how to report a medication error<br><br>Lack of feedback to the reporter or rest of facility on medication errors that have been reported<br><br>Lack of knowledge of which medication errors should be reported<br><br>Systems or forms used to report medication error are long and time consuming<br><br>Lack of knowledge of the usefulness of reporting medication errors<br><br>Lack of a consistent definition of a medication error<br><br>Lack of an anonymous medication error reporting system<br><br>Lack of recognition that a medication error has occurred<br><br>Lack of a culture of reporting medication errors		

		Extra time involved in documenting a medication error<br><br>Fear of disciplinary action<br><br>Fear of being blamed<br><br>Fear of liability or lawsuits<br><br>Not knowing who is responsible for reporting a medication error<br><br>Belief that it is unnecessary to report medication errors not associated with patient harm<br><br>Lack of recognition of the actual or potential harm of a medication error<br><br>Belief that reporting medication errors has little contribution to improving the quality of care<br><br>Difficulty in proving that a medication error actually occurred<br><br>Fear of losing respect of co-workers		
Hartnell et al., 2012 (47)	Focus group and semi-structured interviews<br>30<br>Canada	Extra time required to report<br><br>Extra work required to report	Improved care/improved patient safety	

		Cumbersome IR forms<br><br>Hesitancy about 'telling on' someone else<br><br>Fear of loss of reputation/perceived incompetence<br><br>Perceived severity of error (less severe errors are less likely to be reported)<br><br>Inability to recognise or identify medication errors<br><br>Lack of definitions or standards for reporting<br><br>Lack of belief that reporting makes a difference<br><br>lack of trust about how error reports will be used<br><br>Reporting is the responsibility of someone else<br><br>Fear of reprisal from management/administration<br><br>Fear of exposure to malpractice suits	To prevent patient from receiving wrong medication<br><br>Provides immunity/protection from legal action<br><br>Fear of censure (harsh criticism or blame)<br><br>Perceived severity of error (more severe errors are more likely to be reported because a report will be expected)<br><br>Follow rules or policies<br><br>Ensures accountability	

Hasford et al., 2002 (48)	Questionnaire-based study<br>588<br>Germany	ADR too well known<br><br>ADR too trivial<br><br>Uncertain causality<br><br>Reporting too bureaucratic<br><br>Lack of time<br><br>Rules of conduct unknown<br><br>Suspect that drug prescribed by colleague<br><br>Reporting process unknown<br><br>Lack of financial reimbursement<br><br>Suspect drug was self-medication<br><br>Reports considered useless<br><br>Reporting system unknown<br><br>Fear of legal liability<br><br>Non-serious adverse reaction to established drug	Serious unknown ADR to a new drug<br><br>Serious unknown ADR to an established drug<br><br>Serious known ADR to a new drug	
Heard et al., 2012 (49)	Questionnaire-based study<br>433	I am worried about litigation		Generalised de-identified

	Australia	I don’t want to get into trouble<br><br>My colleagues may be unsupportive<br><br>I am worried about disciplinary action<br><br>I may be blamed unfairly for the event<br><br>I do not want to be discussed in meetings.<br><br>Adverse events reporting makes little contribution to quality care<br><br>I don't know whose responsibility it is to make a report<br><br>A good outcome of the case makes reporting unnecessary<br><br>I do not know which adverse events should be reported.<br><br>Even if I don’t give my details I’m worried they will track me down<br><br>The forms take too long to fill in and just don’t have time<br><br>When I am busy I forget to make a report		feedback about reports received from the anaesthetic community<br><br>Role models e.g. senior colleagues and department directors who openly encourage reporting<br><br>Legislated protection of information you provide from use in litigation<br><br>Ability to report anonymously<br><br>Clear guidelines about what adverse events are errors to report<br><br>Information on

		I don’t feel confident that they information I provide will be kept confidential<br><br>I never get any feedback after I report an adverse event<br><br>I wonder about who else will have access to information I disclose<br><br>As long as the staff involved learn from incidents it is unnecessary to discuss them further<br><br>I would protect my self-interests ahead of the interests of the patient if I could (by hiding or denying error)<br><br>Competition with my peers could prevent me from disclosing an error<br><br>If a doctor is careful enough he or she will not make an error<br><br>It would affect my identity as a doctor to admit to an error<br><br>Other don’t need to know about errors I have made<br><br>Disclosing an error, if you don’t have		how confidentiality will be maintained if you supply your name<br><br>Individualised feedback to you about reports you submit<br><br>Paper forms for reporting provided in each theatre<br><br>More support from colleagues<br><br>Less blame attached to those who report errors<br><br>ANZCA continuing professional development point for

		to, is an optional act of heroism<br><br>I would cover up an error I had made if I could<br><br>If I admit to an error I will feel like a failure<br><br>It would affect my self-esteem to admit to an error<br><br>Doctors who make errors are humiliated my their colleagues<br><br>Medicine has a culture of silence where errors are not talked about<br><br>Doctors who make errors are blamed by their colleagues<br><br>Doctors should not make errors.		reports.<br><br>Access to computer based reporting systems for home<br><br>Education about the purpose of reporting<br><br>Computer based reporting systems<br><br>Training on how to use computer based system<br><br>Training on how to fill in papers forms for reporting<br><br>Payment for time taken to report

Herdeiro et al., 2006 [50]	Questionnaire-based study<br>256<br>Portugal	Lack of time<br><br>Complexity of reporting	Workplace (hospital pharmacists more likely to report than community pharmacists)<br><br>Really serious ADRs are not well documented by the time a drug is marketed'<br><br>Serious and not expected ADRs<br><br>Report an ADR if I were unsure that it was related to the use of a particular drug	Gender<br><br>Age<br><br>Job function (registered, assistant or other pharmacists)<br><br>Possible to determine if a drug is responsible for a particular adverse reaction'<br><br>Cannot contribute to pharmaceutical knowledge<br><br>Interested in articles about ADRs'<br><br>Most correct way to report ADRs in is the pharmaceutical

				literature<br><br>Financially reimbursement for providing the ADR service<br><br>Professional obligation to report ADRs<br><br>Reporting ADRs puts career at risk<br><br>I do not have time to complete the report card<br><br>I do not know how the information in the report card is used<br><br>I talk to pharmaceutical companies about possible ADRs with their

				drugs
Hohenhaus et al., 2008 [51]	Questionnaire-based study<br>175<br>United States of America	Afraid to report a medical error they had made<br><br>Afraid to report a medical error made by someone else<br><br>Might not report if there was no harm to the patient and the error was recognised quickly<br><br>Might not report if a physician told them not to report the error<br><br>Would not report if their supervisor told them not to	Error resulting patient harm<br><br>Error by novice nurse	
Holmstrom et al., 2012 [52]	Questionnaire-based study<br>16<br>United Kingdom	Fear of consequences<br><br>Culture of blame<br><br>Lack of training in MER for health-care professionals<br><br>Lack of time for reporting<br><br>Lack of organizational leadership and support<br><br>Lack of legal protection for individual health-care professionals who have	Provides opportunity for evaluating causes of errors (e.g. root cause analysis)<br><br>Uses a non-punitive approach to reporting<br><br>Provides feedback of results of error analysis for those involved in	Paper-based<br><br>Quick and easy to use

		made an error<br><br>Lack of understanding why reporting is needed<br><br>Concern that no beneficial action will follow<br><br>Non-anonymous reporting<br><br>Perceived to be bureaucratic<br><br>Lack of health-care staff<br><br>Lack of financial resources	reporting<br><br>Easy to use<br><br>Provides opportunity for error data analysis<br><br>Produces recommendations and guidelines for improving medication safety<br><br>Provides confidentiality of reported information<br><br>Provided and maintained by one national organisation<br><br>Integral part of patient safety reporting system<br><br>Reporting of errors is voluntary	

			Reporting of errors is mandatory<br><br>Allows all healthcare professionals to report errors<br><br>Available in electronic format<br><br>Independent reporting system dedicated for medication error reporting<br><br>Provides a choice of reporting anonymously<br><br>Includes reporting of both potential and actual errors	
Hutchinson et al., 2009 [53]	Retrospective analysis of routinely collected data and questionnaire-based study<br>Sample size not stated<br>United Kingdom		Employer treats fairly staff involved in error near miss or incident<br><br>Employer encourages staff to	Knows how to report errors, near misses and incidents<br><br>When errors are reported,

			report errors, near misses or incidents<br><br>Employer treats reports of errors, near misses or incidents confidentially<br><br>Employer does not blame or punish people who make errors.<br><br>Access to a counselling service were also more likely to report.<br><br>Previous reporting behaviours<br><br>Level of risk management	employer takes action to ensure that they do not happen again
Irujo et al., 2007 [54]	Case control study<br>78<br>Spain	Not serious ADR<br><br>Already well known ADR<br><br>Uncertain about causality<br><br>Forgot to report		Age<br><br>Working experience as pharmacist<br><br>Participation in

		Lack of time		a programme for detection and resolution of DRPs<br><br>Education on detection and resolution of DRPs<br><br>Frequently considering the possibility of finding an ADR when attending a patient with symptoms<br><br>Forgetting to report<br><br>Education for ADR reporting<br><br>Awareness of the importance of reporting system<br><br>It is necessary to be sure that the reaction is

				causally related to the use of a particular drug<br><br>Basic knowledge about ADR reporting
Jeffe et al., 2004 (55)	Focus groups<br>109<br>United States of America	Not knowing what to report<br><br>Errors that pose little risk to the patient<br><br>Errors that do not end up harming the patient<br><br>Not knowing how to report<br><br>Fear of disciplinary repercussions (nurse and physicians)<br><br>Fear of legal repercussions (nurse and physicians)<br><br>Fear of repercussions from doctors (nurses)<br><br>Link between reporting and performance reviews (nurses)	Severity of the situation (nurses)<br><br>Likelihood of reoccurrence (nurses)<br><br>Severe events reported as the error would be 'found' out anyway<br><br>Self-protection<br><br>The importance of reporting errors for educational purposes<br><br>Anonymous (physician and nurses)	

		Protecting colleagues from disciplinary action(nurses)<br><br>Lack of confidentiality<br><br>Name, blame, shame culture<br><br>Fear of public exposure<br><br>Staff shortages<br><br>Lack of time<br><br>The lack of simple procedure for reporting errors<br><br>Lack of feedback	Simple (physician and nurses)<br><br>Fast reporting procedures(physician and nurses)<br><br>Receipt of critical feedback about the errors<br><br>Anonymous, phone in system (physicians)<br><br>Educational rather than punitive system (physicians)<br><br>System that was 'lawyer proof'<br><br>Blame free reporting (nurses)	
Jennings et al., 2011 [56]	Focus groups, interviews and questionnaire based study<br>Sample size not stated<br>Australia	Burden of reporting in terms of time<br><br>Lack of accessibility of reporting forms	Clarity of indemnity from prosecution	

		Time elapsed following incident<br><br>Priority of reporting over other work tasks<br><br>Forgetting to report<br><br>Workload<br><br>Fear of disciplinary action<br><br>Fear of potential litigation<br><br>Fear of breaches of confidentiality/anonymity<br><br>Fear of embarrassment within peer group<br><br>Fear that incidents many impact on their likelihood of promotion<br><br>Concern that nothing would change even if the incident was reported<br><br>Lack of familiarity with process		
Johnstone et al., 2008 [57]	Focus groups, semi-structured interviews and questionnaire-based study 35	Frequency of incident-more frequent less likely to report	Seniority of graduate nurses	

	Australia			
Joolaee et al., 2011 [58]	Questionnaire-based study<br>286<br>Iran			Perceived work conditions
Kagan et al., 2008 [59]	Questionnaire-based study<br>201<br>Israel	The practice of ward nurse managers to cover up error, that is dealing with the error themselves without reporting to a higher authority	How the ward's and hospital's dealt with medication error<br><br>How their ward handles error reporting	
Kagan et al., 2013 [60]	Questionnaire-based study<br>247<br>Israel	Medical error incidence	Patient safety culture index<br><br>PSC at organisational level<br><br>PSC at departmental level<br><br>PSC at respondents personal performance level<br><br>Nurses' place of birth and their professional status (academic or non-academic	

			registered nurse)	
Kaldjian et al., 2009 (61)	Questionnaire-based study<br>338<br>United States of America		Feedback	
Karsh et al., 2006 (62)	Focus group<br>14<br>United States of America	Length of report<br><br>Punishment<br><br>Reporting near misses	Feedback<br><br>Mandatory system<br><br>Financial incentives<br><br>Other incentives (protection from malpractice and disciplinary action)<br><br>Support in using system<br><br>Education in using system	
Kennedy et al., 2004 (63)	Questionnaire-based study<br>113<br>United States of America	Not their responsibility to report<br><br>Never thought to report/not required to do so<br><br>Handle errors internally i.e. no corporate system<br><br>No errors worth reporting		

		No time to report<br><br>Forms not available or convenient		
Khan, 2013 [64]	Questionnaire-based study<br>50<br>Saudi Arabia	Unavailability of professional environment to discuss ADR<br><br>Reporting forms are not available<br><br>I do not know how to report<br><br>Reporting forms are too complicated<br><br>Reporting is time consuming<br><br>I am not motivated to report<br><br>I fear legal liability of the reported ADR<br><br>I am not confident whether it is an ADR<br><br>Insufficient knowledge of pharmacotherapy in detecting ADR<br><br>Belief that only safe drugs are marketed-not cause of reaction		
King et al., 2006 [65]	Questionnaire-based study<br>39	Time constraints		

	United States of America	Difficulty locating forms<br><br>Lack of closure/feedback<br><br>Not important<br><br>Fear of disclosure to risk management		
Kingston et al., 2004 [66]	Focus groups<br>33<br>Australia	Lack of knowledge about the reporting process and<br><br>Lack of knowledge about what constitutes an incident<br><br>"Nursing form" by association (not identified as being part of doctors role)<br><br>Time constraint<br><br>Complexity of reporting form<br><br>Lack of feedback<br><br>Lack of legal privileges afforded to the reporting process<br><br>Culture of blame<br><br>No value	Effective and efficient IRS<br><br>IRS with threat or blame<br><br>Prompt, relevant feedback<br><br>IRS that drive improvements<br><br>Monetary payment<br><br>Simplification<br><br>Less time consuming<br><br>Clear definitions of what constitutes an adverse event/near-miss	

			Evidence of value of IRS<br><br>Reporting process to be made more relevant to doctors<br><br>Reporting process less threatening by renaming the form<br><br>Increased awareness and knowledge of IR process<br><br>Protection from liability<br><br>System that doesn’t require input from doctors (nurses)<br><br>Education at orientation (nurses)<br><br>Anonymous reporting	
Kreckler et al.,	Questionnaire-based study	I am too busy to fill out the form		

2009 [67]	137<br>United Kingdom	The form takes too long to complete<br><br>I am worried about litigation<br><br>I do not want the case discussed in meetings<br><br>I never get any feedback<br><br>It makes little contribution to the quality of care<br><br>I am not sure what incidents to report<br><br>The incident was too trivial<br><br>The incident did not result in any harm		
Li et al., 2004 [68]	Questionnaire-based study<br>1653<br>China	Address of reporting agency not available<br><br>Report forms unavailable<br><br>Reporting process unknown<br><br>Unaware of a national ADR reporting system<br><br>Patient confidentiality	Increasing awareness among administrators, doctors & nurses<br><br>Establishing ADR institutes<br><br>Education and training in ADR knowledge and related topics	

		Too busy to report ADR<br><br>ADR sufficiently well documented<br><br>Reluctant to admit that they have caused a patient harm<br><br>Worried about feeling foolish<br><br>Reluctant to admit they may have made a medical error<br><br>Personal ambition to publish a case study		
Martowirono et al., 2012 [(69)]	Focus group<br>22<br>Netherlands	Negatively valued<br><br>Costs time<br><br>Perceived as another administrative task that they have to complete<br><br>Priority<br><br>Do not always agree with the definition of incident<br><br>Incidents that had no major patient consequence<br><br>Incidents that have happened before and has already been reported	Reporting process- ability to report over the phone or send an email<br><br>Anonymous reporting<br><br>Provide the possibility to report without identifying the person involved<br><br>Provide feedback<br><br>Provide feedback to the reporter if an	

		Incidents that was not preventable<br><br>The cause of the incident Is already clear<br><br>Incidents is unlikely to happen again<br><br>Was not an incident but a complication<br><br>Incident already been discussed with the people involved<br><br>The lack of feedback on a report<br><br>Absence of visible system changes were also issues<br><br>Disloyal to colleagues<br><br>Not their responsibility<br><br>legal liability<br><br>Unpleasant working conditions<br><br>Lack of encouragement from superiors to report incidents.<br><br>Incident reporting is emotionally charged	incident on how the report will be handled<br><br>Feedback- communicate the results in terms of systems changes<br><br>Create an incident reporting culture<br><br>Create a culture in which IR is less emotionally charged e.g. by systematically discussing IR within a ward and stimulating role of supervisors<br><br>Simplify the procedure<br><br>Design a procedure in which it is possible to only report the essentials of an incident, e.g. by making a call or	

		Some residents stated that they did not complete IR because they did not think of it whereas others said<br><br>Did not know what to report.<br><br>Did not know how to report<br><br>IRS complicated<br><br>Workload	filing out a card or compact form with standard incidents. If necessary, the resident can be contacted for more information<br><br>Make it easy for a resident to find out if an incident has already been reported<br><br>Clarification what to report<br><br>Clarification about and how to report<br><br>Excite residents to report<br><br>Draw attention to IR e.g. putting up posters with a catchy slogan	
Mayo et al., 2004 (70)	Questionnaire-based study<br>983<br>United States of America	Afraid of manager reaction<br><br>Afraid of co-workers' reactions		

		Not thinking an error was serious enough<br><br>Fear of disciplinary action		
McArdle et al., 2003 (71)	Semi-structured interviews<br>15<br>United Kingdom	It takes too long<br><br>Lack of feedback received<br><br>Lack on incentive<br><br>Cumbersome<br><br>Non-anonymous<br><br>Fear of blame<br><br>Description of medication did not fall into IRS formats-scope of reporting		
Merchant et al., 2005 (72)	Questionnaire-based study<br>207<br>Canada	I think of reporting too late<br><br>Don’t know where CIRS forms are<br><br>Fear of lawyers getting information<br><br>I don't know what sort of incident to report<br><br>I'm too busy<br><br>Fear of record of problem		Unnecessary as anesthesia is safe<br><br>futile as anesthesia is safe

		Don't have CIRS forms<br><br>My incidents are too minor<br><br>Too long<br><br>No value will come of this<br><br>Too much writing<br><br>Incidents I see are other's problem<br><br>Too many tick boxes<br><br>Unsure what 'critical incident' is<br><br>Effort is doomed to failure<br><br>Too difficult<br><br>Form is confusing<br><br>Unimportant to me<br><br>Nothing can be learned from me<br><br>CIRS asks wrong questions		
Mrayyan et al., 2007 (73)	Questionnaire-based study<br>779<br>Jordan	Fear of disciplinary action/lose job<br><br>Errors not serious to warrant		

		reporting<br><br>Fear of reaction from co-workers<br><br>Fear of reaction from nurse managers		
Mustafa et al., 2013 [74]	Questionnaire-based study<br>136<br>Pakistan	Uncertain association<br><br>Awareness<br><br>Concern about legal liability	Seriousness of ADRs<br><br>Unusual reaction<br><br>Reaction to a new product<br><br>Confidence in the diagnosis of ADR	
Naveh et al., 2006 [75]	Questionnaire-based study<br>632<br>Israel	Perceived safety procedures	Perceived safety information flow	Perceived priority of safety<br><br>Unit type
Okuyama et al., 2010 [76]	Questionnaire-based study<br>430<br>Japan		Safety management at ward level	Safety management at the hospital level<br><br>Attitudes of ward safety managers

Osborne et al., 1999 (77)	Questionnaire-based study<br>57<br>United States of America	Error not serious<br><br>Afraid of repercussions<br><br>Afraid of reactions from managers/co-workers		Perceptions of medication errors
Parvizi et al., 2014 (78)	Questionnaire-based study<br>119<br>United Kingdom	Did not know they were expected to do this<br><br>Did not know how to report to MHRA<br><br>I do not see the purpose of reporting<br><br>Lack of time<br><br>Blame<br><br>Direct reporting to the manufacturer<br><br>Not reporting if the types of device failure were considered to be common knowledge<br><br>Reporting only those that were unexpected failures or failures that may affect the patient or user<br><br>Reported by either a nurse or other doctor	Better education of the means of adverse IR<br><br>Improvements in the feedback sent to the reporter on the outcomes of the adverse incidents<br><br>Improvements in the guidance on the type of adverse device related incidents to report<br><br>Improvements in the electronic means of adverse IR<br><br>Improvements in the clinical and	

			adverse incidence governance	
Patrician et al., 2009 [79]	Questionnaire-based study<br>43<br>United States of America	Perceptions that the administration focuses on the individual and not the system<br><br>Nurses are blamed when something bad happened to patients<br><br>Fear adverse consequences for reporting errors<br><br>Nurses believe that their peers will think them incompetent<br><br>Nurses do not think the error was important enough to report<br><br>Fear of administrative response<br><br>Disagreement over error<br><br>Reporting effort<br>Lack of agreement about definition of error<br><br>Lack of error recognition<br>Excessive length of time for contacting physician		
Rasmussen et al.,	Questionnaire-based study		Safety climate	

2014 [80]	124<br>Denmark		Team climate<br><br>Inter-departmental working relationships<br><br>Increased cognitive demands	
Rogers et al., 1988 [81]	Questionnaire-based study<br>1121<br>United States of America	Reporting forms not available<br><br>Event already documented<br><br>Did not get to it/got busy<br><br>Did not believe it was important<br><br>Forms were too much trouble<br><br>Minor or expected side effect<br><br>Did not like interacting with the government<br><br>Liability concerns<br><br>Did not know how to report<br><br>Undetermined as ADE<br><br>Not primary physician		Age<br><br>Time in direct patient care

Rowin et al., 2008 [82]	Descriptive study<br>Sample size not stated<br>United States of America		More likely to report no harm (nurses)<br><br>More likely to report permanent harm, near death, death and unsafe environment (doctors)<br><br>Type of incident: falls and medication (nurse)<br><br>Type of incident: adverse clinical event (doctors)	Temporary harm<br><br>Near miss
Sanghera et al., 2007 [83]	Semi-structured interviews<br>13<br>United Kingdom	Not being aware that an error had occurred<br><br>Detailed paperwork<br><br>Time constraints<br><br>Not understanding incident reporting process<br><br>No benefit (perception that nothing is done with the data)		

		No encouragement by management<br><br>Fear of loss of professional registration<br><br>Fear of being in trouble<br><br>Fear of looking incompetent<br><br>Feeling upset<br><br>Fear will be blamed<br><br>Not wanting to report colleagues' errors		
Sarvadikar et al., 2010 (84)	Questionnaire-based study<br>56<br>United Kingdom		Doctors more likely to report errors with worsening patient outcome	Nurses and pharmacists likely to report error regardless of patient outcome
Schectman et al., 2006 (85)	Questionnaire-based study<br>120<br>United States of America	Unsure of reporting mechanism<br><br>No actual harm came to the patient<br><br>Reporting too difficult and time consuming<br><br>Unsure of what is considered AE/NM	Allow electronic reporting of adverse events and near misses<br><br>Clarify reporting mechanism	

		Inadequate MD participation in scheme<br><br>Concern about consequences of reporting others' error<br><br>Reporting makes no difference (nothing will change)<br><br>Concern about being blamed or judged less competent<br><br>Weaknesses in the reporting system<br><br>Professional behaviours<br><br>Fear of retribution<br><br>Lack of feedback and the perception that change would not result from reports.	Clarify what constitutes an AE/NM<br><br>Allow anonymous reporting<br><br>Increase physician involvement in QI<br><br>Provide feedback on QI projects arising from reports<br><br>Provide individual feedback following report<br><br>Provide summary feedback on a regular basis<br><br>Make reporting mandatory	
Schulmeister et al., 1999 [86]	Questionnaire-based study<br>160<br>United States of America	Minor error<br><br>Fear of disciplinary action		
Sharma et al., 2008 [87]	Questionnaire-based study<br>81<br>United Kingdom	Does not achieve anything<br><br>Not in physicians culture	Anonymous system<br><br>Easily accessible	

		Do not wish to incriminate others<br><br>Do not know how to access forms<br><br>Not bothered<br><br>Do not wish to ask nurse staff<br><br>Lack of time<br><br>Do not know which incidents need to be reported<br><br>Lack of anonymity<br><br>Not in habit of considering it<br><br>Discouraged by senior nurses	forms<br><br>Forms not held by nursing staff	
Soberberg et al., 2009 (88)	Questionnaire-based study<br>317<br>Sweden	I did not have enough time<br><br>I am concerned about possible consequences<br><br>Someone else did it<br><br>It is too complicated<br><br>No one else files incident reports<br><br>It would not make any difference		

		Insufficient routines for reporting		
Soleimani., 2006 (89)	Questionnaire-based study<br>128<br>New Zealand	Threat of public outcry<br><br>Professional consequences/discipline<br><br>Embarrassment in front of colleagues		
Stratton et al., 2004 (90)	Questionnaire-based study<br>284<br>United States of America	No positive feedback is given for passing medications correctly<br><br>Nurse administration focuses on the person rather than looking at the system<br><br>Too much emphasis is placed on medication errors as a measure of the quality of care<br><br>Responses by nursing administration do not match the severity of the error<br><br>Individual/personal reasons<br>Nurses could be blamed if something happened to the patient<br><br>Nurse believe other nurses will think they are incompetent		

		Nurses fear adverse consequences from reporting<br><br>Patient might develop a negative attitude<br><br>Nurses fear reprimand from physician<br><br>Nurses fear losing their license<br><br>Nurses want to avoid potential publicity of medication errors in the media		
Sweis et al., 2000 (91)	Questionnaire-based study<br>280<br>United Kingdom	Busy<br><br>Legal liability<br><br>Fear of breaching patient confidentiality	Serious ADR rather than trivial<br><br>Rarely occurring ADR rather than common ADR<br><br>Confidence in recognising an ADR<br><br>ADR to an established drug rather than new drug<br><br>Active support of	Training in reporting<br><br>Gender<br><br>Type of hospital<br><br>Age

			medical/pharmacy staff<br><br>Written hospital policy for pharmacist ADR reporting<br><br>Training and ADR meeting<br><br>Increasing seniority<br><br>Allocation of time for ADR monitoring<br><br>Publicity and promotion by hospital and CSM<br><br>Better cooperation with clinicians<br><br>Support and encouragement by the pharmacy department<br><br>More ward rounds and direct patient contact	

			Simplify reporting system<br><br>ADR reporting team<br><br>Feedback	
Tariq et al., 2012 (92)	Semi structured interviews<br>23<br>Australia	Lack of time		
Taylor et al., 2004 (93)	Questionnaire-based study<br>140<br>United States of America	Not important to report error that did not harm patient<br><br>Reporting errors does not make any difference<br><br>Unsure about what is considered medical<br><br>Incident report form too complicated<br><br>Concerned about being blamed or judged incompetent<br><br>Concerned about implicating others<br><br>Unsure whose responsibility it is to report errors	Make reporting of errors mandatory<br><br>Different format for IR<br><br>Use of electronic format for reports<br><br>Reward for reporting medical errors<br><br>Better education about what is considered a medical error that should be reported<br><br>Evidence that reporting of errors	

			led to system changes<br><br>Feedback on regular basis and frequencies of reported errors<br><br>Feedback regarding outcome of a specific error that has been reported	
Throckmorton et al., 2007 (94)	Questionnaire-based study<br>435<br>United States of America	Level of harm: no harm	Level of harm<br><br>Working closely to the patient<br><br>Higher scores on the Wakefield's scale<br><br>Fewer years since initial license	
Tobaiqy et al., 2013 (95)	Questionnaire-based study<br>61<br>Saudi Arabia	Lack of awareness<br><br>Workload/time constraints<br><br>Unavailability of reporting form	Continuing education events<br><br>An internet/web based reporting facility	

		Reporting system complexity<br><br>Error too trivial<br><br>Lack of anonymity<br><br>Fear of blame<br><br>Concerns over penalisation<br><br>Difficulty in recognising errors<br><br>Senior staff advised not to report<br><br>Lack of feedback from authority	Training focused on error prevention<br><br>Anonymity of reporting<br><br>A non-punitive reporting culture<br><br>Financial incentives linked to reporting	
Turner et al., (2013) [96]	Semi-structured interviews<br>32<br>United Kingdom	Value-not convinced that the reporting system would deliver improvements in clinical care		
Uribe et al., 2002 [97]	Questionnaire-based study<br>122 United States of America	Time involved in documenting an error<br><br>Extra work involved in reporting<br><br>Hesitancy regarding 'telling' on somebody else<br><br>Thinking that it is unnecessary to report error because it had no negative outcome		Thinking that reporting has little contribution for improvement of quality care<br><br>Not knowing the usefulness of the report<br><br>Lack of

		Not being able to report anonymously<br><br>Fear of lawsuits		knowledge of what should be reported<br><br>Lack of recognition that a medical error has occurred<br><br>Fear of being blamed<br><br>Fear of disciplinary action/ losing job<br><br>Lack of information in how to report<br><br>Lack of interest or motivation for reporting<br><br>Forms or computer locations not available to report medical errors

				Not knowing who is responsible for reporting error
Vessal et al., 2009 (98)	Questionnaire-based study<br>110<br>Iran	Uncertain association<br><br>Too trivial to report<br><br>Too well known to report<br><br>Yellow card not available<br><br>Not enough information from the patient<br><br>Not enough time<br><br>Unaware of the existence of a national ADR reporting system<br><br>Too bureaucratic<br><br>Did not know how to report<br><br>Fear of legal liability<br><br>Unaware of the need to report and ADR	The reaction is of a serious nature<br><br>The reaction is unusual<br><br>The reaction is to a new product<br><br>Reaction not reported before for a particular drug<br><br>Reaction is well recognised for a particular drug<br><br>Any reaction	
Vincent et al., 1998 (99)	Questionnaire-based study<br>198	Unnecessary		Unsupported colleagues

	United Kingdom	Increased workload<br><br>Blame<br><br>Worry litigation<br><br>Busy/forgot		Not knowing which incidents to report<br><br>As long as staff learn from incident it is unnecessary to discuss/report<br><br>Fear disciplinary<br><br>Not wanting incident to be discussed<br><br>Who's responsibility<br><br>Little contribution
Vogus et al., 2007 (100)	Questionnaire-based study<br>1033<br>United States of America	Safety organising<br><br>Unit type (emergency)<br><br>Safety organising and trust<br><br>Safety organising and pathways	Trust in managers<br><br>RN experience<br><br>Unit type (IC)<br><br>Number of beds	Care pathways<br><br>% of RNs with BSN<br><br>Unit type (surgery)

		Patient-to-RN ratio		
Walji et al., 2011 (101)	Semi- structured interviews<br>12<br>Canada	Lack of knowledge about natural health products<br><br>Lack of time/priorities<br><br>Complexity of reporting process	Pharmacists who saw themselves as ‘knowledge generators’ rather than just ‘knowledge users’ were more likely to report and less likely to allow workplace challenges to prevent their taking an extra step	
Walker et al., 1998 (102)	Focus groups and questionnaire-based study<br>43<br>Australia	Minor incidents (documentation and minor variation from the prescription)<br><br>Negative past experience of reporting<br><br>Fear of getting into trouble<br><br>Fear they will somehow stand out from the crowd in the eyes of those in authority<br><br>Feelings of discomfort or uncertainty about being required to report an incident that involved a colleague	More likely to report an incident if patient safety compromised<br><br>Capacity to feedback and improve the situation<br><br>Reporting might help raise people's awareness of problems that could be occurring	Fear of possible punishment senior staff

		This is more difficult if the colleague is a more experienced nurse<br><br>Others expressed with view that they wouldn’t report a friend, perhaps perceiving that the friend would be in trouble if the incident was reported<br><br>Did not always want to admit their mistake<br><br>Might not even realise that an error had occurred<br><br>Incident might be highly incriminating<br><br>If the patient actually came to harm as a result of the error<br><br>If the departure from the prescribed therapy seemed reasonable<br><br>If the problem could be sorted out<br><br>Concern about the time taken to fill in the incident report form<br><br>Inadequate understanding of what constituted an error<br><br>A lack of feedback on the number of medication errors was a problem	Wrong drug<br><br>Wrong route<br><br>Wrong person<br><br>Wrong dose<br><br>Harm to the patient<br><br>A desire to target an individual or professional group to improve practice<br><br>Legal obligation of the nurse to report	

		Perceived inaction on reported errors incidents		
Waring, 2004 (103)	Semi- structured interviews<br>37<br>United Kingdom	Acute medicine and rehab: IR system was regarded as nurse led, dealing with ward issues and the work of non-medical groups<br><br>Anaesthesia: Physicians remained sceptical about the hospital wide reporting system and were generally disinclined to participate in this approach		
Waring, 2005 (104)	Semi-structured interviews<br>28<br>United Kingdom	Fear of blame<br><br>Blame culture<br><br>Peer of punishment<br><br>Fear of blame from pubic<br><br>Fear of litigation<br><br>Fear of professional competence being questioned<br><br>Fear of poor references<br><br>Reprimands from a senior colleague		

		Fear of use of reports-could be used at a later date in the event in medico-legal disputes		
Waters et al., 2012 (105)	Focus groups<br>16<br>Canada	Time<br><br>Fatigue<br><br>High workload<br><br>Relevance of reporting form<br><br>Complexity of reporting-gathering many pieces of information.<br><br>Unit culture<br><br>Fear of blame<br><br>Close knit team<br><br>Other methods of reporting-verbal reporting and team debrief<br><br>Lack of feedback	Previous experience of litigation<br><br>Protection against future litigation<br><br>Professional responsibility<br><br>IR perceived as learning opportunity<br><br>Desire for practice improvement	Risk of litigation
Weissman et al., 2005 (106)	Questionnaire-based study<br>203<br>United States of America	Mandatory<br><br>Non-confidential system<br><br>State run	Serious harm	

		Less harm		
Williams et al., 2013 [107]	Focus groups<br>17<br>United Kingdom	Severity (more likely to report if serious harm	Simpler reporting system<br><br>Targeted report<br><br>Feedback<br><br>Drug-specific error reporting forms<br><br>Electronic forms/systems (easier than paper)<br><br>Anonymous reporting	
Winchester et al., 2012 [108]	Questionnaire-based study<br>120<br>United Kingdom	Concerned about confidentiality<br><br>Did not know the procedure for reporting<br><br>Did not think anything could be done<br><br>Did not feel incident was important enough to report<br><br>Believed source to be low risk<br><br>Reporting was inconvenient	Education<br><br>Adverts/posters<br><br>Training<br><br>Compulsory reporting<br><br>Simple reporting system<br><br>An electronic	

			reporting system	
Yong et al., 2003 (109)	Questionnaire-based study<br>136<br>New Zealand	Time constraints<br><br>Laziness and forgetfulness<br><br>Dislike form filling<br><br>A lot of work for little practical benefit<br><br>Forms too complicated<br><br>Do not believe the system is working<br><br>Many incidents not worth reporting<br><br>Many other tools exist for correcting errors and improving standards<br><br>Dislike the published interpretation of results with diagnostic views by some anaesthetists<br><br>Qualitative result not acceptable<br><br>Feel that the main benefit of IR is local analysis and that very rare events distilled by multi-site monitoring are less important<br><br>Difficulty defining what constitutes incident	Total anonymity and confidentiality<br><br>Protection against punitive action<br><br>Simplify forms and bring up to date<br><br>Easy access to forms<br><br>Electronic data entry<br><br>Incorporating IR form filling at regular M&M meetings<br><br>Mandatory<br><br>Local analysis rather than Australasian wide<br><br>More aggressive follow up and reviewing	

		Inadequate feedback<br><br>Medico-legal implications<br><br>Forms not available/hard to locate<br><br>Lack of appropriate culture within department<br><br>Not accepted as part of private practice culture<br><br>Use of local IR system, hospital based audit<br><br>Incidents are discussed at department level confidentially	Publication of problems<br><br>Aims and purpose should be clarified explicitly<br><br>Select a few incidents to monitor frequency	
Zwart et al., 2011 (110)	Prospective cohort study<br>66<br>Netherlands		Expertise	Communicator<br><br>Collaborator<br><br>Manager<br><br>Health advocate<br><br>Scientist<br><br>Professional

Adverse Drug Event (ADE); Adverse Drug Reaction (ADR); Adverse Event (AE); Australia and New Zealand College of Anesthetists (ANZCA); Bachelor of Science in Nursing (BSN); Critical Incident Reporting Service (CIRS); Drug related problems (DRP); Incident Reporting (IR); Iowa Department of Inspections Appeals (IDIA); Incident Information Management System (IIMS); Intensive Care (IC); Medication Administration Error (MAE); Medication and Healthcare Products Regulatory Agency (MHRA); Medical Doctor (MD); Morbidity and Mortality (M&M); Near Miss (NM); Patient Safety Culture (PSC); Quality Improvement (QI); Register Nurse (RN)

eTable 2: Frequency of factors influencing engagement in incident reporting

		Impact on Reporting Engagement		
**Factor**		**Barrier** Frequency Count *(%)*	**Facilitator** Frequency Count *(%)*	**Negative Case (no impact)** Frequency Count *(%)*
Fear of Adverse Consequences	Adverse consequences	51 *(31.68%)* (1, 2, 4, 11, 14, 17, 18, 20, 21, 23-25, 30, 35, 36, 43, 46, 47, 49, 51, 52, 55, 56, 62, 65, 69-71, 77, 79, 83, 85, 86, 88, 90, 95, 102, 104)	-	3 *(25.00%)* (16, 99, 102)
	Litigation	30 *(18.63%)* (4, 9, 10, 12, 13, 28, 35, 36, 40, 44, 46-49, 55, 56, 64, 66, 67, 69, 72, 74, 81, 91, 97-99, 102, 104, 109)	8 *(61.54%)* (45, 47, 55, 56, 62, 66, 105)	4 *(33.33%)* (13, 39, 97, 105)
	Blame	24 *(14.91%)* (3, 4, 17, 24, 30, 35, 36, 45, 46, 49, 52, 71, 78, 79, 83, 85, 90, 93, 95, 99, 104, 105)	4 *(30.77%)* (47, 49, 55, 66)	1 *(8.33%)* (97)
	Judgment	22 *(13.66%)* (2, 10, 12, 13, 17, 21, 24, 41, 46, 47, 70, 73, 79, 83, 89, 90, 104)		1 *(8.33%)* (44)
	Relationships	12 *(7.45%)* (1, 2, 20, 24, 55, 77, 85, 89, 90, 93, 97, 104)	-	-
	Impact on career	10 *(6.21%)* (15, 24, 30, 55, 56, 72, 73, 83, 90, 104)	-	1 *(8.33%)* (50)
	Protection of self	7 *(4.35%)* (10, 12, 13, 42, 68, 80)	-	-
	Avoid discussion in meetings	4 *(2.48%)* (35, 49, 67, 109)	-	1 *(8.33%)* (99)
	Apprehension about sending inappropriate form	1 *(0.62%)* (43)	-	-
	Non-punitive	-	1 *(7.69%)* (109)	1 *(8.33%)* (8)
	**Total**	**161** ***(100%)***	**13** ***(100%)***	**12** ***(100%)***
Process and Systems of Reporting	Time	29 *(26.36%)* (9, 10, 17, 22-24, 27, 35, 44, 47, 49, 55, 56, 64, 67, 69, 71, 72, 79, 83, 87, 88, 95, 97, 98, 102, 105)	5 *(6.67%)* (37, 39, 55, 66)	-
	Complexity/simplification of reporting	28 *(25.45%)* (10, 27, 29, 35, 44, 46, 47, 50, 55,	15 *(20.00%)* (9, 11, 27, 44, 52, 55, 66,	1 *(14.29%)* (52)

		62, 64, 66, 69, 71, 72, 81, 83, 85, 88, 93, 95, 101, 105, 108, 109)	69, 91, 107-109)	
	Anonymity and/or confidentiality	22 *(20.00%)* (10, 12, 13, 35, 42, 44, 46, 49, 52, 55, 56, 68, 71, 87, 91, 95, 97, 106, 108)	16 *(21.33%)* (29, 39, 49, 52, 53, 55, 66, 69, 85, 87, 95, 107, 109)	1 *(14.29%)* [18]
	Reporting format	10 *(9.09%)* (29, 45, 69, 72, 85, 102, 105, 109)	21 *(28.00%)* (4, 10, 11, 30, 37, 43, 49, 52, 55, 66, 69, 78, 85, 93, 95, 107, 109)	3 *(42.86%)* (13)
	Type of reporting system	5 *(4.55%)* (24, 27, 106, 109)	11 *(14.67%)* (39, 44, 52, 62, 63, 85, 108, 109)	-
	Unknown destination of report	4 *(3.64%)* (10, 13, 44, 78)	-	-
	Not enough information to complete report	3 *(2.73%)*(10, 33, 98)	1 *(1.33%)* (42)	-
	Sharing/access of reports	3 *(2.73%)* (43, 49, 81)	-	-
	Insufficient routines for reporting	1 *(0.91%)* *(88)*	-	-
	Lack of reporting system	1 *(0.91%)* (20)	-	-
	Administrative task	1 *(0.91%)* (69)	-	1 *(14.29%)* (18)
	Relevant to different HCPs	1 *(0.91%)* (103)	2 *(2.67%)* (43, 66)	-
	Reporting focus	1 *(0.91%)* (71)	2 *(2.67%)* (52)	-
	Information not readily available	1 *(0.91%)* (29)	-	-
	Not specified	-	-	1 *(14.29%)* (18)
	When/where to report	-	1 *(1.33%)* (109)	-
	Doesn't require input from doctors	-	1 *(1.33%)* (66)	-
	**Total**	**110** ***(100%)***	**75** ***(100%)***	**7** ***(100%)***
Incident Characteristics	Level of harm	40 *(43.48%)* (6, 12, 13, 21, 24, 28-30, 35, 46-49, 51, 54, 55, 64, 67, 69, 70, 73, 77-79, 81, 85, 86, 93-95, 97-99, 102, 106-108)	26 *(47.27%)* (6, 23, 29, 30, 32, 39, 43, 45, 47, 48, 50, 51, 55, 74, 91, 94, 98, 102, 106)	-
	Cause of incident	19 *(20.65%)* (6, 7, 9, 10, 28, 40, 44-46, 48, 54, 69, 74, 98, 102)	6 *(10.91%)* (6, 39, 42, 50, 91)	*2 (100%)* (50, 54)
	Frequency of incident	18 *(19.57%)* (6, 7, 23, 28, 29, 42-44, 48, 54, 57, 68, 69, 78, 81, 98)	13 *(23.64%)* (6, 23, 43, 55, 74, 91, 98)	-
	Type of incident	13 *(14.13%)* (9, 10, 23, 24, 35, 40, 62, 63, 67, 69, 72, 102, 109)	8 *(14.55%)* (23, 45, 102)	-

	Level of risk	2 *(2.17%)* [30, 55]	1 *(1.82%)* [30]	-
	Patient characteristics	-	1 *(1.82%)* [45]	-
	**Total**	**92** ***(100%)***	**55** ***(100%)***	**2** ***(100%)***
Individual HCP Characteristics	Value/attitude towards reporting	53 *(59.55%)* [1, 4, 6, 9, 10, 15, 17, 21, 23, 24, 28, 35, 42, 44, 46-49, 52, 64-66, 69, 72, 78, 83, 85, 87, 88, 93, 96, 103, 108, 109]	21 *(51.22%)* [10, 18, 19, 30, 32, 39, 45, 47, 50, 52, 55, 60, 66, 72, 105]	12 *(27.91%)* [16, 25, 54, 77, 83, 97, 99]
	Forgetfulness	9 *(10.11%)* [7, 29, 35, 49, 54, 56, 72, 99, 109]	-	1 *(2.33%)* [54]
	Perception of self	9 *(10.11%)* [10, 12-14, 20, 49, 68]	2 *(4.88%)* [101, 110]	6 *(13.95%)* [13, 110]
	Emotional response	6 *(6.74%)* [10, 12-14, 20, 49, 68]	5 *(12.20%)* [29, 30, 69]	-
	Previous reporting behaviors	5 *(5.62%)* [3, 25, 40, 63, 87]	1 *(2.44%)* [53]	1 *(2.33%)* [54]
	Exposure to errors	2 *(2.25%)* [18, 27]	1 *(2.44%)* [105]	-
	Length of time in employment	2 *(2.25%)* [25]	-	1 *(2.33%)* [25]
	Seniority	1 *(1.12%)*[25]	3 *(7.32%)* [57, 91, 100]	4 *(9.30%)* [25, 40, 50, 54]
	Data required for own purposes	1 *(1.12%)*[44]	-	-
	Work hours	1 *(1.12%)*[40]	1 *(2.44%)* [40]	1 *(2.33%)* [38]
	Demographics	-	2 *(4.88%)* [19, 25]	12 *(27.91%)* [16, 18, 25, 40, 50, 54, 81, 91, 100]
	Profession	-	5 *(12.20%)* [82, 84]	5 *(11.63%)* [82, 84, 110]
	**Total**	**89** ***(100%)***	**41** ***(100%)***	**43** ***(100%)***
Knowledge and Skills	Clarify reporting mechanism	36 *(42.86%)*[7, 10, 12, 13, 23, 27-29, 40, 42, 44, 46-49, 55, 56, 64, 66, 68, 69, 78, 81, 83, 85, 93, 98, 108]	2 *(5.56%)* [69, 85]	5 *(33.33%)* [53, 54, 97, 99]
	Adverse event/near miss clarity	31 *(36.90%)*[17, 23, 24, 29, 32, 45-47, 49, 55, 64, 66, 67, 69, 72, 79, 81, 85, 87, 93, 102, 109]	7 *(19.44%)* [11, 49, 66, 69, 78, 85, 93]	2 *(13.33%)* [97, 99]
	Ability in error recognition	7 *(8.33%)* [17, 24, 43, 46, 74, 83, 95]	4 *(11.11%)* [42, 43, 74, 91]	1 *(6.67%)* [97]
	Training	5 *(5.95%)* [15, 18, 42, 45, 52]	21 *(58.33%)* (37, 42-44, 49, 62, 66, 68, 78, 95, 108, 109)	7 *(46.67%)* [15, 37, 54, 91]

	Awareness	4 *(4.76%)* (46, 79, 95, 98)	2 *(5.56%)* (43, 102)	-
	Not enough information about product being reported	1 *(1.19%)* (101)	-	-
	**Total**	**84** ***(100%)***	**36** ***(100%)***	**15** ***(100%)***
Work Environment	Workload/priority	50 *(62.50%)* (1, 4, 7, 12-14, 22, 24, 28-30, 42, 43, 45-48, 50, 52, 54-56, 63, 65-69, 72, 78, 79, 81, 91, 92, 97, 99-101, 105, 109)	6 *(33.33%)* (29, 42, 43, 80, 91)	3 *(30.00%)* (8, 50, 81)
	Accessibility	27 *(33.75%)* (7, 10, 12, 13, 15, 23, 29, 40, 43-46, 56, 63-65, 68, 72, 81, 87, 95, 98, 109)	11 *(61.11%)* (11, 43, 44, 49, 52, 69, 87, 108, 109)	1 *(10.00%)* (97)
	Not specified	2 *(2.50%)* (4, 64)	-	-
	Unit type	1 *(1.25%)* (100)	1 *(5.56%)* (100)	3 *(30.00%)* (75, 100)
	Physical working conditions	-	-	1 *(10.00%)* (38)
	Satisfaction with work environment	-	-	1 *(10.00%)* (58)
	Care pathways	-	-	1 *(10.00%)* (100)
	**Total**	**80** ***(100%)***	**18** ***(100%)***	**10** ***(100%)***
Organization	Feedback/communication	26 *(34.21%)* (4, 5, 8, 15, 17, 24-26, 30, 35, 46, 49, 55, 65-67, 69, 71, 79, 85, 90, 95, 102, 105, 109)	29 *(29.90%)* (4, 10, 11, 42-44, 49, 52, 55, 61, 62, 66, 69, 75, 78, 85, 91, 93, 107, 109)	2 *(9.09%)* (37, 50)
	Reporting culture	17 *(22.37%)* (6, 8, 9, 15, 24, 46, 63, 66, 78, 88, 98, 100, 104, 105, 109)	16 *(16.49%)* (6, 16, 23, 31, 43, 53, 59, 60, 62, 69, 75, 80, 95)	1 *(4.54%)* (16)
	Learning/improvement	7 *(9.21%)* [20, 59, 76, 90, 94, 102, 103]	13 *(13.40%)* (4, 29, 39, 52, 59, 66, 69, 78, 102, 105)	2 *(9.09%)* (8, 53)
	Use of data	7 *(9.21%)* (4, 17, 24, 79, 90)	2 *(2.06%)* (107, 109)	-
	Policy	6 *(7.89%)* (2, 43, 48, 52, 55, 71)	22 *(22.68%)* (9-11, 30, 36, 39, 42-44, 49, 52, 53, 55, 62, 66, 91, 93, 95)	2 *(9.09%)* (37, 50)
	Management response	5 *(6.58%)* (14, 24, 52, 75, 83)	2 *(2.06%)* (30, 76)	4 *(18.18%)* (18, 53, 76)
	Outcomes of analysis	4 *(5.26%)* (47, 104, 109)	1 *(1.03%)* (69)	-
	Resource	2 *(2.63%)* (14, 52)	3 *(3.09%)* (37, 43, 68)	1 *(4.54%)* (37)
	Ownership	1 *(1.32%)* (39)	4 *(4.12%)* (37, 40, 50)	6 *(27.27%)* (37, 91)

	Hierarchy	1 *(1.32%)* [20]	-	-
	Size	-	3 *(3.09%)* [37, 38, 100]	1 *(4.54%)* [38]
	Nursing quality	-	1 *(1.03%)* [18]	-
	Awareness	-	1 *(1.03%)* [69]	-
	Location	-	-	1 *(4.54%)* [38]
	Elapsed time of IRS integration	-	-	1 *(4.54%)* [37]
	Ward rounds	-	-	1 *(4.54%)* [37]
	**Total**	**76** ***(100%)***	**97** ***(100%)***	**22** ***(100%)***
Team Factors	Relationships	13 *(39.39%)* [6, 14, 30, 36, 47, 49, 55, 56, 69, 87, 105]	2 *(10.00%)* [45, 100]	-
	Influence of Seniors	7 *(21.21%)* [25, 45, 51, 59, 87, 95]	1 *(5.00%)* [49]	-
	Peer reporting	5 *(15.15%)* [28, 83, 102]	3 *(15.00%)* [18, 19, 44]	-
	Teamwork/communication	3 *(9.09%)* [20, 43, 55]	7 *(35.00%)* [31, 43, 80, 91]	2 *(66.67%)* [8]
	Support/encouragement	3 *(9.09%)* [35, 49, 69]	1 *(5.00%)* [49]	1 *(33.33%)* [99]
	Medical doctor involvement	1 *(3.03%)* [85]	1 *(5.00%)* [85]	-
	Error committed by junior staff	1 *(3.03%)* [30]	1 *(5.00%)* [51]	-
	Team culture	-	4 *(20.00%)* [10, 19, 60, 80]	-
	**Total**	**33** *(100%)*	20 *(100%)*	**3** *(100%)*
Professional Ethics	Concealment	5 *(21.74%)* [1, 49, 102]	1 *(5.88%)* [55]	-
	Duty	1 *(4.35%)* [9]	8 *(47.06%)* [10, 32, 43, 44, 47, 102]	1 *(25.00%)* [50]
	Accountability	-	2 *(11.76%)* [23, 47]	-
	Responsibility	15 *(65.22%)* [2, 23, 33, 35, 40, 46, 48, 63, 66, 69, 72, 78, 85, 88]	5 *(29.41%)* [33, 34, 91, 105]	1 *(25.00%)* [38]
	Culture	2 *(8.70%)* [49, 87]	-	-
	Legal	-	1 *(5.88%)* [25]	2 *(50.00%)* [25]
	**Total**	**23** *(100%)*	**17** *(100%)*	**4** *(100%)*

References

1. Albolino S, Tartaglia R, Bellandi T, Amicosante AM, Bianchini E, Biggeri A. Patient safety and incident reporting: survey of Italian healthcare workers. Quality & safety in health care. 2010;19 Suppl 3:i8-12.
2. Alsafi E, Bahroon SA, Tamim H, Al-Jahdali HH, Alzahrani S, Al Sayyari A. Physicians' attitudes toward reporting medical errors-an observational study at a general hospital in Saudi Arabia. Journal of patient safety. 2011;7(3):144-7.
3. Anderson JE, Kodate N, Walters R, Dodds A. Can incident reporting improve safety? Healthcare practitioners' views of the effectiveness of incident reporting. International Journal for Quality in Health Care. 2013;25(2):141-50.
4. Arfanis K, Smith A. Informal risk assessment strategies in health care staff: An unrecognized source of resilience? Journal of Evaluation in Clinical Practice. 2012;18(6):1140-6.
5. Armitage G, Newell R, Wright J. Improving the quality of drug error reporting. Journal of Evaluation in Clinical Practice. 2010;16(6):1189-97.
6. Ashcroft DM, Morecroft C, Parker D, Noyce PR. Likelihood of reporting adverse events in community pharmacy: An experimental study. Quality and Safety in Health Care. 2006;15(1):48-52.
7. Bäckström M, Mjörndal T, Dahlqvist R, Nordkvist-Olsson T. Attitudes to reporting adverse drug reactions in northern Sweden. European Journal of Clinical Pharmacology. 2000;56(9-10):729-32.
8. Ballangrud R, Hedelin B, Hall-Lord ML. Nurses' perceptions of patient safety climate in intensive care units: A cross-sectional study. Intensive and Critical Care Nursing. 2012;28(6):344-54.
9. Bateman DN, Sanders GL, Rawlins MD. Attitudes to adverse drug reaction reporting in the Northern Region. British Journal of Clinical Pharmacology. 1992;34(5):421-6.
10. Bawazir SA. ATTITUDE OF COMMUNITY PHARMACISTS IN SAUDI ARABIA TOWARDS ADVERSE DRUG REACTION REPORTING. Saudi Pharmaceutical Journal. 2006;14(1):75-83.
11. Beasley JW, Escoto KH, Karsh BT. Design Elements for a Primary Care Medical Reporting System. Wisconsin Medical Journal. 2004;103(1):56-9.
12. Belton KJ, Lewis SC, Payne S, Rawlins MD, Wood SM. Attitudinal survey of adverse drug reaction reporting by medical practitioners in the United Kingdom [see comments]. British Journal of Clinical Pharmacology. 1995;39(3):223-6.
13. Belton KJ. Attitude survey of adverse drug-reaction reporting by health care professionals across the European Union. European Journal of Clinical Pharmacology. 1997;52(6):423-7.
14. Blegen MA, Vaughn T, Pepper G, Vojir C, Stratton K, Boyd M, et al. Patient and Staff Safety: Voluntary Reporting. American Journal of Medical Quality. 2004;19(2):67-74.
15. Braithwaite J, Westbrook MT, Travaglia JF, Hughes C. Cultural and associated enablers of, and barriers to, adverse incident reporting. Quality & safety in health care. 2010;19(3):229-33.

16. Chang IC, Hsu HM. Predicting medical staff intention to use an online reporting system with modified unified theory of acceptance and use of technology. Telemedicine journal and e-health : the official journal of the American Telemedicine Association. 2012;18(1):67-73.
17. Chiang HY, Pepper GA. Barriers to nurses' reporting of medication administration errors in Taiwan. J Nurs Scholarsh. 2006;38(4):392-9.
18. Chiang HY, Lin SY, Hsu SC, Ma SC. Factors determining hospital nurses' failures in reporting medication errors in Taiwan. Nursing Outlook. 2010;58(1):17-25.
19. Chiang HY, Lin SY, Hsiao YC, Chang Y. Culture influence and predictors for behavioral involvement in patient safety among hospital nurses in Taiwan. Journal of Nursing Care Quality. 2012;27(4):359-67.
20. Church JA, Adams RD, Hendrix LH, Holmes JA, Marks LB, Chen RC. National study to determine the comfort levels of radiation therapists and medical dosimetrists to report errors. Practical Radiation Oncology. 2013;3(4):e165-e70.
21. Clark M, Gray M, Mooney J. New graduate occupational therapists' perceptions of near-misses and mistakes in the workplace. International Journal of Health Care Quality Assurance. 2013;26(6):564-76.
22. Coley KC, Pringle JL, Weber RJ, Rice K, Ramanujam R, Sirio CA. Perceived Barriers in Using a Region-Wide Medication Error Reporting System. Journal of Patient Safety. 2006;2(1):39-44.
23. Cosentino M, Leoni O, Banfi F, Lecchini S, Frigo G. ATTITUDES TO ADVERSE DRUG REACTION REPORTING BY MEDICAL PRACTITIONERS IN A NORTHERN ITALIAN DISTRICT. Pharmacological Research. 1997;35(2):85-8.
24. Covell CL, Ritchie JA. Nurses responses to medication errors: Suggestions for the development of organizational strategies to improve reporting. Journal of Nursing Care Quality. 2009;24(4):287-97.
25. Daly JM, Jogerst GJ. Association of knowledge of adult protective services legislation with rates of reporting of abuse in Iowa nursing homes. J Am Med Dir Assoc. 2005;6(2):113-20.
26. Davies EC, Chandler CIR, Innocent SHS, Kalumuna C, Terlouw DJ, Lalloo DG, et al. Designing adverse event forms for real-world reporting: Participatory research in uganda. PLoS ONE. 2012;7(3).
27. Ehrenpreis ED, Sifuentes H, Ehrenpreis JE, Smith ZL, Marshall ML. Suboptimal reporting of adverse medical events to the FDA Adverse Events Reporting System by nurse practitioners and physician assistants. Expert Opinion on Drug Safety. 2012;11(2):177-83.
28. Eland, Belton, Van G, Meiners, Rawlins, Stricker. Attitudinal survey of voluntary reporting of adverse drug reactions. British Journal of Clinical Pharmacology. 1999;48(4):623-7.
29. Elder NC, Graham D, Brandt E, Hickner J. Barriers and motivators for making error reports from family medicine offices: A report from the American Academy of Family Physicians National Research Network (AAFP NRN) (Journal of the American Board of Family Medicine (2007) 20, 2, (115-123) DOI: 10.3122/jabfm.2007.04.070111). Journal of the American Board of Family Medicine. 2007;20(4):425.

30. Elder NCB, S.M.; Nagy, M.; Kudel, I.; Render, M.L. Nurses' perceptions of error communication and reporting in the intensive care unit. J Patient Saf. 2008;4:162-8.
31. Erler C, Edwards NE, Ritchey S, Pesut DJ, Sands L, Wu J. Perceived Patient Safety Culture in a Critical Care Transport Program. Air Medical Journal. 2013;32(4):208-15.
32. Espin S, Wickson-Griffiths A, Wilson M, Lingard L. To report or not to report: A descriptive study exploring ICU nurses' perceptions of error and error reporting. Intensive and Critical Care Nursing. 2010;26(1):1-9.
33. Espin S, Regehr G, Levinson W, Baker GR, Biancucci C, Lingard L. Factors Influencing Perioperative Nurses' Error Reporting Preferences. AORN Journal. 2007;85(3):527–8, 30–32, 34–36, 39–43.
34. Espin S, Levinson W, Regehr G, Baker GR, Lingard L. Error or "act of God"? A study of patients' and operating room team members' perceptions of error definition, reporting, and disclosure. Surgery. 2006;139(1):6-14.
35. Evans SM, Berry JG, Smith BJ, Esterman A, Selim P, O'Shaughnessy J, et al. Attitudes and barriers to incident reporting: A collaborative hospital study. Quality and Safety in Health Care. 2006;15(1):39-43.
36. Fairbanks RJ, Crittenden CN, O'Gara KG, Wilson MA, Pennington EC, Chin NP, et al. Emergency medical services provider perceptions of the nature of adverse events and near-misses in out-of-hospital care: An ethnographic view. Academic Emergency Medicine. 2008;15(7):633-40.
37. Fukuda H, Imanaka Y, Hirose M, Hayashida K. Impact of system-level activities and reporting design on the number of incident reports for patient safety. Quality & safety in health care. 2010;19(2):122-7.
38. Gaal S, van den Hombergh P, Verstappen W, Wensing M. Patient safety features are more present in larger primary care practices. Health Policy. 2010;97(1):87-91.
39. Garbutt J, Brownstein DR, Klein EJ, Waterman A, Krauss MJ, Marcuse EK, et al. Reporting and disclosing medical errors: Pediatricians' attitudes and behaviors. Archives of Pediatrics and Adolescent Medicine. 2007;161(2):179-85.
40. Generali JA, Danish MA, Rosenbaum SE. Knowledge of and attitudes about adverse drug reaction reporting among Rhode Island pharmacists. The Annals of pharmacotherapy. 1995;29(4):365-9.
41. Gladstone J. Drug administration errors: a study into the factors underlying the occurrence and reporting of drug errors in a district general hospital. Journal of advanced nursing. 1995;22(4):628-37.
42. Green CF, Mottram DR, Raval D, Proudlove C, Randall C. Community pharmacists' attitudes to adverse drug reaction reporting. International Journal of Pharmacy Practice. 1999;7(2):92-9.
43. Green CF, Mottram DR, Rowe PH, Pirmohamed M. Attitudes and knowledge of hospital pharmacists to adverse drug reaction reporting. British Journal of Clinical Pharmacology. 2001;51(1):81-6.

44. Van Grootheest AC, Mes K, De Jong-Van Den Berg LTW. Attitudes of community pharmacists in the Netherlands towards adverse drug reaction reporting. International Journal of Pharmacy Practice. 2002;10(4):267-72.
45. Haines TP, Cornwell P, Fleming J, Varghese P, Gray L. Documentation of in-hospital falls on incident reports: qualitative investigation of an imperfect process. BMC health services research. 2008;8:254.
46. Handler SM, Perera S, Olshansky EF, Studenski SA, Nace DA, Fridsma DB, et al. Identifying Modifiable Barriers to Medication Error Reporting in the Nursing Home Setting. Journal of the American Medical Directors Association. 2007;8(9):568-74.
47. Hartnell N, MacKinnon N, Sketris I, Fleming M. Identifying, understanding and overcoming barriers to medication error reporting in hospitals: A focus group study. BMJ Quality and Safety. 2012;21(5):361-8.
48. Hasford J, Goettler M, Munter KH, Müller-Oerlinghausen B. Physicians' knowledge and attitudes regarding the spontaneous reporting system for adverse drug reactions. Journal of Clinical Epidemiology. 2002;55(9):945-50.
49. Heard GC, Sanderson PM, Thomas RD. Barriers to adverse event and error reporting in anesthesia. Anesthesia and Analgesia. 2012;114(3):604-14.
50. Herdeiro M, Figueiras A, Polónia J, Gestal-Otero JJ. Influence of Pharmacists’ Attitudes on Adverse Drug Reaction Reporting. Drug Safety. 2006;29(4):331-40.
51. Hohenhaus SM. Emergency Nursing and Medical Error-A Survey of Two States. Journal of Emergency Nursing. 2008;34(1):20-5.
52. Holmstrom AR, Airaksinen M, Weiss M, Wuliji T, Chan XH, Laaksonen R. National and local medication error reporting systems: a survey of practices in 16 countries. Journal of patient safety. 2012;8(4):165-76.
53. Hutchinson A, Young TA, Cooper KL, McIntosh A, Karnon JD, Scobie S, et al. Trends in healthcare incident reporting and relationship to safety and quality data in acute hospitals: Results from the National Reporting and Learning System. Quality and Safety in Health Care. 2009;18(1):5-10.
54. Irujo M, Beitia G, Bes-Rastrollo M, Figueiras A, Hernández-Diaz S, Lasheras B. Factors that Influence Under-Reporting of Suspected Adverse Drug Reactions among Community Pharmacists in a Spanish Region. Drug Safety. 2007;30(11):1073-82.
55. Jeffe DB, Dunagan WC, Garbutt J, Burroughs TE, Gallagher TH, Hill PR, et al. Using focus groups to understand physicians' and nurses' perspectives on error reporting in hospitals. Joint Commission journal on quality and safety. 2004;30(9):471-9.
56. Jennings PA, Stella J. Barriers to incident notification in a regional prehospital setting. Emergency Medicine Journal. 2011;28(6):526-9.
57. Johnstone MJ, Kanitsaki O. Patient safety and the integration of graduate nurses into effective organizational clinical risk management systems and processes: an Australian study. Quality management in health care. 2008;17(2):162-73.

58. Joolaee S, Hajibabaee F, Peyrovi H, Haghani H, Bahrani N. The relationship between incidence and report of medication errors and working conditions. International Nursing Review. 2011;58(1):37-44.
59. Kagan I, Barnoy S. Factors associated with reporting of medication errors by Israeli nurses. Journal of Nursing Care Quality. 2008;23(4):353-61.
60. Kagan I, Barnoy S. Organizational safety culture and medical error reporting by israeli nurses. Journal of Nursing Scholarship. 2013;45(3):273-80.
61. Kaldjian LC, Jones EW, Wu BJ, Forman-Hoffman VL, Levi BH, Rosenthal GE. Reporting medical errors to improve patient safety: A survey of physicians in teaching hospitals. Archives of Internal Medicine. 2008;168(1):40-6.
62. Karsh BT, Escoto KH, Beasley JW, Holden RJ. Toward a theoretical approach to medical error reporting system research and design. Applied Ergonomics. 2006;37(3):283-95.
63. Kennedy AG, Littenberg B. Medication error reporting by community pharmacists in Vermont. Journal of the American Pharmacists Association : JAPhA. 2004;44(4):434-8.
64. Khan TM. Community pharmacists' knowledge and perceptions about adverse drug reactions and barriers towards their reporting in eastern region, alahsa, Saudi Arabia. Therapeutic Advances in Drug Safety. 2013;4(2):45-51.
65. King ES, Moyer DV, Couturie MJ, Gaughan JP, Shulkin DJ. Getting doctors to report medical errors: project DISCLOSE. Jt Comm J Qual Patient Saf. 2006;32(7):382-92.
66. Kingston MJ, Evans SM, Smith BJ, Berry JG. Attitudes of doctors and nurses towards incident reporting: A qualitative analysis. Medical Journal of Australia. 2004;181(1):36-9.
67. Kreckler S, Catchpole K, McCulloch P, Handa A. Factors influencing incident reporting in surgical care. Quality and Safety in Health Care. 2009;18(2):116-20.
68. Li Q, Zhang S-m, Chen H-t, Fang S-p, Yu X, Liu D, et al. Awareness and attitudes of healthcare professionals in Wuhan, China to the reporting of adverse drug reactions. Chinese medical journal. 2004;117(6):856-61.
69. Martowirono K, Jansma JD, Van Luijk SJ, Wagner C, Bijnen AB. Possible solutions for barriers in incident reporting by residents. Journal of Evaluation in Clinical Practice. 2012;18(1):76-81.
70. Mayo AM, Duncan D. Nurse Perceptions of Medication Errors: What We Need to Know for Patient Safety. Journal of Nursing Care Quality. 2004;19(3):209-17.
71. McArdle D, Burns N, Ireland A. Attitudes and beliefs of doctors towards medication error reporting. International Journal of Health Care Quality Assurance. 2003;16(7):326-33.
72. Merchant RN, Gully PM. A survey of British Columbia anesthesiologists on a provincial critical incident reporting program. Canadian Journal of Anesthesia. 2005;52(7):680-4.
73. Mrayyan MT, Shishani K, Al-Faouri I. Rate, causes and reporting of medication errors in Jordan: nurses’ perspectives. Journal of Nursing Management. 2007;15(6):659-70.
74. Mustafa G, Saeed ur R, Aziz MT. Adverse drug reaction reporting system at different hospitals of Lahore, Pakistan - An evaluation and patient outcome analysis. Journal of Applied Pharmacy. 2013;5(1):713-9.

75. Naveh E, Katz-Navon T, Stern Z. Readiness to report medical treatment errors: the effects of safety procedures, safety information, and priority of safety. Medical care. 2006;44(2):117-23.
76. Okuyama A, Sasaki M, Kanda K. The relationship between incident reporting by nurses and safety management in hospitals. Quality management in health care. 2010;19(2):164-72.
77. Osborne J, Blais K, Hayes JS. Nurses' Perceptions: When Is It a Medication Error? Journal of Nursing Administration. 1999;29(4):33-8.
78. Parvizi N, Robertson I, McWilliams RG. Medical device adverse incident reporting in interventional radiology. Clinical Radiology. 2014;69(3):263-7.
79. Patrician PA, Brosch LR. Medication error reporting and the work environment in a military setting. Journal of Nursing Care Quality. 2009;24(4):277-86.
80. Rasmussen K, Pedersen AHM, Pape L, Lyngby Mikkelsen K, Madsen MD, Jacob Nielsen K. Work environment influences adverse events in an emergency department. Danish Medical Journal. 2014;61(5).
81. Rogers A, Israel E, Smith CR, et al. Physician knowledge, attitudes, and behavior related to reporting adverse drug events. Archives of Internal Medicine. 1988;148(7):1596-600.
82. Rowin EJ, Lucier D, Pauker SG, Kumar S, Chen J, Salem DN. Does error and adverse event reporting by physicians and nurses differ? Joint Commission journal on quality and patient safety / Joint Commission Resources. 2008;34(9):537-45.
83. Sanghera IS, Franklin BD, Dhillon S. The attitudes and beliefs of healthcare professionals on the causes and reporting of medication errors in a UK Intensive care unit. Anaesthesia. 2007;62(1):53-61.
84. Sarvadikar A, Prescott G, Williams D. Attitudes to reporting medication error among differing healthcare professionals. European Journal of Clinical Pharmacology. 2010;66(8):843-53.
85. Schectman JM, Plews-Ogan ML. Physician perception of hospital safety and barriers to incident reporting. Joint Commission journal on quality and patient safety / Joint Commission Resources. 2006;32(6):337-43.
86. Schulmeister L. Chemotherapy medication errors: descriptions, severity, and contributing factors. Oncology nursing forum. 1999;26(6):1033-42.
87. Sharma A, Jain P, Parmar B, Muzaffar J, Monson JRT. Incident Reporting in Surgical Trainees-Revisited. Journal of Patient Safety. 2008;4(3):191-4.
88. Soderberg J, Grankvist K, Brulin C, Wallin O. Incident reporting practices in the preanalytical phase: Low reported frequencies in the primary health care setting. Scandinavian Journal of Clinical & Laboratory Investigation. 2009;69(7):731-5.
89. Soleimani F. Learning from mistakes in New Zealand hospitals: what else do we need besides "no-fault"? New Zealand Medical Journal. 2006;119(1239):U2099.
90. Stratton KM, Blegen MA, Pepper G, Vaughn T. Reporting of Medication Errors by Pediatric Nurses. Journal of Pediatric Nursing. 2004;19(6):385-92.
91. Sweis D, Wong IK. A Survey on Factors that Could Affect Adverse Drug Reaction Reporting According to Hospital Pharmacists in Great Britain. Drug Safety. 2000;23(2):165-72.

92. Tariq A, Georgiou A, Westbrook J. Medication incident reporting in residential aged care facilities: limitations and risks to residents' safety. BMC geriatrics. 2012;12:67.
93. Taylor JA, Brownstein D, Christakis DA, Blackburn S, Strandjord TP, Klein EJ, et al. Use of incident reports by physicians and nurses to document medical errors in pediatric patients. Pediatrics. 2004;114(3):729-35.
94. Throckmorton T, Etchegaray J. Factors Affecting Incident Reporting by Registered Nurses: The Relationship of Perceptions of the Environment for Reporting Errors, Knowledge of the Nursing Practice Act, and Demographics on Intent to Report Errors. Journal of Perianesthesia Nursing. 2007;22(6):400-12.
95. Tobaiqy M, Stewart D. Exploring health professionals' experiences of medication errors in Saudi Arabia. International Journal of Clinical Pharmacy. 2013;35(4):542-5.
96. Turner S, Ramsay A, Fulop N. The role of professional communities in governing patient safety. Journal of Health, Organisation and Management. 2013;27(4):527-43.
97. Uribe CL, Schweikhart SB, Pathak DS, Marsh GB, Fraley RR. Perceived barriers to medical-error reporting: An exploratory investigation. Journal of Healthcare Management. 2002;47(4):263-80.
98. Vessal G, Mardani Z, Mollai M. Knowledge, attitudes, and perceptions of pharmacists to adverse drug reaction reporting in Iran. Pharmacy World and Science. 2009;31(2):183-7.
99. Vincent C, Stanhope N, Crowley-Murphy M. Reasons for not reporting adverse incidents: An empirical study. Journal of Evaluation in Clinical Practice. 1999;5(1):13-21.
100. Vogus TJ, Sutcliffe KM. The impact of safety organizing, trusted leadership, and care pathways on reported medication errors in hospital nursing units. Medical Care. 2007;45(10):997-1002.
101. Walji R, Boon H, Barnes J, Welsh S, Austin Z, Baker GR. Reporting natural health product related adverse drug reactions: Is it the pharmacist's responsibility? International Journal of Pharmacy Practice. 2011;19(6):383-91.
102. Walker SB, Lowe MJ. Nurses' views on reporting medication incidents. International journal of nursing practice. 1998;4(2):97-102.
103. Waring JJ. A qualitative study of the intra-hospital variations in incident reporting. International Journal for Quality in Health Care. 2004;16(5):347-52.
104. Waring JJ. Beyond blame: Cultural barriers to medical incident reporting. Social Science and Medicine. 2005;60(9):1927-35.
105. Waters NF, Hall WA, Brown H, Espezel H, Palmer L. Perceptions of Canadian labour and delivery nurses about incident reporting: A qualitative descriptive focus group study. International Journal of Nursing Studies. 2012;49(7):811-21.
106. Weissman JS, Annas CL, Epstein AM, Schneider EC, Clarridge B, Kirle L, et al. Error reporting and disclosure systems: views from hospital leaders. JAMA : the journal of the American Medical Association. 2005;293(11):1359-66.

107. Williams SD, Phipps DL, Ashcroft DM. Understanding the attitudes of hospital pharmacists to reporting medication incidents: A qualitative study. Research in Social and Administrative Pharmacy. 2013;9(1):80-9.
108. Winchester SA, Tomkins S, Cliffe S, Batty L, Ncube F, Zuckerman M. Healthcare workers' perceptions of occupational exposure to blood-borne viruses and reporting barriers: a questionnaire-based study. J Hosp Infect. 2012;82(1):36-9.
109. Yong H, Kluger MT. Incident reporting in anaesthesia: A survey of practice in New Zealand. Anaesthesia and Intensive Care. 2003;31(5):555-9.
110. Zwart DLM, Heddema WS, Vermeulen MI, Van Rensen ELJ, Verheij TJM, Kalkman CJ. Lessons learnt from incidents reported by postgraduate trainees in Dutch general practice. A prospective cohort study. BMJ Quality and Safety. 2011;20(10):857-62.
