# Supplementary material for: Development of a theoretical framework of factors affecting patient safety incident reporting: a theoretical review of the literature
Source: BMJ Open. 2017 Dec 27;7(12):e017155. doi: 10.1136/bmjopen-2017-017155 (PMC5770969; doi:10.1136/bmjopen-2017-017155)
Supplement: Author's manuscript [file bmjopen-2017-017155.draft_revisions.pdf]

BMJ Open is committed to open peer review. As part of this commitment we make the peer review history of every article we publish publicly available.

When an article is published we post the peer reviewers' comments and the authors' responses online. We also post the versions of the paper that were used during peer review. These are the versions that the peer review comments apply to.

The versions of the paper that follow are the versions that were submitted during the peer review process. They are not the versions of record or the final published versions. They should not be cited or distributed as the published version of this manuscript.

BMJ Open is an open access journal and the full, final, typeset and author-corrected version of record of the manuscript is available on our site with no access controls, subscription charges or pay-per-view fees (http://bmjopen.bmj.com).

If you have any questions on BMJ Open's open peer review process please email editorial.bmjopen@bmj.com

BMJ Open

Development of a Theoretical Framework of Factors Affecting Patient Safety Incident Reporting: A Theoretical Review of the Literature

Journal:	*BMJ Open*
Manuscript ID	bmjopen-2017-017155
Article Type:	Research
Date Submitted by the Author:	04-Apr-2017
Complete List of Authors:	Archer, Stephanie; Imperial College London, NIHR Imperial Patient Safety Translational Research Centre; Imperial College London,<br>Hull, Louise; King's College London<br>Soukup, Tayana; Imperial College London, NIHR Imperial Patient Safety Translational Research Centre<br>Mayer, Erik; Imperial College London, Dept. of Biosurgery and Surgical Technology<br>Athanasiou, Thanos; Imperial College London, Surgery and Cancer<br>Sevdalis, Nick; King's College London,<br>Darzi, Ara; Imperial College London, Institute of Global Health Innovation
**Primary Subject Heading**:	Health services research
Secondary Subject Heading:	Health policy
Keywords:	Incident reporting, Patient Safety, Service Quality

SCHOLARONE™
Manuscripts

Development of a Theoretical Framework of Factors Affecting Patient Safety Incident Reporting: A Theoretical Review of the Literature

Stephanie Archer, PhD*[1] Email: stephanie.archer@imperial.ac.uk

Louise Hull, PhD*[1,2] Email: louise.hull@kcl.ac.uk

Tayana Soukup, MSc[1] Email: t.soukup@imperial.ac.uk

Erik Mayer, PhD[1] Email: e.mayer@imperial.ac.uk

Thanos Athanasiou, PhD[1] Email: t.athanasiou@imperial.ac.uk

Nick Sevdalis, PhD[1,2] Email: nick.sevdalis@kcl.ac.uk

Ara Darzi, MD[1] Email: a.darzi@imperial.ac.uk

*SA & LH co-first author

Corresponding author: Dr Stephanie Archer, Research Fellow, NIHR Imperial Patient Safety Translational Research Centre, Imperial College London, Medical School Building, Room 5.03, St Mary's Campus, Norfolk Place, London, W2 1PG. Email: Stephanie.archer@imperial.ac.uk Telephone: 020 7594 3192 Fax: 02075943137

[1] Patient Safety Translational Research Centre, Imperial College London, London, UK

[2] Centre for Implementation Science, King's College London, London, UK.

Key Words: Incident reporting, Patient Safety, Service Quality

For peer review only - http://bmjopen.bmj.com/site/about/guidelines.xhtml

Abstract

Objectives: The development and implementation of incident reporting systems within healthcare continues to be a fundamental strategy to reduce preventable patient harm and improve the quality and safety of healthcare. We sought to identify factors contributing to patient safety incident reporting.

Design: To facilitate improvements in incident reporting, a theoretical framework, encompassing factors that act as barriers and enablers of reporting, was developed. Embase, Ovid MEDLINE(R) and PsycINFO were searched to identify relevant articles published between January 1980 and May 2014. A comprehensive search strategy including MeSH terms and keywords was developed to identify relevant articles. Data were extracted by three independent researchers; to ensure the accuracy of data extraction, all studies eligible for inclusion were rescreened by two reviewers.

Results: The literature search identified 3,049 potentially eligible articles; of these, 110 articles, including over 29,726 participants, met the inclusion criteria. In total, 748 barriers were identified (frequency count) across the 110 articles. In comparison, 372 facilitators to incident reporting and 118 negative cases were identified. The top two barriers cited were fear of adverse consequences (161, representing 21.52% of barriers) and process and systems of reporting (110, representing 14.71% of barriers). In comparison, the top two facilitators were organisational (97, representing 26.08% of facilitators) and process and systems of reporting (75, representing 20.16% of facilitators).

Conclusion: A wide range of factors contributing to engagement in incident reporting exist. Efforts that address the current tendency to under-report must consider the full

For peer review only - http://bmjopen.bmj.com/site/about/guidelines.xhtml

range of factors in order to develop interventions as well as a strategic policy approach for improvement.

Article Summary – strengths and limitations

- The synthesis included quantitative, qualitative and mixed methods research and have not restricted the literature to specific incident reporting systems.
- Only articles published in English were included.
- The last systematic search for literature was conducted on 29/05/2014, meaning that literature published since this date will not have been included.
- Studies detailing interventions to improve incident reporting and studies detailing variations in engagement in incident reporting were not included.
- Large heterogeneity across studies in terms of outcome measures and methodologies meant conduction of meta-analysis was precluded.

For peer review only

For peer review only - http://bmjopen.bmj.com/site/about/guidelines.xhtml

Background

The development and implementation of incident reporting systems within healthcare continues to be a fundamental strategy to reduce preventable patient harm and improve the quality and safety of healthcare on a local, regional and national basis.[1, 2] Although coverage and sophistication vary widely, incident reporting systems have now been in place for more than a decade in a number of countries.[3]

A key factor that compromises the ability of incident reporting systems to improve patient safety is underreporting. In the United States it is estimated that 50-96% of incidents are not reported.[2, 4, 5] Failure to report patient safety incidents significantly hinders the underlying goals of incident reporting systems; low levels of reporting makes it is difficult at best to identify and prioritise patient safety risks, and hampers learning from such incidents and ultimately improvements in patient safety. Whilst debate continues to exist regarding whether all patient safety incidents should be reported,[6, 7], it is extremely important to understand the factors that act as barriers and facilitators to incident reporting so that 'sufficient' levels of reporting exist to facilitate learning and improvement.

A number of studies exploring barriers and facilitators to incident reporting have been conducted.[8-11] In addition, a number of literature reviews to identify barriers and facilitators to incident reporting have been published.[12-14] Although previous work has made a valuable contribution to our understanding of factors affecting incident reporting, previous work has been limited in scope (e.g. focusing on the psychological factors affecting incident reporting[14]; focusing on perceived barriers influencing incident reporting by nurses;[13] factors affecting reporting of incidents

For peer review only - http://bmjopen.bmj.com/site/about/guidelines.xhtml

related to medical devices and other healthcare technologies).[12] As such, to date, there has been no definitive synthesis and evaluation of the factors that prevent or promote reporting.

The primary aim of this theoretical review was to systematically identify the factors affecting patient safety incident reporting. The secondary aims were, firstly, to develop theoretical framework, of factors acting as barriers and facilitators to incident reporting to guide implementation of interventions to increase engagement, and, secondly, to determine the prevalence of factors to guide the development of interventions and policies to improve incident reporting.

review only

For peer review only - http://bmjopen.bmj.com/site/about/guidelines.xhtml

Methods

Theoretical Review

A theoretical review was conducted as the overarching goal of the review was to build explanation of factors affecting incident reporting. In line with a theoretical review both quantitative and qualitative data were eligible for inclusion and interpretive methods were used to synthesize findings.

Study searches and selection

A systematic search strategy was developed and an electronic search was carried out in three databases**:** Embase, Ovid MEDLINE(R) and PsycINFO. The last search was conducted on 29/05/2014; whilst the last search was conducted 2 years ago, this reflects the sheer volume of articles that were included in this review. Search terms included those related to patient safety incidents, incident reporting systems, and barriers and facilitators to engagement in reporting (see table 1 for full search terms). Time and language of publications was restricted from 1980 and English language.

TABLE 1 HERE

Eligibility criteria

Inclusion Criteria

1. Studies reporting factors influencing the likelihood of incident report engagement in any healthcare setting (e.g. primary and secondary healthcare) and employing any study designs (e.g. qualitative, quantitative, mixed-methods)

For peer review only

Exclusion Criteria

1. Studies reporting aspects of incident reporting systems and/or incident reporting perceived positively and/or negatively by healthcare professionals without data relating perceptions to incident reporting engagement
2. Studies reporting data relating to disclosure of patient safety incidents to patients or their families (a systematic review of the literature on patient/family disclosure has previously been published)[15]
3. Studies reporting data relating to the effectiveness of interventions to improve incident reporting (a systematic review of the literature on the effectiveness of interventions to increase clinical incident reporting in health care has previously been published.[13]
4. Studies reporting statistical models where the impact of individual barriers and facilitators to engagement in incident reporting was unable to be determined.

The eligibility criteria was developed to maintain a focus on factors having a direct impact upon incident reporting engagement rather than simply identifying and listing factors of incident reporting which were perceived positively or negatively by healthcare professionals. Identifying elements of incident reporting perceived positively or negatively by healthcare professionals does not equate to identify factors that have an impact on reporting behaviour. In such studies, it is not possible to determine the impact on reporting behaviour - the primary focus of this review.

Data extraction

For peer review only - http://bmjopen.bmj.com/site/about/guidelines.xhtml

After the removal of duplicates, two authors (SA and LH) independently reviewed all articles on the basis of the titles and abstract. Three authors (SA, LH and TS) reviewed the articles at full-text stage.Data was extracted using an extraction template. The following data was extracted: first author's name, year of publication, country, study design, study population, sample size, and factors that decrease (barriers), increase (facilitators) or were neither a barrier nor facilitator to engagement in incident reporting (negative cases). To ensure the accuracy of data extraction, all studies eligible for inclusion were rescreened by two reviewers (SA and LH).

Quality Assessment

Many assessment tools and checklists have been developed to appraise the quality and susceptibility to bias of studies (e.g. The Cochrane Collaboration's tool for assessing risk of bias in randomized trials;[16] AMSTAR tool to assess the methodological quality of systematic reviews;[17] tools to assess the quality of qualitative research studies).[18] The decision not to assess the quality of studies was made for a number of reasons. First, the large heterogeneity of study designs would have made comparisons between study designs difficult at best. Second, quality appraisal is not considered necessary for theoretical reviews.[19] Third, it has been argued that it is important, but difficult, to distinguish between 'quality of reporting' and the 'quality of a study'.[20] As such, articles were not excluded from the current review based on 'quality' nor was weight assigned to studies based on quality.

Data analysis and initial theoretical framework development

For peer review only - http://bmjopen.bmj.com/site/about/guidelines.xhtml

A grounded theory approach was used to guide the development of the theoretical framework. Grounded theory is associated with the discovery of theory from data systematically obtained from social research.[21] It has been identified as a method where thorough and theoretically relevant analysis of a topic can be reached, specifically within literature reviews.[22] In light of this, a three-stage approach was undertaken to develop a theory of factors contributing to engagement in patient safety incident reporting. The first stage, *coding*, includes identifying parts of the data that relate the phenomena in question (in this case, incident reporting). During this stage, known as *open coding* in the grounded theory literature, three authors (SA, LH & TS) read and re-read each paper and identified sections of the paper that were relevant to the research question. Initial concepts developed from these were noted down at this stage; in some cases these were consistent with pre-existing literature (e.g. in the case of a standardised scale), but in others allowed for unseen insights to develop across the data corpus (e.g. in qualitative studies). In the second stage, *conceptualising*, or *axial coding*, focused on grouping together the initial codes where there were relationships to form higher order categories. These were given names. Stage three, *categorising*, or *selective coding* focused on linking together similar higher order categories that contained similar concepts which could underpin the reasoning behind the way that the phenomena (in this case, incident reporting) could be explained. Figure 1 displays an example of how these stages were applied.

FIGURE 1 HERE

Engagement in these three stages allowed constant comparison between the articles in the dataset to be performed until a theoretical framework was confirmed.

For peer review only - http://bmjopen.bmj.com/site/about/guidelines.xhtml

The final theoretical framework was reviewed by another member of the research team (NS) and feedback regarding the category descriptors was incorporated. The final theoretical framework of factors contributing to patient safety incident reporting engagement is displayed in Table 2.

TABLE 2 HERE

The theoretical framework developed was used to organise the identification of factors found to affect incident reporting and to quantify their prevalence. This approach is consistent with existing frameworks in the patient safety literature, for example Lawton et al employed a similar approach to quantify the prevalence of factors contributing to patient safety incidents in hospital settings.[23]

Patient and public involvement

No patients were involved in setting the research question or the outcome measures, nor were they involved in the design and implementation of the study. We do not

Findings

The search identified 5,335 records. After duplicates and limits were applied (English language, date restrictions 1980-May 2014), 3,049 records were considered for inclusion. Of these 3,049 records, 2,700 were excluded based on title and abstract screening. A total of 349 articles were considered potentially relevant and were assessed at full-text by two researchers (Kappa 0.70, p<0.001). Of 349 publications,

For peer review only - http://bmjopen.bmj.com/site/about/guidelines.xhtml

33 were not obtainable (requested through the British Library), leaving 314 articles assessed at full-text stage. From these, 80 articles met inclusion criteria.

The reference lists of all included articles were screened for potentially relevant publications, resulting in a further 30 articles that met the inclusion criteria. A total of 110 articles, including over 29,726 participants, were included in the final review (Figure 2). The total number of participants per study ranged from 8-2185 (mean=286.54; median: 134.00). Six studies did not report sample size, thus the sample size calculations represented above are based on 104 articles.[24-29] See eTable 1 for full data extraction.

FIGURE 2 HERE

Study characteristics

Empirical study types and design

In total 110 articles were included; these consisted of 76 quantitative studies (including 72 questionnaire-based studies, 1 secondary analysis of data study, 1 case control study, 1 descriptive study and 1 cohort study) , 21 qualitative studies (including 11 interview-based studies and 10 focus group studies) and 13 mixed-methods studies (1 semi-structured interview and documentary analysis-based study; 1 semi-structured interview and retrospective review of error reports-based study; 2 semi-structured interview and questionnaire-based study; 3 focus group and questionnaire-based studies; 1 semi-structured and structured interview-based study; 1 interview, focus group and analysis of event reports-based study; 1 focus group and semi-structured interview-based study; 1 retrospective analysis of

For peer review only - http://bmjopen.bmj.com/site/about/guidelines.xhtml

routinely collected data and questionnaire-based study; 2 focus groups, interview and questionnaire-based studies).

Countries (Table 3)

The review encompassed research spanning four continents and over 20 countries. The four countries contributing the most studies were the United States of America (n=33), the United Kingdom (n=24), Australia (n=8), and Canada (n=8).

TABLE 3 HERE

Year of Publication

A steady increase in articles was evident over decades: 1980's (n=1),[51] 1990's (n=12),[24, 45, 52, 54, 67, 72, 76, 80, 81, 85, 103, 121] 2000's (n=58), [8-11, 28-35, 37, 40-44, 46-50, 53, 55-59, 64, 66, 69, 74, 75, 77-79, 82, 84, 91-94, 99, 101, 107, 110, 112, 114, 116-119, 125-129] 2010-May 2014 (n=39).[25-27, 36, 38, 39, 60-63, 65, 68, 70, 71, 73, 83, 86-90, 95-98, 100, 102, 104-106, 108, 109, 111, 113, 115, 120, 122-124] This increase is likely to reflect the growing integration of incident reporting systems in healthcare systems worldwide and the increasing realisation that healthcare professionals (HCPs) engagement in incident reporting is far from ideal.

The frequency of barriers and facilitators to incident reporting across the 110 articles, was calculated and rank ordered across the data (Figure 3). Where contributing factors were found not to be barriers or facilitators to incident reporting (e.g. if fear was found not to be a significant predictor of decreased or increased incident reporting), these were counted as negative cases. These negative cases were included to provide a more complete view of the data, and to prevent reporting bias.

For peer review only - http://bmjopen.bmj.com/site/about/guidelines.xhtml

When the same barrier, facilitator or negative case (e.g. fear of adverse consequences) was mentioned more than once within an article, this was reflected in the frequency data presented. In total, 748 barriers to incident reporting were identified (frequency count) compared with 372 facilitators. A total of 118 negative cases were identified. The top two barriers cited were fear of adverse consequences (161, representing 21.52% of barriers) and process and systems of reporting (110, representing 14.71% of barriers). In comparison, the top two facilitators were organisational (97, representing 26.08% of facilitators) and process and systems of reporting (75, representing 20.16% of facilitators). These results illustrate that the factors identified in this review of the literature can act as both a barrier and a facilitator to incident reporting systems depending on context; for example, *process and systems of reporting* was found to be the second most frequently cited barrier, as well as the second most frequently cited facilitator to incident reporting engagement. Whilst this may initially appear contradictory, when considering the complexity/simplicity of reporting it was found that highly complex incident reporting processes and systems were a barrier to incident reporting, whereas simple processes and systems were found to be a facilitator.

FIGURE 3 HERE

Frequency of Barriers to Patient Safety Incident Reporting (eTable 2)

Barriers to incident reporting were mentioned 748 times across the 110 articles (see eTable 2). The three most frequently mentioned barriers to incident reporting included *fear of adverse consequences* (161/748), *process and systems of reporting* (110/748) and *incident characteristics* (92/748).

For peer review only - http://bmjopen.bmj.com/site/about/guidelines.xhtml

Fear of Adverse Consequences

Fear of adverse consequences, as a barrier, was mentioned 161 times, and included a general fear of adverse consequences associated with incident reporting (51/161),[8, 10, 11, 27, 30, 32, 33, 35-37, 42-45, 53-56, 58, 59, 61, 68, 75, 78, 79, 85, 87, 88, 92, 97, 99, 100, 104, 106, 109, 118, 120, 121] fear of litigation (30/161), [8-11, 24, 27, 32, 35, 48, 51, 52, 61, 69, 72, 77, 80, 81, 85, 87, 88, 93, 100, 101, 103, 105, 107, 114, 117, 124, 128] and the fear of blame (24/161). [8, 10, 32, 35, 43, 44, 46, 58-61, 68, 70, 72, 78, 79, 82, 87, 90, 92, 99, 106] Additionally, the fear of judgment (22/161), [10, 24, 35, 43, 53, 59, 67, 79, 80, 88, 92, 99, 104, 107, 109, 116, 126], the fear of the negative impact that incident reporting could have on relationships with other HCPs, patients and the public (12/161), [10, 11, 36, 44, 46, 48, 54, 59, 92, 104, 116, 120] and the fear of a detrimental impact that reporting an incident could have on HCPs career (10/161), [10, 11, 27, 58, 59, 79, 86, 92, 93, 126] such as for example fear of job loss, were also cited as common barriers. Other less frequently mentioned barriers included protection of self (7/161), [24, 76, 80, 107, 122, 127] avoidance of discussion in meetings (4/161), [8, 69, 87, 117] and apprehension of sending an inappropriate form (1/161).[75]

Process and Systems of Reporting

Process and systems of reporting was mentioned as a barrier to reporting 110 times. The most frequently identified barrier to incident reporting was the time required to complete an incident report (29/110), [8, 11, 27, 38, 43, 48, 57, 69, 74, 78, 79, 81, 85, 87, 88, 90, 92, 93, 99-101, 105-107, 114, 118, 121] followed by the complexity of the reporting process (28/110). [8, 9, 11, 31, 33, 35, 38, 44, 46, 51, 73, 78, 79, 88-90, 93, 100, 101, 105-107, 117, 118, 125] Other process and systems of reporting barriers included lack of anonymity and/or confidentiality in reporting (22/110), [8, 11, 24, 27, 35, 48, 50, 68, 73, 74, 76-78, 80, 87, 101, 106, 107, 127] reporting format

For peer review only - http://bmjopen.bmj.com/site/about/guidelines.xhtml

(10/110), [31, 44, 82, 85, 90, 93, 100, 117] and the type of reporting system (e.g. paper-based) (5/110). [38, 50, 92, 117] Less frequently mentioned barriers included lack of information to complete report (3/110), [94, 107, 114] the focus of reporting (1/110), [78] and information to complete report not readily being available (1/110). [31]

Incident Characteristics

Incident characteristics were mentioned as a barrier to reporting 92 times. Level of harm, cause of incident, and frequency of incident were the most frequent incident characteristics acting as barriers to reporting (40/92, 19/42, and 18/92, respectively). HCPs were less likely to report an incident if the patient experienced no or minimal harm. [8, 11, 24, 31, 35, 42-48, 50, 51, 53, 54, 58, 65, 66, 69, 70, 72, 73, 80, 85, 87, 88, 92, 100, 103, 105, 106, 109, 114, 126, 128, 129] Incidents that were deemed to occur frequently were considered too well-known to report. [31, 51, 66, 70, 75, 76, 84, 100, 101, 103, 114, 119, 121, 127-129] Furthermore, if the cause of the incident was deemed unpreventable this acted as a barrier to incident reporting. [35, 52, 66, 81, 82, 85, 100, 101, 103, 107, 114, 119, 124, 128, 129] Other barriers included the type of incident (13/92) [8, 33, 34, 52, 69, 81, 85, 92, 93, 100, 107, 117, 121] and the level of risk (2/110). [11, 58]

Individual HCP Characteristics

Barriers reflective of individual HCP characteristics were cited 89 times. Barriers included a negative attitude/lack of value placed on incident reporting (53/89), [8, 9, 35, 44, 46, 56, 61, 63, 64, 66, 68, 70, 73, 74, 76, 79, 81, 86-88, 92, 93, 99-101, 103, 105, 107, 109, 117, 118, 120, 121, 128] and the perception that incident reporting does not result in improvements typically underlined such negative attitudes and values. A number of studies found that HCPs fail to report incidents because they simply forget (9/89), [8, 27, 31, 72, 87, 93, 117, 119, 129]

For peer review only - http://bmjopen.bmj.com/site/about/guidelines.xhtml

and that the way HCPs perceive themselves can act as a barrier to reporting (9/89).[24, 36, 55, 80, 87, 107, 127] Less frequently mentioned barriers included emotional responses to the incident (6/89),[31, 58, 79, 82, 100] previous reporting behavior (5/89),[34, 37, 52, 60, 74] exposure to errors (2/89), [38, 97] and length of time in employment (2/89).[37]

Knowledge and Skills

Knowledge and skills were cited as barriers to incident reporting 84 times. The two most frequently mentioned barriers related to a lack of reporting clarity (36/84) [9, 11, 24, 27, 31, 35, 38, 44, 46, 51, 52, 70, 73, 76, 79, 80, 87, 88, 100, 101, 103, 105, 107, 114, 119, 121, 127, 128] and a lack of clarity regarding what constitutes an adverse event and/or near miss (31/84).[9, 11, 31, 35, 43, 44, 46, 51, 69, 74, 82, 85, 87, 88, 92, 93, 95, 99, 100, 105, 117, 121] This suggests that a lack of knowledge about what should be reported and how to do this act as barriers. Less frequently cited barriers included an inability in error recognition (7/84),[35, 75, 79, 92, 99, 106, 124] lack of training in reporting (5/84), [68, 76, 82, 86, 97] and lack of awareness (4/84).[35, 43, 106, 114]

Work Environment

Work environment was mentioned 80 times as a barrier to incident reporting. Workload/Priority (50/80) [9, 11, 24, 27, 31, 34, 35, 43, 48, 49, 51, 55-58, 61, 68-70, 72, 75-77, 80, 82, 83, 88-90, 92, 93, 100, 103, 117, 119, 120, 125, 127-129] and accessibility (27/80) [24, 27, 31, 34, 35, 51, 52, 56, 74, 75, 80, 82, 86, 93, 101, 105-107, 114, 117, 119, 121, 127] were the most frequently mentioned work environment barriers, suggesting that high workload does not allow for incident reporting to be prioritised, and that access to the reporting system is problematic (e.g. not enough computer work stations to access reporting forms).

For peer review only - http://bmjopen.bmj.com/site/about/guidelines.xhtml

Organisational Factors

Organisational factors were mentioned 76 times as a barrier to incident reporting. Lack of feedback and communication following incident reporting (26/76) [8, 9, 11, 35, 37, 43, 44, 56, 58, 59, 61, 62, 69, 78, 85-87, 90, 92, 99, 100, 106, 108, 117, 123] and the absence/lack of a positive reporting culture (17/76) [9, 10, 34, 35, 49, 66, 70, 81, 86, 90, 92, 114, 117, 118, 123] were the two most frequently mentioned organisational barriers to reporting. Less frequently mentioned were lack of organisational learning and improvement (7/76),[27, 35, 61, 68, 69, 85, 100] poor organisational use of data (7/76),[43, 59, 61, 92, 99] and poor management response to reports (5/76). [55, 68, 79, 92, 112]

Team Factors

Team factors were mentioned as barriers to engagement in incident reporting 33 times. The three most frequently mentioned barriers included the negative impact that incident reporting could have on working relationships (13/33),[11, 27, 32, 55, 58, 66, 74, 87, 88, 90, 100] the influence of seniors not to report (7/33), [37, 42, 74, 82, 106, 110] and how HCPs feel about reporting their peers (5/33).[79, 85, 103]

Professional Ethics

Professional ethics was the least frequently mentioned barrier to incident reporting (23/748). The most prevalent factor was a lack of personal responsibility to report (15/23) [8, 9, 34, 35, 44, 52, 70, 93, 94, 100, 104, 118, 121, 128] with studies suggesting that HCPs are less likely to report when they feel that reporting is the responsibility of someone else within the team. Concealment was also mentioned as a barrier (5/23).[85, 87, 120]

For peer review only - http://bmjopen.bmj.com/site/about/guidelines.xhtml

Frequency of Facilitators in Patient Safety Incident Reporting (Table e1)

Facilitators of reporting were mentioned 372 times across the 110 articles (see Table 2). Organisational factors were the most frequently mentioned facilitator to incident reporting (97/372), followed by process and systems of reporting (75/372) and incident characteristics (55/372).

Organisational Factors

Organisational factors were mentioned as facilitators 97 times. The two most frequently cited facilitators included the provision of feedback/communication following incident reporting (29/97) [9, 11, 30, 33, 41, 44, 46, 61, 65, 68, 70, 75-77, 87, 100, 101, 107, 112, 117] and a non-punitive incident reporting policy (22/97). [9, 11, 29, 30, 32, 33, 40, 46, 58, 68, 75-77, 81, 87, 101, 106, 107] The existence of a reporting culture (16/97) [29, 33, 39, 66, 75, 96, 100, 106, 110-112, 121, 122] and a focus on learning and improvement from incidents (13/97) [9, 31, 40, 61, 68, 70, 85, 90, 100, 110] were also facilitators to reporting.

Process and Systems of Reporting

Process and systems of reporting was mentioned as a facilitator 75 times. Reporting format, ensuring anonymity and/or confidentiality, and simplification of reporting were the three most frequently cited facilitators accounting for 21/75,[9, 11, 25, 30, 44, 46, 58, 61, 65, 68, 70, 75, 87, 100, 106, 107, 117] 16/75, [9, 11, 29, 31, 40, 44, 65, 68, 74, 87, 100, 106, 117] and 15/75 [9, 11, 30, 38, 65, 68, 73, 77, 81, 100, 101, 117] facilitators within this category. Less frequently mentioned process and systems of reporting facilitators included the type of reporting system used (e.g. electronic reporting) (11/75). [33, 34, 40, 44, 68, 73, 101, 117]

For peer review only - http://bmjopen.bmj.com/site/about/guidelines.xhtml

Incident Characteristics

Incident characteristics were mentioned as a facilitator to reporting 55 times. Level of harm and frequency of an incident were the most frequently cited incident characteristics identified as facilitators to reporting (26/55 [11, 31, 40, 42, 47, 50, 58, 66, 75, 77, 82, 85, 88, 95, 114, 121, 124, 125, 128] and 13/55, [11, 66, 75, 77, 114, 121, 124] respectively). Incidents resulting in severe harm (including death) were more likely to be reported and HCPs were more likely to report incidents that occur infrequently rather than frequently. Less frequently mentioned facilitators included the type of incident (8/55), [82, 85, 121] cause of the incident (6/55), [40, 66, 76, 77, 125] and level of risk (1/55).[58]

Individual HCP Characteristics

Individual HCP characteristics were mentioned 41 times as a facilitator. A positive attitude towards incident reporting and a high value placed on incident reporting was found to increase the likelihood of reporting (21/41). [9, 11, 40, 58, 68, 82, 88, 90, 93, 95, 97, 98, 107, 111, 125] HCPs emotional response to a patient safety incident was also found to increase the likelihood of reporting in a number of studies (5/41).[31, 58, 100] The professional group of HCPs was also found to act as a facilitator to reporting (5/41). [28, 71] Less frequently cited facilitators included previous reporting behavior (1/41), [29] number of hours worked (1/41), [52] and demographics (e.g. gender and age) (2/41). [37, 98]

Knowledge and Skills

Training in reporting was identified as the most frequently mentioned facilitator in this category (21/36).[9, 25, 33, 70, 73, 75, 76, 87, 101, 106, 117, 127] Other facilitators included knowledge regarding what constitutes an adverse event/near miss and the ability to

For peer review only - http://bmjopen.bmj.com/site/about/guidelines.xhtml

recognise an error has occurred (7/36 [9, 30, 44, 46, 70, 87, 100] and 4/36, [75-77, 124] respectively).

Team Factors

Team factors were mentioned 20 times as a facilitator to reporting. Good teamwork/communication (7/20) [39, 75, 77, 122] and a positive team culture (4/20) [98, 107, 111, 122] were the most frequently cited facilitators.

Professional Ethics

Professional ethics was cited as a facilitator 17 times. A strong sense of duty (8/17) [75, 85, 88, 95, 101, 107] and responsibility (5/17) [77, 90, 91, 94] to report increased the likelihood of reporting. Less frequently cited facilitators included accountability (2/17) [88, 121] and a legal obligation to report (1/17).[37]

Work Environment

Work environment was mentioned as a facilitator 18 times. Access to the incident reporting system (11/18), [30, 68, 73-75, 87, 100, 101, 117] and those whose workloads allowed for and those that prioritised incident reporting increased the likelihood of reporting.

Fear of Adverse Consequences

Fear of adverse consequences was mentioned as a facilitator to reporting 13 times and included a fear of litigation and fear of blame increasing the likelihood of reporting (8/13 [9, 11, 27, 33, 82, 88, 90] and 4/13,[9, 11, 87, 88] respectively).

Frequency of Negative Cases (Table e1)

For peer review only - http://bmjopen.bmj.com/site/about/guidelines.xhtml

Negative cases were identified 118 times across the 110 articles (see Table 2). The three most frequently mentioned factors included individual HCP characteristics (43/118), organisational factors (22/118), and knowledge and skills (15/118).

Individual HCP characteristics were mentioned as a negative case 43 times. HCP's attitude and value of incident reporting did not have an impact on reporting behavior (12/43). [37, 48, 54, 72, 79, 96, 129] Similarly, HCPs demographics (e.g. age, gender) had no impact on the likelihood of reporting (12/43).[37, 49, 51, 52, 77, 96, 97, 125, 129] Other less frequently mentioned factors included seniority (4/43),[37, 77, 125, 129] forgetfulness (1/43),[129] previous reporting behavior (1/43),[129] and number of hours worked (1/43).[26] Organisational factors were cited as having no impact on incident reporting 22 times. The most frequently mentioned were the ownership of the organisation (e.g. private/public funded) (6/22)[25, 77] and management response towards incident reporting (4/22).[29, 97, 115] Knowledge and skills were mentioned 15 times. These included the clarity of the reporting mechanism (5/15),[29, 48, 72, 129] knowledge of what constitutes an adverse event/near miss (2/15),[48, 72] ability in error recognition (1/15),[48] and training in error reporting (7/15).[25, 77, 86, 129]

Fear of adverse consequences was cited as having no impact on engagement in incident reporting 12 times. These included a fear of litigation (4/12),[24, 40, 48, 90] a general fear of adverse consequences (3/12),[72, 85, 96] blame (1/12) [48], judgment (1/12),[101] and impact on career (1/12).[125] Work environment was mentioned as as having no impact on reporting 10 times, including workload/priority (3/10)[51, 123, 125] and unit type (3/10).[49, 112] Other less frequently cited work environment factors

For peer review only - http://bmjopen.bmj.com/site/about/guidelines.xhtml

included physical work conditions (1/10),[26] satisfaction with work environment (1/10),[113] and accessibility (1/10).[48]

Across all studies, process and systems of reporting was mentioned 7 times as having no impact on incident reporting; these included reporting format (3/7),[25, 68, 125] complexity/simplification of reporting (1/7),[68] and anonymity and/or confidentiality (1/7).[24] Professional ethics were only mentioned four times as having no impact on the likelihood of incident reporting; these were legal obligation (2/4),[37] duty (1/4),[125] and responsibility (1/4).[26] Team factors were cited as having no impact on the likelihood of reporting 3 times, including teamwork and communication (2/3)[123] and support/encouragement to report (1/3).[109] Incident characteristics were the least frequently mentioned factor which had no impact on reporting. Cause of incident was found to have no impact on engagement in reporting (2/2).[125, 129]

For peer review only

Discussion

It has been suggested that there is a tendency in healthcare to encourage reporting of any and all patient safety incidents, to celebrate large quantities of incident reports and to aim for ever-increasing overall reporting rates. Whilst there are numerous problems associated with this approach[7] (e.g. flooding the system to such a degree that the thorough investigation of each incident reporting is unachievable), it is clear that high levels of underreporting seriously compromises the ability of incident reporting systems to facilitate learning and improvement in patient safety.

This is the first theoretical literature review of factors contributing to patient safety incident reporting. Based on the evidence from 110 articles, we developed a theoretical framework, based on the principles of grounded theory, which summarises a wide range of factors contributing to incident reporting. We purposely sought publications from a range of countries, covering diverse health systems and study populations with a view to incorporating these into one broad theoretical framework. We argue that this is an appropriate approach for this initial explorative work, as multiple theoretical frameworks for individual counties, settings and populations (e.g. nurses working in mental health settings in Australia), would have limited application at this point in time. However, we suggest that those interested in exploring barriers and facilitators in specific settings conduct further research using the theoretical framework presented here.

To improve incident reporting (both the quantity and/or quality) and facilitate the successful implementation of incident reporting systems, we suggest that the

For peer review only - http://bmjopen.bmj.com/site/about/guidelines.xhtml

theoretical framework is best used to prospectively and systematically identify factors within a given context that are likely to affect incident reporting. Those responsible for the effective implementation of incident reporting systems should explore each of the factors listed in our framework for salience. Rather than the framework being used in isolation, we recommend that it be used in conjunction with other implementation theories/frameworks and models to guide, understand and evaluate implementation of incident reporting systems.[130] Based on such prospective analysis, strategies to enhance the adoption, implementation, and sustainability of incident reporting systems can be tailored and selected according to a given setting. As such, using the developed framework will advance our understanding of how to optimally implement incident reporting systems into practice.

We used the developed theoretical framework, based on the evidence-base, to organise our findings and have presented the frequency and rank order (i.e. prevalence) of factors contributing to incident reporting. Whilst this approach is consistent with other frameworks in the patient safety literature,[14, 23] it may be considered as a crude analysis of the existing literature and needs to be interpreted with caution; we acknowledge that it is possible, although unlikely, that a relationship between the number of times a given factor is mentioned in the literature and its impact on incident reporting behaviour might not exist. However, we have been able to provide the first high level overview of a large heterogeneous body of evidence. Furthermore, we acknowledge that weighting the impact of each factor would have been advantageous, however the data did not lend itself to this possibility and we propose that it might not be possible to simply weight factors because of the complex and dynamic interrelationships that are likely to exist between them. Alternatively, we

For peer review only - http://bmjopen.bmj.com/site/about/guidelines.xhtml

For peer review only

suggest that modelling the interrelationships between factors affecting incident reporting engagement is an avenue for future research.

Our results suggest that fear of adverse consequences and ineffective processes/systems of reporting are high priority areas that require consideration to improve engagement in incident reporting. Changes to policy should be considered at an institutional or national level to prevent fear of litigation and blame, as fear of adverse consequences was found to inhibit incident reporting. We believe that it is unlikely that changes made within a single hospital or healthcare system would instill significant reassurance to promote incident reporting. In addition, at an organisational level we found that appropriate systems and processes for reporting need to be implemented to improve incident reporting; simultaneously, lack of, or poorly designed systems significantly hinder reporting. These aspects of reporting rely on well-designed processes and technologies and are arguably the responsibility of the organisational leaders. There is no 'optimum model' for incident reporting systems (e.g. electronic, confidential, anonymous) - systems need to be responsive to users and organisational needs.

Organisational factors and processes/systems of reporting were identified as the two most frequently cited facilitators of reporting, which suggests that healthcare organisations consider these as high priority areas which should be the target of increased focus and resources. For example, our results suggest that organisational policies that foster a reporting and learning culture as well as providing feedback following a report will promote incident reporting. Interestingly, we found that individual HCP characteristics have little impact on engagement in incident reporting.

For peer review only - http://bmjopen.bmj.com/site/about/guidelines.xhtml

This suggests that organisations should be cautious before investing significant resources in these factors, as such investment may result in minimal returns.

Although we have considered the above factors in isolation as illustrative examples, it is important to consider the interconnecting relationships between factors in order to develop intervention packages to improve engagement in incident reporting. Our results suggest that a comprehensive intervention/policy package which targets more than one contributing factor (e.g. establishing a supportive work environment, with mechanisms which optimise shared learning, alongside a national policy to minimise the fear of adverse consequence) is far more likely to result in increased engagement in incident reporting in comparison to interventions that simply target one factor.

Strengths and Limitations

In order to identify as much relevant literature as possible, we have included quantitative, qualitative and mixed methods research and have not restricted the literature to specific incident reporting systems, i.e. departmental, local, regional and national. In addition, the studies included a vast array of health care settings and providers, maximising the generalisability of the results. The resulting evidence has been synthetised into a practical output i.e. a theoretical framework to guide efforts to improve engagement in incident reporting.

The results, and recommendations proposed in this evidence synthesis must be considered in light of several limitations. First, only articles published in English were included, which may generate bias. However, articles spanning four continents from

For peer review only - http://bmjopen.bmj.com/site/about/guidelines.xhtml

over 20 countries were identified, hence we are confident that our findings are of high external validity to guide safety policy globally. Secondly, the last systematic search for literature was conducted on 29/05/2014, meaning that literature published since this date will not have been included. Thirdly, the decision not to include studies detailing interventions to improve incident reporting and studies detailing variations in engagement in incident reporting may skew the findings. This decision was made as it was not possible to determine the relative contribution of individual factors on engagement in incident reporting within such studies. Fourthly, large heterogeneity across studies in terms of outcome measures and methodologies meant conduction of meta-analysis was precluded. This having been said, the synthesis of barriers and facilitators into frequency of reporting provides some evidence towards their respective relative importance, although it is accepted that the frequency of factors may represent those that have been the subject of more research. We recommend that future research applies and evaluates the usefulness of the developed theoretical framework in exploring and improving incident reporting in a variety of settings (e.g. primary and secondary healthcare).

Summary/conclusion

A wide range of factors contributing to engagement in incident reporting exist across varying levels of the healthcare system. Efforts aimed at addressing the current tendency to underreport must consider the full range of factors in order to develop tailored interventions and policy packages for improvement. We suggest the theoretical framework developed here would be useful in understanding factors affecting incident reporting engagement, increasing engagement in incident reporting and ultimately learning from patient safety incidents.

For peer review only - http://bmjopen.bmj.com/site/about/guidelines.xhtml

Acknowledgements:

We would like to thank Kelsey Flott, BA, MSc, from the Centre for Health Policy, Imperial College London, for critically reviewing the manuscript.

Competing Interests:

We declare that we have no competing interests.

Ethical Approval:

None

Funding:

This article represents independent research supported by the National Institute for Health Research (NIHR) Imperial Patient Safety Translational Research Centre. The views expressed are those of the author(s) and not necessarily those of the NHS, the NIHR or the Department of Health. Hull and Sevdalis' research was supported by the National Institute for Health Research (NIHR) Collaboration for Leadership in Applied Health Research and Care South London at King's College Hospital NHS Foundation Trust. Hull and Sevdalis are members of King's Improvement Science, which is part of the NIHR CLAHRC South London and comprises a specialist team of improvement scientists and senior researchers based at King's College London. Its work is funded by King's Health Partners (Guy's and St Thomas' NHS Foundation Trust, King's College Hospital NHS Foundation Trust, King's College London and South London and Maudsley NHS Foundation Trust), Guy's and St Thomas' Charity, the Maudsley Charity and the Health Foundation. The views expressed are those of

For peer review only

the authors and not necessarily those of the NHS, the NIHR or the Department of Health.

Authorship/Contributor statement:

- Conception or design of the work: Stephanie Archer, Louise Hull, Erik Meyer, Nick Sevdalis, Ara Darzi
- Data collection: Stephanie Archer, Louise Hull, Tayana Soukup
- Data analysis and interpretation Stephanie Archer, Louise Hull, Thanos Athanasiou, Nick Sevdalis
- Drafting the article: Stephanie Archer, Thanos Athanasiou, Nick Sevdalis
- Critical revision of the article: Stephanie Archer, Louise Hull, Tayana Soukup, Erik Meyer, Thanos Athanasiou, Nick Sevdalis, Ara Darzi
- Final approval of the version to be published: Stephanie Archer, Louise Hull, Tayana Soukup, Erik Meyer, Thanos Athanasiou, Nick Sevdalis, Ara Darzi

Data sharing

All data from this systematic review and theoretical framework is presented within the publication.

For peer review only - http://bmjopen.bmj.com/site/about/guidelines.xhtml

References

1. Barach P, Small S. Reporting and preventing medical mishaps: lessons from non-medical near miss reporting systems. *BMJ* 2000;320:759-63.

2. Leape L. Error in medicine. *JAMA* 1994;272:1851-57.

3. Department of Health. An organisation with a memory: report of an expert group on learning from adverse events in the NHS. Available from: https://psnet.ahrq.gov/resources/resource/1568. Last accessed March 2017.

4. Nobel D, Pronovost P. Undereporting of patient safety incidents reduces health-cares ability to quantify and accurately measure harm reduction. *J Patient Saf* 2010;6(4): 247-50.

5. Institute of Medicine. To err is human: building a safety health system. Washington, DC: National Academy Press; 1999.

6. Howell AM, Burns EM, Hull L, et al. . International recommendations for national patient safety incident reporting systems: an expert Delphi consensus-building process. *BMJ Qual Saf* Online first: 22 Feb 2016. . doi: 10.1136/bmjqs-2015-004456. Online First: 5 February 2004. doi:10.1136/ard.2003.001234

7. Macrae C. The problem with incident reporting. *BMJ Qual Saf* 2016;25(2):71-5.

8. Evans SM, Berry JG, Smith BJ, et al. Attitudes and barriers to incident reporting: A collaborative hospital study. *Qual Saf Health Care* 2006;15(1):39-43.

9. Kingston MJ, Evans SM, Smith BJ, et al. Attitudes of doctors and nurses towards incident reporting: A qualitative analysis. *Med J Aust* 2004;181(1):36-9.

10. Waring JJ. Beyond blame: Cultural barriers to medical incident reporting. *Soc Sci Med* 2005;60(9):1927-35.

11. Jeffe DB, Dunagan WC, Garbutt J, et al. Using focus groups to understand physicians' and nurses' perspectives on error reporting in hospitals. *Jt Comm J Qual Saf* 2004;30(9):471-9.

For peer review only - http://bmjopen.bmj.com/site/about/guidelines.xhtml

12. Polisena J, Gagliardi A, Urbach D, et al. Factors that influence the recognition, reporting and resolution of incidents related to medical devices and other healthcare technologies: a systematic review. *Syst Rev* 2015:29;4:37.

13. Fung WM, Koh SS, Chow YL. Attitudes and perceived barriers influencing incident reporting by nurses and their correlation with reported incidents: A systematic review. *JBI Libr Syst Rev* 2012;10(1):1-65.

14. Pfeiffer Y, Manser T, Wehner T. Conceptualising barriers to incident reporting: a psychological framework. *Qual Saf Health Care* 2010;19(6):e60.

15. O'Connor E, Coates HM, Yardley IE, et al. Disclosure of patient safety incidents: a comprehensive review. *Int J Qual Health Care* 2010;22(5):371-9.

16. Higgins JP, Altman DG, Gøtzsche PC, et al. The Cochrane Collaboration's tool for assessing risk of bias in randomised trials. *BMJ* 2011;343:d5928.

17. Shea BJ, Grimshaw JM, Wells GA, et al. Development of AMSTAR: a measurement tool to assess the methodological quality of systematic reviews. *BMC Med Res Methodol* 2007;7:10.

18. Long AF, Godfrey M. An evaluation tool to assess the quality of qualitative research studies. *Int J Social Research Methodology* 2004;7(2):181-96.

19. Paré G, Trudel MC, Jaanac M, et al. Synthesizing information systems knowledge: A typology of literature reviews. *Information & Management* 2015;52(2):183-99.

20. Sanderson S, Tatt ID, Higgins JP. Tools for assessing quality and susceptibility to bias in observational studies in epidemiology: a systematic review and annotated bibliography. *Int J Epidemiol* 2007;36(3):666-76.

21. Glaser B, Strauss A. The Discovery of Grounded Theory. Hawthorne, New York: Aldine Publishing Company; 1967.

22. Wolfswinkel J, Furtmueller E, Milderon C. Using grounded theory as a method for rigorously reviewing literature. *Eur J Inf Syst* 2013;22(1):45-55.

For peer review only - http://bmjopen.bmj.com/site/about/guidelines.xhtml

23. Lawton R, McEachan RR, Giles SJ, et al. Development of an evidence-based framework of factors contributing to patient safety incidents in hospital settings: a systematic review. *BMJ Qual Saf* 2012;21(5):369-80.

24. Belton, KJ. Attitude survey of adverse drug-reaction reporting by health care professionals across the European Union. *Eur J Clin Pharmacol* 1997;52(6):423-7.

25. Fukuda H, Imanaka Y, Hirose M, et al. Impact of system-level activities and reporting design on the number of incident reports for patient safety. *Qual Saf Health Care* 2010;19(2):122-7.

26. Gaal S, van den Hombergh P, Verstappen W, et al. Patient safety features are more present in larger primary care practices. *Health Policy* 2010;97(1): 87-91.

27. Jennings PA, Stella J. Barriers to incident notification in a regional prehospital setting. *Emerg Med J* 2011;28(6):526-9.

28. Rowin EJ, Lucier D, Pauker SG, et al. Does error and adverse event reporting by physicians and nurses differ? *Jt Comm J Qual Patient Saf* 2008;34(9):537-45.

29. Hutchinson A, Young TA, Cooper KL, et al. Trends in healthcare incident reporting and relationship to safety and quality data in acute hospitals: Results from the National Reporting and Learning System. *Qual Saf Health Care* 2009;18(1):5-10.

30. Beasley JW, Escoto KH, Karsh BT. Design Elements for a Primary Care Medical Reporting System. *WMS* 2004;103(1):56-9.

31. Elder NC, Graham D, Brandt E, et al. Barriers and motivators for making error reports from family medicine offices: a report from the American Academy of Family Physicians National Research Network (AAFP NRN). *J Am Board Fam Med* 2007;20(2):115-23.

32. Fairbanks RJ, Crittenden CN, O'Gara KG, et al. Emergency medical services provider perceptions of the nature of adverse events and near-misses in out-of-hospital care: An ethnographic view. *Acad Emerg Med* 2008;15(7):633-40.

33. Karsh BT, Escoto KH, Beasley JW, et al. Toward a theoretical approach to medical error reporting system research and design. *Appl Ergon* 2006;37(3):283-95.

For peer review only - http://bmjopen.bmj.com/site/about/guidelines.xhtml

34. Kennedy AG, Littenberg B. Medication error reporting by community pharmacists in Vermont. *J Am Pharm Assoc* 2004;44(4):434-8.

35. Handler SM, Perera S, Olshansky EF, et al. Identifying Modifiable Barriers to Medication Error Reporting in the Nursing Home Setting. *J Am Med Dir Assoc* 2007;8(9):568-74.

36. Church JA, Adams RD, Hendrix LH, et al. National study to determine the comfort levels of radiation therapists and medical dosimetrists to report errors. *Pract Radiat Oncol* 2013;3(4):e165-70.

37. Daly JM, Jogerst GJ. Association of knowledge of adult protective services legislation with rates of reporting of abuse in Iowa nursing homes. *J Am Med Dir Assoc* 2005;6(2):113-20.

38. Ehrenpreis ED, Sifuentes H, Ehrenpreis JE, et al. Suboptimal reporting of adverse medical events to the FDA Adverse Events Reporting System by nurse practitioners and physician assistants. *Expert Opin Drug Saf* 2012;11(2): 177-83.

39. Erler C, Edwards NE, Ritchey S, et al. Perceived Patient Safety Culture in a Critical Care Transport Program. *Air Med J* 2013;32(4):208-15.

40. Garbutt J, Brownstein DR, Klein EJ, et al. Reporting and disclosing medical errors: Pediatricians' attitudes and behaviors. *Arch Pediatr Adolesc Med* 2007;161(2):179-85.

41. Kaldjian LC, Jones EW, Wu BJ, et al. Reporting medical errors to improve patient safety: A survey of physicians in teaching hospitals. *Arch Intern Med* 2008;168(1):40-6.

42. Hohenhaus SM. Emergency Nursing and Medical Error-A Survey of Two States. *J Emerg Nurs* 2008;34(1):20-5.

43. Patrician PA, Brosch LR. Medication error reporting and the work environment in a military setting. *J Nurs Care Qual* 2009;24(4):277-86.

44. Schectman JM, Plews-Ogan ML. Physician perception of hospital safety and barriers to incident reporting. *Jt Comm J Qual Patient Saf* 2006;32(6):337-43.

For peer review only - http://bmjopen.bmj.com/site/about/guidelines.xhtml

45. Schulmeister L. Chemotherapy medication errors: descriptions, severity, and contributing factors. *Oncol Nurs Forum* 1999;26(6):1033-42.

46. Taylor JA, Brownstein D, Christakis DA. Use of incident reports by physicians and nurses to document medical errors in pediatric patients. *Pediatrics* 2004;114(3): 729-35.

47. Throckmorton T, Etchegaray J. Factors Affecting Incident Reporting by Registered Nurses: The Relationship of Perceptions of the Environment for Reporting Errors, Knowledge of the Nursing Practice Act, and Demographics on Intent to Report Errors. *J Perianesth Nurs* 2007;22(6):400-12.

48. Uribe CL, Schweikhart SB, Pathak DS, et al. Perceived barriers to medical-error reporting: An exploratory investigation. *J Healthc Manag* 2002;47(4): 263-79.

49. Vogus TJ, Sutcliffe KM. The impact of safety organizing, trusted leadership, and care pathways on reported medication errors in hospital nursing units. *Med Care* 2007;45(10):997-1002.

50. Weissman JS, Annas CL, Epstein AM, et al. Error reporting and disclosure systems: views from hospital leaders. *JAMA* 2005;293(11): 1359-66.

51. Rogers AS, Israel E, Smith CR, et al. Physician knowledge, attitudes, and behavior related to reporting adverse drug events. *Arch Intern Med* 1988;148(7):1596-1600.

52. Generali JA, Danish MA, Rosenbaum SE. Knowledge of and attitudes about adverse drug reaction reporting among Rhode Island pharmacists. *Ann Pharmacother* 1995;29(4):365-9.

53. Mayo AM, Duncan D. Nurse Perceptions of Medication Errors: What We Need to Know for Patient Safety. *J Nurs Care Qual* 2004;19(3): 209-17.

54. Osborne J, Blais K, Hayes JS. Nurses' Perceptions: When Is It a Medication Error? *J Nurs Adm* 1999;29(4):33-8.

55. Blegen MA, Vaughn T, Pepper G, et al. Patient and Staff Safety: Voluntary Reporting. *Am J Med Qual* 2004;19(2):67-74.

For peer review only

For peer review only - http://bmjopen.bmj.com/site/about/guidelines.xhtml

56. King ES, Moyer DV, Couturie MJ, et al. Getting doctors to report medical errors: project DISCLOSE. *Jt Comm J Qual Patient Saf* 2006;32(7): 382-92.

57. Coley KC, Pringle JL, Weber RJ, et al. Perceived Barriers in Using a Region-Wide Medication Error Reporting System. *J Patient Saf* 2006;2(1):39-44.

58. Elder, NC, Brungs SM, Nagy M, et al. Nurses' perceptions of error communication and reporting in the intensive care unit. *J Patient Saf* 2008; 4(3):162-8.

59. Stratton KM, Blegen MA, Pepper G, et al. Reporting of Medication Errors by Pediatric Nurses. *J Pediatr Nurs* 2004;19(6):385-92.

60. Anderson JE, Kodate N, Walters R, et al. Can incident reporting improve safety? Healthcare practitioners' views of the effectiveness of incident reporting. *Int J Qual Health Care* 2013;25(2):141-50.

61. Arfanis K, Smith A. Informal risk assessment strategies in health care staff: An unrecognized source of resilience? *J Eval Clin Pract* 2012;18(6): 1140-6.

62. Armitage G, Newell R, Wright J. Improving the quality of drug error reporting. *J Eval Clin Pract* 2010;16(6):1189-97.

63. Turner S, Ramsay A, Fulop N. The role of professional communities in governing patient safety. *J Health Organ Manag* 2013;27(4):527-43.

64. Waring, JJ. A qualitative study of the intra-hospital variations in incident reporting. *Int J Qual Health Care* 2004;16(5): 347-52.

65. Williams SD, Phipps DL, Ashcroft DM. Understanding the attitudes of hospital pharmacists to reporting medication incidents: A qualitative study. *Res Social Adm Pharm* 2013;9(1):80-9.

66. Ashcroft DM, Morecroft C, Parker D, et al. Likelihood of reporting adverse events in community pharmacy: An experimental study. *Qual Saf Health Care* 2006;15(1):48-52.

67. Gladstone J. Drug administration errors: a study into the factors underlying the occurrence and reporting of drug errors in a district general hospital. *J Adv Nurs* 1995;22(4):628-37.

For peer review only - http://bmjopen.bmj.com/site/about/guidelines.xhtml

68. Holmström AR, Airaksinen M, Weiss M, et al. National and local medication error reporting systems: a survey of practices in 16 countries. *J Patient Saf* 2012;8(4):165-76.

69. Kreckler S, Catchpole K, McCulloch P, et al. Handa AFactors influencing incident reporting in surgical care. *Qual Saf Health Care* 2009;18(2):116-20.

70. Parvizi N, Robertson I, McWilliams RG. Medical device adverse incident reporting in interventional radiology. *Clin Radiol* 2014;69(3):263-7.

71. Sarvadikar A, Prescott G, Williams D. Attitudes to reporting medication error among differing healthcare professionals. *Eur J Clin Pharmacol* 2010;66(8):843-53.

72. Vincent C, Stanhope N, Crowley-Murphy M. Reasons for not reporting adverse incidents: An empirical study. *J Eval Clin Pract* 1999;5(1):13-21.

73. Winchester SA, Tomkins S, Cliffe S, et al. Healthcare workers' perceptions of occupational exposure to blood-borne viruses and reporting barriers: a questionnaire-based study. *J Hosp Infect* 2012;82(1):36-9.

74. Sharma, A, Jain P, Parmar, B, et al. Incident Reporting in Surgical Trainees-Revisited. *J Patient Saf* 2008;4(3):191-4.

75. Green CF, Mottram DR, Rowe PH, Pirmohamed M. Attitudes and knowledge of hospital pharmacists to adverse drug reaction reporting. *Br J Clin Pharmacol* 2001;51(1):81-6.

76. Green CF, Mottram DR, Raval D, et al. Community pharmacists' attitudes to adverse drug reaction reporting. *Int J Pharm Pract* 1999;7(2):92-9.

77. Sweis D, Wong IC. A Survey on Factors that Could Affect Adverse Drug Reaction Reporting According to Hospital Pharmacists in Great Britain. *Drug Saf* 2000;23(2): 165-72.

78. McArdle D, Burns N, Ireland A. Attitudes and beliefs of doctors towards medication error reporting. *Int J Health Care Qual Assur* 2003;16(7):326-33.

For peer review only

For peer review only - http://bmjopen.bmj.com/site/about/guidelines.xhtml

79. Sanghera IS, Franklin BD, Dhillon S. The attitudes and beliefs of healthcare professionals on the causes and reporting of medication errors in a UK Intensive care unit. *Anaesthesia* 2007;62(1):53-61.

80. Belton KJ, Lewis SC, Payne S, et al. Attitudinal survey of adverse drug reaction reporting by medical practitioners in the United Kingdom. *Br J Clin Pharmacol* 1995;39(3):223-6.

81. Bateman DN, Sanders GL, Rawlins MD. Attitudes to adverse drug reaction reporting in the Northern Region. *Br J Clin Pharmacol* 1992;34(5):421-6.

82. Haines TP, Cornwell P, Fleming J, et al. Documentation of in-hospital falls on incident reports: qualitative investigation of an imperfect process. *BMC Health Serv Res* 2008;11;8:254.

83. Tariq A, Georgiou A, Westbrook J. Medication incident reporting in residential aged care facilities: limitations and risks to residents' safety. *BMC Geriatr* 2012;12:67.

84. Johnstone MJ, Kanitsaki O. Patient safety and the integration of graduate nurses into effective organizational clinical risk management systems and processes: an Australian study. *Qual Manag Health Care* 2008;17(2):162-73.

85. Walker SB, Lowe MJ. Nurses' views on reporting medication incidents. *Int J Nurs Pract* 1998;4(2):97-102.

86. Braithwaite J, Westbrook MT, Travaglia JF, et al. Cultural and associated enablers of, and barriers to, adverse incident reporting. *Qual Saf Health Care* 2010;19(3):229-33.

87. Heard GC, Sanderson PM, Thomas RD. Barriers to adverse event and error reporting in anesthesia. *Anesth Analg* 2012;114(3):604-14.

88. Hartnell N, MacKinnon N, Sketris I, et al. Identifying, understanding and overcoming barriers to medication error reporting in hospitals: A focus group study. *BMJ Qual Saf* 2012;21(5):361-8.

For peer review only - http://bmjopen.bmj.com/site/about/guidelines.xhtml

89. Walji R, Boon H, Barnes J, et al. Reporting natural health product related adverse drug reactions: Is it the pharmacist's responsibility? *Int J Pharm Pract* 2011;19(6):383-91.

90. Waters NF, Hall WA, Brown H, et al. Perceptions of Canadian labour and delivery nurses about incident reporting: A qualitative descriptive focus group study. *Int J Nurs Stud* 2012;49(7):811-21.

91. Espin S, Levinson W, Regehr G, et al. Error or "act of God"? A study of patients' and operating room team members' perceptions of error definition, reporting, and disclosure. *Surgery* 2006;139(1):6-14.

92. Covell CL, Ritchie JA. Nurses responses to medication errors: Suggestions for the development of organizational strategies to improve reporting. *J Nurs Care Qual* 2009;24(4): 287-97.

93. Merchant RN, Gully PM. A survey of British Columbia anesthesiologists on a provincial critical incident reporting program. *Can J Anaesth* 2005;52(7):680-4.

94. Espin S, Regehr G, Levinson W, et al. Factors Influencing Perioperative Nurses' Error Reporting Preferences. *AORN J*2007;85(3): 527-43.

95. Espin S, Wickson-Griffiths A, Wilson M, et al. To report or not to report: A descriptive study exploring ICU nurses' perceptions of error and error reporting. *Intensive Crit Care Nurs* 2010;26(1):1-9.

96. Chang IC, Hsu HM. Predicting medical staff intention to use an online reporting system with modified unified theory of acceptance and use of technology. *Telemed J E Health* 2012;18(1): 67-73.

97. Chiang HY, Lin SY, Hsu SC, et al. Factors determining hospital nurses' failures in reporting medication errors in Taiwan. *Nurs Outlook* 2010;58(1):17-25.

98. Chiang HY, Lin SY, Hsiao YC, et al. Culture influence and predictors for behavioral involvement in patient safety among hospital nurses in Taiwan. *J Nurs Care Qual* 2012;27(4):359-67.

For peer review only - http://bmjopen.bmj.com/site/about/guidelines.xhtml

99. Chiang HY, Pepper GA. Barriers to nurses' reporting of medication administration errors in Taiwan. *J Nurs Scholarsh* 2006;38(4):392-9.

100. Martowirono K, Jansma JD, van Luijk SJ, et al. Possible solutions for barriers in incident reporting by residents. *J Eval Clin Pract* 2012;18(1): 76-81.

101. Mes K, de Jong-van den Berg LTW, Van Grootheest AC. Attitudes of community pharmacists in the Netherlands towards adverse drug reaction reporting. *Int J Pharm Pract* 2002;10(4):267-72.

102. Zwart DL, Heddema WS, Vermeulen MI, et al.. Lessons learnt from incidents reported by postgraduate trainees in Dutch general practice. A prospective cohort study. *BMJ Qual Saf* 2011;20(10):857-62.

103. Eland IA, Belton KJ, van Grootheest AC, et al. Attitudinal survey of voluntary reporting of adverse drug reactions. *Br J Clin Pharmacol* 1999;48(4):623-7.

104. Alsafi E, Bahroon SA, Tamim H, et al. Physicians' attitudes toward reporting medical errors-an observational study at a general hospital in Saudi Arabia. *J Patient Saf* 2011;7(3):144-7.

105. Khan, TM. Community pharmacists' knowledge and perceptions about adverse drug reactions and barriers towards their reporting in eastern region, alahsa, Saudi Arabia. *Ther Adv Drug Saf* 2013;4(2):45-51.

106. Tobaiqy M, Stewart D. Exploring health professionals' experiences of medication errors in Saudi Arabia. *Int J Clin Pharm* 2013;35(4):542-5.

107. Bawazir SA. Attitude of community pharmacists in Saudi Arabia towards adverse drug reaction reporting. *Saudi Pharm J* 2006;14(1):75-83.

108. Davies EC, Chandler CI, Innocent SH, et al. Designing adverse event forms for real-world reporting: Participatory research in Uganda. *PLoS One* 2012;7(3):e32704.

109. Clark M, Gray M, Mooney J. New graduate occupational therapists' perceptions of near-misses and mistakes in the workplace. *Int J Health Care Qual Assur* 2013;26(6):564-76.

For peer review only - http://bmjopen.bmj.com/site/about/guidelines.xhtml

110. Kagan I, Barnoy S. Factors associated with reporting of medication errors by Israeli nurses. *J Nurs Care Qual* 2008;23(4):353-61.

111. Kagan I, Barnoy S. Organizational safety culture and medical error reporting by israeli nurses. *J Nurs Scholarsh* 2013;45(3):273-80.

112. Naveh E, Katz-Navon T, Stern Z. Readiness to report medical treatment errors: the effects of safety procedures, safety information, and priority of safety. *Med Care* 2006;44(2117-23.

113. Joolaee S, Hajibabaee F, Peyrovi H, et al. The relationship between incidence and report of medication errors and working conditions. *Int Nurs Rev* 2011 Mar;58(1):37-44.

114. Vessal G, Mardani Z, Mollai M. Knowledge, attitudes, and perceptions of pharmacists to adverse drug reaction reporting in Iran. *Pharm World Sci* 2009;31(2):183-7.

115. Okuyama A, Sasaki M, Kanda K. The relationship between incident reporting by nurses and safety management in hospitals. *Qual Manag Health Care* 2010;19(2):164-172.

116. Soleimani F. Learning from mistakes in New Zealand hospitals: what else do we need besides "no-fault"? *N Z Med J* 2006;119(1239):U2099.

117. Yong H, Kluger MT. Incident reporting in anaesthesia: A survey of practice in New Zealand. *Anaesth Intensive Care* 2003;31(5):555-9.

118. Söderberg J, Grankvist K, Brulin C, et al. Incident reporting practices in the preanalytical phase: Low reported frequencies in the primary health care setting. *Scand J Clin Lab Invest* 2009;69(7):731-5.

119. Bäckström M, Mjörndal T, Dahlqvist R, et al.. Attitudes to reporting adverse drug reactions in northern Sweden. *Eur J Clin Pharmacol* 2000;56(9-10):729-32.

120. Albolino S, Tartaglia R, Bellandi T, et al. Patient safety and incident reporting: survey of Italian healthcare workers. *Qual Saf Health Care* 2010;19 Suppl 3:i8-12.

121. Cosentino M, Leoni O, Banfi F, et al. Attitudes to adverse drug reaction reporting by medical practitioners in a Northern Italian district. *Pharmacol Res* 1997;35(2):85-8.

For peer review only - http://bmjopen.bmj.com/site/about/guidelines.xhtml

122. Rasmussen K, Pedersen AH, Pape L, et al. Work environment influences adverse events in an emergency department. *Dan Med J* 2014;61(5): A4812.

123. Ballangrud R, Hedelin B, Hall-Lord ML. Nurses' perceptions of patient safety climate in intensive care units: A cross-sectional study. *Intensive Crit Care Nurs* 2012;28(6): 344-54.

124. Mustafa GR, Rasheed S, Aziz MT. Adverse drug reaction reporting system at different hospitals of Lahore, Pakistan - An evaluation and patient outcome analysis. *J App Pharm* 2013;4(1):713-9.

125. Herdeiro MT, Figueiras A, Polónia J, et al.. Influence of Pharmacists' Attitudes on Adverse Drug Reaction Reporting. *Drug Saf* 2006;29(4): 331-40.

126. Mrayyan MT, Shishani K, Al-Faouri I. Rate, causes and reporting of medication errors in Jordan: nurses' perspectives. *J Nurs Manag* 2007;15(6):659-70.

127. Li Q, Zhang SM, Chen HT, et al. Awareness and attitudes of healthcare professionals in Wuhan, China to the reporting of adverse drug reactions. *Chin Med J (Engl)* 2004;117(6): 856-61.

128. Hasford J, Goettler M, Munter KH, et al. Physicians' knowledge and attitudes regarding the spontaneous reporting system for adverse drug reactions. *J Clin Epidemiol* 2002;55(9):945-50.

129. Irujo M, Beitia G, Bes-Rastrollo M, et al. Factors that Influence Under-Reporting of Suspected Adverse Drug Reactions among Community Pharmacists in a Spanish Region. *Drug Saf* 2007;30(11):1073-1082.

130. Nilsen P. Making sense of implementation theories, models and frameworks. *Implement Sci* 2015;10: 53.

For peer review only - http://bmjopen.bmj.com/site/about/guidelines.xhtml

For peer review only

For peer review only - http://bmjopen.bmj.com/site/about/guidelines.xhtml

Table 1: Search Strategy

Category A	Patient Safety Incident: near adj miss* (MeSH heading), adverse adj event*, never adj event* (MeSH entry term), medical adj mistake* (MeSH entry term), error*, mistake* (MeSH entry term), negligen* (MeSH entry term), malpractice* (MeSH heading), failure*, injur* (MeSH entry term), critical adj incident* (MeSH entry term), sentinel adj event*, incident*, harm*, accident* (MeSH heading), medical adj error* (MeSH heading), patient adj safety (MeSH heading)
Category B	Incident Reporting System: risk adj management (MeSH heading), incident adj reporting adj system*, error adj report*, critical adj incident adj technique (MeSH entry term), safety adj report*, incident adj report* (MeSH entry term), reporting adj system, NRLS, national adj reporting adj2 learning adj system.
Category C	Barrier/Facilitator: communication adj barrier* (MeSH heading), feedback (MeSH heading), safety adj culture (MeSH entry term), reporting adj culture, attitude (MeSH heading)*, preventive adj measure* (MeSH entry term), mandatory, voluntary, under-reporting, willingness, blame, obstacle*, incident adj type, level adj of adj harm, fear* (MeSH heading), responsibi*, workload (MeSH heading), trust* (MeSH heading), anonym*, confidential* (MeSH heading), facilit*, barrier*, enabl*, legal, law (MeSH entry term).

For peer review only - http://bmjopen.bmj.com/site/about/guidelines.xhtml

Table 2: Theoretical framework of factors determining engagement in patient safety incident reporting

Category	Descriptions & Examples
**Organisational**	Organisational values, beliefs and policies around incident reporting. This also encompasses any organisational factor which may act as a barrier or facilitator to reporting behavior, such as structure (e.g. size of hospital) and organisational culture.
**Work Environment**	Features of the work environment that act as barriers or facilitators to engagement in incident reporting. Examples of such factors include level of activity, staffing levels and visual prompts.
**Process and systems of Reporting**	Any characteristics or features of the reporting system/process which enables or hinders incident reporting. This includes the complexity of the reporting system, the level of information required and the mode of incident reporting (e.g. paper based or electronic).
**Team factors**	Any factor related to the functioning of different professionals within a group which influences incident reporting behavior. For example, support and encouragement by team members to report incidents, and levels of teamwork and communication.
**Knowledge and Skills**	The acquisition and development of knowledge and skills that enables incident reporting. This includes participation in specific (e.g. form completion) and general (e.g. identifying which incidents warrant reporting) training/educational activities.
**Individual HCP Characteristics**	Characteristics of the HCP that may contribute in some way to engagement in incident reporting. Examples of such factors include seniority, personality and attitudes.
**Professional Ethics**	The accepted standards of personal and professional behavior, values and guiding principles that promote incident reporting. For example, the adoption of sound and consistent ethical practices, such as duty of care.
**Fear of adverse consequences**	Any unpleasant emotion (e.g. guilt) or outcome (e.g. litigation) associated with individual HCPs' incident reporting behavior. A reduction in the likelihood of experiencing fear (e.g. the existence of a non-punitive policy) results in increased incident reporting participation.
**Incident Characteristics**	Characteristics of the patient safety incident which may make HCP's more or less likely to report. These include frequency of error, level of harm and the cause of error.

Note: HCP=Healthcare Professional

For peer review only

For peer review only - http://bmjopen.bmj.com/site/about/guidelines.xhtml

Table 3: Frequency of Articles by Country

Country	Count *(percentage)*
United States of America[9, 11, 28, 30-59]	33 *(30.00 %)*
United Kingdom[10, 29, 60-81]	24 *(21.82 %)*
Australia[8, 27, 82-87]	8 *(7.27%)*
Canada[88-95]	8 *(7.27 %)*
Taiwan[96-99]	4 *(3.64 %)*
Netherlands[100-103]	4 *(3.64 %)*
Saudi Arabia[104-107]	4 *(3.64 %)*
International[24, 26, 108, 109]	4 *(3.64 %)*
Israel[110-112]	3 *(2.73 %)*
Iran[113, 114]	2 *(1.82 %)*
Japan[25, 115]	2 *(1.82 %)*
New Zealand[116, 117]	2 *(1.82 %)*
Sweden[118, 119]	2 *(1.82 %)*
Italy[120, 121]	2 *(1.82 %)*
Denmark[122]	1 (0.91 *%)*
Norway[123]	1 (0.91 *%)*
Pakistan[124]	1 (0.91 *%)*
Portugal[125]	1 (0.91 *%)*
Jordan[126]	1 (0.91 *%)*
China[127]	1 (0.91 *%)*
Germany[128]	1 (0.91 *%)*
Spain[129]	1 (0.91 *%)*

For peer review only - http://bmjopen.bmj.com/site/about/guidelines.xhtml

Figure 1: Example of data coding, conceptualisation and categorisation for theory development

	**Stage 1: Coding (open coding)** *Identification of data that contributes to engagement in incident reporting*	→	**Stage 2: Conceptualising (axial coding)** *Identification of codes of similar content grouped and named*	→	**Stage 3: Categorising (selective coding)** *Identification of similar concepts grouped into broad groups*
Paper 1: "Despite some initial success with this approach, [implementation of incident reporting system] it was stated that the **time** and **resource** demands of managing the process were too great".	Not enough time<br>Not enough resources	→	Time<br>Resources	→	Process and system of reporting
Paper 2: "The concept of a **blame culture** appeared to exist".	People are scared of being blamed	→	Fear of blame	→	Fear of adverse consequences

For peer review only

Figure 2: Flow diagram of the theoretical literature review process

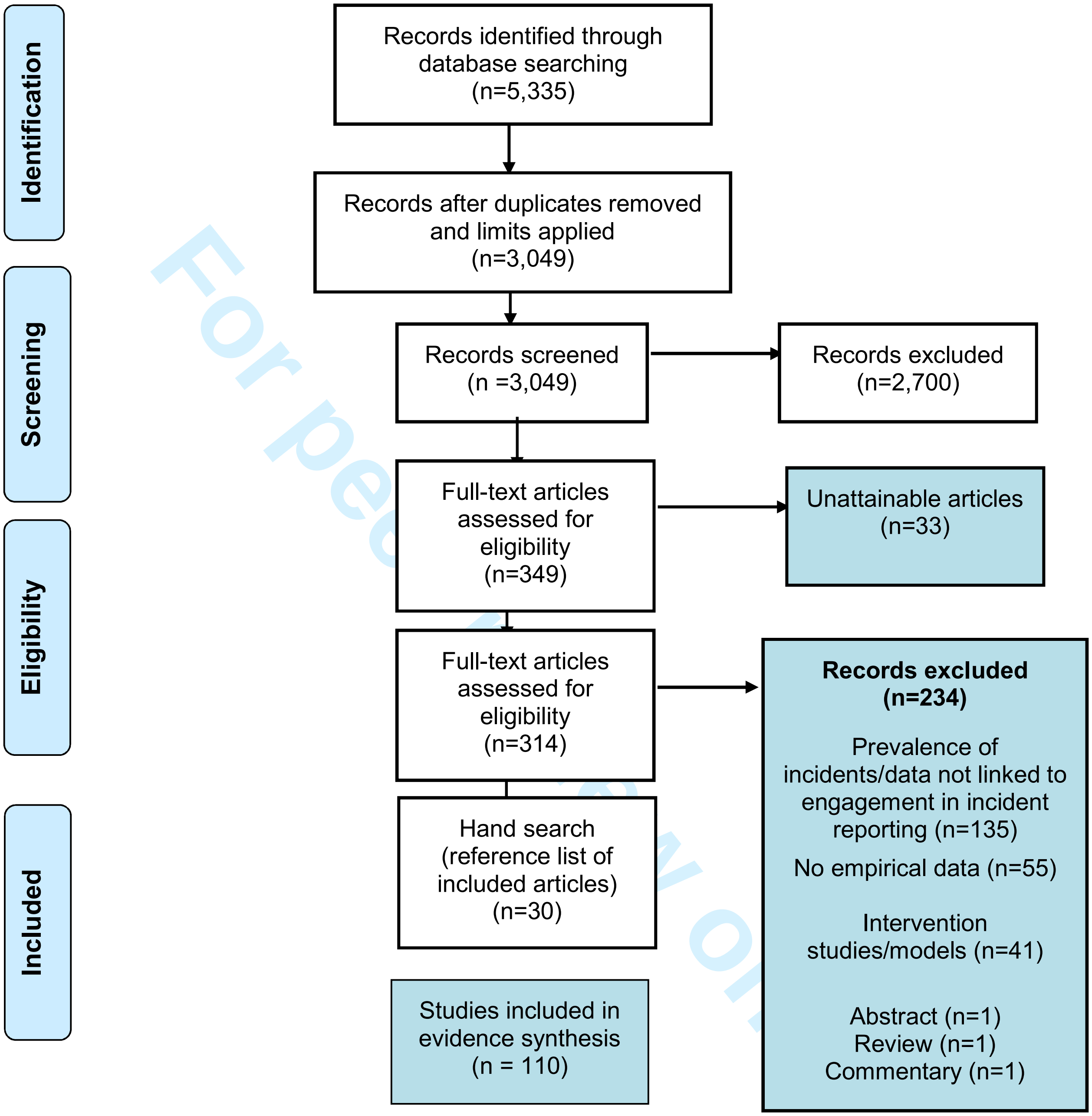

Figure 3: Frequency of categories influencing engagement in patient safety incident reporting

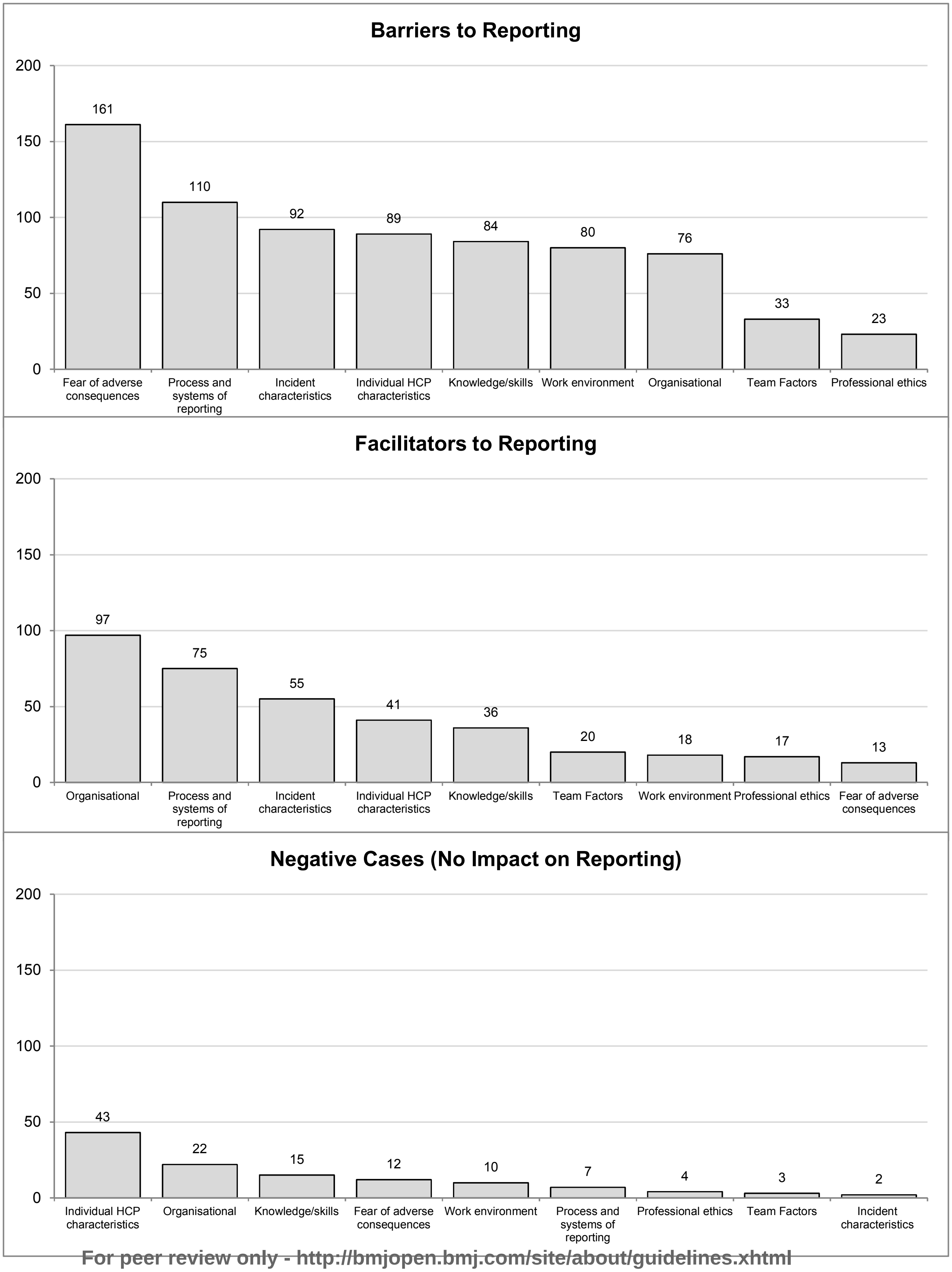

For peer review only - http://bmjopen.bmj.com/site/about/guidelines.xhtml

eTable1: Full data extraction table of included articles

Author, Year	Study Design, Sample Size, Country	Barriers to Incident Reporting	Facilitators of Incident Reporting	Negative cases (No impact)
Albolino et al., 2010 [120]	Questionnaire based-study<br>820<br>Italy	Fear of mistrust in colleagues<br><br>Not considered a priority<br><br>Fear of punishment<br><br>Does not help to improve safety<br><br>Lack of time		
Alsafi et al., 2011 [104]	Questionnaire based-study.<br>107<br>Saudi Arabia	Not my responsibility<br><br>I do not want to lose my good relationship with my colleague<br><br>I might be reported by my colleague in turn<br><br>No incentive to error disclose<br><br>Avoiding punishment<br><br>Avoiding damage to reputation<br><br>It will not be discovered		
Anderson et al., 2013 [60]	Semi-structured interviews and documentary analysis	Experienced in using IR systems (Mental health staff)		

For peer review only - http://bmjopen.bmj.com/site/about/guidelines.xhtml

	62<br>United Kingdom	Blame culture (mental health staff)		
Arfanis et al., 2012 [61]	Semi-structured interviews<br>48<br>United Kingdom	Not used as learning tools to prevent similar occurrences elsewhere.<br><br>Pressures on time<br><br>Resources<br><br>A lack of faith in the established system<br><br>Fruitless and often pointless exercise that has little or no impact on improving patient safety and welfare<br><br>Fear of litigation<br><br>Fear of disciplinary action<br><br>Blame<br><br>The availability and ease of identifying the information<br><br>No feedback	Feedback<br><br>Learning and improvement<br><br>Anonymous web based forum as an add on to IR system	
Armitage et al., 2010 [62]	Semi-structured interviews and retrospective review of error reports<br>40	Lack of feedback		

For peer review only - http://bmjopen.bmj.com/site/about/guidelines.xhtml

	United Kingdom			
Ashcroft et al., 2006 [66]	Questionnaire-based Study<br>275<br>United Kingdom	Local reporting<br><br>Good patient outcome less likely to be reported than poor or bad patient outcome.<br><br>Compliance with a protocol less likely to be reported than a violation or error.<br><br>'Fault-led' attitude<br><br>One-off situations by individuals not report<br><br>Loyalty to colleagues<br><br>National reporting system<br><br>Confidence in National Patient Safety Agency	Local reporting<br><br>Poor or bad patient outcome more likely to be reported than good patient outcome<br><br>Violation of protocol or error more likely to be reported than compliance with protocol.<br><br>'Learn from mistakes' culture<br><br>Individuals making continual mistakes<br><br>National reporting system	
Backstrom et al., 2000 [119]	Questionnaire-based study.<br>748<br>Sweden	Assessment that the reaction is already well known<br><br>Forgetting to report		

For peer review only - http://bmjopen.bmj.com/site/about/guidelines.xhtml

		Hesitance to report on suspicion<br><br>Lack of time<br><br>Giving preference to other matters<br><br>Uncertainty about the existing rules for reporting<br><br>Difficulty in finding the right form		
Ballangrud et al., 2012 [123]	Questionnaire-based study.<br>220<br>Norway	Supervisor/manager expectations, actions promoting safety<br><br>Feedback and communication about error		Organisational learning and continuous improvement<br><br>Teamwork within hospital units<br><br>Communication openness<br><br>Non punitive response to errors<br><br>Staffing
Bateman et al., 1992 [81]	Questionnaire-based study.<br>1181	One case cannot contribute to medical knowledge	Should be financially	

For peer review only - http://bmjopen.bmj.com/site/about/guidelines.xhtml

	United Kingdom	Impossible to determine responsible drug<br><br>Serious ADRs well known when the drug is marketed<br><br>Professional obligation<br><br>Reporting increases personal liability<br><br>Reporting results by badgering by Committee of safety of medicines<br><br>Takes too much time to ADR report	reimbursed<br><br>Would report if easier method	
Bawazir et al., 2006 [107]	Questionnaire-based study.<br>172<br>Saudi Arabia	No reporting forms available<br><br>Reporting address unknown<br><br>Reporting form too complicated<br><br>Reporting ADRs is too time consuming<br><br>All ADRs are known<br><br>Want to publish myself<br><br>Confidentiality<br><br>Patient confidence	An obligation to do so<br><br>There was a fee<br><br>Saw colleagues doing so<br><br>Attention drawn by publication<br><br>Receiving feedback<br><br>Report through the internet	

For peer review only - http://bmjopen.bmj.com/site/about/guidelines.xhtml

		Difficult to admit harm to patient<br><br>Reporting could show ignorance<br><br>Fear of liability<br><br>No motivation<br><br>Insufficient clinical knowledge<br><br>Do not know how to report<br><br>Causality uncertain<br><br>One report make no difference		
Beasley et al., 2004 [30]	Focus groups<br>14<br>United States of America	Punitive system	A feedback system for submitters is necessary to maintain interest.<br><br>Safe and secure access<br><br>There needs to be easy access<br><br>What to report needs to be clearly defined<br><br>The reporting forms	

For peer review only - http://bmjopen.bmj.com/site/about/guidelines.xhtml

			must be simple<br><br>Error reporting must fit into a clinicians current work flow<br><br>A non-punitive system is essential<br><br>Reporter should only be required to report once if there are multiple systems	
Belton et al., 1995 [80]	Questionnaire-based study<br>284<br>United Kingdom	Report forms are not available when needed<br><br>Doctor does not like reporting confidential information<br><br>Doctor unsure how to report an ADR<br><br>Doctor fear he/she may appear foolish about reporting a suspected reaction<br><br>Doctor fears he/she may be exposed to legal liability by reporting reaction<br><br>Doctor too busy to send an ADR		

For peer review only

For peer review only - http://bmjopen.bmj.com/site/about/guidelines.xhtml

		report<br><br>Doctor is reluctant to admit he/she may have caused a patient harm<br><br>Doctor would rather collect and publish personally<br><br>Doctor believe that only safe drugs are marketed		
Belton et al., 1997 [24]	Questionnaire-based study<br>Sample size not reported<br>International: Denmark, France, Ireland, Italy, Netherlands, Portugal, Spain, Sweden, United Kingdom	Telephone number unavailable<br><br>Report forms unavailable<br><br>Address of reporting agency unavailable<br><br>Unsure how to report<br><br>Patient confidentiality<br><br>Worried about appearing foolish<br><br>Worried about legal liability (Not Denmark or Spain)<br><br>Too busy to report ADRs<br><br>Reluctant to admit they have caused a patient harm		Worried about legal liability (Not Denmark or Spain)<br><br>Ambition to publish a personal series of cases (Not Spain, Sweden or Portugal)<br><br>Patient confidentiality (Not Spain)

For peer review only

		Ambition to publish a personal series of cases (Not Spain, Sweden or Portugal)<br><br>Believes that all marketed drugs are safe		
Blegen et al., 2004 [55]	Questionnaire-based study<br>1105<br>United States of America	Administrative response<br><br>Personal fear<br><br>Quality management<br><br>Staffing resources<br><br>Physical resources<br><br>Peer relations<br><br>Job satisfaction		
Braithwaite et al., 2010 [86]	Questionnaire-based study.<br>2185<br>Australia	IIMS training<br><br>Accessibility of reporting system<br><br>Security of IIMS<br><br>Feedback from reports<br><br>Workplace reporting culture<br><br>Value placed on IIMS		Form of training received

For peer review only - http://bmjopen.bmj.com/site/about/guidelines.xhtml

Chang et al., 2012 [96]	Questionnaire-based study<br>183<br>Taiwan		Level of support	Age
Chiang et al., 2006 [99]	Questionnaire-based study.<br>597<br>Taiwan	Being blamed for MAE results<br><br>Adverse consequences from reporting<br><br>Patient's negative attitude<br><br>Physicians' reprimand<br><br>Not recognised MAEs occurred<br><br>Being recognised as incompetent<br><br>Too much time for filling reports<br><br>Think MAEs not important enough to be reported<br><br>Too much time for contacting physicians<br><br>Unclear MAE definition<br><br>Disagreement over MAE<br><br>Unrealistic expectation for administering drugs correctly		

For peer review only

For peer review only - http://bmjopen.bmj.com/site/about/guidelines.xhtml

		No positive feedback<br><br>Much emphasis on MAE as nursing quality provided<br><br>Focus on individual rather than system factors to MAEs<br><br>Administrators' responses to MAEs do not match the severity of the errors		
Chiang et al., 2010 [97]	Questionnaire-based study<br>838<br>Taiwan	Experience of making MAEs<br><br>Nursing professional development<br><br>Fear	Same attitude towards self and co-workers<br><br>MAE reporting rate<br><br>Nursing quality	Age<br><br>Management and leadership<br><br>Administrative barriers<br><br>Reporting process
Chiang et al., 2012 [98]	Questionnaire-based study<br>1049<br>Taiwan		High scores on the safety organising scale<br><br>Tenure of present position<br><br>Self-evaluated IR rates	

For peer review only

			Those more willing to report their own incidents are more likely to report co-workers incidents	
Church et al., 2013 [36]	Questionnaire-based study<br>546<br>United States of America	Hierarchical structure<br><br>Poor communication<br><br>Fear of reprimand<br><br>Reprimand of other therapists and dosimetrists<br><br>Personality<br><br>Lack of reporting system		
Clark et al., 2013 [109]	Questionnaire-based study<br>228<br>International: Australia and New Zealand	Fear of being judged by colleagues<br><br>Personal Guilt<br><br>Feel it as unnecessary<br><br>Near misses are part of life		
Coley et al., 2006 [57]	Focus groups<br>8<br>United States of America	Time consuming<br><br>Inadequate staffing		

For peer review only - http://bmjopen.bmj.com/site/about/guidelines.xhtml

Cosentino et al., 1997 [121]	Questionnaire-based study<br>207<br>Italy	Reaction not clinically relevant<br><br>Awareness of similar reactions<br><br>Unavailability of report forms<br><br>Doubtfulness about which ADRs should be reported<br><br>Confidence about ADRs being well documented before marketing<br><br>Ignorance about reporting procedures<br><br>Too much time required to fill in the report form<br><br>Don't feel obliged to report<br><br>Don't want to create undue alarm<br><br>Uselessness of ADR spontaneous reporting		
Covell et al., 2009 [92]	Semi-structured interviews and questionnaire based study<br>50<br>Canada	Adverse consequences		
Daly et al., 2005 [37]	Questionnaire-based study<br>598	Administrators' length of time in position	Directors of nursings'	Administrators' knowledge of

For peer review only - http://bmjopen.bmj.com/site/about/guidelines.xhtml

	United States of America	Administrators' and Directors' length of time in facility<br><br>Administrators' length of time in profession<br><br>After internal investigation abuse was thought not to exist<br><br>Told not to report the abuse by my boss<br><br>Reported abuse in the past and IDIA did nothing<br><br>Reported abuse in the past and it led to a bad outcome<br><br>Reported abuse in the past and IDIA ruled it out	knowledge of the law in of nursing<br><br>Administrators' level of education	law<br><br>Administrators' belief that 'elders are able to get help if they need it'<br><br>Age of administrators and directors of nursing<br>Director of nursings' length of time in position<br><br>Director of nursings' length of time in profession<br><br>Director of nursings' level of education<br><br>Administrators' knowledge of the law in nursing
Davies et al., 2012	Focus groups	Lack of feedback		

For peer review only - http://bmjopen.bmj.com/site/about/guidelines.xhtml

[108]	19<br>International: United Kingdom/Uganda			
Ehrenpreis et al., 2012 [38]	Questionnaire-based study<br>92<br>United States of America	Unsure how to report appropriately<br><br>Did not see adverse events on a regular basis<br><br>Too busy to make reports<br><br>The existing method was too cumbersome<br><br>Voluntary reporting was not an important process	Easier to use	
Eland et al., 1999 [103]	Questionnaire-based study<br>1357<br>Netherlands	Uncertain association<br><br>Too trivial to report<br><br>Too well known to report<br><br>Unaware of the existence of a nation ADR reporting system<br><br>Unaware of the need to report ADRs<br><br>Did not know how to report ADRs<br><br>Too bureaucratic<br><br>Not enough time		

For peer review only - http://bmjopen.bmj.com/site/about/guidelines.xhtml

		Concerned that the report could be used in legal case for damages by the patient<br><br>If another physician had prescribed the medicine<br><br>Medication brought over counter rather than prescribed		
Elder et al., 2007 [31]	Focus groups<br>139<br>United States of America	Burden of effort<br><br>Lack of time<br><br>Forgetfulness<br><br>Information not readily available<br><br>Computer problems<br><br>Online access<br><br>What to report<br><br>Who should report<br><br>What is an AE<br><br>What information is needed<br><br>Common problems	Perceived benefit of reporting – learning and improvement<br><br>Emotional benefit<br><br>Guilt<br><br>Personal responsibility<br><br>Anonymous reporting<br><br>Easing the burden of reporting<br><br>The more harm, the more likely to report	

For peer review only - http://bmjopen.bmj.com/site/about/guidelines.xhtml

		Rare errors<br><br>Less serious errors unlikely to be reported<br><br>Feeling personally responsible		
Elder et al., 2008 [58]	Focus groups and questionnaire-based study<br>125<br>United States of America	Too busy with other activities<br><br>Didn't reach the patient<br><br>Risk of harm is none or little<br><br>Error made my someone new-give them a break<br><br>Feel worse emotionally<br><br>Feel like a failure<br><br>Fear punishment<br><br>Blame<br><br>Name on permanent record<br><br>Risk losing friends<br><br>Will make enemies on unit<br><br>No feedback so no personal benefits	Asked by management to make specific reports<br><br>Harm actually occurred<br><br>Risk of harm is great<br><br>Error made by someone unable to be spoken to one-to-one<br><br>Feel better emotionally<br><br>Outlet for irritation at situation or person<br><br>Honesty is a virtue	

For peer review only

			Get a "there but for the grace of god" understanding<br><br>Improve clinical practice<br><br>Could be a learning experience for others<br><br>No known penalty for making a report	
Erler et al., 2013 [39]	Questionnaire-based study<br>51<br>United States of America		Higher levels of teamwork<br><br>Communication openness<br><br>Perception of manager actions promoting safety	
Espin et al., 2010 [95]	Semi-structured interviews<br>37<br>Canada	Did not feel it was an error	Patient negligence<br><br>Threat of potential or actual harm to the patient<br><br>Patient advocacy	

For peer review only

For peer review only - http://bmjopen.bmj.com/site/about/guidelines.xhtml

			Following proper procedure<br><br>Error prevention<br><br>Learning opportunities	
Espin, et al., 2007 [94]	Semi-structured interviews<br>13<br>Canada	Domain-specific expertise is a necessary pre-requisite for reporting the error<br><br>Part of the surgeon's responsibility as it fell within the surgical scope of practice.	Events outside of professional boundaries were more likely to be reported<br><br>Responsible for error	
Espin et al., 2006 [91]	Semi-structured and structured interviews<br>28<br>Canada	Responsibility		
Evans et al., 2006[8]	Questionnaire-based study<br>773<br>Australia	I never get any feedback on what action is taken<br><br>I don't feel confident it is kept anonymous<br><br>The incident form takes too long to fill out and I just don't have time<br><br>I am worried about litigation		

For peer review only - http://bmjopen.bmj.com/site/about/guidelines.xhtml

		The incident was too trivial<br><br>When the ward is busy I forget to make a report<br><br>It's not my responsibility to report someone else's mistakes<br><br>I don't know whose responsibility it is to make a report<br><br>I don't want to get into trouble<br><br>When it is a near miss, I don't see any point in reporting it<br><br>Even if I don;t give my details, I am sure that they'll track me down<br><br>The AIMS+ form is too complicated and requires too much detail<br><br>Junior staff are often blamed unfairly for adverse incidents<br><br>I wonder about who else is privy to the information that I disclose<br><br>If I discuss the case with the person involved nothing else needs to be done		

For peer review only

For peer review only - http://bmjopen.bmj.com/site/about/guidelines.xhtml

		I don't want the case discussed in meetings<br><br>I am worried about disciplinary action<br><br>Adverse incident reporting is unlikely to lead to system changes<br><br>My co-workers may be unsupportive		
Fairbanks et al., 2008 [32]	Interviews, focus groups and events reports from an anonymous system<br>15<br>United States of America	Blame and Shame<br><br>Punishment<br><br>Legal factors<br><br>Reluctance to tell on colleagues	Non punitive system	
Fukuda et al., 2010 [25]	Questionnaire-based study<br>Sample size not stated<br>Japan		Decreased time for reporting (nurses and physicians)<br><br>Electronic reporting (physicians)<br><br>Attendance at educational seminars (physicians)<br><br>Hospital size	Non-punitive policy (physicians/nurses)<br><br>Rate of recommendations derived from reported incidents (physicians/nurses)<br><br>Electronic

For peer review only - http://bmjopen.bmj.com/site/about/guidelines.xhtml

			Ownership – university hospital (physicians)<br><br>Ownership – national hospital (nurses)<br><br>Assignment of patient safety manager (physicians)	reporting (nurses)<br><br>Attendance at educational seminars (nurses)<br><br>Elapsed years of incident reporting system (physicians and nurses)<br><br>Attendance at conference (Physicians/nurses)<br><br>Ward rounds (Physicians/nurses)<br><br>Ownership – university hospital (nurses)<br><br>Ownership – national

For peer review only

				hospital (physicians)<br><br>Ownership – municipal + public hospitals + healthcare corporation + other (physicians/nurse)<br><br>Assignment of patient safety manager (nurses)
Gaal et al., 2010 [26]	Observational study<br>Sample size not stated<br>International: Austria, Belgium, England, France, Germany, Israel, The Netherlands, Slovenia, Switzerland, and Wales		Group (>3) practice	Practice setting<br><br>Amount of responsibility<br><br>Hours of work<br><br>Physical working conditions<br><br>Single+ dual practice
Garbutt et al., 2007	Questionnaire-based study	Private practice	Belief that errors	Perceived risk

For peer review only

For peer review only - http://bmjopen.bmj.com/site/about/guidelines.xhtml

[40]	557<br>United States of America		are one of the most serious issues in healthcare<br><br>Belief that they should report serious errors<br><br>Belief that they should report minor errors<br><br>Belief that they should report near misses<br><br>System change to improve patient safety after errors reported<br><br>If error was caused by system rather than individual failures<br><br>Personal involvement in serious errors<br><br>Assurance that the information was	for personal malpractice risk<br><br>Personal involvement in an error

For peer review only - http://bmjopen.bmj.com/site/about/guidelines.xhtml

			confidential<br><br>A non-punitive reporting system<br><br>A process that takes less than 2 minutes to use<br><br>Local to the clinician's unit or department	
Generali et al., 1995 [52]	Questionnaire-based study 235<br>United States of America	Unsure drug caused reaction<br><br>Do not have forms<br><br>Do not know how<br><br>Reaction was expected<br><br>Reporting would not occur to me<br><br>Fear of legal liability<br><br>Not my responsibility<br><br>Hours worked per week (>49 or <40)	Hours worked per week (43-49 hours)<br><br>Work setting	Age<br><br>Gender<br><br>Number of years in practice
Gladstone, 1995 [67]	Questionnaires and semi-structured interviews 107<br>United Kingdom	Fear of management reaction		

For peer review only - http://bmjopen.bmj.com/site/about/guidelines.xhtml

Green et al., 1999 [76]	Structured interview<br>30<br>United Kingdom	Lack of time/too busy<br><br>Well recognised reaction<br><br>Limited time to spend with patients<br><br>Lack of motivation<br><br>More information about ADR needed<br><br>Lack of confidence in making report<br><br>Patient confidentiality<br><br>Patient suffered an ADR to a product counter prescribed by the pharmacists being interviewed	Certainty of ADR<br><br>Suspicious of a reaction<br><br>Training<br><br>Fee for reporting<br><br>Access to patient records<br><br>Feedback<br><br>More time	
Green et al., 2001 [75]	Questionnaire-based study<br>322<br>United Kingdom	Concern that a doctor gets a copy of reporting form<br><br>Lack of confidence in discussing the ADR with the prescriber<br><br>Apprehension about sending in an inappropriate report<br><br>Lack of time to fill in a report<br><br>Concern that a report will generate extra work	Reaction is of a serious nature<br><br>The reaction is unusual<br><br>The reaction is to a new product<br><br>Certainty that the reaction is a ADR<br><br>The reaction is well	

For peer review only - http://bmjopen.bmj.com/site/about/guidelines.xhtml

		The absence of a fee for reporting ADRs<br><br>Lack of time to actively look for ADRs while in clinical practice<br><br>Lack of clinical knowledge makes it difficult to decide whether or not an ADR has occurred<br><br>Don't feel the need to report well recognised reactions<br><br>Reporting cards not available when needed	recognised for a particular agent<br><br>Education/training/ study days or evenings<br><br>More time to spend on wards with patients<br><br>More feedback, reminders and increased awareness<br><br>Encouragement from managers and departments<br><br>Increased collaboration with prescribers and participation on ward round<br><br>Increased accessibility of reporting cards<br><br>Cards specifically designed for the	

For peer review only

			use of pharmacists<br><br>More publicity in journal about reporting scheme<br><br>Online access or telephone based reporting<br><br>Development of local incentives<br><br>Increased confidence in dealing with medical staff<br><br>Making reporting a professional responsibility<br><br>A fee for reporting<br><br>ADR specialist pharmacists<br><br>Increasing awareness among other professionals that pharmacists could report ADRs	

For peer review only

For peer review only - http://bmjopen.bmj.com/site/about/guidelines.xhtml

van Grootheest et al., 2002 [101]	Questionnaire-based study<br>147<br>Netherlands	Causality uncertain<br><br>Too time-consuming<br><br>No reporting forms available<br><br>Reporting address unknown<br><br>Reporting form too complicated<br><br>All adverse reactions are known<br><br>Want to publish myself<br><br>Confidentiality<br><br>Fear of liability<br><br>No motivation<br><br>Insufficient clinical knowledge<br><br>Do not know how to report	Feedback<br><br>Publications<br><br>Information about the national centre<br><br>Simplification of reporting procedure<br><br>Promoting reporting as part of professional duty<br><br>Financial compensation<br><br>More attention to ADR reporting in university curriculum<br><br>Database of national centre available on the internet<br><br>Compulsory reporting<br><br>Peer reporting	Reporting could show ignorance

Comment [L1]: This is the senior author. Should be MES et al. Needs to be moved up.

For peer review only

Haines et al., 2008 [82]	Questionnaire-based study<br>212<br>Australia	Time<br><br>If the ward is very busy<br><br>Patients' responsibility for adverse events<br><br>Cause of the incident<br><br>Other methods of documentation<br><br>Access to previous reports (non filing of incident reports in the notes)<br><br>Poor user friendliness of computer reporter systems<br><br>Made staff feel personally responsible for the form<br><br>Poor access to computers<br><br>Non reporting by role models<br><br>Absence of a definition of a fall<br><br>Blame<br><br>Absence of training	Staff believe that completing IRs improves patient safety<br><br>Staff belief that competing IRs protects against legal liability<br><br>If the patients was harmed/injured<br><br>Patient factors<br><br>Protect staff<br><br>Type of incident - preventable	

For peer review only

For peer review only - http://bmjopen.bmj.com/site/about/guidelines.xhtml

Handler et al., 2007 [35]	Focus group and questionnaire-based study<br>132<br>United States of America	Lack of readily available medication error reporting system or forms<br><br>Lack of information on how to report a medication error<br><br>Lack of feedback to the reporter or rest of facility on medication errors that have been reported<br><br>Lack of knowledge of which medication errors should be reported<br><br>Systems or forms used to report medication error are long and time consuming<br><br>Lack of knowledge of the usefulness of reporting medication errors<br><br>Lack of a consistent definition of a medication error<br><br>Lack of an anonymous medication error reporting system<br><br>Lack of recognition that a medication error has occurred<br><br>Lack of a culture of reporting medication errors		

For peer review only

		Extra time involved in documenting a medication error<br><br>Fear of disciplinary action<br><br>Fear of being blamed<br><br>Fear of liability or lawsuits<br><br>Not knowing who is responsible for reporting a medication error<br><br>Belief that it is unnecessary to report medication errors not associated with patient harm<br><br>Lack of recognition of the actual or potential harm of a medication error<br><br>Belief that reporting medication errors has little contribution to improving the quality of care<br><br>Difficulty in proving that a medication error actually occurred<br><br>Fear of losing respect of co-workers		
Hartnell et al., 2012 [88]	Focus group and semi-structured interviews<br>30<br>Canada	Extra time required to report<br><br>Extra work required to report	Improved care/improved patient safety	

For peer review only

		Cumbersome IR forms<br><br>Hesitancy about 'telling on' someone else<br><br>Fear of loss of reputation/perceived incompetence<br><br>Perceived severity of error (less severe errors are less likely to be reported)<br><br>Inability to recognise or identify medication errors<br><br>Lack of definitions or standards for reporting<br><br>Lack of belief that reporting makes a difference<br><br>lack of trust about how error reports will be used<br><br>Reporting is the responsibility of someone else<br><br>Fear of reprisal from management/administration<br><br>Fear of exposure to malpractice suits	To prevent patient from receiving wrong medication<br><br>Provides immunity/protection from legal action<br><br>Fear of censure (harsh criticism or blame)<br><br>Perceived severity of error (more severe errors are more likely to be reported because a report will be expected)<br><br>Follow rules or policies<br><br>Ensures accountability	

For peer review only - http://bmjopen.bmj.com/site/about/guidelines.xhtml

Hasford et al., 2002 [128]	Questionnaire-based study<br>588<br>Germany	ADR too well known<br><br>ADR too trivial<br><br>Uncertain causality<br><br>Reporting too bureaucratic<br><br>Lack of time<br><br>Rules of conduct unknown<br><br>Suspect that drug prescribed by colleague<br><br>Reporting process unknown<br><br>Lack of financial reimbursement<br><br>Suspect drug was self-medication<br><br>Reports considered useless<br><br>Reporting system unknown<br><br>Fear of legal liability<br><br>Non-serious adverse reaction to established drug	Serious unknown ADR to a new drug<br><br>Serious unknown ADR to an established drug<br><br>Serious known ADR to a new drug	
Heard et al., 2012 [87]	Questionnaire-based study<br>433	I am worried about litigation		Generalised de-identified

For peer review only - http://bmjopen.bmj.com/site/about/guidelines.xhtml

	Australia	I don't want to get into trouble<br><br>My colleagues may be unsupportive<br><br>I am worried about disciplinary action<br><br>I may be blamed unfairly for the event<br><br>I do not want to be discussed in meetings.<br><br>Adverse events reporting makes little contribution to quality care<br><br>I don't know whose responsibility it is to make a report<br><br>A good outcome of the case makes reporting unnecessary<br><br>I do not know which adverse events should be reported.<br><br>Even if I don't give my details I'm worried they will track me down<br><br>The forms take too long to fill in and just don't have time<br><br>When I am busy I forget to make a report		feedback about reports received from the anaesthetic community<br><br>Role models e.g. senior colleagues and department directors who openly encourage reporting<br><br>Legislated protection of information you provide from use in litigation<br><br>Ability to report anonymously<br><br>Clear guidelines about what adverse events are errors to report<br><br>Information on

For peer review only

For peer review only - http://bmjopen.bmj.com/site/about/guidelines.xhtml

		I don't feel confident that they information I provide will be kept confidential<br><br>I never get any feedback after I report an adverse event<br><br>I wonder about who else will have access to information I disclose<br><br>As long as the staff involved learn from incidents it is unnecessary to discuss them further<br><br>I would protect my self-interests ahead of the interests of the patient if I could (by hiding or denying error)<br><br>Competition with my peers could prevent me from disclosing an error<br><br>If a doctor is careful enough he or she will not make an error<br><br>It would affect my identity as a doctor to admit to an error<br><br>Other don't need to know about errors I have made<br><br>Disclosing an error, if you don't have		how confidentiality will be maintained if you supply your name<br><br>Individualised feedback to you about reports you submit<br><br>Paper forms for reporting provided in each theatre<br><br>More support from colleagues<br><br>Less blame attached to those who report errors<br><br>ANZCA continuing professional development point for

For peer review only - http://bmjopen.bmj.com/site/about/guidelines.xhtml

		to, is an optional act of heroism<br><br>I would cover up an error I had made if I could<br><br>If I admit to an error I will feel like a failure<br><br>It would affect my self-esteem to admit to an error<br><br>Doctors who make errors are humiliated my their colleagues<br><br>Medicine has a culture of silence where errors are not talked about<br><br>Doctors who make errors are blamed by their colleagues<br><br>Doctors should not make errors.		reports.<br><br>Access to computer based reporting systems for home<br><br>Education about the purpose of reporting<br><br>Computer based reporting systems<br><br>Training on how to use computer based system<br><br>Training on how to fill in papers forms for reporting<br><br>Payment for time taken to report

For peer review only - http://bmjopen.bmj.com/site/about/guidelines.xhtml

Herdeiro et al., 2006 [125]	Questionnaire-based study<br>256<br>Portugal	Lack of time<br><br>Complexity of reporting	Workplace (hospital pharmacists more likely to report than community pharmacists)<br><br>Really serious ADRs are not well documented by the time a drug is marketed'<br><br>Serious and not expected ADRs<br><br>Report an ADR if I were unsure that it was related to the use of a particular drug	Gender<br><br>Age<br><br>Job function (registered, assistant or other pharmacists)<br><br>Possible to determine if a drug is responsible for a particular adverse reaction'<br><br>Cannot contribute to pharmaceutical knowledge<br><br>Interested in articles about ADRs'<br><br>Most correct way to report ADRs in is the pharmaceutical

For peer review only - http://bmjopen.bmj.com/site/about/guidelines.xhtml

				literature<br><br>Financially reimbursement for providing the ADR service<br><br>Professional obligation to report ADRs<br><br>Reporting ADRs puts career at risk<br><br>I do not have time to complete the report card<br><br>I do not know how the information in the report card is used<br><br>I talk to pharmaceutical companies about possible ADRs with their

For peer review only

For peer review only - http://bmjopen.bmj.com/site/about/guidelines.xhtml

				drugs
Hohenhaus et al., 2008 [42]	Questionnaire-based study<br>175<br>United States of America	Afraid to report a medical error they had made<br><br>Afraid to report a medical error made by someone else<br><br>Might not report if there was no harm to the patient and the error was recognised quickly<br><br>Might not report if a physician told them not to report the error<br><br>Would not report if their supervisor told them not to	Error resulting patient harm<br><br>Error by novice nurse	
Holmstrom et al., 2012 [68]	Questionnaire-based study<br>16<br>United Kingdom	Fear of consequences<br><br>Culture of blame<br><br>Lack of training in MER for health-care professionals<br><br>Lack of time for reporting<br><br>Lack of organizational leadership and support<br><br>Lack of legal protection for individual health-care professionals who have	Provides opportunity for evaluating causes of errors (e.g. root cause analysis)<br><br>Uses a non-punitive approach to reporting<br><br>Provides feedback of results of error analysis for those involved in	Paper-based<br><br>Quick and easy to use

For peer review only - http://bmjopen.bmj.com/site/about/guidelines.xhtml

		made an error<br><br>Lack of understanding why reporting is needed<br><br>Concern that no beneficial action will follow<br><br>Non-anonymous reporting<br><br>Perceived to be bureaucratic<br><br>Lack of health-care staff<br><br>Lack of financial resources	reporting<br><br>Easy to use<br><br>Provides opportunity for error data analysis<br><br>Produces recommendations and guidelines for improving medication safety<br><br>Provides confidentiality of reported information<br><br>Provided and maintained by one national organisation<br><br>Integral part of patient safety reporting system<br><br>Reporting of errors is voluntary	

For peer review only - http://bmjopen.bmj.com/site/about/guidelines.xhtml

			Reporting of errors is mandatory<br><br>Allows all healthcare professionals to report errors<br><br>Available in electronic format<br><br>Independent reporting system dedicated for medication error reporting<br><br>Provides a choice of reporting anonymously<br><br>Includes reporting of both potential and actual errors	
Hutchinson et al., 2009 [29]	Retrospective analysis of routinely collected data and questionnaire-based study<br>Sample size not stated<br>United Kingdom		Employer treats fairly staff involved in error near miss or incident<br><br>Employer encourages staff to	Knows how to report errors, near misses and incidents<br><br>When errors are reported,

For peer review only - http://bmjopen.bmj.com/site/about/guidelines.xhtml

			report errors, near misses or incidents<br><br>Employer treats reports of errors, near misses or incidents confidentially<br><br>Employer does not blame or punish people who make errors.<br><br>Access to a counselling service were also more likely to report.<br><br>Previous reporting behaviours<br><br>Level of risk management	employer takes action to ensure that they do not happen again
Irujo et al., 2007 [129]	Case control study<br>78<br>Spain	Not serious ADR<br><br>Already well known ADR<br><br>Uncertain about causality<br><br>Forgot to report		Age<br><br>Working experience as pharmacist<br><br>Participation in

For peer review only - http://bmjopen.bmj.com/site/about/guidelines.xhtml

		Lack of time		a programme for detection and resolution of DRPs<br><br>Education on detection and resolution of DRPs<br><br>Frequently considering the possibility of finding an ADR when attending a patient with symptoms<br><br>Forgetting to report<br><br>Education for ADR reporting<br><br>Awareness of the importance of reporting system<br><br>It is necessary to be sure that the reaction is

For peer review only - http://bmjopen.bmj.com/site/about/guidelines.xhtml

				causally related to the use of a particular drug<br><br>Basic knowledge about ADR reporting
Jeffe et al., 2004 [11]	Focus groups<br>109<br>United States of America	Not knowing what to report<br><br>Errors that pose little risk to the patient<br><br>Errors that do not end up harming the patient<br><br>Not knowing how to report<br><br>Fear of disciplinary repercussions (nurse and physicians)<br><br>Fear of legal repercussions (nurse and physicians)<br><br>Fear of repercussions from doctors (nurses)<br><br>Link between reporting and performance reviews (nurses)	Severity of the situation (nurses)<br><br>Likelihood of reoccurrence (nurses)<br><br>Severe events reported as the error would be 'found' out anyway<br><br>Self-protection<br><br>The importance of reporting errors for educational purposes<br><br>Anonymous (physician and nurses)	

For peer review only

		Protecting colleagues from disciplinary action(nurses)<br><br>Lack of confidentiality<br><br>Name, blame, shame culture<br><br>Fear of public exposure<br><br>Staff shortages<br><br>Lack of time<br><br>The lack of simple procedure for reporting errors<br><br>Lack of feedback	Simple (physician and nurses)<br><br>Fast reporting procedures(physician and nurses)<br><br>Receipt of critical feedback about the errors<br><br>Anonymous, phone in system (physicians)<br><br>Educational rather than punitive system (physicians)<br><br>System that was 'lawyer proof'<br><br>Blame free reporting (nurses)	
Jennings et al., 2011 [27]	Focus groups, interviews and questionnaire based study<br>Sample size not stated<br>Australia	Burden of reporting in terms of time<br><br>Lack of accessibility of reporting forms	Clarity of indemnity from prosecution	

For peer review only - http://bmjopen.bmj.com/site/about/guidelines.xhtml

		Time elapsed following incident<br><br>Priority of reporting over other work tasks<br><br>Forgetting to report<br><br>Workload<br><br>Fear of disciplinary action<br><br>Fear of potential litigation<br><br>Fear of breaches of confidentiality/anonymity<br><br>Fear of embarrassment within peer group<br><br>Fear that incidents many impact on their likelihood of promotion<br><br>Concern that nothing would change even if the incident was reported<br><br>Lack of familiarity with process		
Johnstone et al., 2008 [84]	Focus groups, semi-structured interviews and questionnaire-based study 35	Frequency of incident-more frequent less likely to report	Seniority of graduate nurses	

For peer review only

For peer review only - http://bmjopen.bmj.com/site/about/guidelines.xhtml

	Australia			
Joolaee et al., 2011 [113]	Questionnaire-based study<br>286<br>Iran			Perceived work conditions
Kagan et al., 2008 [110]	Questionnaire-based study<br>201<br>Israel	The practice of ward nurse managers to cover up error, that is dealing with the error themselves without reporting to a higher authority	How the ward's and hospital's dealt with medication error<br><br>How their ward handles error reporting	
Kagan et al., 2013 [111]	Questionnaire-based study<br>247<br>Israel	Medical error incidence	Patient safety culture index<br><br>PSC at organisational level<br><br>PSC at departmental level<br><br>PSC at respondents personal performance level<br><br>Nurses' place of birth and their professional status (academic or non-academic	

For peer review only - http://bmjopen.bmj.com/site/about/guidelines.xhtml

			registered nurse)	
Kaldjian et al., 2009 [41]	Questionnaire-based study<br>338<br>United States of America		Feedback	
Karsh et al., 2006 [33]	Focus group<br>14<br>United States of America	Length of report<br><br>Punishment<br><br>Reporting near misses	Feedback<br><br>Mandatory system<br><br>Financial incentives<br><br>Other incentives (protection from malpractice and disciplinary action)<br><br>Support in using system<br><br>Education in using system	
Kennedy et al., 2004 [34]	Questionnaire-based study<br>113<br>United States of America	Not their responsibility to report<br><br>Never thought to report/not required to do so<br><br>Handle errors internally i.e. no corporate system<br><br>No errors worth reporting		

For peer review only

		No time to report<br><br>Forms not available or convenient		
Khan, 2013 [105]	Questionnaire-based study<br>50<br>Saudi Arabia	Unavailability of professional environment to discuss ADR<br><br>Reporting forms are not available<br><br>I do not know how to report<br><br>Reporting forms are too complicated<br><br>Reporting is time consuming<br><br>I am not motivated to report<br><br>I fear legal liability of the reported ADR<br><br>I am not confident whether it is an ADR<br><br>Insufficient knowledge of pharmacotherapy in detecting ADR<br><br>Belief that only safe drugs are marketed-not cause of reaction		
King et al., 2006 [56]	Questionnaire-based study<br>39	Time constraints		

For peer review only

For peer review only - http://bmjopen.bmj.com/site/about/guidelines.xhtml

	United States of America	Difficulty locating forms<br><br>Lack of closure/feedback<br><br>Not important<br><br>Fear of disclosure to risk management		
Kingston et al., 2004 [9]	Focus groups<br>33<br>Australia	Lack of knowledge about the reporting process and<br><br>Lack of knowledge about what constitutes an incident<br><br>"Nursing form" by association (not identified as being part of doctors role)<br><br>Time constraint<br><br>Complexity of reporting form<br><br>Lack of feedback<br><br>Lack of legal privileges afforded to the reporting process<br><br>Culture of blame<br><br>No value	Effective and efficient IRS<br><br>IRS with threat or blame<br><br>Prompt, relevant feedback<br><br>IRS that drive improvements<br><br>Monetary payment<br><br>Simplification<br><br>Less time consuming<br><br>Clear definitions of what constitutes an adverse event/near-miss	

For peer review only

For peer review only - http://bmjopen.bmj.com/site/about/guidelines.xhtml

			Evidence of value of IRS<br><br>Reporting process to be made more relevant to doctors<br><br>Reporting process less threatening by renaming the form<br><br>Increased awareness and knowledge of IR process<br><br>Protection from liability<br><br>System that doesn't require input from doctors (nurses)<br><br>Education at orientation (nurses)<br><br>Anonymous reporting	
Kreckler et al.,	Questionnaire-based study	I am too busy to fill out the form		

For peer review only

For peer review only - http://bmjopen.bmj.com/site/about/guidelines.xhtml

2009 [69]	137<br>United Kingdom	The form takes too long to complete<br><br>I am worried about litigation<br><br>I do not want the case discussed in meetings<br><br>I never get any feedback<br><br>It makes little contribution to the quality of care<br><br>I am not sure what incidents to report<br><br>The incident was too trivial<br><br>The incident did not result in any harm		
Li et al., 2004 [127]	Questionnaire-based study<br>1653<br>China	Address of reporting agency not available<br><br>Report forms unavailable<br><br>Reporting process unknown<br><br>Unaware of a national ADR reporting system<br><br>Patient confidentiality	Increasing awareness among administrators, doctors & nurses<br><br>Establishing ADR institutes<br><br>Education and training in ADR knowledge and related topics	

For peer review only - http://bmjopen.bmj.com/site/about/guidelines.xhtml

		Too busy to report ADR<br><br>ADR sufficiently well documented<br><br>Reluctant to admit that they have caused a patient harm<br><br>Worried about feeling foolish<br><br>Reluctant to admit they may have made a medical error<br><br>Personal ambition to publish a case study		
Martowirono et al., 2012 [100]	Focus group<br>22<br>Netherlands	Negatively valued<br><br>Costs time<br><br>Perceived as another administrative task that they have to complete<br><br>Priority<br><br>Do not always agree with the definition of incident<br><br>Incidents that had no major patient consequence<br><br>Incidents that have happened before and has already been reported	Reporting process-ability to report over the phone or send an email<br><br>Anonymous reporting<br><br>Provide the possibility to report without identifying the person involved<br><br>Provide feedback<br><br>Provide feedback to the reporter if an	

For peer review only - http://bmjopen.bmj.com/site/about/guidelines.xhtml

		Incidents that was not preventable<br><br>The cause of the incident Is already clear<br><br>Incidents is unlikely to happen again<br><br>Was not an incident but a complication<br><br>Incident already been discussed with the people involved<br><br>The lack of feedback on a report<br><br>Absence of visible system changes were also issues<br><br>Disloyal to colleagues<br><br>Not their responsibility<br><br>legal liability<br><br>Unpleasant working conditions<br><br>Lack of encouragement from superiors to report incidents.<br><br>Incident reporting is emotionally charged	incident on how the report will be handled<br><br>Feedback-communicate the results in terms of systems changes<br><br>Create an incident reporting culture<br><br>Create a culture in which IR is less emotionally charged e.g. by systematically discussing IR within a ward and stimulating role of supervisors<br><br>Simplify the procedure<br><br>Design a procedure in which it is possible to only report the essentials of an incident, e.g. by making a call or	

For peer review only - http://bmjopen.bmj.com/site/about/guidelines.xhtml

		Some residents stated that they did not complete IR because they did not think of it whereas others said<br><br>Did not know what to report.<br><br>Did not know how to report<br><br>IRS complicated<br><br>Workload	filing out a card or compact form with standard incidents. If necessary, the resident can be contacted for more information<br><br>Make it easy for a resident to find out if an incident has already been reported<br><br>Clarification what to report<br><br>Clarification about and how to report<br><br>Excite residents to report<br><br>Draw attention to IR e.g. putting up posters with a catchy slogan	
Mayo et al., 2004 [53]	Questionnaire-based study<br>983<br>United States of America	Afraid of manager reaction<br><br>Afraid of co-workers' reactions		

For peer review only - http://bmjopen.bmj.com/site/about/guidelines.xhtml

		Not thinking an error was serious enough<br><br>Fear of disciplinary action		
McArdle et al., 2003 [78]	Semi-structured interviews<br>15<br>United Kingdom	It takes too long<br><br>Lack of feedback received<br><br>Lack on incentive<br><br>Cumbersome<br><br>Non-anonymous<br><br>Fear of blame<br><br>Description of medication did not fall into IRS formats-scope of reporting		
Merchant et al., 2005 [93]	Questionnaire-based study<br>207<br>Canada	I think of reporting too late<br><br>Don't know where CIRS forms are<br><br>Fear of lawyers getting information<br><br>I don't know what sort of incident to report<br><br>I'm too busy<br><br>Fear of record of problem		Unnecessary as anesthesia is safe<br><br>futile as anesthesia is safe

For peer review only - http://bmjopen.bmj.com/site/about/guidelines.xhtml

		Don't have CIRS forms<br>My incidents are too minor<br>Too long<br>No value will come of this<br>Too much writing<br>Incidents I see are other's problem<br>Too many tick boxes<br>Unsure what 'critical incident' is<br>Effort is doomed to failure<br>Too difficult<br>Form is confusing<br>Unimportant to me<br>Nothing can be learned from me<br>CIRS asks wrong questions		
Mrayyan et al., 2007 [126]	Questionnaire-based study<br>779<br>Jordan	Fear of disciplinary action/lose job<br>Errors not serious to warrant		

For peer review only

		reporting<br><br>Fear of reaction from co-workers<br><br>Fear of reaction from nurse managers		
Mustafa et al., 2013 [124]	Questionnaire-based study<br>136<br>Pakistan	Uncertain association<br><br>Awareness<br><br>Concern about legal liability	Seriousness of ADRs<br><br>Unusual reaction<br><br>Reaction to a new product<br><br>Confidence in the diagnosis of ADR	
Naveh et al., 2006 [112]	Questionnaire-based study<br>632<br>Israel	Perceived safety procedures	Perceived safety information flow	Perceived priority of safety<br><br>Unit type
Okuyama et al., 2010 [115]	Questionnaire-based study<br>430<br>Japan		Safety management at ward level	Safety management at the hospital level<br><br>Attitudes of ward safety managers

For peer review only

Osborne et al., 1999 [54]	Questionnaire-based study<br>57<br>United States of America	Error not serious<br><br>Afraid of repercussions<br><br>Afraid of reactions from managers/co-workers		Perceptions of medication errors
Parvizi et al., 2014 [70]	Questionnaire-based study<br>119<br>United Kingdom	Did not know they were expected to do this<br><br>Did not know how to report to MHRA<br><br>I do not see the purpose of reporting<br><br>Lack of time<br><br>Blame<br><br>Direct reporting to the manufacturer<br><br>Not reporting if the types of device failure were considered to be common knowledge<br><br>Reporting only those that were unexpected failures or failures that may affect the patient or user<br><br>Reported by either a nurse or other doctor	Better education of the means of adverse IR<br><br>Improvements in the feedback sent to the reporter on the outcomes of the adverse incidents<br><br>Improvements in the guidance on the type of adverse device related incidents to report<br><br>Improvements in the electronic means of adverse IR<br><br>Improvements in the clinical and	

For peer review only - http://bmjopen.bmj.com/site/about/guidelines.xhtml

			adverse incidence governance	
Patrician et al., 2009 [43]	Questionnaire-based study<br>43<br>United States of America	Perceptions that the administration focuses on the individual and not the system<br><br>Nurses are blamed when something bad happened to patients<br><br>Fear adverse consequences for reporting errors<br><br>Nurses believe that their peers will think them incompetent<br><br>Nurses do not think the error was important enough to report<br><br>Fear of administrative response<br><br>Disagreement over error<br><br>Reporting effort<br>Lack of agreement about definition of error<br><br>Lack of error recognition<br>Excessive length of time for contacting physician		
Rasmussen et al.,	Questionnaire-based study		Safety climate	

For peer review only

For peer review only - http://bmjopen.bmj.com/site/about/guidelines.xhtml

2014 [122]	124<br>Denmark		Team climate<br><br>Inter-departmental working relationships<br><br>Increased cognitive demands	
Rogers et al., 1988 [51]	Questionnaire-based study<br>1121<br>United States of America	Reporting forms not available<br><br>Event already documented<br><br>Did not get to it/got busy<br><br>Did not believe it was important<br><br>Forms were too much trouble<br><br>Minor or expected side effect<br><br>Did not like interacting with the government<br><br>Liability concerns<br><br>Did not know how to report<br><br>Undetermined as ADE<br><br>Not primary physician		Age<br><br>Time in direct patient care

For peer review only - http://bmjopen.bmj.com/site/about/guidelines.xhtml

Rowin et al., 2008 [28]	Descriptive study<br>Sample size not stated<br>United States of America		More likely to report no harm (nurses)<br><br>More likely to report permanent harm, near death, death and unsafe environment (doctors)<br><br>Type of incident: falls and medication (nurse)<br><br>Type of incident: adverse clinical event (doctors)	Temporary harm<br><br>Near miss
Sanghera et al., 2007 [79]	Semi-structured interviews<br>13<br>United Kingdom	Not being aware that an error had occurred<br><br>Detailed paperwork<br><br>Time constraints<br><br>Not understanding incident reporting process<br><br>No benefit (perception that nothing is done with the data)		

For peer review only

		No encouragement by management<br><br>Fear of loss of professional registration<br><br>Fear of being in trouble<br><br>Fear of looking incompetent<br><br>Feeling upset<br><br>Fear will be blamed<br><br>Not wanting to report colleagues' errors		
Sarvadikar et al., 2010 [71]	Questionnaire-based study<br>56<br>United Kingdom		Doctors more likely to report errors with worsening patient outcome	Nurses and pharmacists likely to report error regardless of patient outcome
Schectman et al., 2006 [44]	Questionnaire-based study<br>120<br>United States of America	Unsure of reporting mechanism<br><br>No actual harm came to the patient<br><br>Reporting too difficult and time consuming<br><br>Unsure of what is considered AE/NM	Allow electronic reporting of adverse events and near misses<br><br>Clarify reporting mechanism	

For peer review only - http://bmjopen.bmj.com/site/about/guidelines.xhtml

		Inadequate MD participation in scheme<br><br>Concern about consequences of reporting others' error<br><br>Reporting makes no difference (nothing will change)<br><br>Concern about being blamed or judged less competent<br><br>Weaknesses in the reporting system<br><br>Professional behaviours<br><br>Fear of retribution<br><br>Lack of feedback and the perception that change would not result from reports.	Clarify what constitutes an AE/NM<br><br>Allow anonymous reporting<br><br>Increase physician involvement in QI<br><br>Provide feedback on QI projects arising from reports<br><br>Provide individual feedback following report<br><br>Provide summary feedback on a regular basis<br><br>Make reporting mandatory	
Schulmeister et al., 1999 [45]	Questionnaire-based study<br>160<br>United States of America	Minor error<br><br>Fear of disciplinary action		
Sharma et al., 2008 [74]	Questionnaire-based study<br>81<br>United Kingdom	Does not achieve anything<br><br>Not in physicians culture	Anonymous system<br><br>Easily accessible	

For peer review only - http://bmjopen.bmj.com/site/about/guidelines.xhtml

		Do not wish to incriminate others<br><br>Do not know how to access forms<br><br>Not bothered<br><br>Do not wish to ask nurse staff<br><br>Lack of time<br><br>Do not know which incidents need to be reported<br><br>Lack of anonymity<br><br>Not in habit of considering it<br><br>Discouraged by senior nurses	forms<br><br>Forms not held by nursing staff	
Soberberg et al., 2009 [118]	Questionnaire-based study<br>317<br>Sweden	I did not have enough time<br><br>I am concerned about possible consequences<br><br>Someone else did it<br><br>It is too complicated<br><br>No one else files incident reports<br><br>It would not make any difference		

For peer review only - http://bmjopen.bmj.com/site/about/guidelines.xhtml

		Insufficient routines for reporting		
Soleimani., 2006 [116]	Questionnaire-based study<br>128<br>New Zealand	Threat of public outcry<br><br>Professional consequences/discipline<br><br>Embarrassment in front of colleagues		
Stratton et al., 2004 [59]	Questionnaire-based study<br>284<br>United States of America	No positive feedback is given for passing medications correctly<br><br>Nurse administration focuses on the person rather than looking at the system<br><br>Too much emphasis is placed on medication errors as a measure of the quality of care<br><br>Responses by nursing administration do not match the severity of the error<br><br>Individual/personal reasons<br>Nurses could be blamed if something happened to the patient<br><br>Nurse believe other nurses will think they are incompetent		

For peer review only

		Nurses fear adverse consequences from reporting<br><br>Patient might develop a negative attitude<br><br>Nurses fear reprimand from physician<br><br>Nurses fear losing their license<br><br>Nurses want to avoid potential publicity of medication errors in the media		
Sweis et al., 2000 [77]	Questionnaire-based study<br>280<br>United Kingdom	Busy<br><br>Legal liability<br><br>Fear of breaching patient confidentiality	Serious ADR rather than trivial<br><br>Rarely occurring ADR rather than common ADR<br><br>Confidence in recognising an ADR<br><br>ADR to an established drug rather than new drug<br><br>Active support of	Training in reporting<br><br>Gender<br><br>Type of hospital<br><br>Age

For peer review only

			medical/pharmacy staff<br><br>Written hospital policy for pharmacist ADR reporting<br><br>Training and ADR meeting<br><br>Increasing seniority<br><br>Allocation of time for ADR monitoring<br><br>Publicity and promotion by hospital and CSM<br><br>Better cooperation with clinicians<br><br>Support and encouragement by the pharmacy department<br><br>More ward rounds and direct patient contact	

For peer review only - http://bmjopen.bmj.com/site/about/guidelines.xhtml

			Simplify reporting system<br><br>ADR reporting team<br><br>Feedback	
Tariq et al., 2012 [83]	Semi structured interviews<br>23<br>Australia	Lack of time		
Taylor et al., 2004 [46]	Questionnaire-based study<br>140<br>United States of America	Not important to report error that did not harm patient<br><br>Reporting errors does not make any difference<br><br>Unsure about what is considered medical<br><br>Incident report form too complicated<br><br>Concerned about being blamed or judged incompetent<br><br>Concerned about implicating others<br><br>Unsure whose responsibility it is to report errors	Make reporting of errors mandatory<br><br>Different format for IR<br><br>Use of electronic format for reports<br><br>Reward for reporting medical errors<br><br>Better education about what is considered a medical error that should be reported<br><br>Evidence that reporting of errors	

For peer review only

For peer review only - http://bmjopen.bmj.com/site/about/guidelines.xhtml

			led to system changes<br><br>Feedback on regular basis and frequencies of reported errors<br><br>Feedback regarding outcome of a specific error that has been reported	
Throckmorton et al., 2007 [47]	Questionnaire-based study<br>435<br>United States of America	Level of harm: no harm	Level of harm<br><br>Working closely to the patient<br><br>Higher scores on the Wakefield's scale<br><br>Fewer years since initial license	
Tobaiqy et al., 2013 [106]	Questionnaire-based study<br>61<br>Saudi Arabia	Lack of awareness<br><br>Workload/time constraints<br><br>Unavailability of reporting form	Continuing education events<br><br>An internet/web based reporting facility	

For peer review only - http://bmjopen.bmj.com/site/about/guidelines.xhtml

		Reporting system complexity<br><br>Error too trivial<br><br>Lack of anonymity<br><br>Fear of blame<br><br>Concerns over penalisation<br><br>Difficulty in recognising errors<br><br>Senior staff advised not to report<br><br>Lack of feedback from authority	Training focused on error prevention<br><br>Anonymity of reporting<br><br>A non-punitive reporting culture<br><br>Financial incentives linked to reporting	
Turner et al., (2013) [63]	Semi-structured interviews<br>32<br>United Kingdom	Value-not convinced that the reporting system would deliver improvements in clinical care		
Uribe et al., 2002 [48]	Questionnaire-based study<br>122 United States of America	Time involved in documenting an error<br><br>Extra work involved in reporting<br><br>Hesitancy regarding 'telling' on somebody else<br><br>Thinking that it is unnecessary to report error because it had no negative outcome		Thinking that reporting has little contribution for improvement of quality care<br><br>Not knowing the usefulness of the report<br><br>Lack of

For peer review only

		Not being able to report anonymously<br><br>Fear of lawsuits		knowledge of what should be reported<br><br>Lack of recognition that a medical error has occurred<br><br>Fear of being blamed<br><br>Fear of disciplinary action/ losing job<br><br>Lack of information in how to report<br><br>Lack of interest or motivation for reporting<br><br>Forms or computer locations not available to report medical errors

For peer review only - http://bmjopen.bmj.com/site/about/guidelines.xhtml

				Not knowing who is responsible for reporting error
Vessal et al., 2009 [114]	Questionnaire-based study<br>110<br>Iran	Uncertain association<br><br>Too trivial to report<br><br>Too well known to report<br><br>Yellow card not available<br><br>Not enough information from the patient<br><br>Not enough time<br><br>Unaware of the existence of a national ADR reporting system<br><br>Too bureaucratic<br><br>Did not know how to report<br><br>Fear of legal liability<br><br>Unaware of the need to report and ADR	The reaction is of a serious nature<br><br>The reaction is unusual<br><br>The reaction is to a new product<br><br>Reaction not reported before for a particular drug<br><br>Reaction is well recognised for a particular drug<br><br>Any reaction	
Vincent et al., 1998 [72]	Questionnaire-based study<br>198	Unnecessary		Unsupported colleagues

For peer review only - http://bmjopen.bmj.com/site/about/guidelines.xhtml

	United Kingdom	Increased workload<br><br>Blame<br><br>Worry litigation<br><br>Busy/forgot		Not knowing which incidents to report<br><br>As long as staff learn from incident it is unnecessary to discuss/report<br><br>Fear disciplinary<br><br>Not wanting incident to be discussed<br><br>Who's responsibility<br><br>Little contribution
Vogus et al., 2007 [49]	Questionnaire-based study<br>1033<br>United States of America	Safety organising<br><br>Unit type (emergency)<br><br>Safety organising and trust<br><br>Safety organising and pathways	Trust in managers<br><br>RN experience<br><br>Unit type (IC)<br><br>Number of beds	Care pathways<br><br>% of RNs with BSN<br><br>Unit type (surgery)

For peer review only

		Patient-to-RN ratio		
Walji et al., 2011 [89]	Semi- structured interviews<br>12<br>Canada	Lack of knowledge about natural health products<br><br>Lack of time/priorities<br><br>Complexity of reporting process	Pharmacists who saw themselves as 'knowledge generators' rather than just 'knowledge users' were more likely to report and less likely to allow workplace challenges to prevent their taking an extra step	
Walker et al., 1998 [85]	Focus groups and questionnaire-based study<br>43<br>Australia	Minor incidents (documentation and minor variation from the prescription)<br><br>Negative past experience of reporting<br><br>Fear of getting into trouble<br><br>Fear they will somehow stand out from the crowd in the eyes of those in authority<br><br>Feelings of discomfort or uncertainty about being required to report an incident that involved a colleague	More likely to report an incident if patient safety compromised<br><br>Capacity to feedback and improve the situation<br><br>Reporting might help raise people's awareness of problems that could be occurring	Fear of possible punishment senior staff

For peer review only

		This is more difficult if the colleague is a more experienced nurse<br><br>Others expressed with view that they wouldn't report a friend, perhaps perceiving that the friend would be in trouble if the incident was reported<br><br>Did not always want to admit their mistake<br><br>Might not even realise that an error had occurred<br><br>Incident might be highly incriminating<br><br>If the patient actually came to harm as a result of the error<br><br>If the departure from the prescribed therapy seemed reasonable<br><br>If the problem could be sorted out<br><br>Concern about the time taken to fill in the incident report form<br><br>Inadequate understanding of what constituted an error<br><br>A lack of feedback on the number of medication errors was a problem	Wrong drug<br><br>Wrong route<br><br>Wrong person<br><br>Wrong dose<br><br>Harm to the patient<br><br>A desire to target an individual or professional group to improve practice<br><br>Legal obligation of the nurse to report	

For peer review only

For peer review only - http://bmjopen.bmj.com/site/about/guidelines.xhtml

		Perceived inaction on reported errors incidents		
Waring, 2004 [64]	Semi- structured interviews<br>37<br>United Kingdom	Acute medicine and rehab: IR system was regarded as nurse led, dealing with ward issues and the work of non-medical groups<br><br>Anaesthesia: Physicians remained sceptical about the hospital wide reporting system and were generally disinclined to participate in this approach		
Waring, 2005 [10]	Semi-structured interviews<br>28<br>United Kingdom	Fear of blame<br><br>Blame culture<br><br>Peer of punishment<br><br>Fear of blame from pubic<br><br>Fear of litigation<br><br>Fear of professional competence being questioned<br><br>Fear of poor references<br><br>Reprimands from a senior colleague		

For peer review only

		Fear of use of reports-could be used at a later date in the event in medico-legal disputes		
Waters et al., 2012 [90]	Focus groups<br>16<br>Canada	Time<br><br>Fatigue<br><br>High workload<br><br>Relevance of reporting form<br><br>Complexity of reporting-gathering many pieces of information.<br><br>Unit culture<br><br>Fear of blame<br><br>Close knit team<br><br>Other methods of reporting-verbal reporting and team debrief<br><br>Lack of feedback	Previous experience of litigation<br><br>Protection against future litigation<br><br>Professional responsibility<br><br>IR perceived as learning opportunity<br><br>Desire for practice improvement	Risk of litigation
Weissman et al., 2005 [50]	Questionnaire-based study<br>203<br>United States of America	Mandatory<br><br>Non-confidential system<br><br>State run	Serious harm	

For peer review only - http://bmjopen.bmj.com/site/about/guidelines.xhtml

		Less harm		
Williams et al., 2013 [65]	Focus groups<br>17<br>United Kingdom	Severity (more likely to report if serious harm	Simpler reporting system<br><br>Targeted report<br><br>Feedback<br><br>Drug-specific error reporting forms<br><br>Electronic forms/systems (easier than paper)<br><br>Anonymous reporting	
Winchester et al., 2012 [73]	Questionnaire-based study<br>120<br>United Kingdom	Concerned about confidentiality<br><br>Did not know the procedure for reporting<br><br>Did not think anything could be done<br><br>Did not feel incident was important enough to report<br><br>Believed source to be low risk<br><br>Reporting was inconvenient	Education<br><br>Adverts/posters<br><br>Training<br><br>Compulsory reporting<br><br>Simple reporting system<br><br>An electronic	

For peer review only - http://bmjopen.bmj.com/site/about/guidelines.xhtml

			reporting system	
Yong et al., 2003 [117]	Questionnaire-based study<br>136<br>New Zealand	Time constraints<br><br>Laziness and forgetfulness<br><br>Dislike form filling<br><br>A lot of work for little practical benefit<br><br>Forms too complicated<br><br>Do not believe the system is working<br><br>Many incidents not worth reporting<br><br>Many other tools exist for correcting errors and improving standards<br><br>Dislike the published interpretation of results with diagnostic views by some anaesthetists<br><br>Qualitative result not acceptable<br><br>Feel that the main benefit of IR is local analysis and that very rare events distilled by multi-site monitoring are less important<br><br>Difficulty defining what constitutes incident	Total anonymity and confidentiality<br><br>Protection against punitive action<br><br>Simplify forms and bring up to date<br><br>Easy access to forms<br><br>Electronic data entry<br><br>Incorporating IR form filling at regular M&M meetings<br><br>Mandatory<br><br>Local analysis rather than Australasian wide<br><br>More aggressive follow up and reviewing	

For peer review only - http://bmjopen.bmj.com/site/about/guidelines.xhtml

		Inadequate feedback<br><br>Medico-legal implications<br><br>Forms not available/hard to locate<br><br>Lack of appropriate culture within department<br><br>Not accepted as part of private practice culture<br><br>Use of local IR system, hospital based audit<br><br>Incidents are discussed at department level confidentially	Publication of problems<br><br>Aims and purpose should be clarified explicitly<br><br>Select a few incidents to monitor frequency	
Zwart et al., 2011 [102]	Prospective cohort study<br>66<br>Netherlands		Expertise	Communicator<br><br>Collaborator<br><br>Manager<br><br>Health advocate<br><br>Scientist<br><br>Professional

For peer review only - http://bmjopen.bmj.com/site/about/guidelines.xhtml

Adverse Drug Event (ADE); Adverse Drug Reaction (ADR); Adverse Event (AE); Australia and New Zealand College of Anesthetists (ANZCA); Bachelor of Science in Nursing (BSN); Critical Incident Reporting Service (CIRS); Drug related problems (DRP); Incident Reporting (IR); Iowa Department of Inspections Appeals (IDIA); Incident Information Management System (IIMS); Intensive Care (IC); Medication Administration Error (MAE); Medication and Healthcare Products Regulatory Agency (MHRA); Medical Doctor (MD); Morbidity and Mortality (M&M); Near Miss (NM); Patient Safety Culture (PSC); Quality Improvement (QI); Register Nurse (RN)

For peer review only

For peer review only - http://bmjopen.bmj.com/site/about/guidelines.xhtml

eTable 2: Frequency of factors influencing engagement in incident reporting

		Impact on Reporting Engagement		
**Factor**		**Barrier** Frequency Count *(%)*	**Facilitator** Frequency Count *(%)*	**Negative Case (no impact)** Frequency Count *(%)*
Fear of Adverse Consequences	Adverse consequences	51 *(31.68%)* [8, 10, 11, 27, 30, 32, 33, 35-37, 42-45, 53-56, 58, 59, 61, 68, 75, 78, 79, 85, 87, 88, 92, 97, 99, 100, 104, 106, 109, 118, 120, 121]	-	3 *(25.00%)* [72, 85, 96]
	Litigation	30 *(18.63%)* [8-11, 24, 27, 32, 35, 48, 51, 52, 61, 69, 72, 77, 80, 81, 85, 87, 88, 93, 100, 101, 103, 105, 107, 114, 117, 124, 128]	8 *(61.54%)* [9, 11, 27, 33, 82, 88, 90]	4 *(33.33%)* [24, 40, 48, 90]
	Blame	24 *(14.91%)* [8, 10, 32, 35, 43, 44, 46, 58-61, 68, 70, 72, 78, 79, 82, 87, 90, 92, 99, 106]	4 *(30.77%)* [9, 11, 87, 88]	1 *(8.33%)* [48]
	Judgment	22 *(13.66%)* [10, 24, 35, 43, 53, 59, 67, 79, 80, 88, 92, 99, 104, 107, 109, 116, 126]		1 *(8.33%)* [101]
	Relationships	12 *(7.45%)* [10, 11, 36, 44, 46, 48, 54, 59, 92, 104, 116, 120]	-	-
	Impact on career	10 *(6.21%)* [10, 11, 27, 58, 59, 79, 86, 92, 93, 126]	-	1 *(8.33%)* [125]
	Protection of self	7 *(4.35%)* [24, 76, 80, 107, 122, 127]	-	-
	Avoid discussion in meetings	4 *(2.48%)* [8, 69, 87, 117]	-	1 *(8.33%)* [72]
	Apprehension about sending inappropriate form	1 *(0.62%)* [75]	-	-
	Non-punitive	-	1 *(7.69%)* [117]	1 *(8.33%)* [123]
	**Total**	**161** ***(100%)***	**13** ***(100%)***	**12** ***(100%)***
Process and Systems of Reporting	Time	29 *(26.36%)* [8, 11, 27, 38, 43, 48, 57, 69, 74, 78, 79, 81, 85, 87, 88, 90, 92, 93, 99-101, 105-107, 114, 118, 121]	5 *(6.67%)* [9, 11, 25, 40]	-
	Complexity/simplification of reporting	28 *(25.45%)* [8, 9, 11, 31, 33, 35, 38, 44, 46,	15 *(20.00%)* [9, 11, 30, 38, 65, 68, 73,	1 *(14.29%)* [68]

For peer review only - http://bmjopen.bmj.com/site/about/guidelines.xhtml

		51, 73, 78, 79, 88-90, 93, 100, 101, 105-107, 117, 118, 125]	77, 81, 100, 101, 117]	
	Anonymity and/or confidentiality	22 *(20.00%)* [8, 11, 24, 27, 35, 48, 50, 68, 73, 74, 76-78, 80, 87, 101, 106, 107, 127]	16 *(21.33%)* [9, 11, 29, 31, 40, 44, 65, 68, 74, 87, 100, 106, 117]	1 *(14.29%)* [18]
	Reporting format	10 *(9.09%)* [31, 44, 82, 85, 90, 93, 100, 117]	21 *(28.00%)* [9, 11, 25, 30, 44, 46, 58, 61, 65, 68, 70, 75, 87, 100, 106, 107, 117]	3 *(42.86%)* [24]
	Type of reporting system	5 *(4.55%)* [38, 50, 92, 117]	11 *(14.67%)* [33, 34, 40, 44, 68, 73, 101, 117]	-
	Unknown destination of report	4 *(3.64%)* [24, 70, 101, 107]	-	-
	Not enough information to complete report	3 *(2.73%)*[94, 107, 114]	1 *(1.33%)* [76]	-
	Sharing/access of reports	3 *(2.73%)* [51, 75, 87]	-	-
	Insufficient routines for reporting	1 *(0.91%)* [118]	-	-
	Lack of reporting system	1 *(0.91%)* [36]	-	-
	Administrative task	1 *(0.91%)* [100]	-	1 *(14.29%)* [97]
	Relevant to different HCPs	1 *(0.91%)* [64]	2 *(2.67%)* [9, 75]	-
	Reporting focus	1 *(0.91%)* [78]	2 *(2.67%)* [68]	-
	Information not readily available	1 *(0.91%)* [31]	-	-
	Not specified	-	-	1 *(14.29%)* [97]
	When/where to report	-	1 *(1.33%)* [117]	-
	Doesn't require input from doctors	-	1 *(1.33%)* [9]	-
	**Total**	**110** ***(100%)***	**75** ***(100%)***	**7** ***(100%)***
Incident Characteristics	Level of harm	40 *(43.48%)* [8, 11, 24, 31, 35, 42-48, 50, 51, 53, 54, 58, 65, 66, 69, 70, 72, 73, 80, 85, 87, 88, 92, 100, 103, 105, 106, 109, 114, 126, 128, 129]	26 *(47.27%)* [11, 31, 40, 42, 47, 50, 58, 66, 75, 77, 82, 85, 88, 95, 114, 121, 124, 125, 128]	-
	Cause of incident	19 *(20.65%)* [35, 52, 66, 81, 82, 85, 100, 101, 103, 107, 114, 119, 124, 128, 129]	6 *(10.91%)* [40, 66, 76, 77, 125]	2 *(100%)* [125, 129]
	Frequency of incident	18 *(19.57%)* [31, 51, 66, 70, 75, 76, 84, 100, 101, 103, 114, 119, 121, 127-129]	13 *(23.64%)* [11, 66, 75, 77, 114, 121, 124]	-
	Type of incident	13 *(14.13%)* [8, 33,	8 *(14.55%)* [82,	-

85

For peer review only

		34, 52, 69, 81, 85, 92, 93, 100, 107, 117, 121]	85, 121]	
	Level of risk	2 *(2.17%)* [11, 58]	1 *(1.82%)* [58]	-
	Patient characteristics	-	1 *(1.82%)* [82]	-
	**Total**	**92** ***(100%)***	**55** ***(100%)***	**2** ***(100%)***
Individual HCP Characteristics	Value/attitude towards reporting	53 *(59.55%)* [8, 9, 35, 44, 46, 56, 61, 63, 64, 66, 68, 70, 73, 74, 76, 79, 81, 86-88, 92, 93, 99-101, 103, 105, 107, 109, 117, 118, 120, 121, 128]	21 *(51.22%)* [9, 11, 40, 58, 68, 82, 88, 90, 93, 95, 97, 98, 107, 111, 125]	12 *(27.91%)* [37, 48, 54, 72, 79, 96, 129]
	Forgetfulness	9 *(10.11%)* [8, 27, 31, 72, 87, 93, 117, 119, 129]	-	1 *(2.33%)* [129]
	Perception of self	9 *(10.11%)* [24, 36, 55, 80, 87, 107, 127]	2 *(4.88%)* [89, 102]	6 *(13.95%)* [24, 102]
	Emotional response	6 *(6.74%)* [24, 36, 55, 80, 87, 107, 127]	5 *(12.20%)* [31, 58, 100]	-
	Previous reporting behaviors	5 *(5.62%)* [34, 37, 52, 60, 74]	1 *(2.44%)* [29]	1 *(2.33%)* [129]
	Exposure to errors	2 *(2.25%)* [38, 97]	1 *(2.44%)* [90]	-
	Length of time in employment	2 *(2.25%)* [37]	-	1 *(2.33%)* [37]
	Seniority	1 *(1.12%)*[37]	3 *(7.32%)* [49, 77, 84]	4 *(9.30%)* [37, 52, 125, 129]
	Data required for own purposes	1 *(1.12%)*[101]	-	-
	Work hours	1 *(1.12%)*[52]	1 *(2.44%)* [52]	1 *(2.33%)* [26]
	Demographics	-	2 *(4.88%)* [37, 98]	12 *(27.91%)* [37, 49, 51, 52, 77, 96, 97, 125, 129]
	Profession	-	5 *(12.20%)* [28, 71]	5 *(11.63%)* [28, 71, 102]
	**Total**	**89** ***(100%)***	**41** ***(100%)***	**43** ***(100%)***
Knowledge and Skills	Clarify reporting mechanism	36 *(42.86%)*[9, 11, 24, 27, 31, 35, 38, 44, 46, 51, 52, 70, 73, 76, 79, 80, 87, 88, 100, 101, 103, 105, 107, 114, 119, 121, 127, 128]	2 *(5.56%)* [44, 100]	5 *(33.33%)* [29, 48, 72, 129]
	Adverse event/near miss clarity	31 *(36.90%)*[9, 11, 31, 35, 43, 44, 46, 51, 69, 74, 82, 85, 87, 88, 92, 93, 95, 99, 100, 105, 117, 121]	7 *(19.44%)* [9, 30, 44, 46, 70, 87, 100]	2 *(13.33%)* [48, 72]
	Ability in error recognition	7 *(8.33%)* [35, 75, 79, 92, 99, 106, 124]	4 *(11.11%)* [75-77, 124]	1 *(6.67%)* [48]
	Training	5 *(5.95%)* [68, 76, 82,	21 *(58.33%)* [9,	7 *(46.67%)* [25, 77,

For peer review only

For peer review only - http://bmjopen.bmj.com/site/about/guidelines.xhtml

		86, 97]	25, 33, 70, 73, 75, 76, 87, 101, 106, 117, 127]	86, 129]
	Awareness	4 *(4.76%)* [35, 43, 106, 114]	2 *(5.56%)* [75, 85]	-
	Not enough information about product being reported	1 *(1.19%)* [89]	-	-
	**Total**	**84** ***(100%)***	**36** ***(100%)***	**15** ***(100%)***
Work Environment	Workload/priority	50 *(62.50%)* [9, 11, 24, 27, 31, 34, 35, 43, 48, 49, 51, 55-58, 61, 68-70, 72, 75-77, 80, 82, 83, 88-90, 92, 93, 100, 103, 117, 119, 120, 125, 127-129]	6 *(33.33%)* [31, 75-77, 122]	3 *(30.00%)* [51, 123, 125]
	Accessibility	27 *(33.75%)* [24, 27, 31, 34, 35, 51, 52, 56, 74, 75, 80, 82, 86, 93, 101, 105-107, 114, 117, 119, 121, 127]	11 *(61.11%)* [30, 68, 73-75, 87, 100, 101, 117]	1 *(10.00%)* [48]
	Not specified	2 *(2.50%)* [61, 105]	-	-
	Unit type	1 *(1.25%)* [49]	1 *(5.56%)* [49]	3 *(30.00%)* [49, 112]
	Physical working conditions	-	-	1 *(10.00%)* [26]
	Satisfaction with work environment	-	-	1 *(10.00%)* [113]
	Care pathways	-	-	1 *(10.00%)* [49]
	**Total**	**80** ***(100%)***	**18** ***(100%)***	**10** ***(100%)***
Organization	Feedback/communication	26 *(34.21%)* [8, 9, 11, 35, 37, 43, 44, 56, 58, 59, 61, 62, 69, 78, 85-87, 90, 92, 99, 100, 106, 108, 117, 123]	29 *(29.90%)* [9, 11, 30, 33, 41, 44, 46, 61, 65, 68, 70, 75-77, 87, 100, 101, 107, 112, 117]	2 *(9.09%)* [25, 125]
	Reporting culture	17 *(22.37%)* [9, 10, 34, 35, 49, 66, 70, 81, 86, 90, 92, 114, 117, 118, 123]	16 *(16.49%)* [29, 33, 39, 66, 75, 96, 100, 106, 110-112, 121, 122]	1 *(4.54%)* [96]
	Learning/improvement	7 *(9.21%)* [20, 59, 76, 90, 94, 102, 103]	13 *(13.40%)* [9, 31, 40, 61, 68, 70, 85, 90, 100, 110]	2 *(9.09%)* [29, 123]
	Use of data	7 *(9.21%)* [43, 59, 61, 92, 99]	2 *(2.06%)* [65, 117]	-
	Policy	6 *(7.89%)* [11, 68, 75, 78, 104, 128]	22 *(22.68%)* [9, 11, 29, 30, 32, 33, 40, 46, 58, 68, 75-77, 81, 87, 101, 106, 107]	2 *(9.09%)* [25, 125]
	Management response	5 *(6.58%)* [55, 68, 79, 92, 112]	2 *(2.06%)* [58, 115]	4 *(18.18%)* [29, 97, 115]
	Outcomes of analysis	4 *(5.26%)* [10, 88, 117]	1 *(1.03%)* [100]	-

For peer review only - http://bmjopen.bmj.com/site/about/guidelines.xhtml

	Resource	2 *(2.63%)* [55, 68]	3 *(3.09%)* [25, 75, 127]	1 *(4.54%)* [25]
	Ownership	1 *(1.32%)* [40]	4 *(4.12%)* [25, 52, 125]	6 *(27.27%)* [25, 77]
	Hierarchy	1 *(1.32%)* [36]	-	-
	Size	-	3 *(3.09%)* [25, 26, 49]	1 *(4.54%)* [26]
	Nursing quality	-	1 *(1.03%)* [97]	-
	Awareness	-	1 *(1.03%)* [100]	-
	Location	-	-	1 *(4.54%)* [26]
	Elapsed time of IRS integration	-	-	1 *(4.54%)* [25]
	Ward rounds	-	-	1 *(4.54%)* [25]
	**Total**	**76** ***(100%)***	**97** ***(100%)***	**22** ***(100%)***
Team Factors	Relationships	13 *(39.39%)* [11, 27, 32, 55, 58, 66, 74, 87, 88, 90, 100]	2 *(10.00%)* [49, 82]	-
	Influence of Seniors	7 *(21.21%)* [37, 42, 74, 82, 106, 110]	1 *(5.00%)* [87]	-
	Peer reporting	5 *(15.15%)* [79, 85, 103]	3 *(15.00%)* [97, 98, 101]	-
	Teamwork/communication	3 *(9.09%)* [11, 36, 75]	7 *(35.00%)* [39, 75, 77, 122]	2 *(66.67%)* [123]
	Support/encouragement	3 *(9.09%)* [8, 87, 100]	1 *(5.00%)* [87]	1 *(33.33%)* [72]
	Medical doctor involvement	1 *(3.03%)* [44]	1 *(5.00%)* [44]	-
	Error committed by junior staff	1 *(3.03%)* [58]	1 *(5.00%)* [42]	-
	Team culture	-	4 *(20.00%)* [98, 107, 111, 122]	-
	**Total**	**33** *(100%)*	20 *(100%)*	**3** *(100%)*
Professional Ethics	Concealment	5 *(21.74%)* [85, 87, 120]	1 *(5.88%)* [11]	-
	Duty	1 *(4.35%)* [81]	8 *(47.06%)* [75, 85, 88, 95, 101, 107]	1 *(25.00%)* [125]
	Accountability	-	2 *(11.76%)* [88, 121]	-
	Responsibility	15 *(65.22%)* [8, 9, 34, 35, 44, 52, 70, 93, 94, 100, 104, 118, 121, 128]	5 *(29.41%)* [77, 90, 91, 94]	1 *(25.00%)* [26]
	Culture	2 *(8.70%)* [74, 87]	-	-
	Legal	-	1 *(5.88%)* [37]	2 *(50.00%)* [37]
	**Total**	**23** *(100%)*	**17** *(100%)*	**4** *(100%)*

For peer review only - http://bmjopen.bmj.com/site/about/guidelines.xhtml

PRISMA 2009 Checklist

Section/topic	#	Checklist item	Reported on page #
**TITLE**			
Title	1	Identify the report as a systematic review, meta-analysis, or both.	1
**ABSTRACT**			
Structured summary	2	Provide a structured summary including, as applicable: background; objectives; data sources; study eligibility criteria, participants, and interventions; study appraisal and synthesis methods; results; limitations; conclusions and implications of key findings; systematic review registration number.	2
**INTRODUCTION**			
Rationale	3	Describe the rationale for the review in the context of what is already known.	4
Objectives	4	Provide an explicit statement of questions being addressed with reference to participants, interventions, comparisons, outcomes, and study design (PICOS).	4
**METHODS**			
Protocol and registration	5	Indicate if a review protocol exists, if and where it can be accessed (e.g., Web address), and, if available, provide registration information including registration number.	N/A
Eligibility criteria	6	Specify study characteristics (e.g., PICOS, length of follow-up) and report characteristics (e.g., years considered, language, publication status) used as criteria for eligibility, giving rationale.	5-6
Information sources	7	Describe all information sources (e.g., databases with dates of coverage, contact with study authors to identify additional studies) in the search and date last searched.	5
Search	8	Present full electronic search strategy for at least one database, including any limits used, such that it could be repeated.	5
Study selection	9	State the process for selecting studies (i.e., screening, eligibility, included in systematic review, and, if applicable, included in the meta-analysis).	5-6
Data collection process	10	Describe method of data extraction from reports (e.g., piloted forms, independently, in duplicate) and any processes for obtaining and confirming data from investigators.	6-7
Data items	11	List and define all variables for which data were sought (e.g., PICOS, funding sources) and any assumptions and simplifications made.	6-7
Risk of bias in individual studies	12	Describe methods used for assessing risk of bias of individual studies (including specification of whether this was done at the study or outcome level), and how this information is to be used in any data synthesis.	7
Summary measures	13	State the principal summary measures (e.g., risk ratio, difference in means).	7-8
Synthesis of results	14	Describe the methods of handling data and combining results of studies, if done, including measures of consistency (e.g., I^2) for each meta-analysis.	7-9

For peer review only

PRISMA 2009 Checklist

Section/topic	#	Checklist item	Reported on page #
Risk of bias across studies	15	Specify any assessment of risk of bias that may affect the cumulative evidence (e.g., publication bias, selective reporting within studies).	N/A
Additional analyses	16	Describe methods of additional analyses (e.g., sensitivity or subgroup analyses, meta-regression), if done, indicating which were pre-specified.	N/A
**RESULTS**			
Study selection	17	Give numbers of studies screened, assessed for eligibility, and included in the review, with reasons for exclusions at each stage, ideally with a flow diagram.	9-10
Study characteristics	18	For each study, present characteristics for which data were extracted (e.g., study size, PICOS, follow-up period) and provide the citations.	9-10
Risk of bias within studies	19	Present data on risk of bias of each study and, if available, any outcome level assessment (see item 12).	N/A
Results of individual studies	20	For all outcomes considered (benefits or harms), present, for each study: (a) simple summary data for each intervention group (b) effect estimates and confidence intervals, ideally with a forest plot.	10
Synthesis of results	21	Present results of each meta-analysis done, including confidence intervals and measures of consistency.	12-21
Risk of bias across studies	22	Present results of any assessment of risk of bias across studies (see Item 15).	N/A
Additional analysis	23	Give results of additional analyses, if done (e.g., sensitivity or subgroup analyses, meta-regression [see Item 16]).	N/A
**DISCUSSION**			
Summary of evidence	24	Summarize the main findings including the strength of evidence for each main outcome; consider their relevance to key groups (e.g., healthcare providers, users, and policy makers).	22-25
Limitations	25	Discuss limitations at study and outcome level (e.g., risk of bias), and at review-level (e.g., incomplete retrieval of identified research, reporting bias).	25-26
Conclusions	26	Provide a general interpretation of the results in the context of other evidence, and implications for future research.	26-27
**FUNDING**			
Funding	27	Describe sources of funding for the systematic review and other support (e.g., supply of data); role of funders for the systematic review.	27-28

From: Moher D, Liberati A, Tetzlaff J, Altman DG, The PRISMA Group (2009). Preferred Reporting Items for Systematic Reviews and Meta-Analyses: The PRISMA Statement. PLoS Med 6(7): e1000097. doi:10.1371/journal.pmed1000097

For more information, visit: www.prisma-statement.org.

For peer review only - http://bmjopen.bmj.com/site/about/guidelines.xhtml

Development of a Theoretical Framework of Factors Affecting Patient Safety Incident Reporting: A Theoretical Review of the Literature

Journal:	*BMJ Open*
Manuscript ID	bmjopen-2017-017155.R1
Article Type:	Research
Date Submitted by the Author:	24-Jul-2017
Complete List of Authors:	Archer, Stephanie; Imperial College London, NIHR Imperial Patient Safety Translational Research Centre; Imperial College London,<br>Hull, Louise; King's College London<br>Soukup, Tayana; Imperial College London, NIHR Imperial Patient Safety Translational Research Centre<br>Mayer, Erik; Imperial College London, Dept. of Biosurgery and Surgical Technology<br>Athanasiou, Thanos; Imperial College London, Surgery and Cancer<br>Sevdalis, Nick; King's College London,<br>Darzi, Ara; Imperial College London, Institute of Global Health Innovation
**Primary Subject Heading**:	Health services research
Secondary Subject Heading:	Health policy
Keywords:	Incident reporting, Patient Safety, Service Quality

SCHOLARONE™ Manuscripts

For peer review only

Development of a Theoretical Framework of Factors Affecting Patient Safety Incident Reporting: A Theoretical Review of the Literature

Stephanie Archer, PhD*[1] Email: stephanie.archer@imperial.ac.uk

Louise Hull, PhD*[1,2] Email: louise.hull@kcl.ac.uk

Tayana Soukup, MSc[1] Email: t.soukup@imperial.ac.uk

Erik Mayer, PhD[1] Email: e.mayer@imperial.ac.uk

Thanos Athanasiou, PhD[1] Email: t.athanasiou@imperial.ac.uk

Nick Sevdalis, PhD[1,2] Email: nick.sevdalis@kcl.ac.uk

Ara Darzi, MD[1] Email: a.darzi@imperial.ac.uk

*SA & LH co-first author

Corresponding author: Dr Stephanie Archer, Research Fellow, NIHR Imperial Patient Safety Translational Research Centre, Imperial College London, Medical School Building, Room 5.03, St Mary's Campus, Norfolk Place, London, W2 1PG. Email: Stephanie.archer@imperial.ac.uk Telephone: 020 7594 3192 Fax: 02075943137

[1] Patient Safety Translational Research Centre, Imperial College London, London, UK

[2] Centre for Implementation Science, King's College London, London, UK.

Key Words: Incident reporting, Patient Safety, Service Quality

For peer review only - http://bmjopen.bmj.com/site/about/guidelines.xhtml

Abstract

Objectives: The development and implementation of incident reporting systems within healthcare continues to be a fundamental strategy to reduce preventable patient harm and improve the quality and safety of healthcare. We sought to identify factors contributing to patient safety incident reporting.

Design: To facilitate improvements in incident reporting, a theoretical framework, encompassing factors that act as barriers and enablers of reporting, was developed. Embase, Ovid MEDLINE(R) and PsycINFO were searched to identify relevant articles published between January 1980 and May 2014. A comprehensive search strategy including MeSH terms and keywords was developed to identify relevant articles. Data were extracted by three independent researchers; to ensure the accuracy of data extraction, all studies eligible for inclusion were rescreened by two reviewers.

Results: The literature search identified 3,049 potentially eligible articles; of these, 110 articles, including over 29,726 participants, met the inclusion criteria. In total, 748 barriers were identified (frequency count) across the 110 articles. In comparison, 372 facilitators to incident reporting and 118 negative cases were identified. The top two barriers cited were fear of adverse consequences (161, representing 21.52% of barriers) and process and systems of reporting (110, representing 14.71% of barriers). In comparison, the top two facilitators were organisational (97, representing 26.08% of facilitators) and process and systems of reporting (75, representing 20.16% of facilitators).

Conclusion: A wide range of factors contributing to engagement in incident reporting exist. Efforts that address the current tendency to under-report must consider the full

For peer review only - http://bmjopen.bmj.com/site/about/guidelines.xhtml

range of factors in order to develop interventions as well as a strategic policy approach for improvement.

Article Summary – strengths and limitations

- The synthesis included quantitative, qualitative and mixed methods research and have not restricted the literature to specific incident reporting systems.
- Only articles published in English were included.
- The last systematic search for literature was conducted on 29/05/2014, meaning that literature published since this date will not have been included.
- Studies detailing interventions to improve incident reporting and studies detailing variations in engagement in incident reporting were not included.
- Large heterogeneity across studies in terms of outcome measures and methodologies meant conduction of meta-analysis was precluded.

For peer review only - http://bmjopen.bmj.com/site/about/guidelines.xhtml

Background

The development and implementation of incident reporting systems within healthcare continues to be a fundamental strategy to reduce preventable patient harm and improve the quality and safety of healthcare on a local, regional and national basis.[1, 2] Although coverage and sophistication vary widely, incident reporting systems have now been in place for more than a decade in a number of countries.[3]

A key factor that compromises the ability of incident reporting systems to improve patient safety is underreporting. In the United States it is estimated that 50-96% of incidents are not reported.[2, 4, 5] Failure to report patient safety incidents significantly hinders the underlying goals of incident reporting systems; low levels of reporting makes it is difficult at best to identify and prioritise patient safety risks, and hampers learning from such incidents and ultimately improvements in patient safety. Whilst debate continues to exist regarding whether all patient safety incidents should be reported,[6, 7], it is extremely important to understand the factors that act as barriers and facilitators to incident reporting so that 'sufficient' levels of reporting exist to facilitate learning and improvement.

A number of studies exploring barriers and facilitators to incident reporting have been conducted.[8-11] In addition, a number of literature reviews to identify barriers and facilitators to incident reporting have been published.[12-14] Although previous work has made a valuable contribution to our understanding of factors affecting incident reporting, previous work has been limited in scope (e.g. focusing on the psychological factors affecting incident reporting[14]; focusing on perceived barriers influencing incident reporting by nurses;[13] factors affecting reporting of incidents

For peer review only - http://bmjopen.bmj.com/site/about/guidelines.xhtml

related to medical devices and other healthcare technologies).[12] As such, to date, there has been no definitive synthesis and evaluation of the factors that prevent or promote reporting.

The primary aim of this theoretical review was to systematically identify the factors affecting patient safety incident reporting. The secondary aims were, firstly, to develop theoretical framework, of factors acting as barriers and facilitators to incident reporting to guide implementation of interventions to increase engagement, and, secondly, to determine the prevalence of factors to guide the development of interventions and policies to improve incident reporting.

review only

For peer review only - http://bmjopen.bmj.com/site/about/guidelines.xhtml

Methods

Theoretical Review

A theoretical review was conducted as the overarching goal of the review was to build explanation of factors affecting incident reporting. In line with a theoretical review both quantitative and qualitative data were eligible for inclusion and interpretive methods were used to synthesize findings.

Study searches and selection

A systematic search strategy was developed and an electronic search was carried out in three databases**:** Embase, Ovid MEDLINE(R) and PsycINFO. The last search was conducted on 29/05/2014; whilst the last search was conducted 2 years ago, this reflects the sheer volume of articles that were included in this review. Search terms included those related to patient safety incidents, incident reporting systems, and barriers and facilitators to engagement in reporting (see table 1 for full search terms). Time and language of publications was restricted from 1980 and English language.

TABLE 1 HERE

Eligibility criteria

Inclusion Criteria

1. Studies reporting factors influencing the likelihood of incident report engagement in any healthcare setting (e.g. primary and secondary healthcare) and employing any study designs (e.g. qualitative, quantitative, mixed-methods)

For peer review only - http://bmjopen.bmj.com/site/about/guidelines.xhtml

Exclusion Criteria

1. Studies reporting aspects of incident reporting systems and/or incident reporting perceived positively and/or negatively by healthcare professionals without data relating perceptions to incident reporting engagement
2. Studies reporting data relating to disclosure of patient safety incidents to patients or their families (a systematic review of the literature on patient/family disclosure has previously been published)[15]
3. Studies reporting data relating to the effectiveness of interventions to improve incident reporting (a systematic review of the literature on the effectiveness of interventions to increase clinical incident reporting in health care has previously been published.[13]
4. Studies reporting statistical models where the impact of individual barriers and facilitators to engagement in incident reporting was unable to be determined.

The eligibility criteria was developed to maintain a focus on factors having a direct impact upon incident reporting engagement rather than simply identifying and listing factors of incident reporting which were perceived positively or negatively by healthcare professionals. Identifying elements of incident reporting perceived positively or negatively by healthcare professionals does not equate to identify factors that have an impact on reporting behaviour. In such studies, it is not possible to determine the impact on reporting behaviour - the primary focus of this review.

Data extraction

For peer review only - http://bmjopen.bmj.com/site/about/guidelines.xhtml

After the removal of duplicates, two authors (SA and LH) independently reviewed all articles on the basis of the titles and abstract. Three authors (SA, LH and TS) reviewed the articles at full-text stage.Data was extracted using an extraction template. The following data was extracted: first author's name, year of publication, country, study design, study population, sample size, and factors that decrease (barriers), increase (facilitators) or were neither a barrier nor facilitator to engagement in incident reporting (negative cases). To ensure the accuracy of data extraction, all studies eligible for inclusion were rescreened by two reviewers (SA and LH).

Quality Assessment

Many assessment tools and checklists have been developed to appraise the quality and susceptibility to bias of studies (e.g. The Cochrane Collaboration's tool for assessing risk of bias in randomized trials;[16] AMSTAR tool to assess the methodological quality of systematic reviews;[17] tools to assess the quality of qualitative research studies).[18] The decision not to assess the quality of studies was made for a number of reasons. First, the large heterogeneity of study designs would have made comparisons between study designs difficult at best. Second, quality appraisal is not considered necessary for theoretical reviews.[19] Third, it has been argued that it is important, but difficult, to distinguish between 'quality of reporting' and the 'quality of a study'.[20] As such, articles were not excluded from the current review based on 'quality' nor was weight assigned to studies based on quality.

Data analysis and initial theoretical framework development

For peer review only - http://bmjopen.bmj.com/site/about/guidelines.xhtml

A grounded theory approach was used to guide the development of the theoretical framework. Grounded theory is associated with the discovery of theory from data systematically obtained from social research.[21] It has been identified as a method where thorough and theoretically relevant analysis of a topic can be reached, specifically within literature reviews.[22] In light of this, a three-stage approach was undertaken to develop a theory of factors contributing to engagement in patient safety incident reporting. The first stage, *coding*, includes identifying parts of the data that relate the phenomena in question (in this case, incident reporting). During this stage, known as *open coding* in the grounded theory literature, three authors (SA, LH & TS) read and re-read each paper and identified sections of the paper that were relevant to the research question. Initial concepts developed from these were noted down at this stage; in some cases these were consistent with pre-existing literature (e.g. in the case of a standardised scale), but in others allowed for unseen insights to develop across the data corpus (e.g. in qualitative studies). In the second stage, *conceptualising*, or *axial coding*, focused on grouping together the initial codes where there were relationships to form higher order categories. These were given names. Stage three, *categorising*, or *selective coding* focused on linking together similar higher order categories that contained similar concepts which could underpin the reasoning behind the way that the phenomena (in this case, incident reporting) could be explained. Figure 1 displays an example of how these stages were applied.

FIGURE 1 HERE

Engagement in these three stages allowed constant comparison between the articles in the dataset to be performed until a theoretical framework was confirmed.

For peer review only - http://bmjopen.bmj.com/site/about/guidelines.xhtml

The final theoretical framework was reviewed by another member of the research team (NS) and feedback regarding the category descriptors was incorporated. The final theoretical framework of factors contributing to patient safety incident reporting engagement is displayed in Table 2.

For peer review only

<u>TABLE 2 HERE</u>

The theoretical framework developed was used to organise the identification of factors found to affect incident reporting and to quantify their prevalence. This approach is consistent with existing frameworks in the patient safety literature, for example Lawton et al employed a similar approach to quantify the prevalence of factors contributing to patient safety incidents in hospital settings.[23]

Patient and public involvement

No patients were involved in setting the research question or the outcome measures, nor were they involved in the design and implementation of the study. We do not anticipate patients and the public being involved in the dissemination of the work.

Findings

The search identified 5,335 records. After duplicates and limits were applied (English language, date restrictions 1980-May 2014), 3,049 records were considered for inclusion. Of these 3,049 records, 2,700 were excluded based on title and abstract screening. A total of 349 articles were considered potentially relevant and were

For peer review only - http://bmjopen.bmj.com/site/about/guidelines.xhtml

assessed at full-text by two researchers (Kappa 0.70, p<0.001). Of 349 publications, 33 were not obtainable (requested through the British Library), leaving 314 articles assessed at full-text stage. From these, 80 articles met inclusion criteria.

The reference lists of all included articles were screened for potentially relevant publications, resulting in a further 30 articles that met the inclusion criteria. A total of 110 articles, including over 29,726 participants, were included in the final review (Figure 2). The total number of participants per study ranged from 8-2185 (mean=286.54; median: 134.00). Six studies did not report sample size, thus the sample size calculations represented above are based on 104 articles.[24-29] See eTable 1 for full data extraction.

FIGURE 2 HERE

Study characteristics

Empirical study types and design

In total 110 articles were included; these consisted of 76 quantitative studies (including 72 questionnaire-based studies, 1 secondary analysis of data study, 1 case control study, 1 descriptive study and 1 cohort study) , 21 qualitative studies (including 11 interview-based studies and 10 focus group studies) and 13 mixed-methods studies (1 semi-structured interview and documentary analysis-based study; 1 semi-structured interview and retrospective review of error reports-based study; 2 semi-structured interview and questionnaire-based study; 3 focus group and questionnaire-based studies; 1 semi-structured and structured interview-based study; 1 interview, focus group and analysis of event reports-based study; 1 focus group and semi-structured interview-based study; 1 retrospective analysis of

For peer review only - http://bmjopen.bmj.com/site/about/guidelines.xhtml

routinely collected data and questionnaire-based study; 2 focus groups, interview and questionnaire-based studies).

Countries (Table 3)

The review encompassed research spanning four continents and over 20 countries. The four countries contributing the most studies were the United States of America (n=33), the United Kingdom (n=24), Australia (n=8), and Canada (n=8).

TABLE 3 HERE

(Please note that this table includes all 110 references)

Year of Publication

A steady increase in articles was evident over decades: 1980's (n=1),[51] 1990's (n=12),[24, 45, 52, 54, 67, 72, 76, 80, 81, 85, 103, 121] 2000's (n=58), [8-11, 28-35, 37, 40-44, 46-50, 53, 55-59, 64, 66, 69, 74, 75, 77-79, 82, 84, 91-94, 99, 101, 107, 110, 112, 114, 116-119, 125-129] 2010-May 2014 (n=39).[25-27, 36, 38, 39, 60-63, 65, 68, 70, 71, 73, 83, 86-90, 95-98, 100, 102, 104-106, 108, 109, 111, 113, 115, 120, 122-124] This increase is likely to reflect the growing integration of incident reporting systems in healthcare systems worldwide and the increasing realisation that healthcare professionals (HCPs) engagement in incident reporting is far from ideal.

The frequency of barriers and facilitators to incident reporting across the 110 articles, was calculated and rank ordered across the data (Figure 3). Where contributing factors were found not to be barriers or facilitators to incident reporting (e.g. if fear was found not to be a significant predictor of decreased or increased incident reporting), these were counted as negative cases. These negative cases were included to provide a more complete view of the data, and to prevent reporting bias.

For peer review only - http://bmjopen.bmj.com/site/about/guidelines.xhtml

When the same barrier, facilitator or negative case (e.g. fear of adverse consequences) was mentioned more than once within an article, this was reflected in the frequency data presented. In total, 748 barriers to incident reporting were identified (frequency count) compared with 372 facilitators. A total of 118 negative cases were identified. The top two barriers cited were fear of adverse consequences (161, representing 21.52% of barriers) and process and systems of reporting (110, representing 14.71% of barriers). In comparison, the top two facilitators were organisational (97, representing 26.08% of facilitators) and process and systems of reporting (75, representing 20.16% of facilitators). These results illustrate that the factors identified in this review of the literature can act as both a barrier and a facilitator to incident reporting systems depending on context; for example, *process and systems of reporting* was found to be the second most frequently cited barrier, as well as the second most frequently cited facilitator to incident reporting engagement. Whilst this may initially appear contradictory, when considering the complexity/simplicity of reporting it was found that highly complex incident reporting processes and systems were a barrier to incident reporting, whereas simple processes and systems were found to be a facilitator.

FIGURE 3 HERE

Frequency of Barriers to Patient Safety Incident Reporting (eTable 2)

Barriers to incident reporting were mentioned 748 times across the 110 articles (see eTable 2). The three most frequently mentioned barriers to incident reporting included *fear of adverse consequences* (161/748), *process and systems of reporting* (110/748) and *incident characteristics* (92/748).

For peer review only - http://bmjopen.bmj.com/site/about/guidelines.xhtml

Fear of Adverse Consequences

Fear of adverse consequences, as a barrier, was mentioned 161 times, and included a general fear of adverse consequences associated with incident reporting (51/161),[8, 10, 11, 27, 30, 32, 33, 35-37, 42-45, 53-56, 58, 59, 61, 68, 75, 78, 79, 85, 87, 88, 92, 97, 99, 100, 104, 106, 109, 118, 120, 121] fear of litigation (30/161), [8-11, 24, 27, 32, 35, 48, 51, 52, 61, 69, 72, 77, 80, 81, 85, 87, 88, 93, 100, 101, 103, 105, 107, 114, 117, 124, 128] and the fear of blame (24/161). [8, 10, 32, 35, 43, 44, 46, 58-61, 68, 70, 72, 78, 79, 82, 87, 90, 92, 99, 106] Additionally, the fear of judgment (22/161), [10, 24, 35, 43, 53, 59, 67, 79, 80, 88, 92, 99, 104, 107, 109, 116, 126], the fear of the negative impact that incident reporting could have on relationships with other HCPs, patients and the public (12/161), [10, 11, 36, 44, 46, 48, 54, 59, 92, 104, 116, 120] and the fear of a detrimental impact that reporting an incident could have on HCPs career (10/161), [10, 11, 27, 58, 59, 79, 86, 92, 93, 126] such as for example fear of job loss, were also cited as common barriers. Other less frequently mentioned barriers included protection of self (7/161), [24, 76, 80, 107, 122, 127] avoidance of discussion in meetings (4/161), [8, 69, 87, 117] and apprehension of sending an inappropriate form (1/161).[75]

Process and Systems of Reporting

Process and systems of reporting was mentioned as a barrier to reporting 110 times. The most frequently identified barrier to incident reporting was the time required to complete an incident report (29/110), [8, 11, 27, 38, 43, 48, 57, 69, 74, 78, 79, 81, 85, 87, 88, 90, 92, 93, 99-101, 105-107, 114, 118, 121] followed by the complexity of the reporting process (28/110). [8, 9, 11, 31, 33, 35, 38, 44, 46, 51, 73, 78, 79, 88-90, 93, 100, 101, 105-107, 117, 118, 125] Other process and systems of reporting barriers included lack of anonymity and/or confidentiality in reporting (22/110), [8, 11, 24, 27, 35, 48, 50, 68, 73, 74, 76-78, 80, 87, 101, 106, 107, 127] reporting format

For peer review only - http://bmjopen.bmj.com/site/about/guidelines.xhtml

(10/110), [31, 44, 82, 85, 90, 93, 100, 117] and the type of reporting system (e.g. paper-based) (5/110). [38, 50, 92, 117] Less frequently mentioned barriers included lack of information to complete report (3/110), [94, 107, 114] the focus of reporting (1/110), [78] and information to complete report not readily being available (1/110). [31]

Incident Characteristics

Incident characteristics were mentioned as a barrier to reporting 92 times. Level of harm, cause of incident, and frequency of incident were the most frequent incident characteristics acting as barriers to reporting (40/92, 19/42, and 18/92, respectively). HCPs were less likely to report an incident if the patient experienced no or minimal harm. [8, 11, 24, 31, 35, 42-48, 50, 51, 53, 54, 58, 65, 66, 69, 70, 72, 73, 80, 85, 87, 88, 92, 100, 103, 105, 106, 109, 114, 126, 128, 129] Incidents that were deemed to occur frequently were considered too well-known to report. [31, 51, 66, 70, 75, 76, 84, 100, 101, 103, 114, 119, 121, 127-129] Furthermore, if the cause of the incident was deemed unpreventable this acted as a barrier to incident reporting. [35, 52, 66, 81, 82, 85, 100, 101, 103, 107, 114, 119, 124, 128, 129] Other barriers included the type of incident (13/92) [8, 33, 34, 52, 69, 81, 85, 92, 93, 100, 107, 117, 121] and the level of risk (2/110). [11, 58]

Individual HCP Characteristics

Barriers reflective of individual HCP characteristics were cited 89 times. Barriers included a negative attitude/lack of value placed on incident reporting (53/89), [8, 9, 35, 44, 46, 56, 61, 63, 64, 66, 68, 70, 73, 74, 76, 79, 81, 86-88, 92, 93, 99-101, 103, 105, 107, 109, 117, 118, 120, 121, 128] and the perception that incident reporting does not result in improvements typically underlined such negative attitudes and values. A number of studies found that HCPs fail to report incidents because they simply forget (9/89), [8, 27, 31, 72, 87, 93, 117, 119, 129]

For peer review only - http://bmjopen.bmj.com/site/about/guidelines.xhtml

and that the way HCPs perceive themselves can act as a barrier to reporting (9/89).[24, 36, 55, 80, 87, 107, 127] Less frequently mentioned barriers included emotional responses to the incident (6/89),[31, 58, 79, 82, 100] previous reporting behavior (5/89),[34, 37, 52, 60, 74] exposure to errors (2/89), [38, 97] and length of time in employment (2/89).[37]

Knowledge and Skills

Knowledge and skills were cited as barriers to incident reporting 84 times. The two most frequently mentioned barriers related to a lack of reporting clarity (36/84) [9, 11, 24, 27, 31, 35, 38, 44, 46, 51, 52, 70, 73, 76, 79, 80, 87, 88, 100, 101, 103, 105, 107, 114, 119, 121, 127, 128] and a lack of clarity regarding what constitutes an adverse event and/or near miss (31/84).[9, 11, 31, 35, 43, 44, 46, 51, 69, 74, 82, 85, 87, 88, 92, 93, 95, 99, 100, 105, 117, 121] This suggests that a lack of knowledge about what should be reported and how to do this act as barriers. Less frequently cited barriers included an inability in error recognition (7/84),[35, 75, 79, 92, 99, 106, 124] lack of training in reporting (5/84), [68, 76, 82, 86, 97] and lack of awareness (4/84).[35, 43, 106, 114]

Work Environment

Work environment was mentioned 80 times as a barrier to incident reporting. Workload/Priority (50/80) [9, 11, 24, 27, 31, 34, 35, 43, 48, 49, 51, 55-58, 61, 68-70, 72, 75-77, 80, 82, 83, 88-90, 92, 93, 100, 103, 117, 119, 120, 125, 127-129] and accessibility (27/80) [24, 27, 31, 34, 35, 51, 52, 56, 74, 75, 80, 82, 86, 93, 101, 105-107, 114, 117, 119, 121, 127] were the most frequently mentioned work environment barriers, suggesting that high workload does not allow for incident reporting to be prioritised, and that access to the reporting system is problematic (e.g. not enough computer work stations to access reporting forms).

For peer review only - http://bmjopen.bmj.com/site/about/guidelines.xhtml

Organisational Factors

Organisational factors were mentioned 76 times as a barrier to incident reporting. Lack of feedback and communication following incident reporting (26/76) [8, 9, 11, 35, 37, 43, 44, 56, 58, 59, 61, 62, 69, 78, 85-87, 90, 92, 99, 100, 106, 108, 117, 123] and the absence/lack of a positive reporting culture (17/76) [9, 10, 34, 35, 49, 66, 70, 81, 86, 90, 92, 114, 117, 118, 123] were the two most frequently mentioned organisational barriers to reporting. Less frequently mentioned were lack of organisational learning and improvement (7/76),[27, 35, 61, 68, 69, 85, 100] poor organisational use of data (7/76),[43, 59, 61, 92, 99] and poor management response to reports (5/76). [55, 68, 79, 92, 112]

Team Factors

Team factors were mentioned as barriers to engagement in incident reporting 33 times. The three most frequently mentioned barriers included the negative impact that incident reporting could have on working relationships (13/33),[11, 27, 32, 55, 58, 66, 74, 87, 88, 90, 100] the influence of seniors not to report (7/33), [37, 42, 74, 82, 106, 110] and how HCPs feel about reporting their peers (5/33).[79, 85, 103]

Professional Ethics

Professional ethics was the least frequently mentioned barrier to incident reporting (23/748). The most prevalent factor was a lack of personal responsibility to report (15/23) [8, 9, 34, 35, 44, 52, 70, 93, 94, 100, 104, 118, 121, 128] with studies suggesting that HCPs are less likely to report when they feel that reporting is the responsibility of someone else within the team. Concealment was also mentioned as a barrier (5/23).[85, 87, 120]

For peer review only - http://bmjopen.bmj.com/site/about/guidelines.xhtml

Frequency of Facilitators in Patient Safety Incident Reporting (Table e1)

Facilitators of reporting were mentioned 372 times across the 110 articles (see Table 2). Organisational factors were the most frequently mentioned facilitator to incident reporting (97/372), followed by process and systems of reporting (75/372) and incident characteristics (55/372).

Organisational Factors

Organisational factors were mentioned as facilitators 97 times. The two most frequently cited facilitators included the provision of feedback/communication following incident reporting (29/97) [9, 11, 30, 33, 41, 44, 46, 61, 65, 68, 70, 75-77, 87, 100, 101, 107, 112, 117] and a non-punitive incident reporting policy (22/97). [9, 11, 29, 30, 32, 33, 40, 46, 58, 68, 75-77, 81, 87, 101, 106, 107] The existence of a reporting culture (16/97) [29, 33, 39, 66, 75, 96, 100, 106, 110-112, 121, 122] and a focus on learning and improvement from incidents (13/97) [9, 31, 40, 61, 68, 70, 85, 90, 100, 110] were also facilitators to reporting.

Process and Systems of Reporting

Process and systems of reporting was mentioned as a facilitator 75 times. Reporting format, ensuring anonymity and/or confidentiality, and simplification of reporting were the three most frequently cited facilitators accounting for 21/75,[9, 11, 25, 30, 44, 46, 58, 61, 65, 68, 70, 75, 87, 100, 106, 107, 117] 16/75, [9, 11, 29, 31, 40, 44, 65, 68, 74, 87, 100, 106, 117] and 15/75 [9, 11, 30, 38, 65, 68, 73, 77, 81, 100, 101, 117] facilitators within this category. Less frequently mentioned process and systems of reporting facilitators included the type of reporting system used (e.g. electronic reporting) (11/75). [33, 34, 40, 44, 68, 73, 101, 117]

For peer review only - http://bmjopen.bmj.com/site/about/guidelines.xhtml

Incident Characteristics

Incident characteristics were mentioned as a facilitator to reporting 55 times. Level of harm and frequency of an incident were the most frequently cited incident characteristics identified as facilitators to reporting (26/55 [11, 31, 40, 42, 47, 50, 58, 66, 75, 77, 82, 85, 88, 95, 114, 121, 124, 125, 128] and 13/55, [11, 66, 75, 77, 114, 121, 124] respectively). Incidents resulting in severe harm (including death) were more likely to be reported and HCPs were more likely to report incidents that occur infrequently rather than frequently. Less frequently mentioned facilitators included the type of incident (8/55), [82, 85, 121] cause of the incident (6/55), [40, 66, 76, 77, 125] and level of risk (1/55).[58]

Individual HCP Characteristics

Individual HCP characteristics were mentioned 41 times as a facilitator. A positive attitude towards incident reporting and a high value placed on incident reporting was found to increase the likelihood of reporting (21/41). [9, 11, 40, 58, 68, 82, 88, 90, 93, 95, 97, 98, 107, 111, 125] HCPs emotional response to a patient safety incident was also found to increase the likelihood of reporting in a number of studies (5/41).[31, 58, 100] The professional group of HCPs was also found to act as a facilitator to reporting (5/41). [28, 71] Less frequently cited facilitators included previous reporting behavior (1/41), [29] number of hours worked (1/41), [52] and demographics (e.g. gender and age) (2/41). [37, 98]

Knowledge and Skills

Training in reporting was identified as the most frequently mentioned facilitator in this category (21/36).[9, 25, 33, 70, 73, 75, 76, 87, 101, 106, 117, 127] Other facilitators included knowledge regarding what constitutes an adverse event/near miss and the ability to

For peer review only - http://bmjopen.bmj.com/site/about/guidelines.xhtml

recognise an error has occurred (7/36 [9, 30, 44, 46, 70, 87, 100] and 4/36, [75-77, 124] respectively).

Team Factors

Team factors were mentioned 20 times as a facilitator to reporting. Good teamwork/communication (7/20) [39, 75, 77, 122] and a positive team culture (4/20) [98, 107, 111, 122] were the most frequently cited facilitators.

Professional Ethics

Professional ethics was cited as a facilitator 17 times. A strong sense of duty (8/17) [75, 85, 88, 95, 101, 107] and responsibility (5/17) [77, 90, 91, 94] to report increased the likelihood of reporting. Less frequently cited facilitators included accountability (2/17) [88, 121] and a legal obligation to report (1/17).[37]

Work Environment

Work environment was mentioned as a facilitator 18 times. Access to the incident reporting system (11/18), [30, 68, 73-75, 87, 100, 101, 117] and those whose workloads allowed for and those that prioritised incident reporting increased the likelihood of reporting.

Fear of Adverse Consequences

Fear of adverse consequences was mentioned as a facilitator to reporting 13 times and included a fear of litigation and fear of blame increasing the likelihood of reporting (8/13 [9, 11, 27, 33, 82, 88, 90] and 4/13,[9, 11, 87, 88] respectively).

Frequency of Negative Cases (Table e1)

For peer review only - http://bmjopen.bmj.com/site/about/guidelines.xhtml

For peer review only

Negative cases were identified 118 times across the 110 articles (see Table 2). The three most frequently mentioned factors included individual HCP characteristics (43/118), organisational factors (22/118), and knowledge and skills (15/118).

Individual HCP characteristics were mentioned as a negative case 43 times. HCP's attitude and value of incident reporting did not have an impact on reporting behavior (12/43). [37, 48, 54, 72, 79, 96, 129] Similarly, HCPs demographics (e.g. age, gender) had no impact on the likelihood of reporting (12/43).[37, 49, 51, 52, 77, 96, 97, 125, 129] Other less frequently mentioned factors included seniority (4/43),[37, 77, 125, 129] forgetfulness (1/43),[129] previous reporting behavior (1/43),[129] and number of hours worked (1/43).[26] Organisational factors were cited as having no impact on incident reporting 22 times. The most frequently mentioned were the ownership of the organisation (e.g. private/public funded) (6/22)[25, 77] and management response towards incident reporting (4/22).[29, 97, 115] Knowledge and skills were mentioned 15 times. These included the clarity of the reporting mechanism (5/15),[29, 48, 72, 129] knowledge of what constitutes an adverse event/near miss (2/15),[48, 72] ability in error recognition (1/15),[48] and training in error reporting (7/15).[25, 77, 86, 129]

Fear of adverse consequences was cited as having no impact on engagement in incident reporting 12 times. These included a fear of litigation (4/12),[24, 40, 48, 90] a general fear of adverse consequences (3/12),[72, 85, 96] blame (1/12) [48], judgment (1/12),[101] and impact on career (1/12).[125] Work environment was mentioned as as having no impact on reporting 10 times, including workload/priority (3/10)[51, 123, 125] and unit type (3/10).[49, 112] Other less frequently cited work environment factors

For peer review only - http://bmjopen.bmj.com/site/about/guidelines.xhtml

included physical work conditions (1/10),[26] satisfaction with work environment (1/10),[113] and accessibility (1/10).[48]

Across all studies, process and systems of reporting was mentioned 7 times as having no impact on incident reporting; these included reporting format (3/7),[25, 68, 125] complexity/simplification of reporting (1/7),[68] and anonymity and/or confidentiality (1/7).[24] Professional ethics were only mentioned four times as having no impact on the likelihood of incident reporting; these were legal obligation (2/4),[37] duty (1/4),[125] and responsibility (1/4).[26] Team factors were cited as having no impact on the likelihood of reporting 3 times, including teamwork and communication (2/3)[123] and support/encouragement to report (1/3).[109] Incident characteristics were the least frequently mentioned factor which had no impact on reporting. Cause of incident was found to have no impact on engagement in reporting (2/2).[125, 129]

For peer review only

Discussion

It has been suggested that there is a tendency in healthcare to encourage reporting of any and all patient safety incidents, to celebrate large quantities of incident reports and to aim for ever-increasing overall reporting rates. Whilst there are numerous problems associated with this approach[7] (e.g. flooding the system to such a degree that the thorough investigation of each incident reporting is unachievable), it is clear that high levels of underreporting seriously compromises the ability of incident reporting systems to facilitate learning and improvement in patient safety.

This is the first theoretical literature review of factors contributing to patient safety incident reporting. Based on the evidence from 110 articles, we developed a theoretical framework, based on the principles of grounded theory, which summarises a wide range of factors contributing to incident reporting. We purposely sought publications from a range of countries, covering diverse health systems and study populations with a view to incorporating these into one broad theoretical framework. We argue that this is an appropriate approach for this initial explorative work, as multiple theoretical frameworks for individual counties, settings and populations (e.g. nurses working in mental health settings in Australia), would have limited application at this point in time. However, we suggest that those interested in exploring barriers and facilitators in specific settings conduct further research using the theoretical framework presented here.

To improve incident reporting (both the quantity and/or quality) and facilitate the successful implementation of incident reporting systems, we suggest that the

For peer review only - http://bmjopen.bmj.com/site/about/guidelines.xhtml

theoretical framework is best used to prospectively and systematically identify factors within a given context that are likely to affect incident reporting. Those responsible for the effective implementation of incident reporting systems should explore each of the factors listed in our framework for salience. Rather than the framework being used in isolation, we recommend that it be used in conjunction with other implementation theories/frameworks and models to guide, understand and evaluate implementation of incident reporting systems.[130] Based on such prospective analysis, strategies to enhance the adoption, implementation, and sustainability of incident reporting systems can be tailored and selected according to a given setting. As such, using the developed framework will advance our understanding of how to optimally implement incident reporting systems into practice.

We used the developed theoretical framework, based on the evidence-base, to organise our findings and have presented the frequency and rank order (i.e. prevalence) of factors contributing to incident reporting. Whilst this approach is consistent with other frameworks in the patient safety literature,[14, 23] it may be considered as a crude analysis of the existing literature and needs to be interpreted with caution; we acknowledge that it is possible, although unlikely, that a relationship between the number of times a given factor is mentioned in the literature and its impact on incident reporting behaviour might not exist. However, we have been able to provide the first high level overview of a large heterogeneous body of evidence. Furthermore, we acknowledge that weighting the impact of each factor would have been advantageous, however the data did not lend itself to this possibility and we propose that it might not be possible to simply weight factors because of the complex and dynamic interrelationships that are likely to exist between them. Alternatively, we

For peer review only - http://bmjopen.bmj.com/site/about/guidelines.xhtml

For peer review only

suggest that modelling the interrelationships between factors affecting incident reporting engagement is an avenue for future research.

Our results suggest that fear of adverse consequences and ineffective processes/systems of reporting are high priority areas that require consideration to improve engagement in incident reporting. Changes to policy should be considered at an institutional or national level to prevent fear of litigation and blame, as fear of adverse consequences was found to inhibit incident reporting. We believe that it is unlikely that changes made within a single hospital or healthcare system would instill significant reassurance to promote incident reporting. In addition, at an organisational level we found that appropriate systems and processes for reporting need to be implemented to improve incident reporting; simultaneously, lack of, or poorly designed systems significantly hinder reporting. These aspects of reporting rely on well-designed processes and technologies and are arguably the responsibility of the organisational leaders. There is no ‘optimum model’ for incident reporting systems (e.g. electronic, confidential, anonymous) - systems need to be responsive to users and organisational needs.

Organisational factors and processes/systems of reporting were identified as the two most frequently cited facilitators of reporting, which suggests that healthcare organisations consider these as high priority areas which should be the target of increased focus and resources. For example, our results suggest that organisational policies that foster a reporting and learning culture as well as providing feedback following a report will promote incident reporting. Interestingly, we found that individual HCP characteristics have little impact on engagement in incident reporting.

For peer review only - http://bmjopen.bmj.com/site/about/guidelines.xhtml

For peer review only

This suggests that organisations should be cautious before investing significant resources in these factors, as such investment may result in minimal returns.

Although we have considered the above factors in isolation as illustrative examples, it is important to consider the interconnecting relationships between factors in order to develop intervention packages to improve engagement in incident reporting. Our results suggest that a comprehensive intervention/policy package which targets more than one contributing factor (e.g. establishing a supportive work environment, with mechanisms which optimise shared learning, alongside a national policy to minimise the fear of adverse consequence) is far more likely to result in increased engagement in incident reporting in comparison to interventions that simply target one factor.

Strengths and Limitations

In order to identify as much relevant literature as possible, we have included quantitative, qualitative and mixed methods research and have not restricted the literature to specific incident reporting systems, i.e. departmental, local, regional and national. In addition, the studies included a vast array of health care settings and providers, maximising the generalisability of the results. The resulting evidence has been synthetised into a practical output i.e. a theoretical framework to guide efforts to improve engagement in incident reporting.

The results, and recommendations proposed in this evidence synthesis must be considered in light of several limitations. First, only articles published in English were included, which may generate bias. However, articles spanning four continents from

For peer review only - http://bmjopen.bmj.com/site/about/guidelines.xhtml

over 20 countries were identified, hence we are confident that our findings are of high external validity to guide safety policy globally. Secondly, the last systematic search for literature was conducted on 29/05/2014, meaning that literature published since this date will not have been included. We suggest that literature published after the last search could be useful to test the validity of the theoretical framework. Thirdly, the decision not to include studies detailing interventions to improve incident reporting and studies detailing variations in engagement in incident reporting may skew the findings. This decision was made as it was not possible to determine the relative contribution of individual factors on engagement in incident reporting within such studies. Fourthly, large heterogeneity across studies in terms of outcome measures and methodologies meant conduction of meta-analysis was precluded. This having been said, the synthesis of barriers and facilitators into frequency of reporting provides some evidence towards their respective relative importance, although it is accepted that the frequency of factors may represent those that have been the subject of more research. We recommend that future research applies and evaluates the usefulness of the developed theoretical framework in exploring and improving incident reporting in a variety of settings (e.g. primary and secondary healthcare).

Future Research

There are many ways in which future research could test the validity of the theoretical framework presented in the current study. For example, content validity of the theoretical framework could be assessed using expert consensus methods (e.g. Delphi study). In addition, predictive validity could be tested quantitatively by assessing the correlation between, for example, fear of adverse consequences (level

For peer review only - http://bmjopen.bmj.com/site/about/guidelines.xhtml

of fear) and incident reporting behaviour (i.e. number of incidents reported). A negative correlation between number of incidents reported (low) and fear of adverse consequence (high) would provide evidence for predictive validity of the theoretical framework.

Summary/conclusion

A wide range of factors contributing to engagement in incident reporting exist across varying levels of the healthcare system. Efforts aimed at addressing the current tendency to underreport must consider the full range of factors in order to develop tailored interventions and policy packages for improvement. We suggest the theoretical framework developed here would be useful in understanding factors affecting incident reporting engagement, increasing engagement in incident reporting and ultimately learning from patient safety incidents.

Acknowledgements:

We would like to thank Kelsey Flott, BA, MSc, from the Centre for Health Policy, Imperial College London, for critically reviewing the manuscript.

Competing Interests:

We declare that we have no competing interests.

Ethical Approval:

None

Funding:

For peer review only - http://bmjopen.bmj.com/site/about/guidelines.xhtml

This article represents independent research supported by the National Institute for Health Research (NIHR) Imperial Patient Safety Translational Research Centre. The views expressed are those of the author(s) and not necessarily those of the NHS, the NIHR or the Department of Health. Hull and Sevdalis' research was supported by the National Institute for Health Research (NIHR) Collaboration for Leadership in Applied Health Research and Care South London at King's College Hospital NHS Foundation Trust. Hull and Sevdalis are members of King's Improvement Science, which is part of the NIHR CLAHRC South London and comprises a specialist team of improvement scientists and senior researchers based at King's College London. Its work is funded by King's Health Partners (Guy's and St Thomas' NHS Foundation Trust, King's College Hospital NHS Foundation Trust, King's College London and South London and Maudsley NHS Foundation Trust), Guy's and St Thomas' Charity, the Maudsley Charity and the Health Foundation. The views expressed are those of the authors and not necessarily those of the NHS, the NIHR or the Department of Health.

For peer review only

Authorship/Contributor statement:

- Conception or design of the work: Stephanie Archer, Louise Hull, Erik Meyer, Nick Sevdalis, Ara Darzi
- Data collection: Stephanie Archer, Louise Hull, Tayana Soukup
- Data analysis and interpretation Stephanie Archer, Louise Hull, Thanos Athanasiou, Nick Sevdalis
- Drafting the article: Stephanie Archer, Thanos Athanasiou, Nick Sevdalis
- Critical revision of the article: Stephanie Archer, Louise Hull, Tayana Soukup, Erik Meyer, Thanos Athanasiou, Nick Sevdalis, Ara Darzi

For peer review only - http://bmjopen.bmj.com/site/about/guidelines.xhtml

- Final approval of the version to be published: Stephanie Archer, Louise Hull, Tayana Soukup, Erik Meyer, Thanos Athanasiou, Nick Sevdalis, Ara Darzi

Data sharing

All data from this systematic review and theoretical framework is presented within the publication.

For peer review only

References

1. Barach P, Small S. Reporting and preventing medical mishaps: lessons from non-medical near miss reporting systems. *BMJ* 2000;320:759-63.

2. Leape L. Error in medicine. *JAMA* 1994;272:1851-57.

3. Department of Health. An organisation with a memory: report of an expert group on learning from adverse events in the NHS. Available from: https://psnet.ahrq.gov/resources/resource/1568. Last accessed March 2017.

4. Nobel D, Pronovost P. Undereporting of patient safety incidents reduces health-cares ability to quantify and accurately measure harm reduction. *J Patient Saf* 2010;6(4): 247-50.

5. Institute of Medicine. To err is human: building a safety health system. Washington, DC: National Academy Press; 1999.

6. Howell AM, Burns EM, Hull L, et al. . International recommendations for national patient safety incident reporting systems: an expert Delphi consensus-building process. *BMJ Qual Saf* Online first: 22 Feb 2016. . doi: 10.1136/bmjqs-2015-004456. Online First: 5 February 2004. doi:10.1136/ard.2003.001234

7. Macrae C. The problem with incident reporting. *BMJ Qual Saf* 2016;25(2):71-5.

8. Evans SM, Berry JG, Smith BJ, et al. Attitudes and barriers to incident reporting: A collaborative hospital study. *Qual Saf Health Care* 2006;15(1):39-43.

9. Kingston MJ, Evans SM, Smith BJ, et al. Attitudes of doctors and nurses towards incident reporting: A qualitative analysis. *Med J Aust* 2004;181(1):36-9.

10. Waring JJ. Beyond blame: Cultural barriers to medical incident reporting. *Soc Sci Med* 2005;60(9):1927-35.

11. Jeffe DB, Dunagan WC, Garbutt J, et al. Using focus groups to understand physicians' and nurses' perspectives on error reporting in hospitals. *Jt Comm J Qual Saf* 2004;30(9):471-9.

For peer review only - http://bmjopen.bmj.com/site/about/guidelines.xhtml

12. Polisena J, Gagliardi A, Urbach D, et al. Factors that influence the recognition, reporting and resolution of incidents related to medical devices and other healthcare technologies: a systematic review. *Syst Rev* 2015:29;4:37.

13. Fung WM, Koh SS, Chow YL. Attitudes and perceived barriers influencing incident reporting by nurses and their correlation with reported incidents: A systematic review. *JBI Libr Syst Rev* 2012;10(1):1-65.

14. Pfeiffer Y, Manser T, Wehner T. Conceptualising barriers to incident reporting: a psychological framework. *Qual Saf Health Care* 2010;19(6):e60.

15. O'Connor E, Coates HM, Yardley IE, et al. Disclosure of patient safety incidents: a comprehensive review. *Int J Qual Health Care* 2010;22(5):371-9.

16. Higgins JP, Altman DG, Gøtzsche PC, et al. The Cochrane Collaboration's tool for assessing risk of bias in randomised trials. *BMJ* 2011;343:d5928.

17. Shea BJ, Grimshaw JM, Wells GA, et al. Development of AMSTAR: a measurement tool to assess the methodological quality of systematic reviews. *BMC Med Res Methodol* 2007;7:10.

18. Long AF, Godfrey M. An evaluation tool to assess the quality of qualitative research studies. *Int J Social Research Methodology* 2004;7(2):181-96.

19. Paré G, Trudel MC, Jaanac M, et al. Synthesizing information systems knowledge: A typology of literature reviews. *Information & Management* 2015;52(2):183-99.

20. Sanderson S, Tatt ID, Higgins JP. Tools for assessing quality and susceptibility to bias in observational studies in epidemiology: a systematic review and annotated bibliography. *Int J Epidemiol* 2007;36(3):666-76.

21. Glaser B, Strauss A. The Discovery of Grounded Theory. Hawthorne, New York: Aldine Publishing Company; 1967.

22. Wolfswinkel J, Furtmueller E, Milderon C. Using grounded theory as a method for rigorously reviewing literature. *Eur J Inf Syst* 2013;22(1):45-55.

For peer review only

For peer review only - http://bmjopen.bmj.com/site/about/guidelines.xhtml

23. Lawton R, McEachan RR, Giles SJ, et al. Development of an evidence-based framework of factors contributing to patient safety incidents in hospital settings: a systematic review. *BMJ Qual Saf* 2012;21(5):369-80.

24. Belton, KJ. Attitude survey of adverse drug-reaction reporting by health care professionals across the European Union. *Eur J Clin Pharmacol* 1997;52(6):423-7.

25. Fukuda H, Imanaka Y, Hirose M, et al. Impact of system-level activities and reporting design on the number of incident reports for patient safety. *Qual Saf Health Care* 2010;19(2):122-7.

26. Gaal S, van den Hombergh P, Verstappen W, et al. Patient safety features are more present in larger primary care practices. *Health Policy* 2010;97(1): 87-91.

27. Jennings PA, Stella J. Barriers to incident notification in a regional prehospital setting. *Emerg Med J* 2011;28(6):526-9.

28. Rowin EJ, Lucier D, Pauker SG, et al. Does error and adverse event reporting by physicians and nurses differ? *Jt Comm J Qual Patient Saf* 2008;34(9):537-45.

29. Hutchinson A, Young TA, Cooper KL, et al. Trends in healthcare incident reporting and relationship to safety and quality data in acute hospitals: Results from the National Reporting and Learning System. *Qual Saf Health Care* 2009;18(1):5-10.

30. Beasley JW, Escoto KH, Karsh BT. Design Elements for a Primary Care Medical Reporting System. *WMS* 2004;103(1):56-9.

31. Elder NC, Graham D, Brandt E, et al. Barriers and motivators for making error reports from family medicine offices: a report from the American Academy of Family Physicians National Research Network (AAFP NRN). *J Am Board Fam Med* 2007;20(2):115-23.

32. Fairbanks RJ, Crittenden CN, O'Gara KG, et al. Emergency medical services provider perceptions of the nature of adverse events and near-misses in out-of-hospital care: An ethnographic view. *Acad Emerg Med* 2008;15(7):633-40.

33. Karsh BT, Escoto KH, Beasley JW, et al. Toward a theoretical approach to medical error reporting system research and design. *Appl Ergon* 2006;37(3):283-95.

For peer review only - http://bmjopen.bmj.com/site/about/guidelines.xhtml

34. Kennedy AG, Littenberg B. Medication error reporting by community pharmacists in Vermont. *J Am Pharm Assoc* 2004;44(4):434-8.

35. Handler SM, Perera S, Olshansky EF, et al. Identifying Modifiable Barriers to Medication Error Reporting in the Nursing Home Setting. *J Am Med Dir Assoc* 2007;8(9):568-74.

36. Church JA, Adams RD, Hendrix LH, et al. National study to determine the comfort levels of radiation therapists and medical dosimetrists to report errors. *Pract Radiat Oncol* 2013;3(4):e165-70.

37. Daly JM, Jogerst GJ. Association of knowledge of adult protective services legislation with rates of reporting of abuse in Iowa nursing homes. *J Am Med Dir Assoc* 2005;6(2):113-20.

38. Ehrenpreis ED, Sifuentes H, Ehrenpreis JE, et al. Suboptimal reporting of adverse medical events to the FDA Adverse Events Reporting System by nurse practitioners and physician assistants. *Expert Opin Drug Saf* 2012;11(2): 177-83.

39. Erler C, Edwards NE, Ritchey S, et al. Perceived Patient Safety Culture in a Critical Care Transport Program. *Air Med J* 2013;32(4):208-15.

40. Garbutt J, Brownstein DR, Klein EJ, et al. Reporting and disclosing medical errors: Pediatricians' attitudes and behaviors. *Arch Pediatr Adolesc Med* 2007;161(2):179-85.

41. Kaldjian LC, Jones EW, Wu BJ, et al. Reporting medical errors to improve patient safety: A survey of physicians in teaching hospitals. *Arch Intern Med* 2008;168(1):40-6.

42. Hohenhaus SM. Emergency Nursing and Medical Error-A Survey of Two States. *J Emerg Nurs* 2008;34(1):20-5.

43. Patrician PA, Brosch LR. Medication error reporting and the work environment in a military setting. *J Nurs Care Qual* 2009;24(4):277-86.

44. Schectman JM, Plews-Ogan ML. Physician perception of hospital safety and barriers to incident reporting. *Jt Comm J Qual Patient Saf* 2006;32(6):337-43.

For peer review only - http://bmjopen.bmj.com/site/about/guidelines.xhtml

45. Schulmeister L. Chemotherapy medication errors: descriptions, severity, and contributing factors. *Oncol Nurs Forum* 1999;26(6):1033-42.

46. Taylor JA, Brownstein D, Christakis DA. Use of incident reports by physicians and nurses to document medical errors in pediatric patients. *Pediatrics* 2004;114(3): 729-35.

47. Throckmorton T, Etchegaray J. Factors Affecting Incident Reporting by Registered Nurses: The Relationship of Perceptions of the Environment for Reporting Errors, Knowledge of the Nursing Practice Act, and Demographics on Intent to Report Errors. *J Perianesth Nurs* 2007;22(6):400-12.

48. Uribe CL, Schweikhart SB, Pathak DS, et al. Perceived barriers to medical-error reporting: An exploratory investigation. *J Healthc Manag* 2002;47(4): 263-79.

49. Vogus TJ, Sutcliffe KM. The impact of safety organizing, trusted leadership, and care pathways on reported medication errors in hospital nursing units. *Med Care* 2007;45(10):997-1002.

50. Weissman JS, Annas CL, Epstein AM, et al. Error reporting and disclosure systems: views from hospital leaders. *JAMA* 2005;293(11): 1359-66.

51. Rogers AS, Israel E, Smith CR, et al. Physician knowledge, attitudes, and behavior related to reporting adverse drug events. *Arch Intern Med* 1988;148(7):1596-1600.

52. Generali JA, Danish MA, Rosenbaum SE. Knowledge of and attitudes about adverse drug reaction reporting among Rhode Island pharmacists. *Ann Pharmacother* 1995;29(4):365-9.

53. Mayo AM, Duncan D. Nurse Perceptions of Medication Errors: What We Need to Know for Patient Safety. *J Nurs Care Qual* 2004;19(3): 209-17.

54. Osborne J, Blais K, Hayes JS. Nurses' Perceptions: When Is It a Medication Error? *J Nurs Adm* 1999;29(4):33-8.

55. Blegen MA, Vaughn T, Pepper G, et al. Patient and Staff Safety: Voluntary Reporting. *Am J Med Qual* 2004;19(2):67-74.

For peer review only - http://bmjopen.bmj.com/site/about/guidelines.xhtml

56. King ES, Moyer DV, Couturie MJ, et al. Getting doctors to report medical errors: project DISCLOSE. *Jt Comm J Qual Patient Saf* 2006;32(7): 382-92.

57. Coley KC, Pringle JL, Weber RJ, et al. Perceived Barriers in Using a Region-Wide Medication Error Reporting System. *J Patient Saf* 2006;2(1):39-44.

58. Elder, NC, Brungs SM, Nagy M, et al. Nurses' perceptions of error communication and reporting in the intensive care unit. *J Patient Saf* 2008; 4(3):162-8.

59. Stratton KM, Blegen MA, Pepper G, et al. Reporting of Medication Errors by Pediatric Nurses. *J Pediatr Nurs* 2004;19(6):385-92.

60. Anderson JE, Kodate N, Walters R, et al. Can incident reporting improve safety? Healthcare practitioners' views of the effectiveness of incident reporting. *Int J Qual Health Care* 2013;25(2):141-50.

61. Arfanis K, Smith A. Informal risk assessment strategies in health care staff: An unrecognized source of resilience? *J Eval Clin Pract* 2012;18(6): 1140-6.

62. Armitage G, Newell R, Wright J. Improving the quality of drug error reporting. *J Eval Clin Pract* 2010;16(6):1189-97.

63. Turner S, Ramsay A, Fulop N. The role of professional communities in governing patient safety. *J Health Organ Manag* 2013;27(4):527-43.

64. Waring, JJ. A qualitative study of the intra-hospital variations in incident reporting. *Int J Qual Health Care* 2004;16(5): 347-52.

65. Williams SD, Phipps DL, Ashcroft DM. Understanding the attitudes of hospital pharmacists to reporting medication incidents: A qualitative study. *Res Social Adm Pharm* 2013;9(1):80-9.

66. Ashcroft DM, Morecroft C, Parker D, et al. Likelihood of reporting adverse events in community pharmacy: An experimental study. *Qual Saf Health Care* 2006;15(1):48-52.

67. Gladstone J. Drug administration errors: a study into the factors underlying the occurrence and reporting of drug errors in a district general hospital. *J Adv Nurs* 1995;22(4):628-37.

For peer review only - http://bmjopen.bmj.com/site/about/guidelines.xhtml

68. Holmström AR, Airaksinen M, Weiss M, et al. National and local medication error reporting systems: a survey of practices in 16 countries. *J Patient Saf* 2012;8(4):165-76.

69. Kreckler S, Catchpole K, McCulloch P, et al. Handa AFactors influencing incident reporting in surgical care. *Qual Saf Health Care* 2009;18(2):116-20.

70. Parvizi N, Robertson I, McWilliams RG. Medical device adverse incident reporting in interventional radiology. *Clin Radiol* 2014;69(3):263-7.

71. Sarvadikar A, Prescott G, Williams D. Attitudes to reporting medication error among differing healthcare professionals. *Eur J Clin Pharmacol* 2010;66(8):843-53.

72. Vincent C, Stanhope N, Crowley-Murphy M. Reasons for not reporting adverse incidents: An empirical study. *J Eval Clin Pract* 1999;5(1):13-21.

73. Winchester SA, Tomkins S, Cliffe S, et al. Healthcare workers' perceptions of occupational exposure to blood-borne viruses and reporting barriers: a questionnaire-based study. *J Hosp Infect* 2012;82(1):36-9.

74. Sharma, A, Jain P, Parmar, B, et al. Incident Reporting in Surgical Trainees-Revisited. *J Patient Saf* 2008;4(3):191-4.

75. Green CF, Mottram DR, Rowe PH, Pirmohamed M. Attitudes and knowledge of hospital pharmacists to adverse drug reaction reporting. *Br J Clin Pharmacol* 2001;51(1):81-6.

76. Green CF, Mottram DR, Raval D, et al. Community pharmacists' attitudes to adverse drug reaction reporting. *Int J Pharm Pract* 1999;7(2):92-9.

77. Sweis D, Wong IC. A Survey on Factors that Could Affect Adverse Drug Reaction Reporting According to Hospital Pharmacists in Great Britain. *Drug Saf* 2000;23(2): 165-72.

78. McArdle D, Burns N, Ireland A. Attitudes and beliefs of doctors towards medication error reporting. *Int J Health Care Qual Assur* 2003;16(7):326-33.

For peer review only

For peer review only - http://bmjopen.bmj.com/site/about/guidelines.xhtml

79. Sanghera IS, Franklin BD, Dhillon S. The attitudes and beliefs of healthcare professionals on the causes and reporting of medication errors in a UK Intensive care unit. *Anaesthesia* 2007;62(1):53-61.

80. Belton KJ, Lewis SC, Payne S, et al. Attitudinal survey of adverse drug reaction reporting by medical practitioners in the United Kingdom. *Br J Clin Pharmacol* 1995;39(3):223-6.

81. Bateman DN, Sanders GL, Rawlins MD. Attitudes to adverse drug reaction reporting in the Northern Region. *Br J Clin Pharmacol* 1992;34(5):421-6.

82. Haines TP, Cornwell P, Fleming J, et al. Documentation of in-hospital falls on incident reports: qualitative investigation of an imperfect process. *BMC Health Serv Res* 2008;11;8:254.

83. Tariq A, Georgiou A, Westbrook J. Medication incident reporting in residential aged care facilities: limitations and risks to residents' safety. *BMC Geriatr* 2012;12:67.

84. Johnstone MJ, Kanitsaki O. Patient safety and the integration of graduate nurses into effective organizational clinical risk management systems and processes: an Australian study. *Qual Manag Health Care* 2008;17(2):162-73.

85. Walker SB, Lowe MJ. Nurses' views on reporting medication incidents. *Int J Nurs Pract* 1998;4(2):97-102.

86. Braithwaite J, Westbrook MT, Travaglia JF, et al. Cultural and associated enablers of, and barriers to, adverse incident reporting. *Qual Saf Health Care* 2010;19(3):229-33.

87. Heard GC, Sanderson PM, Thomas RD. Barriers to adverse event and error reporting in anesthesia. *Anesth Analg* 2012;114(3):604-14.

88. Hartnell N, MacKinnon N, Sketris I, et al. Identifying, understanding and overcoming barriers to medication error reporting in hospitals: A focus group study. *BMJ Qual Saf* 2012;21(5):361-8.

For peer review only - http://bmjopen.bmj.com/site/about/guidelines.xhtml

89. Walji R, Boon H, Barnes J, et al. Reporting natural health product related adverse drug reactions: Is it the pharmacist's responsibility? *Int J Pharm Pract* 2011;19(6):383-91.

90. Waters NF, Hall WA, Brown H, et al. Perceptions of Canadian labour and delivery nurses about incident reporting: A qualitative descriptive focus group study. *Int J Nurs Stud* 2012;49(7):811-21.

91. Espin S, Levinson W, Regehr G, et al. Error or "act of God"? A study of patients' and operating room team members' perceptions of error definition, reporting, and disclosure. *Surgery* 2006;139(1):6-14.

92. Covell CL, Ritchie JA. Nurses responses to medication errors: Suggestions for the development of organizational strategies to improve reporting. *J Nurs Care Qual* 2009;24(4): 287-97.

93. Merchant RN, Gully PM. A survey of British Columbia anesthesiologists on a provincial critical incident reporting program. *Can J Anaesth* 2005;52(7):680-4.

94. Espin S, Regehr G, Levinson W, et al. Factors Influencing Perioperative Nurses' Error Reporting Preferences. *AORN J*2007;85(3): 527-43.

95. Espin S, Wickson-Griffiths A, Wilson M, et al. To report or not to report: A descriptive study exploring ICU nurses' perceptions of error and error reporting. *Intensive Crit Care Nurs* 2010;26(1):1-9.

96. Chang IC, Hsu HM. Predicting medical staff intention to use an online reporting system with modified unified theory of acceptance and use of technology. *Telemed J E Health* 2012;18(1): 67-73.

97. Chiang HY, Lin SY, Hsu SC, et al. Factors determining hospital nurses' failures in reporting medication errors in Taiwan. *Nurs Outlook* 2010;58(1):17-25.

98. Chiang HY, Lin SY, Hsiao YC, et al. Culture influence and predictors for behavioral involvement in patient safety among hospital nurses in Taiwan. *J Nurs Care Qual* 2012;27(4):359-67.

For peer review only - http://bmjopen.bmj.com/site/about/guidelines.xhtml

99. Chiang HY, Pepper GA. Barriers to nurses' reporting of medication administration errors in Taiwan. *J Nurs Scholarsh* 2006;38(4):392-9.

100. Martowirono K, Jansma JD, van Luijk SJ, et al. Possible solutions for barriers in incident reporting by residents. *J Eval Clin Pract* 2012;18(1): 76-81.

101. Mes K, de Jong-van den Berg LTW, Van Grootheest AC. Attitudes of community pharmacists in the Netherlands towards adverse drug reaction reporting. *Int J Pharm Pract* 2002;10(4):267-72.

102. Zwart DL, Heddema WS, Vermeulen MI, et al.. Lessons learnt from incidents reported by postgraduate trainees in Dutch general practice. A prospective cohort study. *BMJ Qual Saf* 2011;20(10):857-62.

103. Eland IA, Belton KJ, van Grootheest AC, et al. Attitudinal survey of voluntary reporting of adverse drug reactions. *Br J Clin Pharmacol* 1999;48(4):623-7.

104. Alsafi E, Bahroon SA, Tamim H, et al. Physicians' attitudes toward reporting medical errors-an observational study at a general hospital in Saudi Arabia. *J Patient Saf* 2011;7(3):144-7.

105. Khan, TM. Community pharmacists' knowledge and perceptions about adverse drug reactions and barriers towards their reporting in eastern region, alahsa, Saudi Arabia. *Ther Adv Drug Saf* 2013;4(2):45-51.

106. Tobaiqy M, Stewart D. Exploring health professionals' experiences of medication errors in Saudi Arabia. *Int J Clin Pharm* 2013;35(4):542-5.

107. Bawazir SA. Attitude of community pharmacists in Saudi Arabia towards adverse drug reaction reporting. *Saudi Pharm J* 2006;14(1):75-83.

108. Davies EC, Chandler CI, Innocent SH, et al. Designing adverse event forms for real-world reporting: Participatory research in Uganda. *PLoS One* 2012;7(3):e32704.

109. Clark M, Gray M, Mooney J. New graduate occupational therapists' perceptions of near-misses and mistakes in the workplace. *Int J Health Care Qual Assur* 2013;26(6):564-76.

For peer review only - http://bmjopen.bmj.com/site/about/guidelines.xhtml

110. Kagan I, Barnoy S. Factors associated with reporting of medication errors by Israeli nurses. *J Nurs Care Qual* 2008;23(4):353-61.

111. Kagan I, Barnoy S. Organizational safety culture and medical error reporting by israeli nurses. *J Nurs Scholarsh* 2013;45(3):273-80.

112. Naveh E, Katz-Navon T, Stern Z. Readiness to report medical treatment errors: the effects of safety procedures, safety information, and priority of safety. *Med Care* 2006;44(2117-23.

113. Joolaee S, Hajibabaee F, Peyrovi H, et al. The relationship between incidence and report of medication errors and working conditions. *Int Nurs Rev* 2011 Mar;58(1):37-44.

114. Vessal G, Mardani Z, Mollai M. Knowledge, attitudes, and perceptions of pharmacists to adverse drug reaction reporting in Iran. *Pharm World Sci* 2009;31(2):183-7.

115. Okuyama A, Sasaki M, Kanda K. The relationship between incident reporting by nurses and safety management in hospitals. *Qual Manag Health Care* 2010;19(2):164-172.

116. Soleimani F. Learning from mistakes in New Zealand hospitals: what else do we need besides "no-fault"? *N Z Med J* 2006;119(1239):U2099.

117. Yong H, Kluger MT. Incident reporting in anaesthesia: A survey of practice in New Zealand. *Anaesth Intensive Care* 2003;31(5):555-9.

118. Söderberg J, Grankvist K, Brulin C, et al. Incident reporting practices in the preanalytical phase: Low reported frequencies in the primary health care setting. *Scand J Clin Lab Invest* 2009;69(7):731-5.

119. Bäckström M, Mjörndal T, Dahlqvist R, et al.. Attitudes to reporting adverse drug reactions in northern Sweden. *Eur J Clin Pharmacol* 2000;56(9-10):729-32.

120. Albolino S, Tartaglia R, Bellandi T, et al. Patient safety and incident reporting: survey of Italian healthcare workers. *Qual Saf Health Care* 2010;19 Suppl 3:i8-12.

121. Cosentino M, Leoni O, Banfi F, et al. Attitudes to adverse drug reaction reporting by medical practitioners in a Northern Italian district. *Pharmacol Res* 1997;35(2):85-8.

For peer review only - http://bmjopen.bmj.com/site/about/guidelines.xhtml

122. Rasmussen K, Pedersen AH, Pape L, et al. Work environment influences adverse events in an emergency department. *Dan Med J* 2014;61(5): A4812.

123. Ballangrud R, Hedelin B, Hall-Lord ML. Nurses' perceptions of patient safety climate in intensive care units: A cross-sectional study. *Intensive Crit Care Nurs* 2012;28(6): 344-54.

124. Mustafa GR, Rasheed S, Aziz MT. Adverse drug reaction reporting system at different hospitals of Lahore, Pakistan - An evaluation and patient outcome analysis. *J App Pharm* 2013;4(1):713-9.

125. Herdeiro MT, Figueiras A, Polónia J, et al.. Influence of Pharmacists' Attitudes on Adverse Drug Reaction Reporting. *Drug Saf* 2006;29(4): 331-40.

126. Mrayyan MT, Shishani K, Al-Faouri I. Rate, causes and reporting of medication errors in Jordan: nurses' perspectives. *J Nurs Manag* 2007;15(6):659-70.

127. Li Q, Zhang SM, Chen HT, et al. Awareness and attitudes of healthcare professionals in Wuhan, China to the reporting of adverse drug reactions. *Chin Med J (Engl)* 2004;117(6): 856-61.

128. Hasford J, Goettler M, Munter KH, et al. Physicians' knowledge and attitudes regarding the spontaneous reporting system for adverse drug reactions. *J Clin Epidemiol* 2002;55(9):945-50.

129. Irujo M, Beitia G, Bes-Rastrollo M, et al. Factors that Influence Under-Reporting of Suspected Adverse Drug Reactions among Community Pharmacists in a Spanish Region. *Drug Saf* 2007;30(11):1073-1082.

130. Nilsen P. Making sense of implementation theories, models and frameworks. *Implement Sci* 2015;10: 53.

For peer review only

For peer review only - http://bmjopen.bmj.com/site/about/guidelines.xhtml

For peer review only

For peer review only - http://bmjopen.bmj.com/site/about/guidelines.xhtml

Table 1: Search Strategy

Category A	Patient Safety Incident: near adj miss* (MeSH heading), adverse adj event*, never adj event* (MeSH entry term), medical adj mistake* (MeSH entry term), error*, mistake* (MeSH entry term), negligen* (MeSH entry term), malpractice* (MeSH heading), failure*, injur* (MeSH entry term), critical adj incident* (MeSH entry term), sentinel adj event*, incident*, harm*, accident* (MeSH heading), medical adj error* (MeSH heading), patient adj safety (MeSH heading)
Category B	Incident Reporting System: risk adj management (MeSH heading), incident adj reporting adj system*, error adj report*, critical adj incident adj technique (MeSH entry term), safety adj report*, incident adj report* (MeSH entry term), reporting adj system, NRLS, national adj reporting adj2 learning adj system.
Category C	Barrier/Facilitator: communication adj barrier* (MeSH heading), feedback (MeSH heading), safety adj culture (MeSH entry term), reporting adj culture, attitude (MeSH heading)*, preventive adj measure* (MeSH entry term), mandatory, voluntary, under-reporting, willingness, blame, obstacle*, incident adj type, level adj of adj harm, fear* (MeSH heading), responsibi*, workload (MeSH heading), trust* (MeSH heading), anonym*, confidential* (MeSH heading), facilit*, barrier*, enabl*, legal, law (MeSH entry term).

For peer review only

Table 2: Theoretical framework of factors determining engagement in patient safety incident reporting

Category	Descriptions & Examples
**Organisational**	Organisational values, beliefs and policies around incident reporting. This also encompasses any organisational factor which may act as a barrier or facilitator to reporting behavior, such as structure (e.g. size of hospital) and organisational culture.
**Work Environment**	Features of the work environment that act as barriers or facilitators to engagement in incident reporting. Examples of such factors include level of activity, staffing levels and visual prompts.
**Process and systems of Reporting**	Any characteristics or features of the reporting system/process which enables or hinders incident reporting. This includes the complexity of the reporting system, the level of information required and the mode of incident reporting (e.g. paper based or electronic).
**Team factors**	Any factor related to the functioning of different professionals within a group which influences incident reporting behavior. For example, support and encouragement by team members to report incidents, and levels of teamwork and communication.
**Knowledge and Skills**	The acquisition and development of knowledge and skills that enables incident reporting. This includes participation in specific (e.g. form completion) and general (e.g. identifying which incidents warrant reporting) training/educational activities.
**Individual HCP Characteristics**	Characteristics of the HCP that may contribute in some way to engagement in incident reporting. Examples of such factors include seniority, personality and attitudes.
**Professional Ethics**	The accepted standards of personal and professional behavior, values and guiding principles that promote incident reporting. For example, the adoption of sound and consistent ethical practices, such as duty of care.
**Fear of adverse consequences**	Any unpleasant emotion (e.g. guilt) or outcome (e.g. litigation) associated with individual HCPs' incident reporting behavior. A reduction in the likelihood of experiencing fear (e.g. the existence of a non-punitive policy) results in increased incident reporting participation.
**Incident Characteristics**	Characteristics of the patient safety incident which may make HCP's more or less likely to report. These include frequency of error, level of harm and the cause of error.

Note: HCP=Healthcare Professional

For peer review only - http://bmjopen.bmj.com/site/about/guidelines.xhtml

Table 3: Frequency of Articles by Country

Country	Count *(percentage)*
United States of America[9, 11, 28, 30-59]	33 *(30.00 %)*
United Kingdom[10, 29, 60-81]	24 *(21.82 %)*
Australia[8, 27, 82-87]	8 *(7.27%)*
Canada[88-95]	8 *(7.27 %)*
Taiwan[96-99]	4 *(3.64 %)*
Netherlands[100-103]	4 *(3.64 %)*
Saudi Arabia[104-107]	4 *(3.64 %)*
International[24, 26, 108, 109]	4 *(3.64 %)*
Israel[110-112]	3 *(2.73 %)*
Iran[113, 114]	2 *(1.82 %)*
Japan[25, 115]	2 *(1.82 %)*
New Zealand[116, 117]	2 *(1.82 %)*
Sweden[118, 119]	2 *(1.82 %)*
Italy[120, 121]	2 *(1.82 %)*
Denmark[122]	1 (0.91 *%)*
Norway[123]	1 (0.91 *%)*
Pakistan[124]	1 (0.91 *%)*
Portugal[125]	1 (0.91 *%)*
Jordan[126]	1 (0.91 *%)*
China[127]	1 (0.91 *%)*
Germany[128]	1 (0.91 *%)*
Spain[129]	1 (0.91 *%)*

Figure 1: Example of data coding, conceptualisation and categorisation for theory development

Figure 2: Flow diagram of the theoretical literature review process

Figure 3: Frequency of categories influencing engagement in patient safety incident reporting

For peer review only - http://bmjopen.bmj.com/site/about/guidelines.xhtml

	**Stage 1: Coding (open coding)** *Identification of data that contributes to engagement in incident reporting*	→	**Stage 2: Conceptualising (axial coding)** *Identification of codes of similar content grouped and named*	→	**Stage 3: Categorising (selective coding)** *Identification of similar concepts grouped into broad groups*
Paper 1: "Despite some initial success with this approach, [implementation of incident reporting system] it was stated that the **time** and **resource** demands of managing the process were too great".	Not enough time<br>Not enough resources	→	Time<br>Resources	→	Process and system of reporting
Paper 2: "The concept of a **blame culture** appeared to exist".	People are scared of being blamed	→	Fear of blame	→	Fear of adverse consequences

Figure 1: Example of data coding, conceptualisation and categorisation for theory development

57x46mm (300 x 300 DPI)

w only

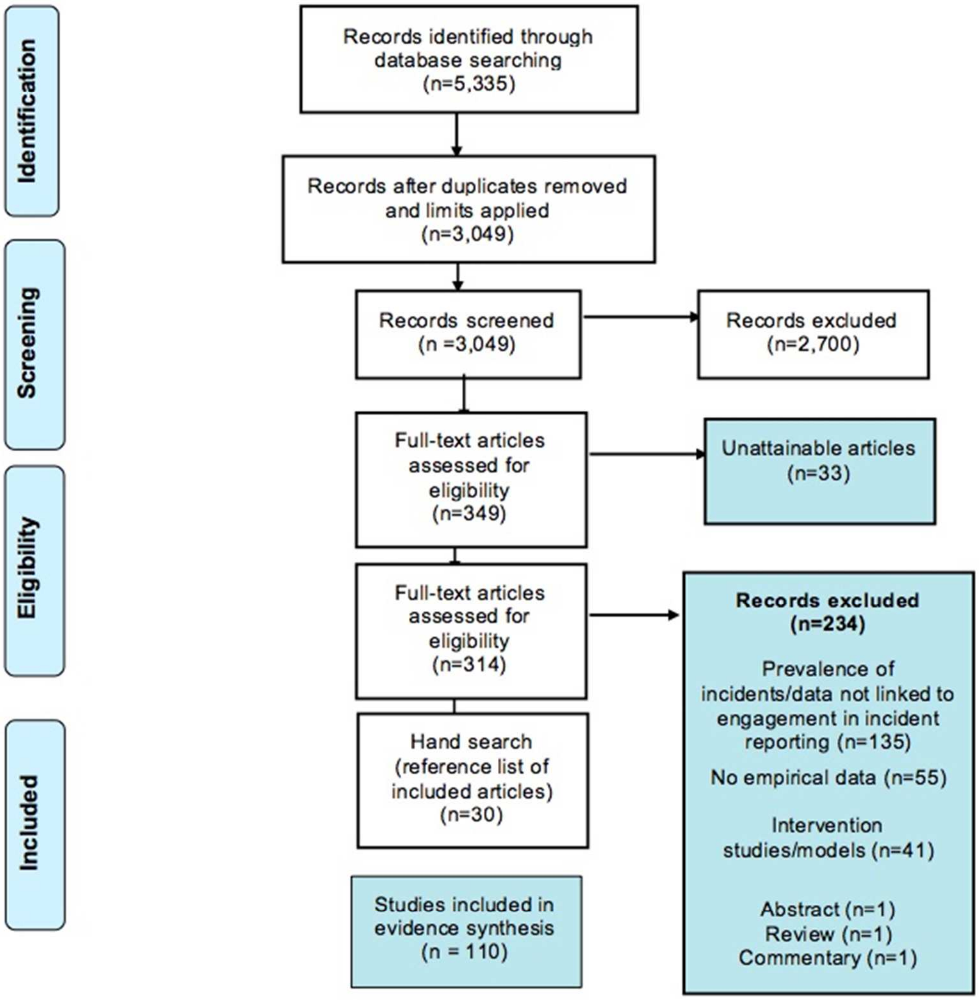

Figure 2: Flow diagram of the theoretical literature review process

54x55mm (300 x 300 DPI)

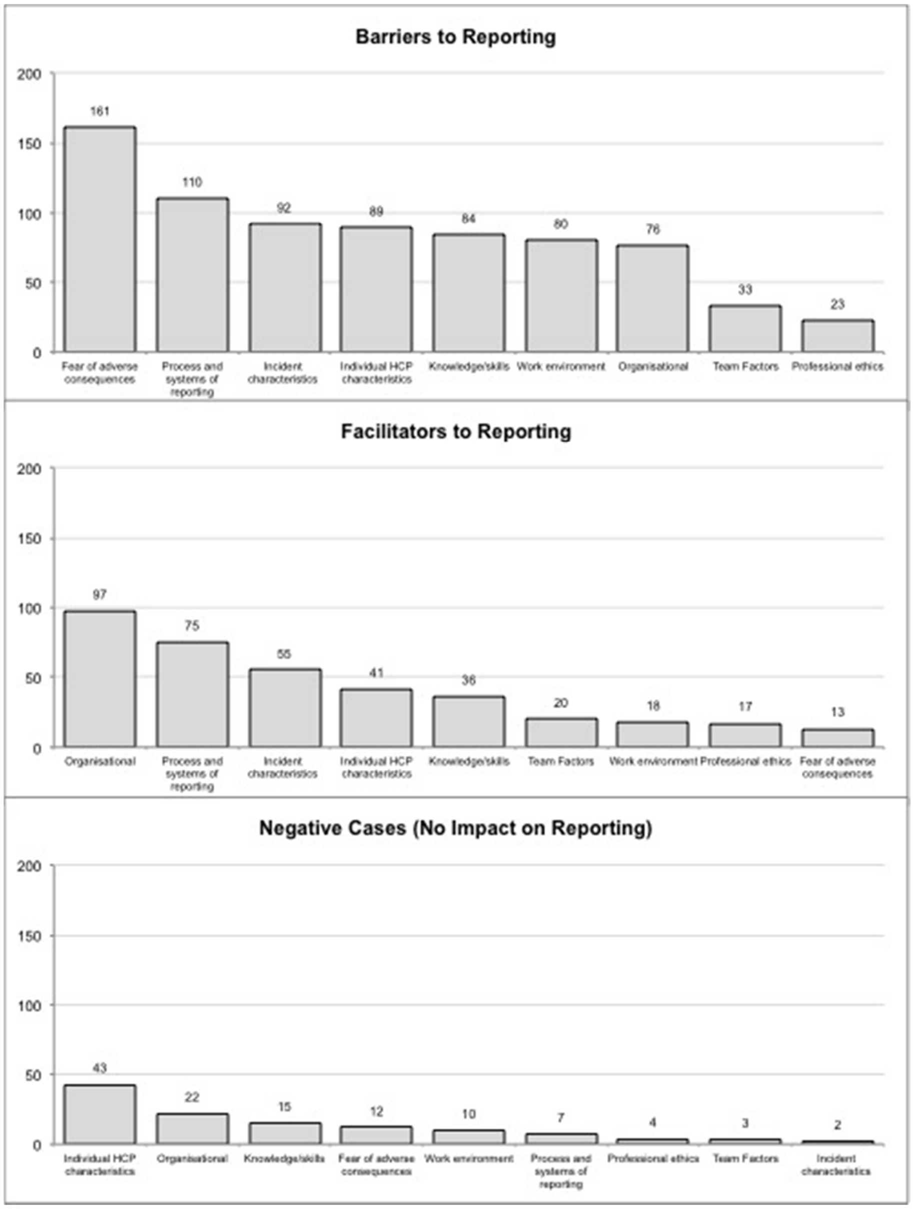

Figure 3: Frequency of categories influencing engagement in patient safety incident reporting

42x55mm (300 x 300 DPI)

eTable1: Full data extraction table of included articles

Author, Year	Study Design, Sample Size, Country	Barriers to Incident Reporting	Facilitators of Incident Reporting	Negative cases (No impact)
Albolino et al., 2010 [120]	Questionnaire based-study<br>820<br>Italy	Fear of mistrust in colleagues<br><br>Not considered a priority<br><br>Fear of punishment<br><br>Does not help to improve safety<br><br>Lack of time		
Alsafi et al., 2011 [104]	Questionnaire based-study.<br>107<br>Saudi Arabia	Not my responsibility<br><br>I do not want to lose my good relationship with my colleague<br><br>I might be reported by my colleague in turn<br><br>No incentive to error disclose<br><br>Avoiding punishment<br><br>Avoiding damage to reputation<br><br>It will not be discovered		
Anderson et al., 2013 [60]	Semi-structured interviews and documentary analysis	Experienced in using IR systems (Mental health staff)		

For peer review only - http://bmjopen.bmj.com/site/about/guidelines.xhtml

	62<br>United Kingdom	Blame culture (mental health staff)		
Arfanis et al., 2012 [61]	Semi-structured interviews<br>48<br>United Kingdom	Not used as learning tools to prevent similar occurrences elsewhere.<br><br>Pressures on time<br><br>Resources<br><br>A lack of faith in the established system<br><br>Fruitless and often pointless exercise that has little or no impact on improving patient safety and welfare<br><br>Fear of litigation<br><br>Fear of disciplinary action<br><br>Blame<br><br>The availability and ease of identifying the information<br><br>No feedback	Feedback<br><br>Learning and improvement<br><br>Anonymous web based forum as an add on to IR system	
Armitage et al., 2010 [62]	Semi-structured interviews and retrospective review of error reports<br>40	Lack of feedback		

For peer review only - http://bmjopen.bmj.com/site/about/guidelines.xhtml

	United Kingdom			
Ashcroft et al., 2006 [66]	Questionnaire-based Study<br>275<br>United Kingdom	Local reporting<br><br>Good patient outcome less likely to be reported than poor or bad patient outcome.<br><br>Compliance with a protocol less likely to be reported than a violation or error.<br><br>'Fault-led' attitude<br><br>One-off situations by individuals not report<br><br>Loyalty to colleagues<br><br>National reporting system<br><br>Confidence in National Patient Safety Agency	Local reporting<br><br>Poor or bad patient outcome more likely to be reported than good patient outcome<br><br>Violation of protocol or error more likely to be reported than compliance with protocol.<br><br>'Learn from mistakes' culture<br><br>Individuals making continual mistakes<br><br>National reporting system	
Backstrom et al., 2000 [119]	Questionnaire-based study.<br>748<br>Sweden	Assessment that the reaction is already well known<br><br>Forgetting to report		

For peer review only

		Hesitance to report on suspicion<br><br>Lack of time<br><br>Giving preference to other matters<br><br>Uncertainty about the existing rules for reporting<br><br>Difficulty in finding the right form		
Ballangrud et al., 2012 [123]	Questionnaire-based study.<br>220<br>Norway	Supervisor/manager expectations, actions promoting safety<br><br>Feedback and communication about error		Organisational learning and continuous improvement<br><br>Teamwork within hospital units<br><br>Communication openness<br><br>Non punitive response to errors<br><br>Staffing
Bateman et al., 1992 [81]	Questionnaire-based study.<br>1181	One case cannot contribute to medical knowledge	Should be financially	

For peer review only - http://bmjopen.bmj.com/site/about/guidelines.xhtml

	United Kingdom	Impossible to determine responsible drug<br><br>Serious ADRs well known when the drug is marketed<br><br>Professional obligation<br><br>Reporting increases personal liability<br><br>Reporting results by badgering by Committee of safety of medicines<br><br>Takes too much time to ADR report	reimbursed<br><br>Would report if easier method	
Bawazir et al., 2006 [107]	Questionnaire-based study.<br>172<br>Saudi Arabia	No reporting forms available<br><br>Reporting address unknown<br><br>Reporting form too complicated<br><br>Reporting ADRs is too time consuming<br><br>All ADRs are known<br><br>Want to publish myself<br><br>Confidentiality<br><br>Patient confidence	An obligation to do so<br><br>There was a fee<br><br>Saw colleagues doing so<br><br>Attention drawn by publication<br><br>Receiving feedback<br><br>Report through the internet	

For peer review only - http://bmjopen.bmj.com/site/about/guidelines.xhtml

		Difficult to admit harm to patient<br><br>Reporting could show ignorance<br><br>Fear of liability<br><br>No motivation<br><br>Insufficient clinical knowledge<br><br>Do not know how to report<br><br>Causality uncertain<br><br>One report make no difference		
Beasley et al., 2004 [30]	Focus groups<br>14<br>United States of America	Punitive system	A feedback system for submitters is necessary to maintain interest.<br><br>Safe and secure access<br><br>There needs to be easy access<br><br>What to report needs to be clearly defined<br><br>The reporting forms	

For peer review only - http://bmjopen.bmj.com/site/about/guidelines.xhtml
For peer review only

			must be simple<br><br>Error reporting must fit into a clinicians current work flow<br><br>A non-punitive system is essential<br><br>Reporter should only be required to report once if there are multiple systems	
Belton et al., 1995 [80]	Questionnaire-based study<br>284<br>United Kingdom	Report forms are not available when needed<br><br>Doctor does not like reporting confidential information<br><br>Doctor unsure how to report an ADR<br><br>Doctor fear he/she may appear foolish about reporting a suspected reaction<br><br>Doctor fears he/she may be exposed to legal liability by reporting reaction<br><br>Doctor too busy to send an ADR		

For peer review only - http://bmjopen.bmj.com/site/about/guidelines.xhtml

		report<br><br>Doctor is reluctant to admit he/she may have caused a patient harm<br><br>Doctor would rather collect and publish personally<br><br>Doctor believe that only safe drugs are marketed		
Belton et al., 1997 [24]	Questionnaire-based study<br>Sample size not reported<br>International: Denmark, France, Ireland, Italy, Netherlands, Portugal, Spain, Sweden, United Kingdom	Telephone number unavailable<br><br>Report forms unavailable<br><br>Address of reporting agency unavailable<br><br>Unsure how to report<br><br>Patient confidentiality<br><br>Worried about appearing foolish<br><br>Worried about legal liability (Not Denmark or Spain)<br><br>Too busy to report ADRs<br><br>Reluctant to admit they have caused a patient harm		Worried about legal liability (Not Denmark or Spain)<br><br>Ambition to publish a personal series of cases (Not Spain, Sweden or Portugal)<br><br>Patient confidentiality (Not Spain)

For peer review only - http://bmjopen.bmj.com/site/about/guidelines.xhtml

		Ambition to publish a personal series of cases (Not Spain, Sweden or Portugal)<br><br>Believes that all marketed drugs are safe		
Blegen et al., 2004 [55]	Questionnaire-based study<br>1105<br>United States of America	Administrative response<br><br>Personal fear<br><br>Quality management<br><br>Staffing resources<br><br>Physical resources<br><br>Peer relations<br><br>Job satisfaction		
Braithwaite et al., 2010 [86]	Questionnaire-based study.<br>2185<br>Australia	IIMS training<br><br>Accessibility of reporting system<br><br>Security of IIMS<br><br>Feedback from reports<br><br>Workplace reporting culture<br><br>Value placed on IIMS		Form of training received

For peer review only - http://bmjopen.bmj.com/site/about/guidelines.xhtml

Chang et al., 2012 [96]	Questionnaire-based study<br>183<br>Taiwan		Level of support	Age
Chiang et al., 2006 [99]	Questionnaire-based study.<br>597<br>Taiwan	Being blamed for MAE results<br><br>Adverse consequences from reporting<br><br>Patient's negative attitude<br><br>Physicians' reprimand<br><br>Not recognised MAEs occurred<br><br>Being recognised as incompetent<br><br>Too much time for filling reports<br><br>Think MAEs not important enough to be reported<br><br>Too much time for contacting physicians<br><br>Unclear MAE definition<br><br>Disagreement over MAE<br><br>Unrealistic expectation for administering drugs correctly		

For peer review only - http://bmjopen.bmj.com/site/about/guidelines.xhtml

		No positive feedback<br><br>Much emphasis on MAE as nursing quality provided<br><br>Focus on individual rather than system factors to MAEs<br><br>Administrators' responses to MAEs do not match the severity of the errors		
Chiang et al., 2010 [97]	Questionnaire-based study<br>838<br>Taiwan	Experience of making MAEs<br><br>Nursing professional development<br><br>Fear	Same attitude towards self and co-workers<br><br>MAE reporting rate<br><br>Nursing quality	Age<br><br>Management and leadership<br><br>Administrative barriers<br><br>Reporting process
Chiang et al., 2012 [98]	Questionnaire-based study<br>1049<br>Taiwan		High scores on the safety organising scale<br><br>Tenure of present position<br><br>Self-evaluated IR rates	

For peer review only - http://bmjopen.bmj.com/site/about/guidelines.xhtml

			Those more willing to report their own incidents are more likely to report co-workers incidents	
Church et al., 2013 [36]	Questionnaire-based study<br>546<br>United States of America	Hierarchical structure<br><br>Poor communication<br><br>Fear of reprimand<br><br>Reprimand of other therapists and dosimetrists<br><br>Personality<br><br>Lack of reporting system		
Clark et al., 2013 [109]	Questionnaire-based study<br>228<br>International: Australia and New Zealand	Fear of being judged by colleagues<br><br>Personal Guilt<br><br>Feel it as unnecessary<br><br>Near misses are part of life		
Coley et al., 2006 [57]	Focus groups<br>8<br>United States of America	Time consuming<br><br>Inadequate staffing		

For peer review only - http://bmjopen.bmj.com/site/about/guidelines.xhtml

Cosentino et al., 1997 [121]	Questionnaire-based study<br>207<br>Italy	Reaction not clinically relevant<br><br>Awareness of similar reactions<br><br>Unavailability of report forms<br><br>Doubtfulness about which ADRs should be reported<br><br>Confidence about ADRs being well documented before marketing<br><br>Ignorance about reporting procedures<br><br>Too much time required to fill in the report form<br><br>Don't feel obliged to report<br><br>Don't want to create undue alarm<br><br>Uselessness of ADR spontaneous reporting		
Covell et al., 2009 [92]	Semi-structured interviews and questionnaire based study<br>50<br>Canada	Adverse consequences		
Daly et al., 2005 [37]	Questionnaire-based study<br>598	Administrators' length of time in position	Directors of nursings'	Administrators' knowledge of

For peer review only - http://bmjopen.bmj.com/site/about/guidelines.xhtml

	United States of America	Administrators' and Directors' length of time in facility<br><br>Administrators' length of time in profession<br><br>After internal investigation abuse was thought not to exist<br><br>Told not to report the abuse by my boss<br><br>Reported abuse in the past and IDIA did nothing<br><br>Reported abuse in the past and it led to a bad outcome<br><br>Reported abuse in the past and IDIA ruled it out	knowledge of the law in of nursing<br><br>Administrators' level of education	law<br><br>Administrators' belief that 'elders are able to get help if they need it'<br><br>Age of administrators and directors of nursing<br>Director of nursings' length of time in position<br><br>Director of nursings' length of time in profession<br><br>Director of nursings' level of education<br><br>Administrators' knowledge of the law in nursing
Davies et al., 2012	Focus groups	Lack of feedback		

For peer review only - http://bmjopen.bmj.com/site/about/guidelines.xhtml

[108]	19<br>International: United Kingdom/Uganda			
Ehrenpreis et al., 2012 [38]	Questionnaire-based study<br>92<br>United States of America	Unsure how to report appropriately<br><br>Did not see adverse events on a regular basis<br><br>Too busy to make reports<br><br>The existing method was too cumbersome<br><br>Voluntary reporting was not an important process	Easier to use	
Eland et al., 1999 [103]	Questionnaire-based study<br>1357<br>Netherlands	Uncertain association<br><br>Too trivial to report<br><br>Too well known to report<br><br>Unaware of the existence of a nation ADR reporting system<br><br>Unaware of the need to report ADRs<br><br>Did not know how to report ADRs<br><br>Too bureaucratic<br><br>Not enough time		

For peer review only - http://bmjopen.bmj.com/site/about/guidelines.xhtml

		Concerned that the report could be used in legal case for damages by the patient<br><br>If another physician had prescribed the medicine<br><br>Medication brought over counter rather than prescribed		
Elder et al., 2007 [31]	Focus groups<br>139<br>United States of America	Burden of effort<br><br>Lack of time<br><br>Forgetfulness<br><br>Information not readily available<br><br>Computer problems<br><br>Online access<br><br>What to report<br><br>Who should report<br><br>What is an AE<br><br>What information is needed<br><br>Common problems	Perceived benefit of reporting – learning and improvement<br><br>Emotional benefit<br><br>Guilt<br><br>Personal responsibility<br><br>Anonymous reporting<br><br>Easing the burden of reporting<br><br>The more harm, the more likely to report	

For peer review only - http://bmjopen.bmj.com/site/about/guidelines.xhtml

		Rare errors<br><br>Less serious errors unlikely to be reported<br><br>Feeling personally responsible		
Elder et al., 2008 [58]	Focus groups and questionnaire-based study<br>125<br>United States of America	Too busy with other activities<br><br>Didn't reach the patient<br><br>Risk of harm is none or little<br><br>Error made my someone new-give them a break<br><br>Feel worse emotionally<br><br>Feel like a failure<br><br>Fear punishment<br><br>Blame<br><br>Name on permanent record<br><br>Risk losing friends<br><br>Will make enemies on unit<br><br>No feedback so no personal benefits	Asked by management to make specific reports<br><br>Harm actually occurred<br><br>Risk of harm is great<br><br>Error made by someone unable to be spoken to one-to-one<br><br>Feel better emotionally<br><br>Outlet for irritation at situation or person<br><br>Honesty is a virtue	

For peer review only - http://bmjopen.bmj.com/site/about/guidelines.xhtml

			Get a “there but for the grace of god” understanding<br><br>Improve clinical practice<br><br>Could be a learning experience for others<br><br>No known penalty for making a report	
Erler et al., 2013 [39]	Questionnaire-based study<br>51<br>United States of America		Higher levels of teamwork<br><br>Communication openness<br><br>Perception of manager actions promoting safety	
Espin et al., 2010 [95]	Semi-structured interviews<br>37<br>Canada	Did not feel it was an error	Patient negligence<br><br>Threat of potential or actual harm to the patient<br><br>Patient advocacy	

For peer review only - http://bmjopen.bmj.com/site/about/guidelines.xhtml

			Following proper procedure<br><br>Error prevention<br><br>Learning opportunities	
Espin, et al., 2007 [94]	Semi-structured interviews<br>13<br>Canada	Domain-specific expertise is a necessary pre-requisite for reporting the error<br><br>Part of the surgeon's responsibility as it fell within the surgical scope of practice.	Events outside of professional boundaries were more likely to be reported<br><br>Responsible for error	
Espin et al., 2006 [91]	Semi-structured and structured interviews<br>28<br>Canada	Responsibility		
Evans et al., 2006[8]	Questionnaire-based study<br>773<br>Australia	I never get any feedback on what action is taken<br><br>I don't feel confident it is kept anonymous<br><br>The incident form takes too long to fill out and I just don't have time<br><br>I am worried about litigation		

For peer review only - http://bmjopen.bmj.com/site/about/guidelines.xhtml

		The incident was too trivial<br><br>When the ward is busy I forget to make a report<br><br>It's not my responsibility to report someone else's mistakes<br><br>I don’t know whose responsibility it is to make a report<br><br>I don't want to get into trouble<br><br>When it is a near miss, I don't see any point in reporting it<br><br>Even if I don;t give my details, I am sure that they'll track me down<br><br>The AIMS+ form is too complicated and requires too much detail<br><br>Junior staff are often blamed unfairly for adverse incidents<br><br>I wonder about who else is privy to the information that I disclose<br><br>If I discuss the case with the person involved nothing else needs to be done		

For peer review only - http://bmjopen.bmj.com/site/about/guidelines.xhtml

		I don't want the case discussed in meetings<br><br>I am worried about disciplinary action<br><br>Adverse incident reporting is unlikely to lead to system changes<br><br>My co-workers may be unsupportive		
Fairbanks et al., 2008 [32]	Interviews, focus groups and events reports from an anonymous system<br>15<br>United States of America	Blame and Shame<br><br>Punishment<br><br>Legal factors<br><br>Reluctance to tell on colleagues	Non punitive system	
Fukuda et al., 2010 [25]	Questionnaire-based study<br>Sample size not stated<br>Japan		Decreased time for reporting (nurses and physicians)<br><br>Electronic reporting (physicians)<br><br>Attendance at educational seminars (physicians)<br><br>Hospital size	Non-punitive policy (physicians/nurses)<br><br>Rate of recommendations derived from reported incidents (physicians/nurses)<br><br>Electronic

For peer review only - http://bmjopen.bmj.com/site/about/guidelines.xhtml

			Ownership – university hospital (physicians)<br><br>Ownership – national hospital (nurses)<br><br>Assignment of patient safety manager (physicians)	reporting (nurses)<br><br>Attendance at educational seminars (nurses)<br><br>Elapsed years of incident reporting system (physicians and nurses)<br><br>Attendance at conference (Physicians/nurses)<br><br>Ward rounds (Physicians/nurses)<br><br>Ownership – university hospital (nurses)<br><br>Ownership – national

For peer review only

For peer review only - http://bmjopen.bmj.com/site/about/guidelines.xhtml

				hospital (physicians)<br><br>Ownership – municipal + public hospitals + healthcare corporation + other (physicians/nurse)<br><br>Assignment of patient safety manager (nurses)
Gaal et al., 2010 [26]	Observational study<br>Sample size not stated<br>International: Austria, Belgium, England, France, Germany, Israel, The Netherlands, Slovenia, Switzerland, and Wales		Group (>3) practice	Practice setting<br><br>Amount of responsibility<br><br>Hours of work<br><br>Physical working conditions<br><br>Single+ dual practice
Garbutt et al., 2007	Questionnaire-based study	Private practice	Belief that errors	Perceived risk

For peer review only

[40]	557<br>United States of America		are one of the most serious issues in healthcare<br><br>Belief that they should report serious errors<br><br>Belief that they should report minor errors<br><br>Belief that they should report near misses<br><br>System change to improve patient safety after errors reported<br><br>If error was caused by system rather than individual failures<br><br>Personal involvement in serious errors<br><br>Assurance that the information was	for personal malpractice risk<br><br>Personal involvement in an error

For peer review only - http://bmjopen.bmj.com/site/about/guidelines.xhtml

			confidential<br><br>A non-punitive reporting system<br><br>A process that takes less than 2 minutes to use<br><br>Local to the clinician's unit or department	
Generali et al., 1995 [52]	Questionnaire-based study 235<br>United States of America	Unsure drug caused reaction<br><br>Do not have forms<br><br>Do not know how<br><br>Reaction was expected<br><br>Reporting would not occur to me<br><br>Fear of legal liability<br><br>Not my responsibility<br><br>Hours worked per week (>49 or <40)	Hours worked per week (43-49 hours)<br><br>Work setting	Age<br><br>Gender<br><br>Number of years in practice
Gladstone, 1995 [67]	Questionnaires and semi-structured interviews 107<br>United Kingdom	Fear of management reaction		

For peer review only - http://bmjopen.bmj.com/site/about/guidelines.xhtml

Green et al., 1999 [76]	Structured interview<br>30<br>United Kingdom	Lack of time/too busy<br><br>Well recognised reaction<br><br>Limited time to spend with patients<br><br>Lack of motivation<br><br>More information about ADR needed<br><br>Lack of confidence in making report<br><br>Patient confidentiality<br><br>Patient suffered an ADR to a product counter prescribed by the pharmacists being interviewed	Certainty of ADR<br><br>Suspicious of a reaction<br><br>Training<br><br>Fee for reporting<br><br>Access to patient records<br><br>Feedback<br><br>More time	
Green et al., 2001 [75]	Questionnaire-based study<br>322<br>United Kingdom	Concern that a doctor gets a copy of reporting form<br><br>Lack of confidence in discussing the ADR with the prescriber<br><br>Apprehension about sending in an inappropriate report<br><br>Lack of time to fill in a report<br><br>Concern that a report will generate extra work	Reaction is of a serious nature<br><br>The reaction is unusual<br><br>The reaction is to a new product<br><br>Certainty that the reaction is a ADR<br><br>The reaction is well	

For peer review only - http://bmjopen.bmj.com/site/about/guidelines.xhtml

		The absence of a fee for reporting ADRs<br><br>Lack of time to actively look for ADRs while in clinical practice<br><br>Lack of clinical knowledge makes it difficult to decide whether or not an ADR has occurred<br><br>Don't feel the need to report well recognised reactions<br><br>Reporting cards not available when needed	recognised for a particular agent<br><br>Education/training/ study days or evenings<br><br>More time to spend on wards with patients<br><br>More feedback, reminders and increased awareness<br><br>Encouragement from managers and departments<br><br>Increased collaboration with prescribers and participation on ward round<br><br>Increased accessibility of reporting cards<br><br>Cards specifically designed for the	

For peer review only - http://bmjopen.bmj.com/site/about/guidelines.xhtml

			use of pharmacists<br><br>More publicity in journal about reporting scheme<br><br>Online access or telephone based reporting<br><br>Development of local incentives<br><br>Increased confidence in dealing with medical staff<br><br>Making reporting a professional responsibility<br><br>A fee for reporting<br><br>ADR specialist pharmacists<br><br>Increasing awareness among other professionals that pharmacists could report ADRs	

For peer review only - http://bmjopen.bmj.com/site/about/guidelines.xhtml

van Grootheest et al., 2002 [101]	Questionnaire-based study<br>147<br>Netherlands	Causality uncertain<br><br>Too time-consuming<br><br>No reporting forms available<br><br>Reporting address unknown<br><br>Reporting form too complicated<br><br>All adverse reactions are known<br><br>Want to publish myself<br><br>Confidentiality<br><br>Fear of liability<br><br>No motivation<br><br>Insufficient clinical knowledge<br><br>Do not know how to report	Feedback<br><br>Publications<br><br>Information about the national centre<br><br>Simplification of reporting procedure<br><br>Promoting reporting as part of professional duty<br><br>Financial compensation<br><br>More attention to ADR reporting in university curriculum<br><br>Database of national centre available on the internet<br><br>Compulsory reporting<br><br>Peer reporting	Reporting could show ignorance

For peer review only

Haines et al., 2008 [82]	Questionnaire-based study<br>212<br>Australia	Time<br><br>If the ward is very busy<br><br>Patients' responsibility for adverse events<br><br>Cause of the incident<br><br>Other methods of documentation<br><br>Access to previous reports (non filing of incident reports in the notes)<br><br>Poor user friendliness of computer reporter systems<br><br>Made staff feel personally responsible for the form<br><br>Poor access to computers<br><br>Non reporting by role models<br><br>Absence of a definition of a fall<br><br>Blame<br><br>Absence of training	Staff believe that completing IRs improves patient safety<br><br>Staff belief that competing IRs protects against legal liability<br><br>If the patients was harmed/injured<br><br>Patient factors<br><br>Protect staff<br><br>Type of incident - preventable	

For peer review only

Handler et al., 2007 [35]	Focus group and questionnaire-based study<br>132<br>United States of America	Lack of readily available medication error reporting system or forms<br><br>Lack of information on how to report a medication error<br><br>Lack of feedback to the reporter or rest of facility on medication errors that have been reported<br><br>Lack of knowledge of which medication errors should be reported<br><br>Systems or forms used to report medication error are long and time consuming<br><br>Lack of knowledge of the usefulness of reporting medication errors<br><br>Lack of a consistent definition of a medication error<br><br>Lack of an anonymous medication error reporting system<br><br>Lack of recognition that a medication error has occurred<br><br>Lack of a culture of reporting medication errors		

For peer review only

		Extra time involved in documenting a medication error<br><br>Fear of disciplinary action<br><br>Fear of being blamed<br><br>Fear of liability or lawsuits<br><br>Not knowing who is responsible for reporting a medication error<br><br>Belief that it is unnecessary to report medication errors not associated with patient harm<br><br>Lack of recognition of the actual or potential harm of a medication error<br><br>Belief that reporting medication errors has little contribution to improving the quality of care<br><br>Difficulty in proving that a medication error actually occurred<br><br>Fear of losing respect of co-workers		
Hartnell et al., 2012 [88]	Focus group and semi-structured interviews<br>30<br>Canada	Extra time required to report<br><br>Extra work required to report	Improved care/improved patient safety	

For peer review only - http://bmjopen.bmj.com/site/about/guidelines.xhtml

		Cumbersome IR forms<br><br>Hesitancy about 'telling on' someone else<br><br>Fear of loss of reputation/perceived incompetence<br><br>Perceived severity of error (less severe errors are less likely to be reported)<br><br>Inability to recognise or identify medication errors<br><br>Lack of definitions or standards for reporting<br><br>Lack of belief that reporting makes a difference<br><br>lack of trust about how error reports will be used<br><br>Reporting is the responsibility of someone else<br><br>Fear of reprisal from management/administration<br><br>Fear of exposure to malpractice suits	To prevent patient from receiving wrong medication<br><br>Provides immunity/protection from legal action<br><br>Fear of censure (harsh criticism or blame)<br><br>Perceived severity of error (more severe errors are more likely to be reported because a report will be expected)<br><br>Follow rules or policies<br><br>Ensures accountability	

For peer review only - http://bmjopen.bmj.com/site/about/guidelines.xhtml

Hasford et al., 2002 [128]	Questionnaire-based study<br>588<br>Germany	ADR too well known<br><br>ADR too trivial<br><br>Uncertain causality<br><br>Reporting too bureaucratic<br><br>Lack of time<br><br>Rules of conduct unknown<br><br>Suspect that drug prescribed by colleague<br><br>Reporting process unknown<br><br>Lack of financial reimbursement<br><br>Suspect drug was self-medication<br><br>Reports considered useless<br><br>Reporting system unknown<br><br>Fear of legal liability<br><br>Non-serious adverse reaction to established drug	Serious unknown ADR to a new drug<br><br>Serious unknown ADR to an established drug<br><br>Serious known ADR to a new drug	
Heard et al., 2012 [87]	Questionnaire-based study<br>433	I am worried about litigation		Generalised de-identified

For peer review only - http://bmjopen.bmj.com/site/about/guidelines.xhtml

	Australia	I don't want to get into trouble<br><br>My colleagues may be unsupportive<br><br>I am worried about disciplinary action<br><br>I may be blamed unfairly for the event<br><br>I do not want to be discussed in meetings.<br><br>Adverse events reporting makes little contribution to quality care<br><br>I don't know whose responsibility it is to make a report<br><br>A good outcome of the case makes reporting unnecessary<br><br>I do not know which adverse events should be reported.<br><br>Even if I don't give my details I'm worried they will track me down<br><br>The forms take too long to fill in and just don't have time<br><br>When I am busy I forget to make a report		feedback about reports received from the anaesthetic community<br><br>Role models e.g. senior colleagues and department directors who openly encourage reporting<br><br>Legislated protection of information you provide from use in litigation<br><br>Ability to report anonymously<br><br>Clear guidelines about what adverse events are errors to report<br><br>Information on

For peer review only - http://bmjopen.bmj.com/site/about/guidelines.xhtml

		I don't feel confident that they information I provide will be kept confidential<br><br>I never get any feedback after I report an adverse event<br><br>I wonder about who else will have access to information I disclose<br><br>As long as the staff involved learn from incidents it is unnecessary to discuss them further<br><br>I would protect my self-interests ahead of the interests of the patient if I could (by hiding or denying error)<br><br>Competition with my peers could prevent me from disclosing an error<br><br>If a doctor is careful enough he or she will not make an error<br><br>It would affect my identity as a doctor to admit to an error<br><br>Other don't need to know about errors I have made<br><br>Disclosing an error, if you don't have		how confidentiality will be maintained if you supply your name<br><br>Individualised feedback to you about reports you submit<br><br>Paper forms for reporting provided in each theatre<br><br>More support from colleagues<br><br>Less blame attached to those who report errors<br><br>ANZCA continuing professional development point for

For peer review only

		to, is an optional act of heroism<br><br>I would cover up an error I had made if I could<br><br>If I admit to an error I will feel like a failure<br><br>It would affect my self-esteem to admit to an error<br><br>Doctors who make errors are humiliated my their colleagues<br><br>Medicine has a culture of silence where errors are not talked about<br><br>Doctors who make errors are blamed by their colleagues<br><br>Doctors should not make errors.		reports.<br><br>Access to computer based reporting systems for home<br><br>Education about the purpose of reporting<br><br>Computer based reporting systems<br><br>Training on how to use computer based system<br><br>Training on how to fill in papers forms for reporting<br><br>Payment for time taken to report

For peer review only - http://bmjopen.bmj.com/site/about/guidelines.xhtml

Herdeiro et al., 2006 [125]	Questionnaire-based study<br>256<br>Portugal	Lack of time<br><br>Complexity of reporting	Workplace (hospital pharmacists more likely to report than community pharmacists)<br><br>Really serious ADRs are not well documented by the time a drug is marketed'<br><br>Serious and not expected ADRs<br><br>Report an ADR if I were unsure that it was related to the use of a particular drug	Gender<br><br>Age<br><br>Job function (registered, assistant or other pharmacists)<br><br>Possible to determine if a drug is responsible for a particular adverse reaction'<br><br>Cannot contribute to pharmaceutical knowledge<br><br>Interested in articles about ADRs'<br><br>Most correct way to report ADRs in is the pharmaceutical

For peer review only - http://bmjopen.bmj.com/site/about/guidelines.xhtml

				literature<br><br>Financially reimbursement for providing the ADR service<br><br>Professional obligation to report ADRs<br><br>Reporting ADRs puts career at risk<br><br>I do not have time to complete the report card<br><br>I do not know how the information in the report card is used<br><br>I talk to pharmaceutical companies about possible ADRs with their

For peer review only

For peer review only - http://bmjopen.bmj.com/site/about/guidelines.xhtml

				drugs
Hohenhaus et al., 2008 [42]	Questionnaire-based study<br>175<br>United States of America	Afraid to report a medical error they had made<br><br>Afraid to report a medical error made by someone else<br><br>Might not report if there was no harm to the patient and the error was recognised quickly<br><br>Might not report if a physician told them not to report the error<br><br>Would not report if their supervisor told them not to	Error resulting patient harm<br><br>Error by novice nurse	
Holmstrom et al., 2012 [68]	Questionnaire-based study<br>16<br>United Kingdom	Fear of consequences<br><br>Culture of blame<br><br>Lack of training in MER for health-care professionals<br><br>Lack of time for reporting<br><br>Lack of organizational leadership and support<br><br>Lack of legal protection for individual health-care professionals who have	Provides opportunity for evaluating causes of errors (e.g. root cause analysis)<br><br>Uses a non-punitive approach to reporting<br><br>Provides feedback of results of error analysis for those involved in	Paper-based<br><br>Quick and easy to use

For peer review only - http://bmjopen.bmj.com/site/about/guidelines.xhtml

For peer review only

		made an error<br><br>Lack of understanding why reporting is needed<br><br>Concern that no beneficial action will follow<br><br>Non-anonymous reporting<br><br>Perceived to be bureaucratic<br><br>Lack of health-care staff<br><br>Lack of financial resources	reporting<br><br>Easy to use<br><br>Provides opportunity for error data analysis<br><br>Produces recommendations and guidelines for improving medication safety<br><br>Provides confidentiality of reported information<br><br>Provided and maintained by one national organisation<br><br>Integral part of patient safety reporting system<br><br>Reporting of errors is voluntary	

For peer review only - http://bmjopen.bmj.com/site/about/guidelines.xhtml

			Reporting of errors is mandatory<br><br>Allows all healthcare professionals to report errors<br><br>Available in electronic format<br><br>Independent reporting system dedicated for medication error reporting<br><br>Provides a choice of reporting anonymously<br><br>Includes reporting of both potential and actual errors	
Hutchinson et al., 2009 [29]	Retrospective analysis of routinely collected data and questionnaire-based study<br>Sample size not stated<br>United Kingdom		Employer treats fairly staff involved in error near miss or incident<br><br>Employer encourages staff to	Knows how to report errors, near misses and incidents<br><br>When errors are reported,

For peer review only - http://bmjopen.bmj.com/site/about/guidelines.xhtml

			report errors, near misses or incidents<br><br>Employer treats reports of errors, near misses or incidents confidentially<br><br>Employer does not blame or punish people who make errors.<br><br>Access to a counselling service were also more likely to report.<br><br>Previous reporting behaviours<br><br>Level of risk management	employer takes action to ensure that they do not happen again
Irujo et al., 2007 [129]	Case control study<br>78<br>Spain	Not serious ADR<br><br>Already well known ADR<br><br>Uncertain about causality<br><br>Forgot to report		Age<br><br>Working experience as pharmacist<br><br>Participation in

For peer review only - http://bmjopen.bmj.com/site/about/guidelines.xhtml

		Lack of time		a programme for detection and resolution of DRPs<br><br>Education on detection and resolution of DRPs<br><br>Frequently considering the possibility of finding an ADR when attending a patient with symptoms<br><br>Forgetting to report<br><br>Education for ADR reporting<br><br>Awareness of the importance of reporting system<br><br>It is necessary to be sure that the reaction is

For peer review only - http://bmjopen.bmj.com/site/about/guidelines.xhtml

				causally related to the use of a particular drug<br><br>Basic knowledge about ADR reporting
Jeffe et al., 2004 [11]	Focus groups<br>109<br>United States of America	Not knowing what to report<br><br>Errors that pose little risk to the patient<br><br>Errors that do not end up harming the patient<br><br>Not knowing how to report<br><br>Fear of disciplinary repercussions (nurse and physicians)<br><br>Fear of legal repercussions (nurse and physicians)<br><br>Fear of repercussions from doctors (nurses)<br><br>Link between reporting and performance reviews (nurses)	Severity of the situation (nurses)<br><br>Likelihood of reoccurrence (nurses)<br><br>Severe events reported as the error would be 'found' out anyway<br><br>Self-protection<br><br>The importance of reporting errors for educational purposes<br><br>Anonymous (physician and nurses)	

For peer review only - http://bmjopen.bmj.com/site/about/guidelines.xhtml

		Protecting colleagues from disciplinary action(nurses)<br><br>Lack of confidentiality<br><br>Name, blame, shame culture<br><br>Fear of public exposure<br><br>Staff shortages<br><br>Lack of time<br><br>The lack of simple procedure for reporting errors<br><br>Lack of feedback	Simple (physician and nurses)<br><br>Fast reporting procedures(physician and nurses)<br><br>Receipt of critical feedback about the errors<br><br>Anonymous, phone in system (physicians)<br><br>Educational rather than punitive system (physicians)<br><br>System that was 'lawyer proof'<br><br>Blame free reporting (nurses)	
Jennings et al., 2011 [27]	Focus groups, interviews and questionnaire based study<br>Sample size not stated<br>Australia	Burden of reporting in terms of time<br><br>Lack of accessibility of reporting forms	Clarity of indemnity from prosecution	

For peer review only - http://bmjopen.bmj.com/site/about/guidelines.xhtml

		Time elapsed following incident<br><br>Priority of reporting over other work tasks<br><br>Forgetting to report<br><br>Workload<br><br>Fear of disciplinary action<br><br>Fear of potential litigation<br><br>Fear of breaches of confidentiality/anonymity<br><br>Fear of embarrassment within peer group<br><br>Fear that incidents many impact on their likelihood of promotion<br><br>Concern that nothing would change even if the incident was reported<br><br>Lack of familiarity with process		
Johnstone et al., 2008 [84]	Focus groups, semi-structured interviews and questionnaire-based study 35	Frequency of incident-more frequent less likely to report	Seniority of graduate nurses	

For peer review only - http://bmjopen.bmj.com/site/about/guidelines.xhtml

	Australia			
Joolaee et al., 2011 [113]	Questionnaire-based study<br>286<br>Iran			Perceived work conditions
Kagan et al., 2008 [110]	Questionnaire-based study<br>201<br>Israel	The practice of ward nurse managers to cover up error, that is dealing with the error themselves without reporting to a higher authority	How the ward's and hospital's dealt with medication error<br><br>How their ward handles error reporting	
Kagan et al., 2013 [111]	Questionnaire-based study<br>247<br>Israel	Medical error incidence	Patient safety culture index<br><br>PSC at organisational level<br><br>PSC at departmental level<br><br>PSC at respondents personal performance level<br><br>Nurses' place of birth and their professional status (academic or non-academic	

For peer review only - http://bmjopen.bmj.com/site/about/guidelines.xhtml

			registered nurse)	
Kaldjian et al., 2009 [41]	Questionnaire-based study<br>338<br>United States of America		Feedback	
Karsh et al., 2006 [33]	Focus group<br>14<br>United States of America	Length of report<br><br>Punishment<br><br>Reporting near misses	Feedback<br><br>Mandatory system<br><br>Financial incentives<br><br>Other incentives (protection from malpractice and disciplinary action)<br><br>Support in using system<br><br>Education in using system	
Kennedy et al., 2004 [34]	Questionnaire-based study<br>113<br>United States of America	Not their responsibility to report<br><br>Never thought to report/not required to do so<br><br>Handle errors internally i.e. no corporate system<br><br>No errors worth reporting		

For peer review only

		No time to report<br><br>Forms not available or convenient		
Khan, 2013 [105]	Questionnaire-based study<br>50<br>Saudi Arabia	Unavailability of professional environment to discuss ADR<br><br>Reporting forms are not available<br><br>I do not know how to report<br><br>Reporting forms are too complicated<br><br>Reporting is time consuming<br><br>I am not motivated to report<br><br>I fear legal liability of the reported ADR<br><br>I am not confident whether it is an ADR<br><br>Insufficient knowledge of pharmacotherapy in detecting ADR<br><br>Belief that only safe drugs are marketed-not cause of reaction		
King et al., 2006 [56]	Questionnaire-based study<br>39	Time constraints		

For peer review only - http://bmjopen.bmj.com/site/about/guidelines.xhtml

	United States of America	Difficulty locating forms<br><br>Lack of closure/feedback<br><br>Not important<br><br>Fear of disclosure to risk management		
Kingston et al., 2004 [9]	Focus groups<br>33<br>Australia	Lack of knowledge about the reporting process and<br><br>Lack of knowledge about what constitutes an incident<br><br>"Nursing form" by association (not identified as being part of doctors role)<br><br>Time constraint<br><br>Complexity of reporting form<br><br>Lack of feedback<br><br>Lack of legal privileges afforded to the reporting process<br><br>Culture of blame<br><br>No value	Effective and efficient IRS<br><br>IRS with threat or blame<br><br>Prompt, relevant feedback<br><br>IRS that drive improvements<br><br>Monetary payment<br><br>Simplification<br><br>Less time consuming<br><br>Clear definitions of what constitutes an adverse event/near-miss	

For peer review only - http://bmjopen.bmj.com/site/about/guidelines.xhtml

			Evidence of value of IRS<br><br>Reporting process to be made more relevant to doctors<br><br>Reporting process less threatening by renaming the form<br><br>Increased awareness and knowledge of IR process<br><br>Protection from liability<br><br>System that doesn't require input from doctors (nurses)<br><br>Education at orientation (nurses)<br><br>Anonymous reporting	
Kreckler et al.,	Questionnaire-based study	I am too busy to fill out the form		

For peer review only - http://bmjopen.bmj.com/site/about/guidelines.xhtml

2009 [69]	137<br>United Kingdom	The form takes too long to complete<br><br>I am worried about litigation<br><br>I do not want the case discussed in meetings<br><br>I never get any feedback<br><br>It makes little contribution to the quality of care<br><br>I am not sure what incidents to report<br><br>The incident was too trivial<br><br>The incident did not result in any harm		
Li et al., 2004 [127]	Questionnaire-based study<br>1653<br>China	Address of reporting agency not available<br><br>Report forms unavailable<br><br>Reporting process unknown<br><br>Unaware of a national ADR reporting system<br><br>Patient confidentiality	Increasing awareness among administrators, doctors & nurses<br><br>Establishing ADR institutes<br><br>Education and training in ADR knowledge and related topics	

For peer review only - http://bmjopen.bmj.com/site/about/guidelines.xhtml

		Too busy to report ADR<br><br>ADR sufficiently well documented<br><br>Reluctant to admit that they have caused a patient harm<br><br>Worried about feeling foolish<br><br>Reluctant to admit they may have made a medical error<br><br>Personal ambition to publish a case study		
Martowirono et al., 2012 [100]	Focus group<br>22<br>Netherlands	Negatively valued<br><br>Costs time<br><br>Perceived as another administrative task that they have to complete<br><br>Priority<br><br>Do not always agree with the definition of incident<br><br>Incidents that had no major patient consequence<br><br>Incidents that have happened before and has already been reported	Reporting process-ability to report over the phone or send an email<br><br>Anonymous reporting<br><br>Provide the possibility to report without identifying the person involved<br><br>Provide feedback<br><br>Provide feedback to the reporter if an	

For peer review only - http://bmjopen.bmj.com/site/about/guidelines.xhtml

		Incidents that was not preventable<br><br>The cause of the incident Is already clear<br><br>Incidents is unlikely to happen again<br><br>Was not an incident but a complication<br><br>Incident already been discussed with the people involved<br><br>The lack of feedback on a report<br><br>Absence of visible system changes were also issues<br><br>Disloyal to colleagues<br><br>Not their responsibility<br><br>legal liability<br><br>Unpleasant working conditions<br><br>Lack of encouragement from superiors to report incidents.<br><br>Incident reporting is emotionally charged	incident on how the report will be handled<br><br>Feedback-communicate the results in terms of systems changes<br><br>Create an incident reporting culture<br><br>Create a culture in which IR is less emotionally charged e.g. by systematically discussing IR within a ward and stimulating role of supervisors<br><br>Simplify the procedure<br><br>Design a procedure in which it is possible to only report the essentials of an incident, e.g. by making a call or	

For peer review only - http://bmjopen.bmj.com/site/about/guidelines.xhtml

		Some residents stated that they did not complete IR because they did not think of it whereas others said<br><br>Did not know what to report.<br><br>Did not know how to report<br><br>IRS complicated<br><br>Workload	filing out a card or compact form with standard incidents. If necessary, the resident can be contacted for more information<br><br>Make it easy for a resident to find out if an incident has already been reported<br><br>Clarification what to report<br><br>Clarification about and how to report<br><br>Excite residents to report<br><br>Draw attention to IR e.g. putting up posters with a catchy slogan	
Mayo et al., 2004 [53]	Questionnaire-based study<br>983<br>United States of America	Afraid of manager reaction<br><br>Afraid of co-workers' reactions		

For peer review only - http://bmjopen.bmj.com/site/about/guidelines.xhtml

		Not thinking an error was serious enough<br><br>Fear of disciplinary action		
McArdle et al., 2003 [78]	Semi-structured interviews<br>15<br>United Kingdom	It takes too long<br><br>Lack of feedback received<br><br>Lack on incentive<br><br>Cumbersome<br><br>Non-anonymous<br><br>Fear of blame<br><br>Description of medication did not fall into IRS formats-scope of reporting		
Merchant et al., 2005 [93]	Questionnaire-based study<br>207<br>Canada	I think of reporting too late<br><br>Don't know where CIRS forms are<br><br>Fear of lawyers getting information<br><br>I don't know what sort of incident to report<br><br>I'm too busy<br><br>Fear of record of problem		Unnecessary as anesthesia is safe<br><br>futile as anesthesia is safe

For peer review only - http://bmjopen.bmj.com/site/about/guidelines.xhtml

		Don't have CIRS forms<br>My incidents are too minor<br>Too long<br>No value will come of this<br>Too much writing<br>Incidents I see are other's problem<br>Too many tick boxes<br>Unsure what 'critical incident' is<br>Effort is doomed to failure<br>Too difficult<br>Form is confusing<br>Unimportant to me<br>Nothing can be learned from me<br>CIRS asks wrong questions		
Mrayyan et al., 2007 [126]	Questionnaire-based study<br>779<br>Jordan	Fear of disciplinary action/lose job<br>Errors not serious to warrant		

For peer review only

		reporting<br><br>Fear of reaction from co-workers<br><br>Fear of reaction from nurse managers		
Mustafa et al., 2013 [124]	Questionnaire-based study<br>136<br>Pakistan	Uncertain association<br><br>Awareness<br><br>Concern about legal liability	Seriousness of ADRs<br><br>Unusual reaction<br><br>Reaction to a new product<br><br>Confidence in the diagnosis of ADR	
Naveh et al., 2006 [112]	Questionnaire-based study<br>632<br>Israel	Perceived safety procedures	Perceived safety information flow	Perceived priority of safety<br><br>Unit type
Okuyama et al., 2010 [115]	Questionnaire-based study<br>430<br>Japan		Safety management at ward level	Safety management at the hospital level<br><br>Attitudes of ward safety managers

For peer review only - http://bmjopen.bmj.com/site/about/guidelines.xhtml

Osborne et al., 1999 [54]	Questionnaire-based study<br>57<br>United States of America	Error not serious<br><br>Afraid of repercussions<br><br>Afraid of reactions from managers/co-workers		Perceptions of medication errors
Parvizi et al., 2014 [70]	Questionnaire-based study<br>119<br>United Kingdom	Did not know they were expected to do this<br><br>Did not know how to report to MHRA<br><br>I do not see the purpose of reporting<br><br>Lack of time<br><br>Blame<br><br>Direct reporting to the manufacturer<br><br>Not reporting if the types of device failure were considered to be common knowledge<br><br>Reporting only those that were unexpected failures or failures that may affect the patient or user<br><br>Reported by either a nurse or other doctor	Better education of the means of adverse IR<br><br>Improvements in the feedback sent to the reporter on the outcomes of the adverse incidents<br><br>Improvements in the guidance on the type of adverse device related incidents to report<br><br>Improvements in the electronic means of adverse IR<br><br>Improvements in the clinical and	

For peer review only - http://bmjopen.bmj.com/site/about/guidelines.xhtml

			adverse incidence governance	
Patrician et al., 2009 [43]	Questionnaire-based study<br>43<br>United States of America	Perceptions that the administration focuses on the individual and not the system<br><br>Nurses are blamed when something bad happened to patients<br><br>Fear adverse consequences for reporting errors<br><br>Nurses believe that their peers will think them incompetent<br><br>Nurses do not think the error was important enough to report<br><br>Fear of administrative response<br><br>Disagreement over error<br><br>Reporting effort<br>Lack of agreement about definition of error<br><br>Lack of error recognition<br>Excessive length of time for contacting physician		
Rasmussen et al.,	Questionnaire-based study		Safety climate	

For peer review only

2014 [122]	124<br>Denmark		Team climate<br><br>Inter-departmental working relationships<br><br>Increased cognitive demands	
Rogers et al., 1988 [51]	Questionnaire-based study<br>1121<br>United States of America	Reporting forms not available<br><br>Event already documented<br><br>Did not get to it/got busy<br><br>Did not believe it was important<br><br>Forms were too much trouble<br><br>Minor or expected side effect<br><br>Did not like interacting with the government<br><br>Liability concerns<br><br>Did not know how to report<br><br>Undetermined as ADE<br><br>Not primary physician		Age<br><br>Time in direct patient care

For peer review only - http://bmjopen.bmj.com/site/about/guidelines.xhtml

Rowin et al., 2008 [28]	Descriptive study<br>Sample size not stated<br>United States of America		More likely to report no harm (nurses)<br><br>More likely to report permanent harm, near death, death and unsafe environment (doctors)<br><br>Type of incident: falls and medication (nurse)<br><br>Type of incident: adverse clinical event (doctors)	Temporary harm<br><br>Near miss
Sanghera et al., 2007 [79]	Semi-structured interviews<br>13<br>United Kingdom	Not being aware that an error had occurred<br><br>Detailed paperwork<br><br>Time constraints<br><br>Not understanding incident reporting process<br><br>No benefit (perception that nothing is done with the data)		

For peer review only - http://bmjopen.bmj.com/site/about/guidelines.xhtml

		No encouragement by management<br><br>Fear of loss of professional registration<br><br>Fear of being in trouble<br><br>Fear of looking incompetent<br><br>Feeling upset<br><br>Fear will be blamed<br><br>Not wanting to report colleagues' errors		
Sarvadikar et al., 2010 [71]	Questionnaire-based study<br>56<br>United Kingdom		Doctors more likely to report errors with worsening patient outcome	Nurses and pharmacists likely to report error regardless of patient outcome
Schectman et al., 2006 [44]	Questionnaire-based study<br>120<br>United States of America	Unsure of reporting mechanism<br><br>No actual harm came to the patient<br><br>Reporting too difficult and time consuming<br><br>Unsure of what is considered AE/NM	Allow electronic reporting of adverse events and near misses<br><br>Clarify reporting mechanism	

For peer review only - http://bmjopen.bmj.com/site/about/guidelines.xhtml

		Inadequate MD participation in scheme<br><br>Concern about consequences of reporting others' error<br><br>Reporting makes no difference (nothing will change)<br><br>Concern about being blamed or judged less competent<br><br>Weaknesses in the reporting system<br><br>Professional behaviours<br><br>Fear of retribution<br><br>Lack of feedback and the perception that change would not result from reports.	Clarify what constitutes an AE/NM<br><br>Allow anonymous reporting<br><br>Increase physician involvement in QI<br><br>Provide feedback on QI projects arising from reports<br><br>Provide individual feedback following report<br><br>Provide summary feedback on a regular basis<br><br>Make reporting mandatory	
Schulmeister et al., 1999 [45]	Questionnaire-based study<br>160<br>United States of America	Minor error<br><br>Fear of disciplinary action		
Sharma et al., 2008 [74]	Questionnaire-based study<br>81<br>United Kingdom	Does not achieve anything<br><br>Not in physicians culture	Anonymous system<br><br>Easily accessible	

For peer review only - http://bmjopen.bmj.com/site/about/guidelines.xhtml

		Do not wish to incriminate others<br><br>Do not know how to access forms<br><br>Not bothered<br><br>Do not wish to ask nurse staff<br><br>Lack of time<br><br>Do not know which incidents need to be reported<br><br>Lack of anonymity<br><br>Not in habit of considering it<br><br>Discouraged by senior nurses	forms<br><br>Forms not held by nursing staff	
Soberberg et al., 2009 [118]	Questionnaire-based study<br>317<br>Sweden	I did not have enough time<br><br>I am concerned about possible consequences<br><br>Someone else did it<br><br>It is too complicated<br><br>No one else files incident reports<br><br>It would not make any difference		

For peer review only - http://bmjopen.bmj.com/site/about/guidelines.xhtml

		Insufficient routines for reporting		
Soleimani., 2006 [116]	Questionnaire-based study<br>128<br>New Zealand	Threat of public outcry<br><br>Professional consequences/discipline<br><br>Embarrassment in front of colleagues		
Stratton et al., 2004 [59]	Questionnaire-based study<br>284<br>United States of America	No positive feedback is given for passing medications correctly<br><br>Nurse administration focuses on the person rather than looking at the system<br><br>Too much emphasis is placed on medication errors as a measure of the quality of care<br><br>Responses by nursing administration do not match the severity of the error<br><br>Individual/personal reasons<br>Nurses could be blamed if something happened to the patient<br><br>Nurse believe other nurses will think they are incompetent		

For peer review only - http://bmjopen.bmj.com/site/about/guidelines.xhtml

		Nurses fear adverse consequences from reporting<br><br>Patient might develop a negative attitude<br><br>Nurses fear reprimand from physician<br><br>Nurses fear losing their license<br><br>Nurses want to avoid potential publicity of medication errors in the media		
Sweis et al., 2000 [77]	Questionnaire-based study<br>280<br>United Kingdom	Busy<br><br>Legal liability<br><br>Fear of breaching patient confidentiality	Serious ADR rather than trivial<br><br>Rarely occurring ADR rather than common ADR<br><br>Confidence in recognising an ADR<br><br>ADR to an established drug rather than new drug<br><br>Active support of	Training in reporting<br><br>Gender<br><br>Type of hospital<br><br>Age

For peer review only - http://bmjopen.bmj.com/site/about/guidelines.xhtml

			medical/pharmacy staff<br><br>Written hospital policy for pharmacist ADR reporting<br><br>Training and ADR meeting<br><br>Increasing seniority<br><br>Allocation of time for ADR monitoring<br><br>Publicity and promotion by hospital and CSM<br><br>Better cooperation with clinicians<br><br>Support and encouragement by the pharmacy department<br><br>More ward rounds and direct patient contact	

For peer review only - http://bmjopen.bmj.com/site/about/guidelines.xhtml

			Simplify reporting system<br><br>ADR reporting team<br><br>Feedback	
Tariq et al., 2012 [83]	Semi structured interviews<br>23<br>Australia	Lack of time		
Taylor et al., 2004 [46]	Questionnaire-based study<br>140<br>United States of America	Not important to report error that did not harm patient<br><br>Reporting errors does not make any difference<br><br>Unsure about what is considered medical<br><br>Incident report form too complicated<br><br>Concerned about being blamed or judged incompetent<br><br>Concerned about implicating others<br><br>Unsure whose responsibility it is to report errors	Make reporting of errors mandatory<br><br>Different format for IR<br><br>Use of electronic format for reports<br><br>Reward for reporting medical errors<br><br>Better education about what is considered a medical error that should be reported<br><br>Evidence that reporting of errors	

For peer review only - http://bmjopen.bmj.com/site/about/guidelines.xhtml

			led to system changes<br><br>Feedback on regular basis and frequencies of reported errors<br><br>Feedback regarding outcome of a specific error that has been reported	
Throckmorton et al., 2007 [47]	Questionnaire-based study<br>435<br>United States of America	Level of harm: no harm	Level of harm<br><br>Working closely to the patient<br><br>Higher scores on the Wakefield's scale<br><br>Fewer years since initial license	
Tobaiqy et al., 2013 [106]	Questionnaire-based study<br>61<br>Saudi Arabia	Lack of awareness<br><br>Workload/time constraints<br><br>Unavailability of reporting form	Continuing education events<br><br>An internet/web based reporting facility	

For peer review only - http://bmjopen.bmj.com/site/about/guidelines.xhtml

		Reporting system complexity<br><br>Error too trivial<br><br>Lack of anonymity<br><br>Fear of blame<br><br>Concerns over penalisation<br><br>Difficulty in recognising errors<br><br>Senior staff advised not to report<br><br>Lack of feedback from authority	Training focused on error prevention<br><br>Anonymity of reporting<br><br>A non-punitive reporting culture<br><br>Financial incentives linked to reporting	
Turner et al., (2013) [63]	Semi-structured interviews<br>32<br>United Kingdom	Value-not convinced that the reporting system would deliver improvements in clinical care		
Uribe et al., 2002 [48]	Questionnaire-based study<br>122 United States of America	Time involved in documenting an error<br><br>Extra work involved in reporting<br><br>Hesitancy regarding 'telling' on somebody else<br><br>Thinking that it is unnecessary to report error because it had no negative outcome		Thinking that reporting has little contribution for improvement of quality care<br><br>Not knowing the usefulness of the report<br><br>Lack of

For peer review only - http://bmjopen.bmj.com/site/about/guidelines.xhtml

		Not being able to report anonymously<br><br>Fear of lawsuits		knowledge of what should be reported<br><br>Lack of recognition that a medical error has occurred<br><br>Fear of being blamed<br><br>Fear of disciplinary action/ losing job<br><br>Lack of information in how to report<br><br>Lack of interest or motivation for reporting<br><br>Forms or computer locations not available to report medical errors

For peer review only

				Not knowing who is responsible for reporting error
Vessal et al., 2009 [114]	Questionnaire-based study<br>110<br>Iran	Uncertain association<br><br>Too trivial to report<br><br>Too well known to report<br><br>Yellow card not available<br><br>Not enough information from the patient<br><br>Not enough time<br><br>Unaware of the existence of a national ADR reporting system<br><br>Too bureaucratic<br><br>Did not know how to report<br><br>Fear of legal liability<br><br>Unaware of the need to report and ADR	The reaction is of a serious nature<br><br>The reaction is unusual<br><br>The reaction is to a new product<br><br>Reaction not reported before for a particular drug<br><br>Reaction is well recognised for a particular drug<br><br>Any reaction	
Vincent et al., 1998 [72]	Questionnaire-based study<br>198	Unnecessary		Unsupported colleagues

For peer review only - http://bmjopen.bmj.com/site/about/guidelines.xhtml

	United Kingdom	Increased workload<br><br>Blame<br><br>Worry litigation<br><br>Busy/forgot		Not knowing which incidents to report<br><br>As long as staff learn from incident it is unnecessary to discuss/report<br><br>Fear disciplinary<br><br>Not wanting incident to be discussed<br><br>Who's responsibility<br><br>Little contribution
Vogus et al., 2007 [49]	Questionnaire-based study<br>1033<br>United States of America	Safety organising<br><br>Unit type (emergency)<br><br>Safety organising and trust<br><br>Safety organising and pathways	Trust in managers<br><br>RN experience<br><br>Unit type (IC)<br><br>Number of beds	Care pathways<br><br>% of RNs with BSN<br><br>Unit type (surgery)

For peer review only - http://bmjopen.bmj.com/site/about/guidelines.xhtml

		Patient-to-RN ratio		
Walji et al., 2011 [89]	Semi- structured interviews<br>12<br>Canada	Lack of knowledge about natural health products<br><br>Lack of time/priorities<br><br>Complexity of reporting process	Pharmacists who saw themselves as 'knowledge generators' rather than just 'knowledge users' were more likely to report and less likely to allow workplace challenges to prevent their taking an extra step	
Walker et al., 1998 [85]	Focus groups and questionnaire-based study<br>43<br>Australia	Minor incidents (documentation and minor variation from the prescription)<br><br>Negative past experience of reporting<br><br>Fear of getting into trouble<br><br>Fear they will somehow stand out from the crowd in the eyes of those in authority<br><br>Feelings of discomfort or uncertainty about being required to report an incident that involved a colleague	More likely to report an incident if patient safety compromised<br><br>Capacity to feedback and improve the situation<br><br>Reporting might help raise people's awareness of problems that could be occurring	Fear of possible punishment senior staff

For peer review only - http://bmjopen.bmj.com/site/about/guidelines.xhtml

		This is more difficult if the colleague is a more experienced nurse<br><br>Others expressed with view that they wouldn't report a friend, perhaps perceiving that the friend would be in trouble if the incident was reported<br><br>Did not always want to admit their mistake<br><br>Might not even realise that an error had occurred<br><br>Incident might be highly incriminating<br><br>If the patient actually came to harm as a result of the error<br><br>If the departure from the prescribed therapy seemed reasonable<br><br>If the problem could be sorted out<br><br>Concern about the time taken to fill in the incident report form<br><br>Inadequate understanding of what constituted an error<br><br>A lack of feedback on the number of medication errors was a problem	Wrong drug<br><br>Wrong route<br><br>Wrong person<br><br>Wrong dose<br><br>Harm to the patient<br><br>A desire to target an individual or professional group to improve practice<br><br>Legal obligation of the nurse to report	

For peer review only - http://bmjopen.bmj.com/site/about/guidelines.xhtml

		Perceived inaction on reported errors incidents		
Waring, 2004 [64]	Semi- structured interviews<br>37<br>United Kingdom	Acute medicine and rehab: IR system was regarded as nurse led, dealing with ward issues and the work of non-medical groups<br><br>Anaesthesia: Physicians remained sceptical about the hospital wide reporting system and were generally disinclined to participate in this approach		
Waring, 2005 [10]	Semi-structured interviews<br>28<br>United Kingdom	Fear of blame<br><br>Blame culture<br><br>Peer of punishment<br><br>Fear of blame from pubic<br><br>Fear of litigation<br><br>Fear of professional competence being questioned<br><br>Fear of poor references<br><br>Reprimands from a senior colleague		

For peer review only - http://bmjopen.bmj.com/site/about/guidelines.xhtml

		Fear of use of reports-could be used at a later date in the event in medico-legal disputes		
Waters et al., 2012 [90]	Focus groups<br>16<br>Canada	Time<br><br>Fatigue<br><br>High workload<br><br>Relevance of reporting form<br><br>Complexity of reporting-gathering many pieces of information.<br><br>Unit culture<br><br>Fear of blame<br><br>Close knit team<br><br>Other methods of reporting-verbal reporting and team debrief<br><br>Lack of feedback	Previous experience of litigation<br><br>Protection against future litigation<br><br>Professional responsibility<br><br>IR perceived as learning opportunity<br><br>Desire for practice improvement	Risk of litigation
Weissman et al., 2005 [50]	Questionnaire-based study<br>203<br>United States of America	Mandatory<br><br>Non-confidential system<br><br>State run	Serious harm	

For peer review only - http://bmjopen.bmj.com/site/about/guidelines.xhtml

		Less harm		
Williams et al., 2013 [65]	Focus groups<br>17<br>United Kingdom	Severity (more likely to report if serious harm	Simpler reporting system<br><br>Targeted report<br><br>Feedback<br><br>Drug-specific error reporting forms<br><br>Electronic forms/systems (easier than paper)<br><br>Anonymous reporting	
Winchester et al., 2012 [73]	Questionnaire-based study<br>120<br>United Kingdom	Concerned about confidentiality<br><br>Did not know the procedure for reporting<br><br>Did not think anything could be done<br><br>Did not feel incident was important enough to report<br><br>Believed source to be low risk<br><br>Reporting was inconvenient	Education<br><br>Adverts/posters<br><br>Training<br><br>Compulsory reporting<br><br>Simple reporting system<br><br>An electronic	

For peer review only - http://bmjopen.bmj.com/site/about/guidelines.xhtml

			reporting system	
Yong et al., 2003 [117]	Questionnaire-based study<br>136<br>New Zealand	Time constraints<br><br>Laziness and forgetfulness<br><br>Dislike form filling<br><br>A lot of work for little practical benefit<br><br>Forms too complicated<br><br>Do not believe the system is working<br><br>Many incidents not worth reporting<br><br>Many other tools exist for correcting errors and improving standards<br><br>Dislike the published interpretation of results with diagnostic views by some anaesthetists<br><br>Qualitative result not acceptable<br><br>Feel that the main benefit of IR is local analysis and that very rare events distilled by multi-site monitoring are less important<br><br>Difficulty defining what constitutes incident	Total anonymity and confidentiality<br><br>Protection against punitive action<br><br>Simplify forms and bring up to date<br><br>Easy access to forms<br><br>Electronic data entry<br><br>Incorporating IR form filling at regular M&M meetings<br><br>Mandatory<br><br>Local analysis rather than Australasian wide<br><br>More aggressive follow up and reviewing	

For peer review only - http://bmjopen.bmj.com/site/about/guidelines.xhtml

		Inadequate feedback<br><br>Medico-legal implications<br><br>Forms not available/hard to locate<br><br>Lack of appropriate culture within department<br><br>Not accepted as part of private practice culture<br><br>Use of local IR system, hospital based audit<br><br>Incidents are discussed at department level confidentially	Publication of problems<br><br>Aims and purpose should be clarified explicitly<br><br>Select a few incidents to monitor frequency	
Zwart et al., 2011 [102]	Prospective cohort study<br>66<br>Netherlands		Expertise	Communicator<br><br>Collaborator<br><br>Manager<br><br>Health advocate<br><br>Scientist<br><br>Professional

For peer review only - http://bmjopen.bmj.com/site/about/guidelines.xhtml

Adverse Drug Event (ADE); Adverse Drug Reaction (ADR); Adverse Event (AE); Australia and New Zealand College of Anesthetists (ANZCA); Bachelor of Science in Nursing (BSN); Critical Incident Reporting Service (CIRS); Drug related problems (DRP); Incident Reporting (IR); Iowa Department of Inspections Appeals (IDIA); Incident Information Management System (IIMS); Intensive Care (IC); Medication Administration Error (MAE); Medication and Healthcare Products Regulatory Agency (MHRA); Medical Doctor (MD); Morbidity and Mortality (M&M); Near Miss (NM); Patient Safety Culture (PSC); Quality Improvement (QI); Register Nurse (RN)

For peer review only

For peer review only - http://bmjopen.bmj.com/site/about/guidelines.xhtml

eTable 2: Frequency of factors influencing engagement in incident reporting

Factor		Impact on Reporting Engagement: **Barrier** Frequency Count *(%)*	**Facilitator** Frequency Count *(%)*	**Negative Case (no impact)** Frequency Count *(%)*
Fear of Adverse Consequences	Adverse consequences	51 *(31.68%)* [8, 10, 11, 27, 30, 32, 33, 35-37, 42-45, 53-56, 58, 59, 61, 68, 75, 78, 79, 85, 87, 88, 92, 97, 99, 100, 104, 106, 109, 118, 120, 121]	-	3 *(25.00%)* [72, 85, 96]
	Litigation	30 *(18.63%)* [8-11, 24, 27, 32, 35, 48, 51, 52, 61, 69, 72, 77, 80, 81, 85, 87, 88, 93, 100, 101, 103, 105, 107, 114, 117, 124, 128]	8 *(61.54%)* [9, 11, 27, 33, 82, 88, 90]	4 *(33.33%)* [24, 40, 48, 90]
	Blame	24 *(14.91%)* [8, 10, 32, 35, 43, 44, 46, 58-61, 68, 70, 72, 78, 79, 82, 87, 90, 92, 99, 106]	4 *(30.77%)* [9, 11, 87, 88]	1 *(8.33%)* [48]
	Judgment	22 *(13.66%)* [10, 24, 35, 43, 53, 59, 67, 79, 80, 88, 92, 99, 104, 107, 109, 116, 126]		1 *(8.33%)* [101]
	Relationships	12 *(7.45%)* [10, 11, 36, 44, 46, 48, 54, 59, 92, 104, 116, 120]	-	-
	Impact on career	10 *(6.21%)* [10, 11, 27, 58, 59, 79, 86, 92, 93, 126]	-	1 *(8.33%)* [125]
	Protection of self	7 *(4.35%)* [24, 76, 80, 107, 122, 127]	-	-
	Avoid discussion in meetings	4 *(2.48%)* [8, 69, 87, 117]	-	1 *(8.33%)* [72]
	Apprehension about sending inappropriate form	1 *(0.62%)* [75]	-	-
	Non-punitive	-	1 *(7.69%)* [117]	1 *(8.33%)* [123]
	**Total**	**161** ***(100%)***	**13** ***(100%)***	**12** ***(100%)***
Process and Systems of Reporting	Time	29 *(26.36%)* [8, 11, 27, 38, 43, 48, 57, 69, 74, 78, 79, 81, 85, 87, 88, 90, 92, 93, 99-101, 105-107, 114, 118, 121]	5 *(6.67%)* [9, 11, 25, 40]	-
	Complexity/simplification of reporting	28 *(25.45%)* [8, 9, 11, 31, 33, 35, 38, 44, 46,	15 *(20.00%)* [9, 11, 30, 38, 65, 68, 73,	1 *(14.29%)* [68]

For peer review only - http://bmjopen.bmj.com/site/about/guidelines.xhtml

		51, 73, 78, 79, 88-90, 93, 100, 101, 105-107, 117, 118, 125]	77, 81, 100, 101, 117]	
	Anonymity and/or confidentiality	22 *(20.00%)* [8, 11, 24, 27, 35, 48, 50, 68, 73, 74, 76-78, 80, 87, 101, 106, 107, 127]	16 *(21.33%)* [9, 11, 29, 31, 40, 44, 65, 68, 74, 87, 100, 106, 117]	1 *(14.29%)* [18]
	Reporting format	10 *(9.09%)* [31, 44, 82, 85, 90, 93, 100, 117]	21 *(28.00%)* [9, 11, 25, 30, 44, 46, 58, 61, 65, 68, 70, 75, 87, 100, 106, 107, 117]	3 *(42.86%)* [24]
	Type of reporting system	5 *(4.55%)* [38, 50, 92, 117]	11 *(14.67%)* [33, 34, 40, 44, 68, 73, 101, 117]	-
	Unknown destination of report	4 *(3.64%)* [24, 70, 101, 107]	-	-
	Not enough information to complete report	3 *(2.73%)*[94, 107, 114]	1 *(1.33%)* [76]	-
	Sharing/access of reports	3 *(2.73%)* [51, 75, 87]	-	-
	Insufficient routines for reporting	1 *(0.91%)* *[118]*	-	-
	Lack of reporting system	1 *(0.91%)* [36]	-	-
	Administrative task	1 *(0.91%)* [100]	-	1 *(14.29%)* [97]
	Relevant to different HCPs	1 *(0.91%)* [64]	2 *(2.67%)* [9, 75]	-
	Reporting focus	1 *(0.91%)* [78]	2 *(2.67%)* [68]	-
	Information not readily available	1 *(0.91%)* [31]	-	-
	Not specified	-	-	1 *(14.29%)* [97]
	When/where to report	-	1 *(1.33%)* [117]	-
	Doesn't require input from doctors	-	1 *(1.33%)* [9]	-
	**Total**	**110** ***(100%)***	**75** ***(100%)***	**7** ***(100%)***
Incident Characteristics	Level of harm	40 *(43.48%)* [8, 11, 24, 31, 35, 42-48, 50, 51, 53, 54, 58, 65, 66, 69, 70, 72, 73, 80, 85, 87, 88, 92, 100, 103, 105, 106, 109, 114, 126, 128, 129]	26 *(47.27%)* [11, 31, 40, 42, 47, 50, 58, 66, 75, 77, 82, 85, 88, 95, 114, 121, 124, 125, 128]	-
	Cause of incident	19 *(20.65%)* [35, 52, 66, 81, 82, 85, 100, 101, 103, 107, 114, 119, 124, 128, 129]	6 *(10.91%)* [40, 66, 76, 77, 125]	2 *(100%)* [125, 129]
	Frequency of incident	18 *(19.57%)* [31, 51, 66, 70, 75, 76, 84, 100, 101, 103, 114, 119, 121, 127-129]	13 *(23.64%)* [11, 66, 75, 77, 114, 121, 124]	-
	Type of incident	13 *(14.13%)* [8, 33,	8 *(14.55%)* [82,	-

For peer review only - http://bmjopen.bmj.com/site/about/guidelines.xhtml

		34, 52, 69, 81, 85, 92, 93, 100, 107, 117, 121]	85, 121]	
	Level of risk	2 *(2.17%)* [11, 58]	1 *(1.82%)* [58]	-
	Patient characteristics	-	1 *(1.82%)* [82]	-
	**Total**	**92** ***(100%)***	**55** ***(100%)***	**2** ***(100%)***
Individual HCP Characteristics	Value/attitude towards reporting	53 *(59.55%)* [8, 9, 35, 44, 46, 56, 61, 63, 64, 66, 68, 70, 73, 74, 76, 79, 81, 86-88, 92, 93, 99-101, 103, 105, 107, 109, 117, 118, 120, 121, 128]	21 *(51.22%)* [9, 11, 40, 58, 68, 82, 88, 90, 93, 95, 97, 98, 107, 111, 125]	12 *(27.91%)* [37, 48, 54, 72, 79, 96, 129]
	Forgetfulness	9 *(10.11%)* [8, 27, 31, 72, 87, 93, 117, 119, 129]	-	1 *(2.33%)* [129]
	Perception of self	9 *(10.11%)* [24, 36, 55, 80, 87, 107, 127]	2 *(4.88%)* [89, 102]	6 *(13.95%)* [24, 102]
	Emotional response	6 *(6.74%)* [24, 36, 55, 80, 87, 107, 127]	5 *(12.20%)* [31, 58, 100]	-
	Previous reporting behaviors	5 *(5.62%)* [34, 37, 52, 60, 74]	1 *(2.44%)* [29]	1 *(2.33%)* [129]
	Exposure to errors	2 *(2.25%)* [38, 97]	1 *(2.44%)* [90]	-
	Length of time in employment	2 *(2.25%)* [37]	-	1 *(2.33%)* [37]
	Seniority	1 *(1.12%)*[37]	3 *(7.32%)* [49, 77, 84]	4 *(9.30%)* [37, 52, 125, 129]
	Data required for own purposes	1 *(1.12%)*[101]	-	-
	Work hours	1 *(1.12%)*[52]	1 *(2.44%)* [52]	1 *(2.33%)* [26]
	Demographics	-	2 *(4.88%)* [37, 98]	12 *(27.91%)* [37, 49, 51, 52, 77, 96, 97, 125, 129]
	Profession	-	5 *(12.20%)* [28, 71]	5 *(11.63%)* [28, 71, 102]
	**Total**	**89** ***(100%)***	**41** ***(100%)***	**43** ***(100%)***
Knowledge and Skills	Clarify reporting mechanism	36 *(42.86%)*[9, 11, 24, 27, 31, 35, 38, 44, 46, 51, 52, 70, 73, 76, 79, 80, 87, 88, 100, 101, 103, 105, 107, 114, 119, 121, 127, 128]	2 *(5.56%)* [44, 100]	5 *(33.33%)* [29, 48, 72, 129]
	Adverse event/near miss clarity	31 *(36.90%)*[9, 11, 31, 35, 43, 44, 46, 51, 69, 74, 82, 85, 87, 88, 92, 93, 95, 99, 100, 105, 117, 121]	7 *(19.44%)* [9, 30, 44, 46, 70, 87, 100]	2 *(13.33%)* [48, 72]
	Ability in error recognition	7 *(8.33%)* [35, 75, 79, 92, 99, 106, 124]	4 *(11.11%)* [75-77, 124]	1 *(6.67%)* [48]
	Training	5 *(5.95%)* [68, 76, 82,	21 *(58.33%)* [9,	7 *(46.67%)* [25, 77,

For peer review only

For peer review only - http://bmjopen.bmj.com/site/about/guidelines.xhtml

		86, 97]	25, 33, 70, 73, 75, 76, 87, 101, 106, 117, 127]	86, 129]
	Awareness	4 *(4.76%)* [35, 43, 106, 114]	2 *(5.56%)* [75, 85]	-
	Not enough information about product being reported	1 *(1.19%)* [89]	-	-
	**Total**	**84** ***(100%)***	**36** ***(100%)***	**15** ***(100%)***
Work Environment	Workload/priority	50 *(62.50%)* [9, 11, 24, 27, 31, 34, 35, 43, 48, 49, 51, 55-58, 61, 68-70, 72, 75-77, 80, 82, 83, 88-90, 92, 93, 100, 103, 117, 119, 120, 125, 127-129]	6 *(33.33%)* [31, 75-77, 122]	3 *(30.00%)* [51, 123, 125]
	Accessibility	27 *(33.75%)* [24, 27, 31, 34, 35, 51, 52, 56, 74, 75, 80, 82, 86, 93, 101, 105-107, 114, 117, 119, 121, 127]	11 *(61.11%)* [30, 68, 73-75, 87, 100, 101, 117]	1 *(10.00%)* [48]
	Not specified	2 *(2.50%)* [61, 105]	-	-
	Unit type	1 *(1.25%)* [49]	1 *(5.56%)* [49]	3 *(30.00%)* [49, 112]
	Physical working conditions	-	-	1 *(10.00%)* [26]
	Satisfaction with work environment	-	-	1 *(10.00%)* [113]
	Care pathways	-	-	1 *(10.00%)* [49]
	**Total**	**80** ***(100%)***	**18** ***(100%)***	**10** ***(100%)***
Organization	Feedback/communication	26 *(34.21%)* [8, 9, 11, 35, 37, 43, 44, 56, 58, 59, 61, 62, 69, 78, 85-87, 90, 92, 99, 100, 106, 108, 117, 123]	29 *(29.90%)* [9, 11, 30, 33, 41, 44, 46, 61, 65, 68, 70, 75-77, 87, 100, 101, 107, 112, 117]	2 *(9.09%)* [25, 125]
	Reporting culture	17 *(22.37%)* [9, 10, 34, 35, 49, 66, 70, 81, 86, 90, 92, 114, 117, 118, 123]	16 *(16.49%)* [29, 33, 39, 66, 75, 96, 100, 106, 110-112, 121, 122]	1 *(4.54%)* [96]
	Learning/improvement	7 *(9.21%)* [20, 59, 76, 90, 94, 102, 103]	13 *(13.40%)* [9, 31, 40, 61, 68, 70, 85, 90, 100, 110]	2 *(9.09%)* [29, 123]
	Use of data	7 *(9.21%)* [43, 59, 61, 92, 99]	2 *(2.06%)* [65, 117]	-
	Policy	6 *(7.89%)* [11, 68, 75, 78, 104, 128]	22 *(22.68%)* [9, 11, 29, 30, 32, 33, 40, 46, 58, 68, 75-77, 81, 87, 101, 106, 107]	2 *(9.09%)* [25, 125]
	Management response	5 *(6.58%)* [55, 68, 79, 92, 112]	2 *(2.06%)* [58, 115]	4 *(18.18%)* [29, 97, 115]
	Outcomes of analysis	4 *(5.26%)* [10, 88, 117]	1 *(1.03%)* [100]	-

For peer review only - http://bmjopen.bmj.com/site/about/guidelines.xhtml

	Resource	2 *(2.63%)* [55, 68]	3 *(3.09%)* [25, 75, 127]	1 *(4.54%)* [25]
	Ownership	1 *(1.32%)* [40]	4 *(4.12%)* [25, 52, 125]	6 *(27.27%)* [25, 77]
	Hierarchy	1 *(1.32%)* [36]	-	-
	Size	-	3 *(3.09%)* [25, 26, 49]	1 *(4.54%)* [26]
	Nursing quality	-	1 *(1.03%)* [97]	-
	Awareness	-	1 *(1.03%)* [100]	-
	Location	-	-	1 *(4.54%)* [26]
	Elapsed time of IRS integration	-	-	1 *(4.54%)* [25]
	Ward rounds	-	-	1 *(4.54%)* [25]
	**Total**	**76** *(100%)*	**97** *(100%)*	**22** *(100%)*
Team Factors	Relationships	13 *(39.39%)* [11, 27, 32, 55, 58, 66, 74, 87, 88, 90, 100]	2 *(10.00%)* [49, 82]	-
	Influence of Seniors	7 *(21.21%)* [37, 42, 74, 82, 106, 110]	1 *(5.00%)* [87]	-
	Peer reporting	5 *(15.15%)* [79, 85, 103]	3 *(15.00%)* [97, 98, 101]	-
	Teamwork/communication	3 *(9.09%)* [11, 36, 75]	7 *(35.00%)* [39, 75, 77, 122]	2 *(66.67%)* [123]
	Support/encouragement	3 *(9.09%)* [8, 87, 100]	1 *(5.00%)* [87]	1 *(33.33%)* [72]
	Medical doctor involvement	1 *(3.03%)* [44]	1 *(5.00%)* [44]	-
	Error committed by junior staff	1 *(3.03%)* [58]	1 *(5.00%)* [42]	-
	Team culture	-	4 *(20.00%)* [98, 107, 111, 122]	-
	**Total**	**33** *(100%)*	20 *(100%)*	**3** *(100%)*
Professional Ethics	Concealment	5 *(21.74%)* [85, 87, 120]	1 *(5.88%)* [11]	-
	Duty	1 *(4.35%)* [81]	8 *(47.06%)* [75, 85, 88, 95, 101, 107]	1 *(25.00%)* [125]
	Accountability	-	2 *(11.76%)* [88, 121]	-
	Responsibility	15 *(65.22%)* [8, 9, 34, 35, 44, 52, 70, 93, 94, 100, 104, 118, 121, 128]	5 *(29.41%)* [77, 90, 91, 94]	1 *(25.00%)* [26]
	Culture	2 *(8.70%)* [74, 87]	-	-
	Legal	-	1 *(5.88%)* [37]	2 *(50.00%)* [37]
	**Total**	**23** *(100%)*	**17** *(100%)*	**4** *(100%)*

For peer review only - http://bmjopen.bmj.com/site/about/guidelines.xhtml

PRISMA 2009 Checklist

Section/topic	#	Checklist item	Reported on page #
**TITLE**			
Title	1	Identify the report as a systematic review, meta-analysis, or both.	1
**ABSTRACT**			
Structured summary	2	Provide a structured summary including, as applicable: background; objectives; data sources; study eligibility criteria, participants, and interventions; study appraisal and synthesis methods; results; limitations; conclusions and implications of key findings; systematic review registration number.	2
**INTRODUCTION**			
Rationale	3	Describe the rationale for the review in the context of what is already known.	4
Objectives	4	Provide an explicit statement of questions being addressed with reference to participants, interventions, comparisons, outcomes, and study design (PICOS).	4
**METHODS**			
Protocol and registration	5	Indicate if a review protocol exists, if and where it can be accessed (e.g., Web address), and, if available, provide registration information including registration number.	N/A
Eligibility criteria	6	Specify study characteristics (e.g., PICOS, length of follow-up) and report characteristics (e.g., years considered, language, publication status) used as criteria for eligibility, giving rationale.	5-6
Information sources	7	Describe all information sources (e.g., databases with dates of coverage, contact with study authors to identify additional studies) in the search and date last searched.	5
Search	8	Present full electronic search strategy for at least one database, including any limits used, such that it could be repeated.	5
Study selection	9	State the process for selecting studies (i.e., screening, eligibility, included in systematic review, and, if applicable, included in the meta-analysis).	5-6
Data collection process	10	Describe method of data extraction from reports (e.g., piloted forms, independently, in duplicate) and any processes for obtaining and confirming data from investigators.	6-7
Data items	11	List and define all variables for which data were sought (e.g., PICOS, funding sources) and any assumptions and simplifications made.	6-7
Risk of bias in individual studies	12	Describe methods used for assessing risk of bias of individual studies (including specification of whether this was done at the study or outcome level), and how this information is to be used in any data synthesis.	7
Summary measures	13	State the principal summary measures (e.g., risk ratio, difference in means).	7-8
Synthesis of results	14	Describe the methods of handling data and combining results of studies, if done, including measures of consistency (e.g., I^2) for each meta-analysis.	7-9

For peer review only

PRISMA 2009 Checklist

Section/topic	#	Checklist item	Reported on page #
Risk of bias across studies	15	Specify any assessment of risk of bias that may affect the cumulative evidence (e.g., publication bias, selective reporting within studies).	N/A
Additional analyses	16	Describe methods of additional analyses (e.g., sensitivity or subgroup analyses, meta-regression), if done, indicating which were pre-specified.	N/A
**RESULTS**			
Study selection	17	Give numbers of studies screened, assessed for eligibility, and included in the review, with reasons for exclusions at each stage, ideally with a flow diagram.	9-10
Study characteristics	18	For each study, present characteristics for which data were extracted (e.g., study size, PICOS, follow-up period) and provide the citations.	9-10
Risk of bias within studies	19	Present data on risk of bias of each study and, if available, any outcome level assessment (see item 12).	N/A
Results of individual studies	20	For all outcomes considered (benefits or harms), present, for each study: (a) simple summary data for each intervention group (b) effect estimates and confidence intervals, ideally with a forest plot.	10
Synthesis of results	21	Present results of each meta-analysis done, including confidence intervals and measures of consistency.	12-21
Risk of bias across studies	22	Present results of any assessment of risk of bias across studies (see Item 15).	N/A
Additional analysis	23	Give results of additional analyses, if done (e.g., sensitivity or subgroup analyses, meta-regression [see Item 16]).	N/A
**DISCUSSION**			
Summary of evidence	24	Summarize the main findings including the strength of evidence for each main outcome; consider their relevance to key groups (e.g., healthcare providers, users, and policy makers).	22-25
Limitations	25	Discuss limitations at study and outcome level (e.g., risk of bias), and at review-level (e.g., incomplete retrieval of identified research, reporting bias).	25-26
Conclusions	26	Provide a general interpretation of the results in the context of other evidence, and implications for future research.	26-27
**FUNDING**			
Funding	27	Describe sources of funding for the systematic review and other support (e.g., supply of data); role of funders for the systematic review.	27-28

From: Moher D, Liberati A, Tetzlaff J, Altman DG, The PRISMA Group (2009). Preferred Reporting Items for Systematic Reviews and Meta-Analyses: The PRISMA Statement. PLoS Med 6(7): e1000097. doi:10.1371/journal.pmed1000097

For more information, visit: **www.prisma-statement.org**.

For peer review only - http://bmjopen.bmj.com/site/about/guidelines.xhtml
